# Genetic variants in the inositol phosphate metabolism pathway and risk of different types of cancer

**DOI:** 10.1038/srep08473

**Published:** 2015-02-16

**Authors:** Juan Tan, Chen-Yang Yu, Zhen-Hua Wang, Hao-Yan Chen, Jian Guan, Ying-Xuan Chen, Jing-Yuan Fang

**Affiliations:** 1 State Key Laboratory of Oncogene and Related Genes, Key Laboratory of Gastroenterology & Hepatology, Ministry of Health, Division of Gastroenterology and Hepatology, Ren Ji Hospital, School of Medicine, Shanghai Jiao Tong University, Shanghai Cancer Institute, Shanghai Institution of Digestive Disease, 145 Middle Shandong Rd, Shanghai 200001, China; 2 Department of Otolaryngology, The Affiliated Sixth People's Hospital, Otolaryngology Institute of Shanghai Jiao Tong University, Shanghai 200233, China

## Abstract

Members of the inositol phosphate metabolism pathway regulate cell proliferation, migration and phosphatidylinositol-3-kinase (PI3K)/Akt signaling, and are frequently dysregulated in cancer. Whether germline genetic variants in inositol phosphate metabolism pathway are associated with cancer risk remains to be clarified. We examined the association between inositol phosphate metabolism pathway genes and risk of eight types of cancer using data from genome-wide association studies. Logistic regression models were applied to evaluate SNP-level associations. Gene- and pathway-based associations were tested using the permutation-based adaptive rank-truncated product method. The overall inositol phosphate metabolism pathway was significantly associated with risk of lung cancer (P = 2.00 × 10^−4^), esophageal squamous cell carcinoma (P = 5.70 × 10^−3^), gastric cancer (P = 3.03 × 10^−2^) and renal cell carcinoma (P = 1.26 × 10^−2^), but not with pancreatic cancer (P = 1.40 × 10^−1^), breast cancer (P = 3.03 × 10^−1^), prostate cancer (P = 4.51 × 10^−1^), and bladder cancer (P = 6.30 × 10^−1^). Our results provide a link between inherited variation in the overall inositol phosphate metabolism pathway and several individual genes and cancer. Further studies will be needed to validate these positive findings, and to explore its mechanisms.

The worldwide burden of cancer continues to grow, especially in developing countries^1^. However, the etiology of this highly lethal disease remains largely unclear. Numerous researchers have showed that genetic factors play a vital role in an individual's risk of cancer. Recent genome-wide association studies (GWAS) have established many chromosome regions as susceptibility loci for different types of cancer^2^,^3^,^4^. However, it is supposed that these studies illustrate only a small part of the heritability^5^. Thus, as a complementary approach, pathway-based analysis of GWAS data was appealing to identify signaling pathways or genes abundant with disease-related single nucleotide polymorphisms (SNPs) whose single effects may be too small to be found by traditional single-locus methods^6^,^7^.

Members of the inositol phosphate metabolism pathway contain inositol and its associated factors, such as inositol phosphates, phosphoinositides (PI), the enzymes they need for synthesis and transformation, and so on. They play crucial roles in normal physiological conditions, such as insulin signaling, PI3K/Akt signaling, endocytosis, vesicle trafficking, cell migration, proliferation, apoptosis and maintaining the state of homeostasis for many second messages^8^. In addition, the mechanism of phosphatidylinositol-3-kinase (PI3K)-dependent Akt activation is a paradigm of PI-dependent activation of signaling cascades, and, deregulation of PI3K-dependent signaling pathways is linked to the development of many cancers. Lots of oncogenes, such as *K-Ras* and *Her2*, promote tumor growth by activating the PI3K pathway^9^,^10^,^11^. Furthermore, metabolic reprogramming may make cancer more aggressive, such as promoting both tumor growth and invasiveness^12^,^13^,^14^.

Given the significance of this pathway, its tight deregulation may disrupt homeostasis and promote tumorigenesis. Genetic variations of the inositol phosphate metabolism pathway proteins could correlate with cancer predisposition. Although numerous studies have already investigated the role of related SNPs in cancer, the coverage of genes was limited and the sample sizes were relatively small^15^,^16^,^17^,^18^,^19^,^20^,^21^. Therefore, we evaluated associations between inositol phosphate metabolism genes and the risk of eight different types of cancer (lung cancer, bladder cancer, esophageal squamous cell carcinoma (ESCC), gastric cancer (GC), prostate cancer, breast cancer, renal cell carcinoma (RCC) and pancreatic cancer), using a comprehensive pathway-based analysis of the first phase of GWAS available in dbGAP database(www.ncbi.nlm.nih.gov/gap). Our results suggested that the overall inositol phosphate metabolism pathway may be associated with four different types of cancer development.

## Results

### Association of cancer risk with individual SNPs

The SNPs with P < 0.001 are shown in Supplementary Table SI. Our results showed that SNPs in this pathway have not reached genome-wide significance except SNP of PLCE1 for ESCC/GC, which was consistent with the original GWAS for each study. For lung cancer, we found 3 SNPs across three inositol phosphate metabolism genes with P < 0.001, including rs13021302 (*INPP5D*), rs11083841 (*CALM3*), and rs11668501 (*ITPKC*) (Supplementary Table SI). For ESCC, seven SNPs in *PLCE1* were significantly related with ESCC risk, exceeding the Bonferroni-corrected threshold, which was previously identified by the initial GWAS; and a further five SNPs in *INPP4B* (rs336407, rs336298, rs3775692 and rs336332) and *INPP5A* (rs10747068) with P < 0.001 (Supplementary Table SI). For GC, seven SNPs in *PLCE1* were significantly associated with GC risk, exceeding the Bonferroni-corrected threshold, which was previously identified by the initial GWAS, as with ESCC; and one SNP in *ITPKB* (rs3754378) with P < 0.001 (Supplementary Table SI). The seven SNPs in *PLCE1* associated with ESCC and GC were in high LD (r^2^ ≥ 0.8) with each other, representing an independent signal. For RCC, no SNP reached the Bonferroni-corrected significance level, but 5 SNPs across three inositol phosphate metabolism genes (*IP6K1, IP6K2, PLCB1*) were found to have a statistical significance at a significant level of 0.001. In pancreatic cancer, rs11922130 and rs9861030 across gene *PLCD1* and rs11044171 across *PIK3C2G* were associated with cancer risk (P < 0.001). However, no SNP achieved the significance level of 0.001 for breast cancer, prostate cancer and bladder cancer.

### Association of cancer risk with individual genes

**Figure 1:**
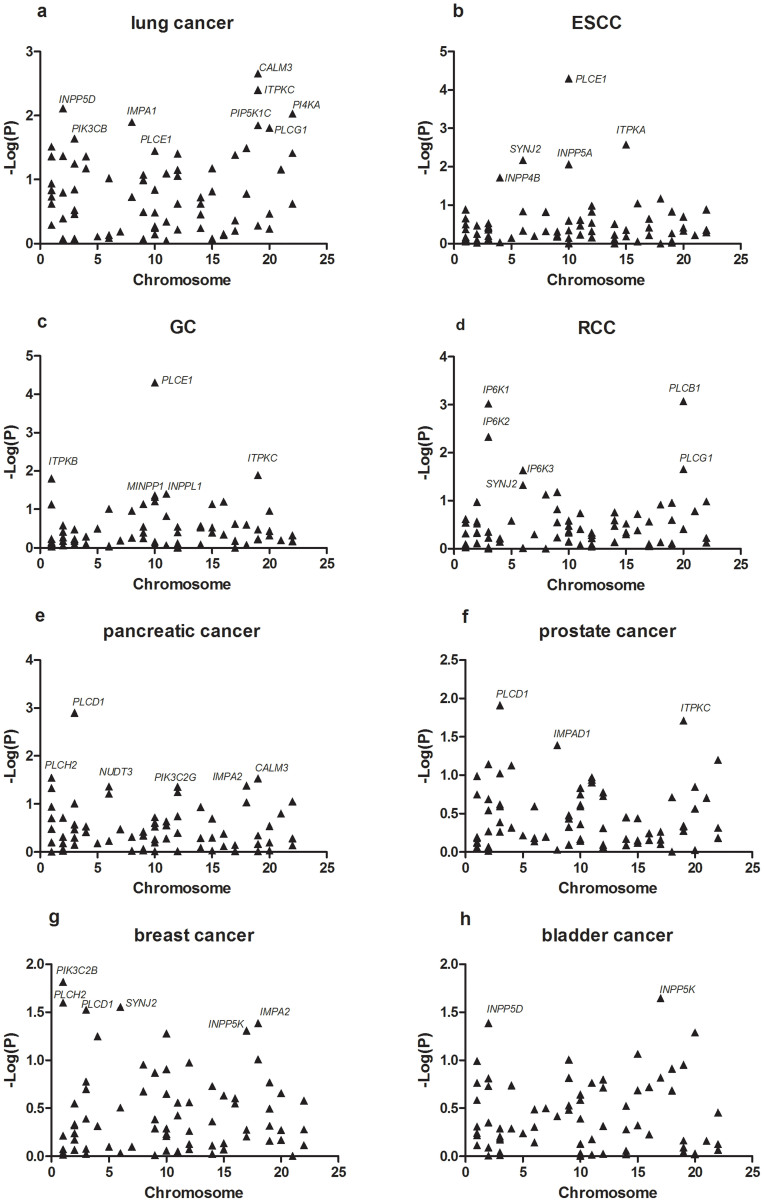
The associations between inositol phosphate metabolism pathway genes and risk of eight different types of cancer. (a) lung cancer; (b) ESCC; (c) GC; (d) RCC; (e) pancreatic cancer; (f) prostate cancer; (g) breast cancer; (h) bladder cancer. For clarity, not all genes are labeled. Details can be found in Table 1 and Supplementary Table SII.

Gene-level analysis was conducted among the inositol phosphate metabolism pathway associated genes. We identified 17 genes that were significantly associated with lung cancer risk (P < 0.05; Table 1 and Figure 1), among which *CALM3* showed the most significance (P = 0.0022). For ESCC and GC, *PLCE1* showed the strongest association with a significance level (P = 5.00 × 10^−5^) that exceeded the Bonferroni-corrected threshold; a further four genes: *ITPKA*, *SYNJ2*, *INPP5A* and *INPP4B* were significantly associated with ESCC (P < 0.05); and five additional genes: *ITPKC*, *ITPKB*, *INPPL1*, *MINPP1* and *INPP5A* were significantly associated with GC (P < 0.05; Table 1 and Figure 1). Six genes were significantly associated with risk of RCC: *PLCB1, IP6K1, IP6K2, PLCG1 IP6K3 AND SYNJ2* (P < 0.05; Table 1 and Figure 1), none of which exceeded the Bonferroni-corrected threshold. In pancreatic cancer, there were seven genes achieved the significance level of 0.05. We observed three genes and six genes significantly associated with risk of prostate cancer and breast cancer with P < 0.05, respectively. And two genes showed to be significant in bladder cancer (P < 0.05; Table 1 and Figure 1).

### Association of cancer risk with the overall pathways

The pathway-level analysis was conducted using the ARTP method. Of the eight different types of cancer analyzed (Table 2), the most statistically significant association was seen for lung cancer (P = 2.00 × 10^−4^). Three other types of cancer were significant: ESCC (P = 5.70 × 10^−3^), GC (P = 3.03 × 10^−2^) and RCC (P = 1.26 × 10^−2^), but not with pancreatic cancer (P = 1.40 × 10^−1^), breast cancer (P = 3.03 × 10^−1^), prostate cancer (P = 4.51 × 10^−1^), and bladder cancer (P = 6.30 × 10^−1^). However, after excluding genes (i.e. removing all SNPs within the gene) previously identified by the initial GWAS (*PLCE1* from GC and ESCC), the association was not significant for either ESCC (P = 0.13) or GC (P = 0.27).

## Discussion

Somatic mutations and deregulation of inositol phosphate metabolism genes, such as *PTEN* or *PIK3CA*, are associated with cancer development and progression, including brain, colon, breast, prostate and hepatocellular cancers^9^,^10^,^22^,^23^,^24^,^25^. Until now, it is unclear whether germline genetic variants in the inositol phosphate metabolism pathway are involved with the development of cancer. Here, our pathway-based analysis of GWAS data has shown that common germline variations in the inositol phosphate metabolism genes may be important susceptibility factors for cancer. The most statistically significant association between genetic variants in this pathway and risk of cancer was observed for lung cancer. Three other types of cancer (ESCC, GC and RCC) showed nominally significant associations (P < 0.05). Rather than germline genetic polymorphisms in candidate inositol phosphate metabolism genes that have been reported before (e.g., *PTEN, PIK3CA and INPP4B*), the present study greatly extends the coverage of the pathway-related genes and observed novel significant associations between genetic variants of the pathway-related genes and risk of cancer^15^,^16^,^17^,^18^,^19^,^21^.

As far as we know, the present study is the first to examine the role of genetic variation in inositol phosphate metabolism genes and risk of upper gastrointestinal (UGI) cancers in a high-risk Chinese population and of lung cancer and RCC in a European population. Previous single pathway analyses found that genetic variants in several signaling pathway were associated with UGI cancer in a high-risk population in north central China, including epidermal growth factor receptor signaling and GC risk, Fas signaling pathway and GC risk as well as DNA repair pathway and UGI cancers risk^26^,^27^,^28^. However, few fractions of overlap between those pathways mentioned above and the inositol phosphate metabolism pathway were found, moreover, no study has focused on inositol phosphate metabolism pathway in UGI cancers. A recent pathway-based analysis in the Korean Non-Small Cell Lung Cancer Study showed that inositol phosphate metabolism had significant statistics, and our observation of associations between genetic variants of this pathway and lung cancer in a European population provides additional evidence for this metabolism^29^. For bladder cancer, prostate cancer, breast cancer and pancreatic cancer, the same databases were used to conduct a pathway-based analysis in which inositol phosphate metabolism showed no significant associations^30^,^31^,^32^,^33^. However, the number of genes related to this pathway in the four studies mentioned above was small, and only 54 genes collecting from KEGG were included^30^,^31^,^32^,^33^. In our study, the number of pathway-related genes increased to 76 based on two publicly available pathway resources (KEGG and REACTOME), and the associations were still not significant between the inositol phosphate metabolism pathway and those four types of cancer.

Our gene-based analysis highlighted 17 lung cancer susceptibility genes, of which the most significant was *CALM3* encoding calmodulin, which also significantly associated with pancreatic cancer risk. Calmodulin, a ubiquitous, highly conserved intracellular Ca^2+^ sensor of 17 kDa, mediates many of the actions of Ca^2+^ involved in the regulation of a wide variety of cellular events. The T > A polymorphism at position −34 (−34T > A) in the promoter region of the human *CALM3*, which could result in differential regulation of the transcription of the *CALM3* gene, was differently distributed between familial hypertrophic cardiomyopathy patients and controls^34^. Nevertheless, little is known about the functions and cellular mechanisms of *CALM3* in lung cancer. Further studies are now needed to confirm the association and explore the underlying biological mechanisms in cancer.

Common germline variations in inositol phosphate metabolism were significantly associated with GC and ESCC risk in our study. Gene *PLCE1* contributes to the strongest gene-based association with ESCC and GC risk, which was previously identified by the initial GWAS^2^. We identified seven significant SNPs in *PLCE1* (P < 0.001) in strong LD (r^2^ ≥ 0.80), which represented an independent signal associated with the risk of ESCC and GC. In addition, we found that this gene also associated with lung cancer (P = 0.036, Table 1). This notion is supported by a meta-analysis of 13 case-control studies, including more than 11 000 subjects, which showed that the *PLCE1* rs2274223 polymorphism was statistically significant associated with an increasing risk of ESCC and GC^20^. *PLCE1* encodes a phospholipase enzyme that hydrolyzes phosphatidylinositol 4,5-bisphosphate(PtdIns-4,5-P2; PIP2) to produce inositol 1,4,5-trisphosphate (InsP3; IP3) and diacylglycerol(DAG), thus triggering a signaling cascade resulting in gene expression, cell growth and differentiation. Recent reports indicate that PLCE1 is a pivotal molecule involved in the pathogenesis of several cancers, including esophageal, gastric, skin, bladder, lung and colorectal cancer^2^,^20^,^35^,^36^,^37^,^38^. However, the role of PLCE1 in the pathogenesis of these cancers has not yet been fully clarified and was inconsistent in different cancer. *PLCE1* could be a cancer suppressor for sporadic colorectal cancer, based on the low level of PLCE1 found in human sporadic colorectal cancer tissue in comparison to that of non-small-cell lung cancer (NSCLC) where *PLCE1* expression is high^35^,^36^.

In this study, we found that six genes contributed to higher risks for RCC, of which *PLCB1* in 20p12 was the most significant (P = 0.00085). We identified two significant SNPs in *PLCB1* (P < 0.001) in weak LD (r^2^ < 0.20), which represented two independent signal associated with the risk of RCC. The variant allele of rs4813865, which is an intronic polymorphism (T > G), was associated with the risk of RCC (per allele OR: 0.81, 95% CI: 0.74–0.90, P = 4.30 * 10^−5^), as was the variant G allele of rs2223538 which is also an intronic polymorphism (T > G; per allele OR: 1.26, 95% CI: 1.12–1.42, P = 0.00016). The PLC*β*1 protein, a key enzyme in nuclear signal transduction among the enzymes of the inositol lipid cycle, catalyzes the formation of IP3 and DAG from PIP2 and participates in G protein coupled receptor (GPCR)-mediated signaling^39^. Altered expression of nuclear PLCβ1 could be involved in many cellular processes such as proliferation, differentiation and cell apoptotic pathways^40^. Recently, *PLCB1* was identified as a tumor suppressor gene in head and neck cancer^41^. Given that *PLCB1* is one of the key regulators in signal transduction or an important tumor suppressor genes, it is possible that one or more of these SNPs may change the expression of PLCβ1 or modify protein interactions that might manipulate the development of cancer. However, the function and mechanism of action of *PLCB1* in RCC is unknown.

The lack of a pathway-based association for the overall inositol phosphate metabolism pathway with pancreatic, prostate, breast and bladder cancer, may reflect the complex process of occurrence and development of tumors and the small role of genetic variants in inositol phosphate metabolism playing in the pathogenesis of those four types of cancer. Alternatively, it may reflect differences in the multiple pathogenic mechanisms and in the complex risk factors between tumors. However, significant associations for several individual genes in this pathway were found in those four cancers. The strongest gene-based association was *PLCD1* for pancreatic cancer and prostate cancer, *PIK3C2B* for breast cancer, and *INPP5K* for bladder cancer, respectively. *PLCD1* also contributed to the risk of breast cancer. *PLCD1* encodes a protein phospholipase C delta 1, which functions as a tumor suppressor in several types of cancer, including ESCC, GC, breast cancer and colorectal cancer^42^,^43^,^44^,^45^ and plays a role in regulating cell cycle progression^46^. Consistently, our observation of associations between *PLCD1* and breast cancer further proved its carcinogenic potential. *PIK3C2B* codes for the class II PI3K enzyme PIK3C2β, which could regulate cell migration and proliferation^47^,^48^. Thus, genetic variants in *PIK3C2B* may alter PIK3C2β expression, influencing the migration and survival of tumor cells. *INPP5K*, an inositol polyphosphate 5-phosphatase, can play a role in the regulation of insulin signaling, glucose transport and actin cytoskeletal rearrangement^49^,^50^,^51^. Although *INPP5K* was the most significantly gene associated with bladder cancer, its convincingness was relatively weak because of its number of SNPs in this gene for bladder cancer. Also, these findings will need to be verified in future studies.

Here, instead of one-by-one SNP analysis, we used a resampling-based ARTP method to systematically study associations between inositol phosphate metabolism and the risk of eight different types of cancer, which would provide new biological prospective and highlight additional candidate loci of complex diseases^6^,^7^. The relatively large sample size made the results more convincing. In addition to our comprehensive assessment of both gene- and pathway-associations, the examination of a large number of SNPs involved in inositol phosphate metabolism is another important advantage of our study.

Several limitations of our study should be taken into account. First, we had no information on environmental factors for cancer, such as smoking, *Helicobacter pylori* (*H. pylori*) infection, other lifestyle and dietary factors, etc. However, the distribution of these risk factors for cancer was found to be independent of the genetic variants. Previous GWAS of lung cancer and bladder cancer showed that those SNPs with P < 10^−7^ did not make a material change of genetic effects after additional adjustment for smoking, suggesting that the association of SNPs with risk of these two cancers is not entirely explained by the association with smoking^3^,^4^. In addition, although there is strong evidence that *H. pylori* played a role in the development of GC, the high prevalence of *H. pylori* infection in both GC cases and the matched controls indicated that our results may be less likely to be distorted by the lack of this environmental factor^27^. However, we still cannot completely rule out the residual confoundings by smoking or other environmental factors, further information and studies are required to confirm these associations. Second, our selection of inositol phosphate metabolism genes could be limited. We may miss genes because the annotation of the human genome is incomplete, and those unknown genes couldn't be assigned to this pathway. Third, the significance thresholds were comparatively less stringent for SNPs than the GWAS significance criteria (P = 5 * 10^−7^). However, the genome-wide significant criteria may be overly conservative for detecting modest associations and the significance thresholds used in the present study has been applied in many other articles about pathway-based analysis of GWAS data^26^,^27^,^28^,^52^,^53^. Fourth, further functional experiments are needed to clarify the mechanisms underlying the new findings between the genetic variants and risk of cancer because our study was just an association study.

In conclusion, our pathway-based analysis identified the germline genetic variations of the overall inositol phosphate metabolism pathway and several individual genes that are associated with the risk of lung, UGI cancers and RCC, as well as individual genes that are related with pancreatic, prostate, breast and bladder cancer risk, suggesting that inositol phosphate metabolism pathway genes are involved in the occurrence and progression of different types of cancer. Confirmation of these results in other independent databases, combined with advanced knowledge about the cellular mechanisms underlying these positive findings, is now demanded to solidify our findings. Our study, therefore, may open up new research avenues for future studies on the pathogenesis of these cancers.

## Methods

### Identification of eligible studies

After the exclusion criteria were applied, we evaluated genetic variants of the inositol phosphate metabolism pathway and (1) the risk of lung cancer in 3779 cases and 3837 controls from the Maryland lung cancer study (dbGAP number: phs000336; consent group: cancer in all age groups, other diseases in adults only, and methods(CADM)) performed in the United States^3^; (2) the risk of gastric adenocarcinoma and ESCC in a study (dbGAP number: phs000361) on a Chinese population, including 1625 cases of gastric cancer, 1896 cases of ESCC and 2097 controls^2^; (3) the risk of RCC in 1311 cases and 3424 controls of European origin (dbGAP number: phs000351)^54^; (4) the risk of pancreatic cancer in 2452 affected individuals and 2461 unaffected controls (dbGAP number: phs000206; consent group: CADM) on a European population^55^; (5) the risk of prostate cancer in a nested case-control study (dbGAP number: phs000207) including 1147 cases and 1098 controls of European background^56^; (6) the risk of breast cancer in 1144 postmenopausal women of European descent with invasive breast cancer and 1141 controls (dbGAP number: phs000147)^57^; and (7) the risk of bladder cancer in 3495 cases and 5101 controls (dbGAP number: phs000361) performed in a European population^4^ (Table 2).

This study is based on an in-silicon re-analysis of the human genotyping data downloaded from dbGAP (www.ncbi.nlm.nih.gov/gap). The informed consent of each participant was obtained by the researchers submitting the data.

### Gene and SNP selection for the inositol phosphate metabolism pathway

We identified the gene in our analysis if it was referenced in at least one of the databases as follows: inositol phosphate metabolism in KEGG (http://www.genome.jp/dbget-bin/www_bget?pathway:map00562, retrieved on 5 May 2014) and inositol phosphate metabolism in REACTOME (http://www.reactome.org/PathwayBrowser/#DIAGRAM=1483249&PATH=1430728, retrieved on 5 May 2014). There is no pathway data for inositol phosphate metabolism in BioCarta and the NCI Pathway Interaction Database. Seventy-six genes were recognized in the inositol phosphate metabolism pathway. SNPs located in the respective gene and within the 20 kb upstream or 10 kb downstream of the gene, with a minor allele frequency(MAF) of 5% (in cases and controls combined), were included in our analysis. Some SNPs located between genes were counted twice because of the overlap between genes' flanking area. We excluded four genes(MTM1, *NUDT10, NUDT11, OCRL*) located on the X chromosome. After quality control filters, SNPs mapping to three genes (*IMPA1*, *MINPP1* and *PIP4K2B*) in bladder cancer were not found, leaving 69 genes in bladder cancer analysis (Table 2). The full list of these genes is shown in Supplementary Table SII.

### Quality control

DNAs were genotyped as part of the GWAS as described previously^2^,^3^,^4^,^57^. Data are available upon request from the NIH Data Access Committee. We used the same criteria for the different data sets. We excluded SNPs with a call rate of <90%; SNPs with MAF <5% (in cases and controls combined); SNPs deviating from the Hardy–Weinberg equilibrium (P < 0.0001, in controls); subjects with a completion rate of all SNPs < 94%; and gender discordant subjects or unexpected duplicate pairs. After these exclusion criteria were applied, 1421 SNPs in the inositol phosphate metabolism pathway genes remained for lung cancer analysis; 1352 SNPs for ESCC; 1350 SNPs for GC; 1524 SNPs for RCC; 1613 SNPs for pancreatic cancer; 1535 SNPs for prostate cancer; 1747 SNPs for breast cancer; and 610 SNPs for bladder cancer (Table 2; Supplementary Table SIII). Linkage disequilibrium (LD) was further computed between any two SNPs in the same chromosome among the controls.

### Statistical analyses

Principal component analysis (PCA) for each study group was performed with the use of the EIGENSTRAT program to account for potential population stratification or admixture in these samples^58^. No evidence for obvious problems with population stratification was found in UGI, prostate and bladder cancer, so we did not consider population substructure in those three studies^2^,^4^,^56^. The same number of eigenvectors obtained from PCA analysis as the original GWAS for each study was included as covariates in logistic regression models.

For each SNP, odds ratios (ORs) and 95% confidence intervals (CIs) for one minor allele were calculated using unconditional logistic regression in an additive model, adjusting for age, gender, and/or study/principal components of population stratification (Table 2). A Bonferroni-corrected significance threshold was calculated from 1421 SNPs for lung cancer (P = 3.52 × 10^−5^, 0.05/1421 SNPs); 1352 SNPs (P = 3.70 × 10^−5^, 0.05/1352 SNPs) for ESCC; 1350 SNPs (P = 3.70 × 10^−5^, 0.05/1350 SNPs) for GC; 1524 SNPs for RCC (P = 3.28 × 10^−5^, 0.05/1524 SNPs); 1613 SNPs for pancreatic cancer (P = 3.10 × 10^−5^, 0.05/1613 SNPs); 1535 SNPs for prostate cancer (P = 3.26 × 10^−5^, 0.05/1535 SNPs); 1747 SNPs (P = 2.86 × 10^−5^, 0.05/1747 SNPs) for breast cancer; and 610 SNPs for bladder cancer (P = 8.20 × 10^−5^, 0.05/610 SNPs). Because only a few SNPs reached the Bonferroni-corrected significance level, statistical significance for SNP-level analyses was defined as P < 0.001.

We performed gene-level associations using the adaptive rank truncated product (ARTP) approach, which adaptively combines single SNP *p*-values within each gene region to obtain a single test statistic for the gene and assess significance of the test via a permutation-based sampling procedure (20 000 resamplings)^6^. We also conducted pathway analysis to evaluate the association between a set of candidate genes included in the overall inositol phosphate metabolism pathway and cancer risk. Using the ARTP method with 20 000 resamplings, we obtained a single test statistic for the overall pathway for each type of cancer. For gene- and pathway-based analyses, statistical significance was declared if P value was <0.05. In addition, a more stringent Bonferroni-corrected significance threshold for gene-based analysis was performed to account for testing 72 genes (P = 6.94 × 10^−4^, 0.05/72 genes), except bladder cancer (P = 7.25 × 10^−4^, 0.05/69 genes). Statistical analyses were performed using R language and Plink v1.07.

## Author Contributions

J.T. and C.Y.Y. contribute equally to the work. J.T. and Y.X.C. designed and supervised the project. J.G. and H.Y.C. performed the material preparation. J.T. and C.Y.Y. analysed the data. J.T., Y.X.C., Z.H.W. and J.Y.F. wrote the manuscript. All authors reviewed the manuscript.

## Supplementary Material

Supplementary Information Supplementary Table SI, SII, SIII

## Figures and Tables

**Table 1:** Gene- and most significant SNP-based P-values for inositol phosphate metabolism pathway genes and risk of cancer

Types of cancer (Pathway P*)	*Gene*	Chr. (cytoband)	Gene-level P^g^	No. of SNPs	Most significant SNP	Associated SNP P^n^
**Lung cancer**						
(2.00E-04)	*CALM3*	19q13	2.20E-03	5	rs11083841	3.68E-04
	*ITPKC*	19q13	3.95E-03	8	rs11668501	5.90E-04
	*INPP5D*	2q37	7.70E-03	50	rs13021302	2.07E-04
	*PI4KA*	22q11	9.35E-03	10	rs2072513	1.24E-03
	*IMPA1*	8q21	1.26E-02	1	rs1967328	1.18E-02
	*PIP5K1C*	19p13	1.42E-02	8	rs2270083	2.10E-03
	*PLCG1*	20q12-q13	1.55E-02	4	rs2235360	4.96E-03
	*PIK3CB*	3q22	2.27E-02	8	rs531577	5.89E-03
	*PIK3CD*	1p36.2	3.06E-02	8	rs12075554	4.34E-03
	*PIK3C3*	18q12	3.23E-02	8	rs3764459	6.79E-03
	*PLCE1*	10q23	3.56E-02	57	rs11187842	1.04E-03
	*INPP5J*	22q12	3.85E-02	19	rs4820944	2.54E-03
	*PIP4K2C*	12q13	3.95E-02	4	rs775250	1.22E-02
	*INPP5K*	17p13	4.10E-02	10	rs1879488	6.00E-03
	*PIKFYVE*	2q34	4.25E-02	13	rs10189031	8.82E-03
	*PI4KB*	1q21	4.30E-02	7	rs5022636	1.34E-02
	*PI4K2B*	4p15	4.31E-02	7	rs3115231	9.19E-03
**ESCC**						
(5.70E-03)	*PLCE1*	10q23	5.00E-05	60	rs3765524	5.55E-08
	*ITPKA*	15q15	2.60E-03	2	rs2305030	1.50E-03
	*SYNJ2*	6q25	6.60E-03	36	rs2025641	3.00E-04
	*INPP5A*	10q26	8.60E-03	28	rs10747068	5.00E-04
	*INPP4B*	4q31	1.89E-02	109	rs336407	3.00E-04
**GC**						
(3.03E-02)	*PLCE1*	10q23	5.00E-05	60	rs3781264	4.18E-11
	*ITPKC*	19q13	1.28E-02	7	rs890934	2.40E-03
	*ITPKB*	1q42	1.56E-02	25	rs3754378	1.00E-03
	*INPPL1*	11q13	3.93E-02	5	rs7110260	9.00E-03
	*MINPP1*	10q23	4.32E-02	13	rs3843597	5.30E-03
	*INPP5A*	10q26	4.65E-02	28	rs7091957	3.20E-03
**RCC**						
(1.26E-02)	*PLCB1*	20p12	8.50E-04	248	rs4813865	4.30E-05
	*IP6K1*	3p21	9.50E-04	5	rs6802890	1.97E-04
	*IP6K2*	3p21	4.70E-03	6	rs3172494	9.78E-04
	*PLCG1*	20q12-q13	2.20E-02	7	rs6129760	4.59E-03
	*IP6K3*	6p21	2.31E-02	26	rs471942	1.03E-03
	*SYNJ2*	6q25	4.74E-02	40	rs12663163	1.76E-03
**Pancreatic cancer**						
(1.40E-01)	*PLCD1*	3p22-p21	1.25E-03	8	rs11922130	1.79E-04
	*PLCH2*	1p36	2.84E-02	10	rs7535528	3.26E-03
	*CALM3*	19q13	2.91E-02	6	rs7258489	5.56E-03
	*IMPA2*	18p11	4.13E-02	25	rs1262056	2.13E-03
	*NUDT3*	6p21.2	4.30E-02	6	rs206937	1.19E-02
	*PIK3C2G*	12p12	4.34E-02	86	rs11044171	9.79E-04
	*ITPKB*	1q42.13	4.62E-02	26	rs10916019	2.62E-03
**Prostate cancer**						
(4.51E-01)	*PLCD1*	3p22-p21	1.23E-02	7	rs4389435	2.14E-03
	*ITPKC*	19q13	1.94E-02	7	rs3865451	3.90E-03
	*IMPAD1*	8q12.1	4.06E-02	7	rs13257046	6.65E-03
**Breast cancer**						
(3.04E-01)	*PIK3C2B*	1q32	1.53E-02	17	rs2271421	6.59E-03
	*PLCH2*	1p36	2.52E-02	11	rs2236395	2.57E-03
	*SYNJ2*	6q25	2.80E-02	44	rs2295893	8.78E-04
	*PLCD1*	3p22-p21	2.97E-02	17	rs137625	3.51E-03
	*IMPA2*	18p11	4.12E-02	26	rs684680	2.24E-03
	*INPP5K*	17p13	4.41E-02	11	rs7214615	6.49E-03
**Bladder cancer**						
(6.63E-01)	*INPP5K*	17p13	2.25E-02	1	rs2270227	2.35E-02
	*INPP5D*	2q37	4.09E-02	24	rs10933435	2.67E-03

Gene-based p-values (P^g^) are shown in order of lowest to highest p-value. Only genes with P^g^ < 0.05, are shown in table. The most significant SNP (nonminal p -value (P^n^)) in each gene is indicated.

Abbreviations: GC, gastric carcinoma; ESCC, esophageal squamous cell carcinomas; RCC: renal cell carcinoma; Chr, chromosome; SNP, single nucleotide polymorphism.

**Table 2:** Characteristics and pathway-based P-values for inositol phosphate metabolism in eight different types of cancer

Types of cancer	Reference number of dbGaP	Ancestry	Case	Control	No. of genes	No. of SNPs	Adjustment factors	Study type
Lung cancer	phs000336	European	3779	3837	72	1421	Age(5 y-intervals), sex, study, 3 PCs*	Three cohort studies
ESCC	phs000361	Asian	1896	2097	72	1352	Age(10 y-intervals), sex, study	One cohort study and one case-control study
GC	phs000361	Asian	1625	2097	72	1350	Age(10 y-intervals), sex, study	One cohort study and one case-control study
RCC	phs000351	European	1311	3424	72	1524	Sex, study, 2 PCs*	Three cohort studies and one case-control study
Panreatic cancer	phs000206	European	2452	2461	72	1613	Age(10 y-intervals), sex, study, 5 PCs*	Fourteen cohort studies and two case-control studies
Prostate cancer	phs000207	European	1147	1098	72	1535	Age(10 y-intervals), family history	One case-control study
Breast cancer	phs000147	European	1144	1141	72	1747	Age(10 y-intervals), 3 PCs*	One case-control study
Bladder cancer	phs000346	European	3495	5101	69^a^	610	Age(5 y-intervals), sex, study, 5 PCs*	Two case-control studies and three cohort studies

^a^In bladder cancer, SNPs mapping to three genes (*IMPA1*, *MINPP1* and *PIP4K2B*) were not found after quality control filters, leaving 69 genes in bladder cancer analysis.

*The number of significant principal components (PCs) in each study was based on the original GWAS for each study.

Abbreviations: GC, gastric carcinoma; ESCC, esophageal squamous cell carcinomas; RCC: renal cell carcinoma.
