# Supplementary material for: Genetic variants in the inositol phosphate metabolism pathway and risk of different types of cancer
Source: Sci Rep. 2015 Feb 16;5:8473. doi: 10.1038/srep08473 (PMC4329558; doi:10.1038/srep08473)
Supplement: Supplementary Information — Supplementary Table SI, SII, SIII [file srep08473-s1.pdf]

Genetic variants in the inositol phosphate metabolism pathway and risk of different types of cancer

Juan Tan [1,*], Chen-Yang Yu [1,*], Zhen-Hua Wang [1] , Hao-Yan Chen [1], Jian Guan [2] , Ying-Xuan Chen [1, #] & Jing-Yuan Fang [1]

Supplementary Table SI. SNPs associated with risk of cancer with P<0.001. [a]

SNP	Chr.(cytoband)	*Gene*	OR	L95	U95	P value
**Lung cancer**						
rs13021302	2	*INPP5D*	1.244	1.109	1.396	2.07E-04
rs11083841	19	*CALM3*	1.168	1.072	1.272	3.68E-04
rs11668501	19	*ITPKC*	1.16	1.066	1.263	5.90E-04
**ESCC**						
rs336407	4	*INPP4B*	1.179	1.078	1.289	3.06E-04
rs336298	4	*INPP4B*	1.175	1.074	1.284	4.03E-04
rs3775692	4	*INPP4B*	1.193	1.08	1.319	5.37E-04
rs336332	4	*INPP4B*	1.168	1.068	1.276	6.46E-04
rs2025641	6	*SYNJ2*	1.246	1.106	1.404	2.86E-04
rs3765524	10	*PLCE1*	1.344	1.208	1.496	5.55E-08
rs2274223	10	*PLCE1*	1.34	1.205	1.49	7.13E-08
rs3781264	10	*PLCE1*	1.38	1.227	1.553	8.56E-08
rs12263737	10	*PLCE1*	1.336	1.201	1.486	9.67E-08
rs753724	10	*PLCE1*	1.374	1.22	1.549	1.82E-07
rs11187842	10	*PLCE1*	1.375	1.22	1.55	1.84E-07
rs3740360	10	*PLCE1*	1.351	1.198	1.523	9.15E-07
rs10747068	10	*INPP5A*	1.259	1.106	1.433	4.79E-04
**GC**						
rs3754378	1	*ITPKB*	0.8368	0.7527	0.9303	9.81E-04
rs3781264	10	*PLCE1*	1.51	1.336	1.706	4.18E-11
rs753724	10	*PLCE1*	1.489	1.316	1.686	2.85E-10
rs11187842	10	*PLCE1*	1.488	1.314	1.684	3.44E-10
rs3740360	10	*PLCE1*	1.479	1.306	1.674	7.28E-10
rs2274223	10	*PLCE1*	1.398	1.253	1.56	1.99E-09
rs3765524	10	*PLCE1*	1.398	1.252	1.56	2.23E-09
rs12263737	10	*PLCE1*	1.385	1.241	1.545	5.61E-09
**RCC**						
rs6802890	3	*IP6K1*	1.203	1.091	1.326	1.97E-04
rs9855505	3	*IP6K1*	1.199	1.088	1.321	2.57E-04
rs3172494	3	*IP6K2*	0.7866	0.682	0.9073	9.78E-04
rs4813865	20	*PLCB1*	0.8148	0.7386	0.8988	4.30E-05
rs2223538	20	*PLCB1*	1.259	1.117	1.419	1.55E-04
**Pancreatic cancer**						
rs11922130	3	*PLCD1*	1.404	1.176	1.677	1.79E-04
rs9861030	3	*PLCD1*	1.359	1.133	1.63	9.66E-04
rs11044171	12	*PIK3C2G*	0.8339	0.7485	0.929	9.79E-04

[a] SNPs were listed in Chromosomal order.

Supplementary Table SII. The associations between all inositol phosphate metabolism pathway genes and risk of cancers.

Gene [a]	Lung cancer*		ESCC		GC		RCC	
	N.SNP	P value	N.SNP	P value	N.SNP	P value	N.SNP	P value
ALDH6A1	3	2.40E-01	23	9.89E-01	23	2.84E-01	4	7.30E-01
CALM1	8	3.48E-01	7	3.08E-01	7	2.94E-01	8	1.75E-01
CALM2	8	4.03E-01	7	3.38E-01	7	6.65E-01	10	4.58E-01
CALM3	5	2.20E-03	5	5.43E-01	5	5.86E-01	6	7.81E-01
CDIPT	1	7.40E-01	2	8.86E-01	2	4.55E-01	3	4.20E-01
IMPA1	1	1.26E-02	7	4.81E-01	7	5.48E-01	3	7.48E-02
IMPA2	25	1.65E-01	23	6.69E-02	23	2.51E-01	26	7.40E-01
IMPAD1	6	1.86E-01	6	1.49E-01	6	1.08E-01	6	9.87E-01
INPP1	9	1.58E-01	12	3.48E-01	12	3.82E-01	11	7.75E-01
INPP4A	10	8.41E-01	8	9.52E-01	8	8.74E-01	10	2.99E-01
INPP4B	114	6.69E-02	109	1.89E-02	108	5.10E-01	122	6.14E-01
INPP5A	30	3.26E-01	28	8.60E-03	28	4.65E-02	33	3.36E-01
INPP5B	2	2.38E-01	1	3.17E-01	1	5.85E-01	2	4.84E-01
INPP5D	50	7.70E-03	43	8.31E-01	43	3.83E-01	50	1.06E-01
INPP5E	2	8.45E-02	4	4.88E-01	4	5.63E-01	4	1.52E-01
INPP5J	19	3.85E-02	15	4.39E-01	15	6.67E-01	20	5.98E-01
INPP5K	10	4.10E-02	9	2.27E-01	9	6.50E-01	10	7.98E-01
INPPL1	3	7.97E-02	5	5.89E-01	5	3.93E-02	5	1.83E-01
IP6K1	5	3.42E-01	4	2.96E-01	4	3.30E-01	5	9.50E-04
IP6K2	6	8.35E-01	2	6.37E-01	2	5.95E-01	6	4.70E-03
IP6K3	26	9.49E-02	18	4.63E-01	18	9.19E-01	26	2.31E-02
IPMK	5	5.53E-01	9	4.58E-01	9	6.89E-01	7	4.49E-01
IPPK	4	3.20E-01	6	6.67E-01	6	7.23E-02	8	2.81E-01
ISYNA1	2	5.22E-01	1	8.59E-01	1	3.34E-01	1	2.52E-01
ITPK1	39	1.89E-01	38	5.91E-01	38	2.69E-01	41	2.58E-01
ITPKA	1	8.37E-01	2	2.65E-03	2	7.27E-02	3	3.04E-01
ITPKB	23	5.11E-01	25	7.39E-01	25	1.56E-02	25	2.40E-01
ITPKC	8	3.95E-03	7	1.46E-01	7	1.28E-02	8	8.63E-01
MINPP1	9	1.44E-01	13	2.51E-01	13	4.32E-02	11	7.28E-01
MIOX	2	2.38E-01	2	5.02E-01	2	6.79E-01	4	1.04E-01
NUDT3	6	7.37E-01	4	1.44E-01	4	9.63E-02	6	9.64E-01
NUDT4	11	8.71E-02	8	6.91E-01	8	3.96E-01	11	9.00E-01
PI4K2A	8	5.72E-01	8	9.93E-01	8	8.09E-01	8	2.63E-01
PI4K2B	7	4.31E-02	8	9.35E-01	8	7.96E-01	7	7.15E-01
PI4KA	10	9.35E-03	13	1.29E-01	11	4.71E-01	14	7.47E-01
PI4KB	7	4.30E-02	7	8.86E-01	7	7.44E-01	25	2.40E-01
PIK3C2A	4	9.00E-01	3	2.43E-01	4	8.52E-01	5	8.31E-01
PIK3C2B	18	1.46E-01	20	2.22E-01	20	9.29E-01	18	2.86E-01
PIK3C2G	77	7.02E-02	71	1.02E-01	72	2.85E-01	85	4.66E-01

PIK3C3	8	3.23E-02	7	9.93E-01	7	8.35E-01	8	1.21E-01
PIK3CA	11	1.42E-01	10	7.46E-01	10	5.98E-01	11	5.90E-01
PIK3CB	8	2.27E-02	1	3.64E-01	1	8.16E-01	9	9.81E-01
PIK3CD	8	3.06E-02	9	1.29E-01	9	7.02E-01	8	9.33E-01
PIK3CG	14	6.51E-01	13	6.37E-01	14	6.38E-01	16	5.05E-01
PIKFYVE	13	4.25E-02	19	5.62E-01	21	5.31E-01	13	2.72E-01
PIP4K2A	49	5.40E-01	40	9.70E-01	37	6.04E-02	51	7.03E-01
PIP4K2B	8	6.31E-01	8	6.03E-01	8	9.84E-01	10	8.84E-01
PIP4K2C	4	3.95E-02	4	2.92E-01	4	9.46E-01	4	8.23E-01
PIP5K1A	3	1.84E-01	4	6.87E-01	4	9.25E-01	4	8.15E-01
PIP5K1B	62	1.02E-01	67	6.04E-01	67	4.02E-01	68	5.81E-01
PIP5K1C	8	1.42E-02	9	9.43E-01	9	5.98E-01	9	1.11E-01
PIP5KL1	3	8.46E-01	5	6.09E-01	5	2.80E-01	4	6.64E-02
PLCB1	245	3.40E-01	213	3.85E-01	213	3.63E-01	248	8.50E-04
PLCB2	5	1.52E-01	7	4.46E-01	7	2.98E-01	7	5.07E-01
PLCB3	6	4.54E-01	7	3.35E-01	7	1.49E-01	7	3.90E-01
PLCB4	72	5.84E-01	73	4.64E-01	73	4.74E-01	76	3.88E-01
PLCD1	5	2.98E-01	5	4.37E-01	5	6.61E-01	7	4.47E-01
PLCD3	14	4.34E-01	16	3.83E-01	16	2.39E-01	16	2.72E-01
PLCD4	1	8.84E-01	4	8.07E-01	4	2.61E-01	3	4.74E-01
PLCE1	57	3.56E-02	60	5.00E-05	60	5.00E-05	61	4.61E-01
PLCG1	4	1.55E-02	5	1.99E-01	5	1.10E-01	7	2.20E-02
PLCG2	99	7.10E-01	76	8.89E-02	75	6.27E-02	103	1.90E-01
PLCH1	16	5.60E-02	10	3.93E-01	10	7.88E-01	15	9.26E-01
PLCH2	10	1.15E-01	7	4.29E-01	7	7.33E-02	10	8.02E-01
PLCZ1	20	2.37E-01	17	4.75E-01	17	9.89E-01	22	6.05E-01
PLD4	5	5.75E-01	4	7.67E-01	4	8.24E-01	4	3.36E-01
PPIP5K1	9	6.66E-02	7	6.53E-01	7	4.00E-01	9	4.59E-01
PPIP5K2	9	7.80E-01	7	7.10E-01	7	3.16E-01	9	2.64E-01
PTEN	9	7.15E-01	9	7.17E-01	9	8.10E-01	11	4.27E-01
SYNJ1	10	6.92E-02	9	6.06E-01	10	6.35E-01	13	1.65E-01
SYNJ2	40	8.14E-01	36	6.65E-03	36	8.95E-01	40	4.74E-02
TPI1	2	6.08E-01	1	1.48E-01	1	7.69E-01	2	5.25E-01

	**Pancreatic cancer**		**Breast cancer**		**Prostate cancer**		**Bladder cancer**	
**Gene** [a]	**N.SNP**	**P value**	**N.SNP**	**P value**	**N.SNP**	**P value**	**N.SNP**	**P value**
ALDH6A1	5	8.16E-01	5	4.35E-01	5	3.48E-01	1	2.99E-01
CALM1	8	5.13E-01	8	9.47E-01	8	3.54E-01	2	9.50E-01
CALM2	10	8.66E-01	8	4.76E-01	8	2.87E-01	4	1.53E-01
CALM3	6	2.91E-02	5	1.69E-01	5	4.63E-01	2	8.12E-01
CDIPT	3	7.60E-01	3	2.48E-01	3	5.67E-01	2	1.89E-01
IMPA1	2	9.45E-01	3	2.10E-01	3	9.39E-01	-	-
IMPA2	25	4.13E-02	26	4.12E-02	26	1.93E-01	10	1.22E-01
IMPAD1	6	4.84E-01	5	1.11E-01	7	4.06E-02	2	3.84E-01
INPP1	11	4.84E-01	11	5.75E-01	11	2.03E-01	5	1.85E-01

INPP4A	10	1.92E-01	10	4.64E-01	10	9.69E-01	4	8.09E-01
INPP4B	120	2.91E-01	122	5.64E-02	119	7.45E-02	49	5.13E-01
INPP5A	33	9.64E-01	33	5.84E-01	33	6.76E-01	12	9.31E-01
INPP5B	2	1.14E-01	1	8.47E-01	2	1.77E-01	2	4.90E-01
INPP5D	51	6.58E-01	51	8.56E-01	51	8.54E-01	24	4.09E-02
INPP5E	4	4.54E-01	4	9.76E-01	4	3.33E-01	2	2.96E-01
INPP5J	20	5.14E-01	19	2.64E-01	19	4.82E-01	3	8.57E-01
INPP5K	8	7.17E-01	11	4.90E-02	10	7.86E-01	1	2.25E-02
INPPL1	5	2.76E-01	4	3.76E-01	4	1.24E-01	1	9.62E-01
IP6K1	5	6.98E-01	5	1.66E-01	5	5.44E-01	2	6.50E-01
IP6K2	6	2.66E-01	6	9.44E-01	6	4.08E-01	2	9.11E-01
IP6K3	26	5.83E-01	26	3.11E-01	26	6.53E-01	9	3.24E-01
IPMK	7	2.95E-01	6	6.15E-01	6	2.42E-01	2	2.57E-01
IPPK	8	8.64E-01	8	1.35E-01	8	4.69E-01	4	9.83E-02
ISYNA1	1	9.57E-01	1	4.82E-01	2	4.54E-01	2	1.12E-01
ITPK1	41	1.15E-01	41	1.85E-01	42	8.17E-01	14	8.76E-01
ITPKA	3	9.37E-01	3	8.50E-01	3	7.60E-01	2	2.05E-01
ITPKB	26	4.62E-02	26	9.67E-01	26	7.38E-01	8	2.60E-01
ITPKC	8	4.53E-01	7	3.18E-01	7	1.94E-02	3	9.03E-01
MINPP1	10	1.88E-01	10	8.78E-01	11	4.32E-01	-	-
MIOX	4	7.28E-01	4	7.67E-01	4	6.54E-01	2	7.48E-01
NUDT3	6	4.30E-02	249	9.29E-01	6	7.23E-01	1	7.16E-01
NUDT4	11	5.63E-02	11	1.05E-01	11	4.88E-01	5	1.92E-01
PI4K2A	8	2.43E-01	8	2.24E-01	8	1.45E-01	3	7.41E-01
PI4K2B	7	3.83E-01	7	4.86E-01	7	4.80E-01	1	1.82E-01
PI4KA	14	8.80E-02	13	5.26E-01	14	6.34E-02	6	3.50E-01
PI4KB	7	1.95E-01	7	6.10E-01	7	6.53E-01	3	5.73E-01
PIK3C2A	5	2.29E-01	6	2.76E-01	5	1.06E-01	11	1.72E-01
PIK3C2B	17	3.28E-01	17	1.52E-02	18	6.35E-01	11	1.72E-01
PIK3C2G	86	4.34E-02	86	7.46E-01	87	1.67E-01	36	4.84E-01
PIK3C3	8	9.14E-02	8	9.76E-02	8	9.91E-01	3	2.07E-01
PIK3CA	11	9.74E-02	11	8.36E-01	12	2.39E-01	1	5.13E-01
PIK3CB	9	3.39E-01	9	1.99E-01	9	9.43E-02	1	6.73E-01
PIK3CD	7	6.22E-01	8	8.47E-01	8	8.63E-01	2	1.02E-01
PIK3CG	17	3.36E-01	17	7.93E-01	17	6.34E-01	6	3.17E-01
PIKFYVE	14	9.47E-01	14	2.81E-01	14	5.37E-01	7	9.85E-01
PIP4K2A	51	5.42E-01	51	1.24E-01	52	7.12E-01	25	9.89E-01
PIP4K2B	10	9.62E-01	11	6.24E-01	11	5.41E-01	-	-
PIP4K2C	4	3.98E-01	4	8.39E-01	4	8.56E-01	2	4.88E-01
PIP5K1A	4	9.89E-01	3	9.13E-01	3	7.61E-01	1	7.70E-01
PIP5K1B	68	3.75E-01	67	5.11E-01	63	3.66E-01	13	1.52E-01
PIP5K1C	10	6.81E-01	8	6.91E-01	10	5.26E-01	2	6.87E-01
PIP5KL1	4	9.28E-01	3	4.11E-01	3	8.01E-01	1	3.28E-01
PLCB1	251	2.86E-01	245	2.20E-01	250	2.72E-01	106	9.51E-01

PLCB2	7	2.00E-01	10	2.32E-01	9	3.63E-01	2	4.76E-01
PLCB3	7	5.19E-01	7	8.94E-01	7	1.14E-01	2	6.64E-01
PLCB4	75	6.30E-01	77	5.34E-01	77	9.40E-01	41	9.34E-01
PLCD1	8	1.25E-03	17	2.96E-02	7	1.23E-02	2	6.19E-01
PLCD3	17	9.42E-01	16	5.30E-01	17	6.88E-01	8	1.52E-01
PLCD4	3	6.57E-01	3	6.69E-01	3	7.21E-02	2	4.46E-01
PLCE1	63	2.49E-01	61	5.13E-01	60	2.55E-01	24	4.06E-01
PLCG1	7	9.38E-01	6	6.75E-01	6	1.42E-01	1	5.13E-02
PLCG2	103	4.16E-01	103	2.82E-01	102	6.99E-01	62	5.92E-01
PLCH1	14	5.03E-01	15	4.07E-01	17	2.52E-01	4	9.77E-01
PLCH2	10	2.84E-02	11	2.51E-02	11	1.02E-01	2	6.01E-01
PLCZ1	23	1.80E-01	24	2.75E-01	23	1.87E-01	6	9.42E-01
PLD4	5	8.39E-01	5	7.76E-01	5	6.78E-01	2	5.25E-01
PPIP5K1	9	5.04E-01	9	7.33E-01	9	7.06E-01	6	8.59E-02
PPIP5K2	9	6.61E-01	9	7.93E-01	9	6.09E-01	5	5.76E-01
PTEN	11	6.22E-01	11	5.23E-02	11	1.78E-01	3	2.27E-01
SYNJ1	13	1.57E-01	14	9.96E-01	14	1.97E-01	8	6.92E-01
SYNJ2	41	6.03E-02	44	2.79E-02	44	2.53E-01	16	4.96E-01
TPI1	2	9.61E-01	2	5.46E-01	2	8.02E-01	1	1.58E-01

[a] Genes were listed in alphabetical order.

* For lung cancer, there were four studies in the original GWAS data, incluing the Environment and Genetics in Lung Cancer Etiology (EAGLE); the Prostate, Lung, Colon, Ovary Screening Trial (PLCO); the Alpha-Tocopherol, Beta-Carotene Cancer Prevention Study (ATBC); the Cancer Prevention Study II Nutrition Cohort (CPS-II). We just downloaded the CADM concent group without the study EAGLE. The original article of lung cancer GWAS selected two PCs for PLCO , one for each EAGLE and ATBC, and no PCs were selected for the CPS-II study. We choosed the same number of PCs for each study in lung cancer. Therefore, we adjusted 3 PCs for lung cancer.
N.SNP: number of SNPs.

Supplementary Table SIII. The SNPs and located genes included in the study for cancers.

**lung cancer**		**ESCC**		**GC**		**RCC**	
SNP	*Gene*	SNP	*Gene*	SNP	*Gene*	SNP	*Gene*
rs2072294	*ALDH6A1*	rs17096208	*ALDH6A1*	rs17096208	*ALDH6A1*	rs3742809	*ALDH6A1*
rs3742809	*ALDH6A1*	rs1984511	*ALDH6A1*	rs1984511	*ALDH6A1*	rs765719	*ALDH6A1*
rs765719	*ALDH6A1*	rs2006731	*ALDH6A1*	rs2006731	*ALDH6A1*	rs17096208	*ALDH6A1*
rs1058903	*CALM1*	rs2006732	*ALDH6A1*	rs2006732	*ALDH6A1*	rs2300193	*ALDH6A1*
rs2300497	*CALM1*	rs2072293	*ALDH6A1*	rs2072293	*ALDH6A1*	rs6575129	*CALM1*
rs2300502	*CALM1*	rs2072294	*ALDH6A1*	rs2072294	*ALDH6A1*	rs7144433	*CALM1*
rs3213718	*CALM1*	rs2239556	*ALDH6A1*	rs2239556	*ALDH6A1*	rs2300497	*CALM1*

rs5871	*CALM1*	rs2239557	*ALDH6A1*	rs2239557	*ALDH6A1*	rs2300502	*CALM1*
rs6575129	*CALM1*	rs2300193	*ALDH6A1*	rs2300193	*ALDH6A1*	rs3213718	*CALM1*
rs7144433	*CALM1*	rs2878782	*ALDH6A1*	rs2878782	*ALDH6A1*	rs5871	*CALM1*
rs8006462	*CALM1*	rs3742805	*ALDH6A1*	rs3742805	*ALDH6A1*	rs1058903	*CALM1*
rs10865222	*CALM2*	rs3742809	*ALDH6A1*	rs3742809	*ALDH6A1*	rs8006462	*CALM1*
rs13410472	*CALM2*	rs3815330	*ALDH6A1*	rs3815330	*ALDH6A1*	rs8006462	*CALM1*
rs17036320	*CALM2*	rs4232872	*ALDH6A1*	rs4232872	*ALDH6A1*	rs4953470	*CALM2*
rs4953470	*CALM2*	rs4243645	*ALDH6A1*	rs4243645	*ALDH6A1*	rs815808	*CALM2*
rs7581908	*CALM2*	rs4646855	*ALDH6A1*	rs4646855	*ALDH6A1*	rs815804	*CALM2*
rs815802	*CALM2*	rs4646857	*ALDH6A1*	rs4646857	*ALDH6A1*	rs10865222	*CALM2*
rs815804	*CALM2*	rs4646858	*ALDH6A1*	rs4646858	*ALDH6A1*	rs17036320	*CALM2*
rs815808	*CALM2*	rs4646859	*ALDH6A1*	rs4646859	*ALDH6A1*	rs815802	*CALM2*
rs10113	*CALM3*	rs4646861	*ALDH6A1*	rs4646861	*ALDH6A1*	rs815815	*CALM2*
rs11083841	*CALM3*	rs4646864	*ALDH6A1*	rs4646864	*ALDH6A1*	rs13410472	*CALM2*
rs1126510	*CALM3*	rs765719	*ALDH6A1*	rs765719	*ALDH6A1*	rs7581908	*CALM2*
rs7258489	*CALM3*	rs8204	*ALDH6A1*	rs8204	*ALDH6A1*	rs9808216	*CALM2*
rs7259810	*CALM3*	rs1058903	*CALM1*	rs1058903	*CALM1*	rs4274528	*CALM3*
rs3815822	*CDIPT*	rs2300497	*CALM1*	rs2300497	*CALM1*	rs7258489	*CALM3*
rs1967328	*IMPA1*	rs2300502	*CALM1*	rs2300502	*CALM1*	rs7259810	*CALM3*
rs1020294	*IMPA2*	rs3213718	*CALM1*	rs3213718	*CALM1*	rs10113	*CALM3*
rs1250171	*IMPA2*	rs5871	*CALM1*	rs5871	*CALM1*	rs11083841	*CALM3*
rs1262056	*IMPA2*	rs7144433	*CALM1*	rs7144433	*CALM1*	rs1126510	*CALM3*
rs16976931	*IMPA2*	rs8006462	*CALM1*	rs8006462	*CALM1*	rs1126510	*CALM3*
rs16976956	*IMPA2*	rs10865222	*CALM2*	rs10865222	*CALM2*	rs3815822	*CDIPT*
rs17593321	*IMPA2*	rs17036320	*CALM2*	rs17036320	*CALM2*	rs12917712	*CDIPT*
rs1787984	*IMPA2*	rs7581908	*CALM2*	rs7581908	*CALM2*	rs4787483	*CDIPT*
rs2360082	*IMPA2*	rs815802	*CALM2*	rs815802	*CALM2*	rs4787483	*CDIPT*
rs3786284	*IMPA2*	rs815804	*CALM2*	rs815804	*CALM2*	rs1967328	*IMPA1*
rs3786305	*IMPA2*	rs815808	*CALM2*	rs815808	*CALM2*	rs2912821	*IMPA1*
rs613993	*IMPA2*	rs815815	*CALM2*	rs815815	*CALM2*	rs2955005	*IMPA1*
rs628419	*IMPA2*	rs10113	*CALM3*	rs10113	*CALM3*	rs2955005	*IMPA1*
rs630110	*IMPA2*	rs11083841	*CALM3*	rs11083841	*CALM3*	rs679246	*IMPA2*
rs636173	*IMPA2*	rs1126510	*CALM3*	rs1126510	*CALM3*	rs1787984	*IMPA2*
rs638063	*IMPA2*	rs7258489	*CALM3*	rs7258489	*CALM3*	rs662383	*IMPA2*
rs647077	*IMPA2*	rs7259810	*CALM3*	rs7259810	*CALM3*	rs3786305	*IMPA2*
rs6505700	*IMPA2*	rs12917712	*CDIPT*	rs12917712	*CDIPT*	rs647077	*IMPA2*
rs662383	*IMPA2*	rs3815822	*CDIPT*	rs3815822	*CDIPT*	rs2360082	*IMPA2*
rs663591	*IMPA2*	rs16909509	*IMPA1*	rs16909509	*IMPA1*	rs7506045	*IMPA2*
rs679246	*IMPA2*	rs1967328	*IMPA1*	rs1967328	*IMPA1*	rs6505700	*IMPA2*
rs684680	*IMPA2*	rs2300494	*IMPA1*	rs2300494	*IMPA1*	rs663591	*IMPA2*
rs7235476	*IMPA2*	rs2912821	*IMPA1*	rs2912821	*IMPA1*	rs638063	*IMPA2*
rs7244678	*IMPA2*	rs2955005	*IMPA1*	rs2955005	*IMPA1*	rs9973072	*IMPA2*
rs7506045	*IMPA2*	rs2955006	*IMPA1*	rs2955006	*IMPA1*	rs684680	*IMPA2*
rs9973072	*IMPA2*	rs6473293	*IMPA1*	rs6473293	*IMPA1*	rs636173	*IMPA2*

rs1044731	*IMPAD1*	rs1020294	*IMPA2*	rs1020294	*IMPA2*	rs7244678	*IMPA2*
rs13257046	*IMPAD1*	rs1250171	*IMPA2*	rs1250171	*IMPA2*	rs16976931	*IMPA2*
rs4738558	*IMPAD1*	rs1262056	*IMPA2*	rs1262056	*IMPA2*	rs17593321	*IMPA2*
rs6474091	*IMPAD1*	rs16976931	*IMPA2*	rs16976931	*IMPA2*	rs3786284	*IMPA2*
rs6997875	*IMPAD1*	rs16976956	*IMPA2*	rs16976956	*IMPA2*	rs7235476	*IMPA2*
rs8718	*IMPAD1*	rs1787984	*IMPA2*	rs1787984	*IMPA2*	rs16976956	*IMPA2*
rs10931450	*INPP1*	rs2002212	*IMPA2*	rs2002212	*IMPA2*	rs628419	*IMPA2*
rs1108939	*INPP1*	rs2360082	*IMPA2*	rs2360082	*IMPA2*	rs1020294	*IMPA2*
rs2067416	*INPP1*	rs3786284	*IMPA2*	rs3786284	*IMPA2*	rs1262056	*IMPA2*
rs2067417	*INPP1*	rs3786305	*IMPA2*	rs3786305	*IMPA2*	rs1250171	*IMPA2*
rs2067418	*INPP1*	rs613993	*IMPA2*	rs613993	*IMPA2*	rs613993	*IMPA2*
rs2067434	*INPP1*	rs628419	*IMPA2*	rs628419	*IMPA2*	rs630110	*IMPA2*
rs291429	*INPP1*	rs636173	*IMPA2*	rs636173	*IMPA2*	rs9955952	*IMPA2*
rs3791809	*INPP1*	rs638063	*IMPA2*	rs638063	*IMPA2*	rs9955952	*IMPA2*
rs909270	*INPP1*	rs647077	*IMPA2*	rs647077	*IMPA2*	rs6474091	*IMPAD1*
rs11676357	*INPP4A*	rs6505700	*IMPA2*	rs6505700	*IMPA2*	rs8718	*IMPAD1*
rs17504837	*INPP4A*	rs662383	*IMPA2*	rs662383	*IMPA2*	rs1044731	*IMPAD1*
rs222	*INPP4A*	rs663591	*IMPA2*	rs663591	*IMPA2*	rs4738558	*IMPAD1*
rs2278206	*INPP4A*	rs684680	*IMPA2*	rs684680	*IMPA2*	rs13257046	*IMPAD1*
rs2278210	*INPP4A*	rs7235476	*IMPA2*	rs7235476	*IMPA2*	rs6997875	*IMPAD1*
rs2278214	*INPP4A*	rs7244678	*IMPA2*	rs7244678	*IMPA2*	rs291429	*INPP1*
rs3754876	*INPP4A*	rs7506045	*IMPA2*	rs7506045	*IMPA2*	rs3791809	*INPP1*
rs3754886	*INPP4A*	rs9955952	*IMPA2*	rs9955952	*IMPA2*	rs2067416	*INPP1*
rs3820947	*INPP4A*	rs9973072	*IMPA2*	rs9973072	*IMPA2*	rs2067417	*INPP1*
rs6734569	*INPP4A*	rs1044731	*IMPAD1*	rs1044731	*IMPAD1*	rs2067418	*INPP1*
rs10000770	*INPP4B*	rs13257046	*IMPAD1*	rs13257046	*IMPAD1*	rs2067434	*INPP1*
rs10013734	*INPP4B*	rs4738558	*IMPAD1*	rs4738558	*IMPAD1*	rs10931450	*INPP1*
rs10020322	*INPP4B*	rs6474091	*IMPAD1*	rs6474091	*IMPAD1*	rs909270	*INPP1*
rs1017527	*INPP4B*	rs6997875	*IMPAD1*	rs6997875	*IMPAD1*	rs1108939	*INPP1*
rs10519631	*INPP4B*	rs8718	*IMPAD1*	rs8718	*IMPAD1*	rs2736619	*INPP1*
rs10519638	*INPP4B*	rs10931450	*INPP1*	rs10931450	*INPP1*	rs1866852	*INPP1*
rs10519649	*INPP4B*	rs1108939	*INPP1*	rs1108939	*INPP1*	rs1866852	*INPP1*
rs10857395	*INPP4B*	rs1866852	*INPP1*	rs1866852	*INPP1*	rs3820947	*INPP4A*
rs11736202	*INPP4B*	rs2067416	*INPP1*	rs2067416	*INPP1*	rs3754886	*INPP4A*
rs11930321	*INPP4B*	rs2067417	*INPP1*	rs2067417	*INPP1*	rs11676357	*INPP4A*
rs11943687	*INPP4B*	rs2067418	*INPP1*	rs2067418	*INPP1*	rs2278214	*INPP4A*
rs1219266	*INPP4B*	rs2067434	*INPP1*	rs2067434	*INPP1*	rs17504837	*INPP4A*
rs1219274	*INPP4B*	rs2736619	*INPP1*	rs2736619	*INPP1*	rs222	*INPP4A*
rs1219275	*INPP4B*	rs291429	*INPP1*	rs291429	*INPP1*	rs2278210	*INPP4A*
rs12498546	*INPP4B*	rs291471	*INPP1*	rs291471	*INPP1*	rs6734569	*INPP4A*
rs12504681	*INPP4B*	rs3791809	*INPP1*	rs3791809	*INPP1*	rs2278206	*INPP4A*
rs13109869	*INPP4B*	rs909270	*INPP1*	rs909270	*INPP1*	rs3754876	*INPP4A*
rs13119090	*INPP4B*	rs11676357	*INPP4A*	rs11676357	*INPP4A*	rs3754876	*INPP4A*
rs13125853	*INPP4B*	rs222	*INPP4A*	rs222	*INPP4A*	rs17380914	*INPP4B*

rs13133181	*INPP4B*	rs2278206	*INPP4A*	rs2278206	*INPP4A*	rs11736202	*INPP4B*
rs13148456	*INPP4B*	rs2278210	*INPP4A*	rs2278210	*INPP4A*	rs336361	*INPP4B*
rs1353624	*INPP4B*	rs2278214	*INPP4A*	rs2278214	*INPP4A*	rs3775601	*INPP4B*
rs1364920	*INPP4B*	rs3754876	*INPP4A*	rs3754876	*INPP4A*	rs3775605	*INPP4B*
rs1373036	*INPP4B*	rs3754886	*INPP4A*	rs3754886	*INPP4A*	rs2667096	*INPP4B*
rs1390994	*INPP4B*	rs3820947	*INPP4A*	rs3820947	*INPP4A*	rs1511252	*INPP4B*
rs1391099	*INPP4B*	rs10013734	*INPP4B*	rs10013734	*INPP4B*	rs2667100	*INPP4B*
rs1425520	*INPP4B*	rs1017527	*INPP4B*	rs1017527	*INPP4B*	rs3775616	*INPP4B*
rs1425522	*INPP4B*	rs10519631	*INPP4B*	rs10519631	*INPP4B*	rs4956433	*INPP4B*
rs1425531	*INPP4B*	rs10519649	*INPP4B*	rs10519649	*INPP4B*	rs6537101	*INPP4B*
rs1425533	*INPP4B*	rs10857395	*INPP4B*	rs10857395	*INPP4B*	rs3822135	*INPP4B*
rs1443187	*INPP4B*	rs11736202	*INPP4B*	rs11736202	*INPP4B*	rs336298	*INPP4B*
rs1476122	*INPP4B*	rs11930321	*INPP4B*	rs11930321	*INPP4B*	rs17468315	*INPP4B*
rs1489577	*INPP4B*	rs11942593	*INPP4B*	rs11942593	*INPP4B*	rs336308	*INPP4B*
rs1489578	*INPP4B*	rs11943397	*INPP4B*	rs11943397	*INPP4B*	rs336332	*INPP4B*
rs1497391	*INPP4B*	rs11943687	*INPP4B*	rs11943687	*INPP4B*	rs6842783	*INPP4B*
rs1497393	*INPP4B*	rs1219266	*INPP4B*	rs1219266	*INPP4B*	rs12498546	*INPP4B*
rs1497400	*INPP4B*	rs1219274	*INPP4B*	rs1219274	*INPP4B*	rs978752	*INPP4B*
rs1511252	*INPP4B*	rs1219275	*INPP4B*	rs1219275	*INPP4B*	rs3775641	*INPP4B*
rs17015594	*INPP4B*	rs12498546	*INPP4B*	rs12498546	*INPP4B*	rs336407	*INPP4B*
rs17015754	*INPP4B*	rs12644329	*INPP4B*	rs12644329	*INPP4B*	rs336408	*INPP4B*
rs17015882	*INPP4B*	rs13109869	*INPP4B*	rs13109869	*INPP4B*	rs13148456	*INPP4B*
rs17015920	*INPP4B*	rs13119090	*INPP4B*	rs13119090	*INPP4B*	rs10519631	*INPP4B*
rs17016027	*INPP4B*	rs13125853	*INPP4B*	rs13125853	*INPP4B*	rs3775664	*INPP4B*
rs17380914	*INPP4B*	rs13133181	*INPP4B*	rs13133181	*INPP4B*	rs17015594	*INPP4B*
rs17468315	*INPP4B*	rs13148456	*INPP4B*	rs13148456	*INPP4B*	rs336391	*INPP4B*
rs1872293	*INPP4B*	rs1353624	*INPP4B*	rs1353624	*INPP4B*	rs336394	*INPP4B*
rs1872297	*INPP4B*	rs1364920	*INPP4B*	rs1364920	*INPP4B*	rs1219275	*INPP4B*
rs1907107	*INPP4B*	rs1373036	*INPP4B*	rs1373036	*INPP4B*	rs336384	*INPP4B*
rs1907108	*INPP4B*	rs1390994	*INPP4B*	rs1390994	*INPP4B*	rs1219266	*INPP4B*
rs1907134	*INPP4B*	rs1391099	*INPP4B*	rs1391099	*INPP4B*	rs10013734	*INPP4B*
rs1982965	*INPP4B*	rs1425520	*INPP4B*	rs1425520	*INPP4B*	rs1219274	*INPP4B*
rs1992418	*INPP4B*	rs1425522	*INPP4B*	rs1425522	*INPP4B*	rs2636683	*INPP4B*
rs2017146	*INPP4B*	rs1425531	*INPP4B*	rs1425531	*INPP4B*	rs1390994	*INPP4B*
rs2055212	*INPP4B*	rs1425533	*INPP4B*	rs1425533	*INPP4B*	rs2055212	*INPP4B*
rs2165819	*INPP4B*	rs1443187	*INPP4B*	rs1443187	*INPP4B*	rs3775671	*INPP4B*
rs2627804	*INPP4B*	rs1476122	*INPP4B*	rs1476122	*INPP4B*	rs2627804	*INPP4B*
rs2627813	*INPP4B*	rs1489577	*INPP4B*	rs1489577	*INPP4B*	rs17015754	*INPP4B*
rs2635429	*INPP4B*	rs1489578	*INPP4B*	rs1489578	*INPP4B*	rs13109869	*INPP4B*
rs2636632	*INPP4B*	rs1497391	*INPP4B*	rs1497391	*INPP4B*	rs6846839	*INPP4B*
rs2636638	*INPP4B*	rs1497393	*INPP4B*	rs1497393	*INPP4B*	rs6847049	*INPP4B*
rs2636643	*INPP4B*	rs1497400	*INPP4B*	rs1497400	*INPP4B*	rs3113511	*INPP4B*
rs2636660	*INPP4B*	rs1511252	*INPP4B*	rs1511252	*INPP4B*	rs2636660	*INPP4B*
rs2636670	*INPP4B*	rs17015594	*INPP4B*	rs17015594	*INPP4B*	rs2636643	*INPP4B*

rs2636671	*INPP4B*	rs17015882	*INPP4B*	rs17015882	*INPP4B*	rs1907134	*INPP4B*
rs2636683	*INPP4B*	rs17016027	*INPP4B*	rs17016027	*INPP4B*	rs2627813	*INPP4B*
rs2667096	*INPP4B*	rs17468315	*INPP4B*	rs17468315	*INPP4B*	rs2636632	*INPP4B*
rs2667100	*INPP4B*	rs1872293	*INPP4B*	rs1872293	*INPP4B*	rs1872297	*INPP4B*
rs2874870	*INPP4B*	rs1872297	*INPP4B*	rs1872297	*INPP4B*	rs3822152	*INPP4B*
rs3113511	*INPP4B*	rs1907107	*INPP4B*	rs1907107	*INPP4B*	rs10519638	*INPP4B*
rs331941	*INPP4B*	rs1907108	*INPP4B*	rs1907108	*INPP4B*	rs3775692	*INPP4B*
rs331946	*INPP4B*	rs1907134	*INPP4B*	rs1907134	*INPP4B*	rs17015882	*INPP4B*
rs336298	*INPP4B*	rs1982965	*INPP4B*	rs1982965	*INPP4B*	rs1907108	*INPP4B*
rs336308	*INPP4B*	rs1992418	*INPP4B*	rs1992418	*INPP4B*	rs3775696	*INPP4B*
rs336332	*INPP4B*	rs2017146	*INPP4B*	rs2017146	*INPP4B*	rs1907107	*INPP4B*
rs336361	*INPP4B*	rs2055212	*INPP4B*	rs2055212	*INPP4B*	rs2636638	*INPP4B*
rs336384	*INPP4B*	rs2165819	*INPP4B*	rs2165819	*INPP4B*	rs1872293	*INPP4B*
rs336391	*INPP4B*	rs2200997	*INPP4B*	rs2200997	*INPP4B*	rs2636670	*INPP4B*
rs336394	*INPP4B*	rs2627804	*INPP4B*	rs2627804	*INPP4B*	rs2636671	*INPP4B*
rs336407	*INPP4B*	rs2627808	*INPP4B*	rs2627808	*INPP4B*	rs3756121	*INPP4B*
rs336408	*INPP4B*	rs2627813	*INPP4B*	rs2627813	*INPP4B*	rs960678	*INPP4B*
rs3756121	*INPP4B*	rs2635429	*INPP4B*	rs2635429	*INPP4B*	rs17015920	*INPP4B*
rs3756125	*INPP4B*	rs2636632	*INPP4B*	rs2636632	*INPP4B*	rs2635429	*INPP4B*
rs3775601	*INPP4B*	rs2636638	*INPP4B*	rs2636638	*INPP4B*	rs716762	*INPP4B*
rs3775605	*INPP4B*	rs2636643	*INPP4B*	rs2636643	*INPP4B*	rs1497393	*INPP4B*
rs3775616	*INPP4B*	rs2636670	*INPP4B*	rs2636670	*INPP4B*	rs1353624	*INPP4B*
rs3775641	*INPP4B*	rs2636671	*INPP4B*	rs2636671	*INPP4B*	rs17016027	*INPP4B*
rs3775664	*INPP4B*	rs2636683	*INPP4B*	rs2636683	*INPP4B*	rs11930321	*INPP4B*
rs3775671	*INPP4B*	rs2667096	*INPP4B*	rs2667096	*INPP4B*	rs1982965	*INPP4B*
rs3775692	*INPP4B*	rs2667100	*INPP4B*	rs2667100	*INPP4B*	rs1497400	*INPP4B*
rs3775696	*INPP4B*	rs2874870	*INPP4B*	rs2874870	*INPP4B*	rs1489578	*INPP4B*
rs3775707	*INPP4B*	rs3113511	*INPP4B*	rs3113511	*INPP4B*	rs1489577	*INPP4B*
rs3775720	*INPP4B*	rs331946	*INPP4B*	rs331946	*INPP4B*	rs3775707	*INPP4B*
rs3822135	*INPP4B*	rs336298	*INPP4B*	rs336298	*INPP4B*	rs6820463	*INPP4B*
rs3822152	*INPP4B*	rs336332	*INPP4B*	rs336332	*INPP4B*	rs3756125	*INPP4B*
rs4690699	*INPP4B*	rs336361	*INPP4B*	rs336361	*INPP4B*	rs1497391	*INPP4B*
rs4956433	*INPP4B*	rs336384	*INPP4B*	rs336384	*INPP4B*	rs3775720	*INPP4B*
rs6814775	*INPP4B*	rs336391	*INPP4B*	rs336391	*INPP4B*	rs1391099	*INPP4B*
rs6820463	*INPP4B*	rs336394	*INPP4B*	rs336394	*INPP4B*	rs7668133	*INPP4B*
rs6830886	*INPP4B*	rs336407	*INPP4B*	rs336407	*INPP4B*	rs6837163	*INPP4B*
rs6837163	*INPP4B*	rs336408	*INPP4B*	rs336408	*INPP4B*	rs11930849	*INPP4B*
rs6842783	*INPP4B*	rs3756121	*INPP4B*	rs3756125	*INPP4B*	rs713071	*INPP4B*
rs6846839	*INPP4B*	rs3756125	*INPP4B*	rs3775601	*INPP4B*	rs1425533	*INPP4B*
rs6847049	*INPP4B*	rs3775601	*INPP4B*	rs3775605	*INPP4B*	rs1425531	*INPP4B*
rs713071	*INPP4B*	rs3775605	*INPP4B*	rs3775616	*INPP4B*	rs1364920	*INPP4B*
rs716762	*INPP4B*	rs3775616	*INPP4B*	rs3775641	*INPP4B*	rs7691439	*INPP4B*
rs745719	*INPP4B*	rs3775641	*INPP4B*	rs3775669	*INPP4B*	rs975136	*INPP4B*
rs7668133	*INPP4B*	rs3775669	*INPP4B*	rs3775671	*INPP4B*	rs10857395	*INPP4B*

rs7685002	*INPP4B*	rs3775671	*INPP4B*	rs3775692	*INPP4B*	rs10519649	*INPP4B*
rs7688435	*INPP4B*	rs3775692	*INPP4B*	rs3775696	*INPP4B*	rs1476122	*INPP4B*
rs7691439	*INPP4B*	rs3775696	*INPP4B*	rs3775707	*INPP4B*	rs1425520	*INPP4B*
rs960678	*INPP4B*	rs3775707	*INPP4B*	rs3775720	*INPP4B*	rs6814775	*INPP4B*
rs966457	*INPP4B*	rs3775720	*INPP4B*	rs3822129	*INPP4B*	rs11943687	*INPP4B*
rs975136	*INPP4B*	rs3822129	*INPP4B*	rs3822135	*INPP4B*	rs1425522	*INPP4B*
rs978752	*INPP4B*	rs3822135	*INPP4B*	rs3822152	*INPP4B*	rs7688435	*INPP4B*
rs10747068	*INPP5A*	rs3822152	*INPP4B*	rs4690699	*INPP4B*	rs2874870	*INPP4B*
rs10781583	*INPP5A*	rs4690699	*INPP4B*	rs4690728	*INPP4B*	rs2017146	*INPP4B*
rs10781585	*INPP5A*	rs4690728	*INPP4B*	rs6537101	*INPP4B*	rs7685002	*INPP4B*
rs11146457	*INPP5A*	rs6537101	*INPP4B*	rs6537129	*INPP4B*	rs745719	*INPP4B*
rs11146487	*INPP5A*	rs6537129	*INPP4B*	rs6814775	*INPP4B*	rs13125853	*INPP4B*
rs11818345	*INPP5A*	rs6814775	*INPP4B*	rs6819498	*INPP4B*	rs13119090	*INPP4B*
rs12267364	*INPP5A*	rs6819498	*INPP4B*	rs6820463	*INPP4B*	rs1017527	*INPP4B*
rs12412313	*INPP5A*	rs6820463	*INPP4B*	rs6830886	*INPP4B*	rs10020322	*INPP4B*
rs2246288	*INPP5A*	rs6830886	*INPP4B*	rs6837163	*INPP4B*	rs13133181	*INPP4B*
rs2492768	*INPP5A*	rs6837163	*INPP4B*	rs6842783	*INPP4B*	rs6830886	*INPP4B*
rs2767419	*INPP5A*	rs6842783	*INPP4B*	rs713071	*INPP4B*	rs10000770	*INPP4B*
rs2786900	*INPP5A*	rs713071	*INPP4B*	rs716762	*INPP4B*	rs11943397	*INPP4B*
rs2803989	*INPP5A*	rs716762	*INPP4B*	rs745719	*INPP4B*	rs1373036	*INPP4B*
rs2803997	*INPP5A*	rs745719	*INPP4B*	rs7668133	*INPP4B*	rs11942593	*INPP4B*
rs3793669	*INPP5A*	rs7668133	*INPP4B*	rs7685002	*INPP4B*	rs10009093	*INPP4B*
rs3793670	*INPP5A*	rs7685002	*INPP4B*	rs7688435	*INPP4B*	rs1443187	*INPP4B*
rs3793673	*INPP5A*	rs7688435	*INPP4B*	rs960678	*INPP4B*	rs12644329	*INPP4B*
rs3793687	*INPP5A*	rs960678	*INPP4B*	rs966457	*INPP4B*	rs4690699	*INPP4B*
rs4272721	*INPP5A*	rs966457	*INPP4B*	rs978752	*INPP4B*	rs2165819	*INPP4B*
rs4394754	*INPP5A*	rs978752	*INPP4B*	rs10747068	*INPP5A*	rs331941	*INPP4B*
rs4880419	*INPP5A*	rs10747068	*INPP5A*	rs10781585	*INPP5A*	rs331946	*INPP4B*
rs7084105	*INPP5A*	rs10781585	*INPP5A*	rs11146457	*INPP5A*	rs966457	*INPP4B*
rs7091957	*INPP5A*	rs11146457	*INPP5A*	rs11146487	*INPP5A*	rs12504681	*INPP4B*
rs7903076	*INPP5A*	rs11146487	*INPP5A*	rs1133400	*INPP5A*	rs1992418	*INPP4B*
rs7907513	*INPP5A*	rs1133400	*INPP5A*	rs11818345	*INPP5A*	rs4690728	*INPP4B*
rs7912273	*INPP5A*	rs11818345	*INPP5A*	rs12267364	*INPP5A*	rs6537129	*INPP4B*
rs7914354	*INPP5A*	rs12267364	*INPP5A*	rs12412313	*INPP5A*	rs6537129	*INPP4B*
rs7914822	*INPP5A*	rs12412313	*INPP5A*	rs12775208	*INPP5A*	rs7914354	*INPP5A*
rs873946	*INPP5A*	rs12775208	*INPP5A*	rs2246288	*INPP5A*	rs10747068	*INPP5A*
rs913193	*INPP5A*	rs2246288	*INPP5A*	rs2492768	*INPP5A*	rs4272721	*INPP5A*
rs4653342	*INPP5B*	rs2492768	*INPP5A*	rs2767419	*INPP5A*	rs7903076	*INPP5A*
rs473279	*INPP5B*	rs2767419	*INPP5A*	rs2786900	*INPP5A*	rs4394754	*INPP5A*
rs10193128	*INPP5D*	rs2786900	*INPP5A*	rs2803989	*INPP5A*	rs11818345	*INPP5A*
rs10203185	*INPP5D*	rs2803989	*INPP5A*	rs3793669	*INPP5A*	rs7912273	*INPP5A*
rs10803668	*INPP5D*	rs3793669	*INPP5A*	rs3793670	*INPP5A*	rs2786900	*INPP5A*
rs10929316	*INPP5D*	rs3793670	*INPP5A*	rs3793673	*INPP5A*	rs2492768	*INPP5A*
rs10933435	*INPP5D*	rs3793673	*INPP5A*	rs3793687	*INPP5A*	rs7084105	*INPP5A*

rs11673739	*INPP5D*	rs3793687	*INPP5A*	rs4394754	*INPP5A*	rs7907513	*INPP5A*
rs11674483	*INPP5D*	rs4394754	*INPP5A*	rs4880419	*INPP5A*	rs7091957	*INPP5A*
rs11682728	*INPP5D*	rs4880419	*INPP5A*	rs7084105	*INPP5A*	rs4880419	*INPP5A*
rs11693862	*INPP5D*	rs7084105	*INPP5A*	rs7091957	*INPP5A*	rs12412313	*INPP5A*
rs12694922	*INPP5D*	rs7091957	*INPP5A*	rs7903076	*INPP5A*	rs1133400	*INPP5A*
rs12694923	*INPP5D*	rs7903076	*INPP5A*	rs7907513	*INPP5A*	rs10781583	*INPP5A*
rs13021302	*INPP5D*	rs7907513	*INPP5A*	rs7912273	*INPP5A*	rs7914822	*INPP5A*
rs13031194	*INPP5D*	rs7912273	*INPP5A*	rs7914354	*INPP5A*	rs11146457	*INPP5A*
rs13385922	*INPP5D*	rs7914354	*INPP5A*	rs873946	*INPP5A*	rs2767419	*INPP5A*
rs1400349	*INPP5D*	rs873946	*INPP5A*	rs913193	*INPP5A*	rs2246288	*INPP5A*
rs14243	*INPP5D*	rs913193	*INPP5A*	rs473279	*INPP5B*	rs2803997	*INPP5A*
rs3792117	*INPP5D*	rs473279	*INPP5B*	rs10193128	*INPP5D*	rs873946	*INPP5A*
rs3890760	*INPP5D*	rs10193128	*INPP5D*	rs10203185	*INPP5D*	rs3793669	*INPP5A*
rs4073363	*INPP5D*	rs10203185	*INPP5D*	rs10803668	*INPP5D*	rs3793670	*INPP5A*
rs4246649	*INPP5D*	rs10803668	*INPP5D*	rs10933435	*INPP5D*	rs913193	*INPP5A*
rs4257390	*INPP5D*	rs10933435	*INPP5D*	rs11673739	*INPP5D*	rs3793673	*INPP5A*
rs4315512	*INPP5D*	rs11673739	*INPP5D*	rs11674347	*INPP5D*	rs11146487	*INPP5A*
rs4335931	*INPP5D*	rs11674347	*INPP5D*	rs11674483	*INPP5D*	rs10781585	*INPP5A*
rs4356648	*INPP5D*	rs11674483	*INPP5D*	rs11682728	*INPP5D*	rs12267364	*INPP5A*
rs4436949	*INPP5D*	rs11682728	*INPP5D*	rs11693862	*INPP5D*	rs2803989	*INPP5A*
rs4439944	*INPP5D*	rs11693862	*INPP5D*	rs12694922	*INPP5D*	rs3793687	*INPP5A*
rs4468807	*INPP5D*	rs12694922	*INPP5D*	rs12694923	*INPP5D*	rs12775208	*INPP5A*
rs4503982	*INPP5D*	rs12694923	*INPP5D*	rs13021302	*INPP5D*	rs2737398	*INPP5A*
rs4571051	*INPP5D*	rs13021302	*INPP5D*	rs13385922	*INPP5D*	rs2737398	*INPP5A*
rs4603757	*INPP5D*	rs13385922	*INPP5D*	rs13432536	*INPP5D*	rs473279	*INPP5B*
rs4663784	*INPP5D*	rs13432536	*INPP5D*	rs1400349	*INPP5D*	rs4653342	*INPP5B*
rs4663834	*INPP5D*	rs1400349	*INPP5D*	rs14243	*INPP5D*	rs4653342	*INPP5B*
rs4973063	*INPP5D*	rs14243	*INPP5D*	rs3792117	*INPP5D*	rs1400349	*INPP5D*
rs4973599	*INPP5D*	rs3792117	*INPP5D*	rs3890760	*INPP5D*	rs12694922	*INPP5D*
rs6431580	*INPP5D*	rs3890760	*INPP5D*	rs4073363	*INPP5D*	rs12694923	*INPP5D*
rs6431586	*INPP5D*	rs4073363	*INPP5D*	rs4257390	*INPP5D*	rs4246649	*INPP5D*
rs6437089	*INPP5D*	rs4257390	*INPP5D*	rs4335931	*INPP5D*	rs4973063	*INPP5D*
rs6715810	*INPP5D*	rs4335931	*INPP5D*	rs4356648	*INPP5D*	rs4439944	*INPP5D*
rs6720896	*INPP5D*	rs4356648	*INPP5D*	rs4436949	*INPP5D*	rs4257390	*INPP5D*
rs6740918	*INPP5D*	rs4436949	*INPP5D*	rs4439944	*INPP5D*	rs4073363	*INPP5D*
rs7425956	*INPP5D*	rs4439944	*INPP5D*	rs4468807	*INPP5D*	rs4973599	*INPP5D*
rs7566856	*INPP5D*	rs4468807	*INPP5D*	rs4503982	*INPP5D*	rs4315512	*INPP5D*
rs7569837	*INPP5D*	rs4503982	*INPP5D*	rs4973063	*INPP5D*	rs4603757	*INPP5D*
rs7570061	*INPP5D*	rs4973063	*INPP5D*	rs6431586	*INPP5D*	rs7425956	*INPP5D*
rs7580869	*INPP5D*	rs6431586	*INPP5D*	rs6437089	*INPP5D*	rs4356648	*INPP5D*
rs7584458	*INPP5D*	rs6437089	*INPP5D*	rs6715810	*INPP5D*	rs7608422	*INPP5D*
rs7608422	*INPP5D*	rs6715810	*INPP5D*	rs6720896	*INPP5D*	rs7566856	*INPP5D*
rs9247	*INPP5D*	rs6720896	*INPP5D*	rs6740918	*INPP5D*	rs4335931	*INPP5D*
rs9288685	*INPP5D*	rs6740918	*INPP5D*	rs7421653	*INPP5D*	rs6437089	*INPP5D*

rs9750891	*INPP5D*	rs7421653	*INPP5D*	rs7425956	*INPP5D*	rs9750891	*INPP5D*
rs1128877	*INPP5E*	rs7425956	*INPP5D*	rs7566856	*INPP5D*	rs7570061	*INPP5D*
rs1130635	*INPP5E*	rs7566856	*INPP5D*	rs7569837	*INPP5D*	rs9288685	*INPP5D*
rs1004243	*INPP5J*	rs7569837	*INPP5D*	rs7570061	*INPP5D*	rs10193128	*INPP5D*
rs2017301	*INPP5J*	rs7570061	*INPP5D*	rs7580869	*INPP5D*	rs10933435	*INPP5D*
rs2074736	*INPP5J*	rs7580869	*INPP5D*	rs7584458	*INPP5D*	rs11693862	*INPP5D*
rs2074739	*INPP5J*	rs7584458	*INPP5D*	rs7608422	*INPP5D*	rs11682728	*INPP5D*
rs2232176	*INPP5J*	rs7608422	*INPP5D*	rs9247	*INPP5D*	rs10929316	*INPP5D*
rs2232183	*INPP5J*	rs9247	*INPP5D*	rs9288685	*INPP5D*	rs11673739	*INPP5D*
rs2240430	*INPP5J*	rs9288685	*INPP5D*	rs9750891	*INPP5D*	rs6715810	*INPP5D*
rs2240432	*INPP5J*	rs9750891	*INPP5D*	rs1127152	*INPP5E*	rs10803668	*INPP5D*
rs3747152	*INPP5J*	rs1127152	*INPP5E*	rs1127162	*INPP5E*	rs7569837	*INPP5D*
rs3761431	*INPP5J*	rs1127162	*INPP5E*	rs1128877	*INPP5E*	rs13385922	*INPP5D*
rs3788428	*INPP5J*	rs1128877	*INPP5E*	rs1130635	*INPP5E*	rs6720896	*INPP5D*
rs4820944	*INPP5J*	rs1130635	*INPP5E*	rs2074736	*INPP5J*	rs4571051	*INPP5D*
rs5753463	*INPP5J*	rs2074736	*INPP5J*	rs2074739	*INPP5J*	rs4503982	*INPP5D*
rs5753469	*INPP5J*	rs2074739	*INPP5J*	rs2232176	*INPP5J*	rs4436949	*INPP5D*
rs5753472	*INPP5J*	rs2232176	*INPP5J*	rs2240430	*INPP5J*	rs6740918	*INPP5D*
rs5753480	*INPP5J*	rs2240430	*INPP5J*	rs2240432	*INPP5J*	rs7584458	*INPP5D*
rs5997872	*INPP5J*	rs2240432	*INPP5J*	rs2283877	*INPP5J*	rs3890760	*INPP5D*
rs8135641	*INPP5J*	rs2283877	*INPP5J*	rs3747152	*INPP5J*	rs10203185	*INPP5D*
rs917208	*INPP5J*	rs3747152	*INPP5J*	rs3761431	*INPP5J*	rs11674483	*INPP5D*
rs10521113	*INPP5K*	rs3761431	*INPP5J*	rs3788428	*INPP5J*	rs4468807	*INPP5D*
rs1109303	*INPP5K*	rs3788428	*INPP5J*	rs4820944	*INPP5J*	rs9247	*INPP5D*
rs15362	*INPP5K*	rs4820944	*INPP5J*	rs5753469	*INPP5J*	rs4663784	*INPP5D*
rs17761155	*INPP5K*	rs5753469	*INPP5J*	rs5753472	*INPP5J*	rs3792117	*INPP5D*
rs1879488	*INPP5K*	rs5753472	*INPP5J*	rs5753480	*INPP5J*	rs14243	*INPP5D*
rs2270227	*INPP5K*	rs5753480	*INPP5J*	rs5997872	*INPP5J*	rs13031194	*INPP5D*
rs2270229	*INPP5K*	rs5997872	*INPP5J*	rs8135641	*INPP5J*	rs4663834	*INPP5D*
rs2277669	*INPP5K*	rs8135641	*INPP5J*	rs10521113	*INPP5K*	rs6431580	*INPP5D*
rs2358973	*INPP5K*	rs10521113	*INPP5K*	rs1109303	*INPP5K*	rs6431586	*INPP5D*
rs7218128	*INPP5K*	rs1109303	*INPP5K*	rs11652315	*INPP5K*	rs13021302	*INPP5D*
rs11235468	*INPPL1*	rs11652315	*INPP5K*	rs15362	*INPP5K*	rs7580869	*INPP5D*
rs2276048	*INPPL1*	rs15362	*INPP5K*	rs1879488	*INPP5K*	rs1130635	*INPP5E*
rs514933	*INPPL1*	rs1879488	*INPP5K*	rs2270227	*INPP5K*	rs1128877	*INPP5E*
rs3749237	*IP6K1*	rs2270227	*INPP5K*	rs2270229	*INPP5K*	rs1127162	*INPP5E*
rs6802890	*IP6K1*	rs2270229	*INPP5K*	rs2277669	*INPP5K*	rs1127152	*INPP5E*
rs7629936	*IP6K1*	rs2277669	*INPP5K*	rs2358973	*INPP5K*	rs1127152	*INPP5E*
rs9829155	*IP6K1*	rs2358973	*INPP5K*	rs11235468	*INPPL1*	rs5997872	*INPP5J*
rs9855505	*IP6K1*	rs11235468	*INPPL1*	rs2276048	*INPPL1*	rs2074736	*INPP5J*
rs12497850	*IP6K2*	rs2276048	*INPPL1*	rs514933	*INPPL1*	rs2240430	*INPP5J*
rs3172494	*IP6K2*	rs514933	*INPPL1*	rs651933	*INPPL1*	rs917208	*INPP5J*
rs4077495	*IP6K2*	rs651933	*INPPL1*	rs7110260	*INPPL1*	rs1004243	*INPP5J*
rs6766238	*IP6K2*	rs7110260	*INPPL1*	rs3749237	*IP6K1*	rs5753463	*INPP5J*

rs9882443	*IP6K2*	rs3749237	*IP6K1*	rs6802890	*IP6K1*	rs2283877	*INPP5J*
rs990211	*IP6K2*	rs6802890	*IP6K1*	rs7629936	*IP6K1*	rs2017301	*INPP5J*
rs10947433	*IP6K3*	rs7629936	*IP6K1*	rs9855505	*IP6K1*	rs3747152	*INPP5J*
rs10947435	*IP6K3*	rs9855505	*IP6K1*	rs12497850	*IP6K2*	rs2240432	*INPP5J*
rs12211490	*IP6K3*	rs12497850	*IP6K2*	rs4077495	*IP6K2*	rs5753469	*INPP5J*
rs1536500	*IP6K3*	rs4077495	*IP6K2*	rs10947435	*IP6K3*	rs8135641	*INPP5J*
rs1536501	*IP6K3*	rs10947435	*IP6K3*	rs12211490	*IP6K3*	rs2232183	*INPP5J*
rs2281829	*IP6K3*	rs12211490	*IP6K3*	rs2281829	*IP6K3*	rs5753472	*INPP5J*
rs2966	*IP6K3*	rs2281829	*IP6K3*	rs2966	*IP6K3*	rs2074739	*INPP5J*
rs3818532	*IP6K3*	rs2966	*IP6K3*	rs4304152	*IP6K3*	rs2232176	*INPP5J*
rs4304152	*IP6K3*	rs4304152	*IP6K3*	rs4711345	*IP6K3*	rs3788428	*INPP5J*
rs4711345	*IP6K3*	rs4711345	*IP6K3*	rs4711348	*IP6K3*	rs3761431	*INPP5J*
rs4711348	*IP6K3*	rs4711348	*IP6K3*	rs4713668	*IP6K3*	rs5753480	*INPP5J*
rs4713668	*IP6K3*	rs4713668	*IP6K3*	rs542441	*IP6K3*	rs4820944	*INPP5J*
rs471942	*IP6K3*	rs542441	*IP6K3*	rs622917	*IP6K3*	rs2358973	*INPP5K*
rs498114	*IP6K3*	rs622917	*IP6K3*	rs6457740	*IP6K3*	rs7218128	*INPP5K*
rs542441	*IP6K3*	rs6457740	*IP6K3*	rs6904716	*IP6K3*	rs1879488	*INPP5K*
rs622917	*IP6K3*	rs6904716	*IP6K3*	rs6919321	*IP6K3*	rs1109303	*INPP5K*
rs6457740	*IP6K3*	rs6919321	*IP6K3*	rs6933607	*IP6K3*	rs15362	*INPP5K*
rs649775	*IP6K3*	rs6933607	*IP6K3*	rs755495	*IP6K3*	rs2277669	*INPP5K*
rs652049	*IP6K3*	rs755495	*IP6K3*	rs9380374	*IP6K3*	rs10521113	*INPP5K*
rs6904716	*IP6K3*	rs9380374	*IP6K3*	rs9380376	*IP6K3*	rs17761155	*INPP5K*
rs6919321	*IP6K3*	rs9380376	*IP6K3*	rs9469583	*IP6K3*	rs2270227	*INPP5K*
rs6933607	*IP6K3*	rs9469583	*IP6K3*	rs11006086	*IPMK*	rs2270229	*INPP5K*
rs755495	*IP6K3*	rs11006086	*IPMK*	rs1199098	*IPMK*	rs11235468	*INPPL1*
rs9380374	*IP6K3*	rs1199098	*IPMK*	rs1416764	*IPMK*	rs651933	*INPPL1*
rs9380376	*IP6K3*	rs1416764	*IPMK*	rs1867571	*IPMK*	rs514933	*INPPL1*
rs9469583	*IP6K3*	rs1867571	*IPMK*	rs2275442	*IPMK*	rs2276048	*INPPL1*
rs11006086	*IPMK*	rs2275442	*IPMK*	rs6481383	*IPMK*	rs7110260	*INPPL1*
rs1199098	*IPMK*	rs6481383	*IPMK*	rs7068428	*IPMK*	rs7110260	*INPPL1*
rs6481383	*IPMK*	rs7068428	*IPMK*	rs7087498	*IPMK*	rs3749237	*IP6K1*
rs7068428	*IPMK*	rs7087498	*IPMK*	rs7899961	*IPMK*	rs9855505	*IP6K1*
rs7899961	*IPMK*	rs7899961	*IPMK*	rs10992420	*IPPK*	rs9829155	*IP6K1*
rs1980705	*IPPK*	rs10992420	*IPPK*	rs1980705	*IPPK*	rs6802890	*IP6K1*
rs7043114	*IPPK*	rs1980705	*IPPK*	rs7043114	*IPPK*	rs7629936	*IP6K1*
rs7863890	*IPPK*	rs7043114	*IPPK*	rs7863890	*IPPK*	rs990211	*IP6K2*
rs9969804	*IPPK*	rs7863890	*IPPK*	rs912261	*IPPK*	rs3172494	*IP6K2*
rs13344313	*ISYNA1*	rs912261	*IPPK*	rs9969804	*IPPK*	rs12497850	*IP6K2*
rs731945	*ISYNA1*	rs9969804	*IPPK*	rs731945	*ISYNA1*	rs9882443	*IP6K2*
rs1006888	*ITPK1*	rs731945	*ISYNA1*	rs1006888	*ITPK1*	rs4077495	*IP6K2*
rs10136012	*ITPK1*	rs1006888	*ITPK1*	rs1043542	*ITPK1*	rs6766238	*IP6K2*
rs1043542	*ITPK1*	rs1043542	*ITPK1*	rs11446	*ITPK1*	rs498114	*IP6K3*
rs11446	*ITPK1*	rs11446	*ITPK1*	rs11625662	*ITPK1*	rs2281829	*IP6K3*
rs11625662	*ITPK1*	rs11625662	*ITPK1*	rs11628021	*ITPK1*	rs542441	*IP6K3*

rs11628021	*ITPK1*	rs11628021	*ITPK1*	rs12434958	*ITPK1*	rs9380374	*IP6K3*
rs12435325	*ITPK1*	rs12434958	*ITPK1*	rs12435325	*ITPK1*	rs3818532	*IP6K3*
rs12435423	*ITPK1*	rs12435325	*ITPK1*	rs12435423	*ITPK1*	rs649775	*IP6K3*
rs12586382	*ITPK1*	rs12435423	*ITPK1*	rs12586382	*ITPK1*	rs2966	*IP6K3*
rs12587187	*ITPK1*	rs12586382	*ITPK1*	rs12587187	*ITPK1*	rs10947433	*IP6K3*
rs12589455	*ITPK1*	rs12587187	*ITPK1*	rs12589455	*ITPK1*	rs4713668	*IP6K3*
rs1612612	*ITPK1*	rs12589455	*ITPK1*	rs12895695	*ITPK1*	rs471942	*IP6K3*
rs17128706	*ITPK1*	rs12895695	*ITPK1*	rs1612612	*ITPK1*	rs6457740	*IP6K3*
rs17128737	*ITPK1*	rs1612612	*ITPK1*	rs17128706	*ITPK1*	rs622917	*IP6K3*
rs1740596	*ITPK1*	rs17128706	*ITPK1*	rs17128737	*ITPK1*	rs652049	*IP6K3*
rs1740598	*ITPK1*	rs17128737	*ITPK1*	rs1740596	*ITPK1*	rs9469583	*IP6K3*
rs1740689	*ITPK1*	rs1740596	*ITPK1*	rs1740598	*ITPK1*	rs10947435	*IP6K3*
rs1740694	*ITPK1*	rs1740598	*ITPK1*	rs1740689	*ITPK1*	rs4304152	*IP6K3*
rs1740696	*ITPK1*	rs1740689	*ITPK1*	rs1740694	*ITPK1*	rs1536500	*IP6K3*
rs1740698	*ITPK1*	rs1740694	*ITPK1*	rs1740696	*ITPK1*	rs6919321	*IP6K3*
rs2180369	*ITPK1*	rs1740696	*ITPK1*	rs1740698	*ITPK1*	rs9380376	*IP6K3*
rs2295394	*ITPK1*	rs1740698	*ITPK1*	rs2180369	*ITPK1*	rs12211490	*IP6K3*
rs2402226	*ITPK1*	rs2180369	*ITPK1*	rs2295394	*ITPK1*	rs1536501	*IP6K3*
rs2749509	*ITPK1*	rs2295394	*ITPK1*	rs2402226	*ITPK1*	rs4711345	*IP6K3*
rs3783910	*ITPK1*	rs2402226	*ITPK1*	rs2749509	*ITPK1*	rs755495	*IP6K3*
rs3783913	*ITPK1*	rs2749509	*ITPK1*	rs3783910	*ITPK1*	rs4711348	*IP6K3*
rs3783914	*ITPK1*	rs3783910	*ITPK1*	rs3783913	*ITPK1*	rs6904716	*IP6K3*
rs3783919	*ITPK1*	rs3783913	*ITPK1*	rs3783914	*ITPK1*	rs6933607	*IP6K3*
rs3783925	*ITPK1*	rs3783914	*ITPK1*	rs3783919	*ITPK1*	rs6933607	*IP6K3*
rs4586354	*ITPK1*	rs3783919	*ITPK1*	rs3783925	*ITPK1*	rs1199098	*IPMK*
rs4900164	*ITPK1*	rs3783925	*ITPK1*	rs4586354	*ITPK1*	rs7068428	*IPMK*
rs4905029	*ITPK1*	rs4586354	*ITPK1*	rs4900164	*ITPK1*	rs11006086	*IPMK*
rs4905043	*ITPK1*	rs4900164	*ITPK1*	rs4905029	*ITPK1*	rs6481383	*IPMK*
rs749619	*ITPK1*	rs4905029	*ITPK1*	rs4905043	*ITPK1*	rs7899961	*IPMK*
rs768356	*ITPK1*	rs4905043	*ITPK1*	rs749619	*ITPK1*	rs1867571	*IPMK*
rs941541	*ITPK1*	rs749619	*ITPK1*	rs768356	*ITPK1*	rs1416764	*IPMK*
rs941542	*ITPK1*	rs768356	*ITPK1*	rs941542	*ITPK1*	rs1416764	*IPMK*
rs941578	*ITPK1*	rs941542	*ITPK1*	rs957362	*ITPK1*	rs1980705	*IPPK*
rs957362	*ITPK1*	rs957362	*ITPK1*	rs1757463	*ITPKA*	rs7043114	*IPPK*
rs1757463	*ITPKA*	rs1757463	*ITPKA*	rs2305030	*ITPKA*	rs7863890	*IPPK*
rs10495249	*ITPKB*	rs2305030	*ITPKA*	rs10495249	*ITPKB*	rs9969804	*IPPK*
rs1050492	*ITPKB*	rs10495249	*ITPKB*	rs10916019	*ITPKB*	rs4744143	*IPPK*
rs10916019	*ITPKB*	rs10916019	*ITPKB*	rs1144836	*ITPKB*	rs912261	*IPPK*
rs1144836	*ITPKB*	rs1144836	*ITPKB*	rs1144838	*ITPKB*	rs13285641	*IPPK*
rs1144838	*ITPKB*	rs1144838	*ITPKB*	rs1144841	*ITPKB*	rs10992420	*IPPK*
rs1144841	*ITPKB*	rs1144841	*ITPKB*	rs12077348	*ITPKB*	rs10992420	*IPPK*
rs12077348	*ITPKB*	rs12077348	*ITPKB*	rs12094617	*ITPKB*	rs731945	*ISYNA1*
rs12094617	*ITPKB*	rs12094617	*ITPKB*	rs1288934	*ITPKB*	rs731945	*ISYNA1*
rs1341283	*ITPKB*	rs1288934	*ITPKB*	rs1341283	*ITPKB*	rs1043542	*ITPK1*

rs17522524	*ITPKB*	rs1341283	*ITPKB*	rs17522524	*ITPKB*	rs11446	*ITPK1*
rs2236604	*ITPKB*	rs17522524	*ITPKB*	rs2236604	*ITPKB*	rs3783925	*ITPK1*
rs3754378	*ITPKB*	rs2236604	*ITPKB*	rs3754378	*ITPKB*	rs2295394	*ITPK1*
rs3754390	*ITPKB*	rs3754378	*ITPKB*	rs3754390	*ITPKB*	rs4900164	*ITPK1*
rs3768373	*ITPKB*	rs3754390	*ITPKB*	rs3754407	*ITPKB*	rs1006888	*ITPK1*
rs3768405	*ITPKB*	rs3754407	*ITPKB*	rs3768373	*ITPKB*	rs2402226	*ITPK1*
rs3768408	*ITPKB*	rs3768373	*ITPKB*	rs3768405	*ITPKB*	rs4586354	*ITPK1*
rs3768414	*ITPKB*	rs3768405	*ITPKB*	rs3768408	*ITPKB*	rs11625662	*ITPK1*
rs6667260	*ITPKB*	rs3768408	*ITPKB*	rs3768414	*ITPKB*	rs941578	*ITPK1*
rs697845	*ITPKB*	rs3768414	*ITPKB*	rs3820635	*ITPKB*	rs12587187	*ITPK1*
rs697851	*ITPKB*	rs3820635	*ITPKB*	rs6667260	*ITPKB*	rs3783919	*ITPK1*
rs708766	*ITPKB*	rs6667260	*ITPKB*	rs697845	*ITPKB*	rs17128706	*ITPK1*
rs708772	*ITPKB*	rs697845	*ITPKB*	rs697851	*ITPKB*	rs3783914	*ITPK1*
rs708776	*ITPKB*	rs697851	*ITPKB*	rs708766	*ITPKB*	rs12589455	*ITPK1*
rs10420685	*ITPKC*	rs708766	*ITPKB*	rs708772	*ITPKB*	rs3783913	*ITPK1*
rs11668501	*ITPKC*	rs708772	*ITPKB*	rs708777	*ITPKB*	rs12435423	*ITPK1*
rs1870087	*ITPKC*	rs708777	*ITPKB*	rs10420685	*ITPKC*	rs12435325	*ITPK1*
rs2604913	*ITPKC*	rs10420685	*ITPKC*	rs1870087	*ITPKC*	rs12586382	*ITPK1*
rs3745216	*ITPKC*	rs1870087	*ITPKC*	rs2604913	*ITPKC*	rs4905029	*ITPK1*
rs3865451	*ITPKC*	rs2604913	*ITPKC*	rs3745216	*ITPKC*	rs17128737	*ITPK1*
rs3865452	*ITPKC*	rs3745216	*ITPKC*	rs3865451	*ITPKC*	rs3783910	*ITPK1*
rs890934	*ITPKC*	rs3865451	*ITPKC*	rs3865452	*ITPKC*	rs11628021	*ITPK1*
rs10509407	*MINPP1*	rs3865452	*ITPKC*	rs890934	*ITPKC*	rs1612612	*ITPK1*
rs10509408	*MINPP1*	rs890934	*ITPKC*	rs10509407	*MINPP1*	rs1740696	*ITPK1*
rs11202426	*MINPP1*	rs10509407	*MINPP1*	rs10509408	*MINPP1*	rs1740598	*ITPK1*
rs11202429	*MINPP1*	rs10509408	*MINPP1*	rs10788550	*MINPP1*	rs10136012	*ITPK1*
rs1408377	*MINPP1*	rs10788550	*MINPP1*	rs11202426	*MINPP1*	rs1740596	*ITPK1*
rs2311115	*MINPP1*	rs11202426	*MINPP1*	rs11202429	*MINPP1*	rs1740694	*ITPK1*
rs3843597	*MINPP1*	rs11202429	*MINPP1*	rs11202434	*MINPP1*	rs2180369	*ITPK1*
rs3847448	*MINPP1*	rs11202434	*MINPP1*	rs1408377	*MINPP1*	rs941542	*ITPK1*
rs3847452	*MINPP1*	rs1408377	*MINPP1*	rs2147287	*MINPP1*	rs749619	*ITPK1*
rs4824152	*MIOX*	rs2147287	*MINPP1*	rs2311115	*MINPP1*	rs2749509	*ITPK1*
rs9616854	*MIOX*	rs2311115	*MINPP1*	rs2871690	*MINPP1*	rs1740689	*ITPK1*
rs1057691	*NUDT3*	rs2871690	*MINPP1*	rs3843597	*MINPP1*	rs1740698	*ITPK1*
rs10947494	*NUDT3*	rs3843597	*MINPP1*	rs3847448	*MINPP1*	rs4905043	*ITPK1*
rs16883137	*NUDT3*	rs3847448	*MINPP1*	rs3847452	*MINPP1*	rs941541	*ITPK1*
rs206937	*NUDT3*	rs3847452	*MINPP1*	rs8138406	*MIOX*	rs957362	*ITPK1*
rs3798560	*NUDT3*	rs8138406	*MIOX*	rs9616854	*MIOX*	rs768356	*ITPK1*
rs464553	*NUDT3*	rs9616854	*MIOX*	rs10947494	*NUDT3*	rs12895695	*ITPK1*
rs11107007	*NUDT4*	rs10947494	*NUDT3*	rs206937	*NUDT3*	rs12434958	*ITPK1*
rs11107008	*NUDT4*	rs206937	*NUDT3*	rs3798560	*NUDT3*	rs12434958	*ITPK1*
rs12597	*NUDT4*	rs3798560	*NUDT3*	rs464553	*NUDT3*	rs1757463	*ITPKA*
rs12816436	*NUDT4*	rs464553	*NUDT3*	rs11107007	*NUDT4*	rs170296	*ITPKA*
rs17790482	*NUDT4*	rs11107007	*NUDT4*	rs11107008	*NUDT4*	rs2305030	*ITPKA*

rs17837158	*NUDT4*	rs11107008	*NUDT4*	rs12597	*NUDT4*	rs2305030	*ITPKA*
rs4247307	*NUDT4*	rs12597	*NUDT4*	rs17837158	*NUDT4*	rs10916019	*ITPKB*
rs4761517	*NUDT4*	rs17837158	*NUDT4*	rs4247307	*NUDT4*	rs697845	*ITPKB*
rs7487813	*NUDT4*	rs4247307	*NUDT4*	rs4761517	*NUDT4*	rs1144841	*ITPKB*
rs7973701	*NUDT4*	rs4761517	*NUDT4*	rs7973701	*NUDT4*	rs1144838	*ITPKB*
rs7977140	*NUDT4*	rs7973701	*NUDT4*	rs7977140	*NUDT4*	rs3754378	*ITPKB*
rs10444068	*PI4K2A*	rs7977140	*NUDT4*	rs10786364	*PI4K2A*	rs2236604	*ITPKB*
rs10786364	*PI4K2A*	rs10786364	*PI4K2A*	rs11189310	*PI4K2A*	rs1288934	*ITPKB*
rs11189310	*PI4K2A*	rs11189310	*PI4K2A*	rs11189321	*PI4K2A*	rs17522524	*ITPKB*
rs11189321	*PI4K2A*	rs11189321	*PI4K2A*	rs12253425	*PI4K2A*	rs3820635	*ITPKB*
rs2065672	*PI4K2A*	rs12253425	*PI4K2A*	rs2065672	*PI4K2A*	rs1144836	*ITPKB*
rs3890727	*PI4K2A*	rs2065672	*PI4K2A*	rs3890727	*PI4K2A*	rs3768373	*ITPKB*
rs4919128	*PI4K2A*	rs3890727	*PI4K2A*	rs4919128	*PI4K2A*	rs12077348	*ITPKB*
rs6584138	*PI4K2A*	rs4919128	*PI4K2A*	rs6584138	*PI4K2A*	rs3754390	*ITPKB*
rs3115231	*PI4K2B*	rs6584138	*PI4K2A*	rs11737332	*PI4K2B*	rs12094617	*ITPKB*
rs313533	*PI4K2B*	rs11737332	*PI4K2B*	rs3115231	*PI4K2B*	rs708766	*ITPKB*
rs313541	*PI4K2B*	rs3115231	*PI4K2B*	rs313533	*PI4K2B*	rs697851	*ITPKB*
rs313548	*PI4K2B*	rs313533	*PI4K2B*	rs313541	*PI4K2B*	rs1341283	*ITPKB*
rs313566	*PI4K2B*	rs313541	*PI4K2B*	rs313548	*PI4K2B*	rs3768405	*ITPKB*
rs3796780	*PI4K2B*	rs313548	*PI4K2B*	rs313566	*PI4K2B*	rs3768408	*ITPKB*
rs7661189	*PI4K2B*	rs313566	*PI4K2B*	rs3796780	*PI4K2B*	rs708772	*ITPKB*
rs1558657	*PI4KA*	rs3796780	*PI4K2B*	rs7661189	*PI4K2B*	rs3768414	*ITPKB*
rs165793	*PI4KA*	rs7661189	*PI4K2B*	rs10483104	*PI4KA*	rs10495249	*ITPKB*
rs165862	*PI4KA*	rs10483104	*PI4KA*	rs1558657	*PI4KA*	rs708776	*ITPKB*
rs165924	*PI4KA*	rs11705170	*PI4KA*	rs165598	*PI4KA*	rs6667260	*ITPKB*
rs178051	*PI4KA*	rs1558657	*PI4KA*	rs165793	*PI4KA*	rs708777	*ITPKB*
rs178058	*PI4KA*	rs165598	*PI4KA*	rs165862	*PI4KA*	rs708777	*ITPKB*
rs17820181	*PI4KA*	rs165793	*PI4KA*	rs165924	*PI4KA*	rs3865451	*ITPKC*
rs2072513	*PI4KA*	rs165862	*PI4KA*	rs178058	*PI4KA*	rs3865452	*ITPKC*
rs4822606	*PI4KA*	rs165924	*PI4KA*	rs178070	*PI4KA*	rs2604913	*ITPKC*
rs9608386	*PI4KA*	rs178058	*PI4KA*	rs2072513	*PI4KA*	rs890934	*ITPKC*
rs1056847	*PI4KB*	rs178070	*PI4KA*	rs4822606	*PI4KA*	rs11668501	*ITPKC*
rs1752382	*PI4KB*	rs17820181	*PI4KA*	rs9608386	*PI4KA*	rs10420685	*ITPKC*
rs1752388	*PI4KB*	rs2072513	*PI4KA*	rs1056847	*PI4KB*	rs3745216	*ITPKC*
rs2031797	*PI4KB*	rs4822606	*PI4KA*	rs1752382	*PI4KB*	rs1870087	*ITPKC*
rs2298265	*PI4KB*	rs9608386	*PI4KA*	rs1752388	*PI4KB*	rs11202426	*MINPP1*
rs4971030	*PI4KB*	rs1056847	*PI4KB*	rs2031797	*PI4KB*	rs10509407	*MINPP1*
rs5022636	*PI4KB*	rs1752382	*PI4KB*	rs2298265	*PI4KB*	rs3843597	*MINPP1*
rs11604561	*PIK3C2A*	rs1752388	*PI4KB*	rs4971030	*PI4KB*	rs2311115	*MINPP1*
rs2040859	*PIK3C2A*	rs2031797	*PI4KB*	rs5022636	*PI4KB*	rs11202429	*MINPP1*
rs3950680	*PIK3C2A*	rs2298265	*PI4KB*	rs11604561	*PIK3C2A*	rs3847448	*MINPP1*
rs7946010	*PIK3C2A*	rs4971030	*PI4KB*	rs3950680	*PIK3C2A*	rs3847452	*MINPP1*
rs11240748	*PIK3C2B*	rs5022636	*PI4KB*	rs621246	*PIK3C2A*	rs10509408	*MINPP1*
rs12031854	*PIK3C2B*	rs3950680	*PIK3C2A*	rs7946010	*PIK3C2A*	rs1408377	*MINPP1*

rs12061474	*PIK3C2B*	rs621246	*PIK3C2A*	rs2271421	*PIK3C2B*	rs2147287	*MINPP1*
rs12119503	*PIK3C2B*	rs7946010	*PIK3C2A*	rs2271424	*PIK3C2B*	rs10788550	*MINPP1*
rs17334387	*PIK3C2B*	rs2271421	*PIK3C2B*	rs2271427	*PIK3C2B*	rs10788550	*MINPP1*
rs2137255	*PIK3C2B*	rs2271424	*PIK3C2B*	rs11240748	*PIK3C2B*	rs4824152	*MIOX*
rs2271415	*PIK3C2B*	rs2271427	*PIK3C2B*	rs12031854	*PIK3C2B*	rs9616854	*MIOX*
rs2271421	*PIK3C2B*	rs11240748	*PIK3C2B*	rs12061474	*PIK3C2B*	rs4824157	*MIOX*
rs2271424	*PIK3C2B*	rs12031854	*PIK3C2B*	rs1553920	*PIK3C2B*	rs8138406	*MIOX*
rs2271427	*PIK3C2B*	rs12061474	*PIK3C2B*	rs16853737	*PIK3C2B*	rs8138406	*MIOX*
rs2999484	*PIK3C2B*	rs1553920	*PIK3C2B*	rs17334387	*PIK3C2B*	rs16883137	*NUDT3*
rs3014637	*PIK3C2B*	rs16853737	*PIK3C2B*	rs2137255	*PIK3C2B*	rs10947494	*NUDT3*
rs3106366	*PIK3C2B*	rs17334387	*PIK3C2B*	rs2271415	*PIK3C2B*	rs1057691	*NUDT3*
rs3747633	*PIK3C2B*	rs2137255	*PIK3C2B*	rs2999484	*PIK3C2B*	rs464553	*NUDT3*
rs3747636	*PIK3C2B*	rs2271415	*PIK3C2B*	rs3014637	*PIK3C2B*	rs206937	*NUDT3*
rs4951373	*PIK3C2B*	rs2999484	*PIK3C2B*	rs3106366	*PIK3C2B*	rs3798560	*NUDT3*
rs6594014	*PIK3C2B*	rs3014637	*PIK3C2B*	rs3747633	*PIK3C2B*	rs4761517	*NUDT4*
rs7556371	*PIK3C2B*	rs3106366	*PIK3C2B*	rs3747636	*PIK3C2B*	rs4247307	*NUDT4*
rs10160860	*PIK3C2G*	rs3747633	*PIK3C2B*	rs4951373	*PIK3C2B*	rs11107007	*NUDT4*
rs10505810	*PIK3C2G*	rs3747636	*PIK3C2B*	rs6594014	*PIK3C2B*	rs17837158	*NUDT4*
rs10505811	*PIK3C2G*	rs4951373	*PIK3C2B*	rs7554895	*PIK3C2B*	rs11107008	*NUDT4*
rs10505824	*PIK3C2G*	rs6594014	*PIK3C2B*	rs7556371	*PIK3C2B*	rs7487813	*NUDT4*
rs10743273	*PIK3C2G*	rs7554895	*PIK3C2B*	rs10505810	*PIK3C2G*	rs7977140	*NUDT4*
rs10770333	*PIK3C2G*	rs7556371	*PIK3C2B*	rs10505824	*PIK3C2G*	rs17790482	*NUDT4*
rs10770359	*PIK3C2G*	rs10505810	*PIK3C2G*	rs10734680	*PIK3C2G*	rs12597	*NUDT4*
rs10770372	*PIK3C2G*	rs10505824	*PIK3C2G*	rs10743273	*PIK3C2G*	rs7973701	*NUDT4*
rs10841019	*PIK3C2G*	rs10734680	*PIK3C2G*	rs10770333	*PIK3C2G*	rs12816436	*NUDT4*
rs10841023	*PIK3C2G*	rs10743273	*PIK3C2G*	rs10770359	*PIK3C2G*	rs6584138	*PI4K2A*
rs10841025	*PIK3C2G*	rs10770333	*PIK3C2G*	rs10841019	*PIK3C2G*	rs11189310	*PI4K2A*
rs10841036	*PIK3C2G*	rs10770359	*PIK3C2G*	rs10841023	*PIK3C2G*	rs3890727	*PI4K2A*
rs10841041	*PIK3C2G*	rs10841019	*PIK3C2G*	rs10841025	*PIK3C2G*	rs4919128	*PI4K2A*
rs10841043	*PIK3C2G*	rs10841023	*PIK3C2G*	rs10841036	*PIK3C2G*	rs2065672	*PI4K2A*
rs10841048	*PIK3C2G*	rs10841025	*PIK3C2G*	rs10841049	*PIK3C2G*	rs11189321	*PI4K2A*
rs10841049	*PIK3C2G*	rs10841036	*PIK3C2G*	rs11044004	*PIK3C2G*	rs10444068	*PI4K2A*
rs11044004	*PIK3C2G*	rs10841049	*PIK3C2G*	rs11044026	*PIK3C2G*	rs10786364	*PI4K2A*
rs11044026	*PIK3C2G*	rs11044004	*PIK3C2G*	rs11044058	*PIK3C2G*	rs3115231	*PI4K2B*
rs11044058	*PIK3C2G*	rs11044026	*PIK3C2G*	rs11044070	*PIK3C2G*	rs313548	*PI4K2B*
rs11044070	*PIK3C2G*	rs11044058	*PIK3C2G*	rs11044075	*PIK3C2G*	rs7661189	*PI4K2B*
rs11044075	*PIK3C2G*	rs11044070	*PIK3C2G*	rs11044082	*PIK3C2G*	rs313566	*PI4K2B*
rs11044082	*PIK3C2G*	rs11044075	*PIK3C2G*	rs11044084	*PIK3C2G*	rs313541	*PI4K2B*
rs11044084	*PIK3C2G*	rs11044082	*PIK3C2G*	rs11044103	*PIK3C2G*	rs313533	*PI4K2B*
rs11044103	*PIK3C2G*	rs11044084	*PIK3C2G*	rs11044155	*PIK3C2G*	rs3796780	*PI4K2B*
rs11044165	*PIK3C2G*	rs11044103	*PIK3C2G*	rs11044165	*PIK3C2G*	rs11705170	*PI4KA*
rs11044171	*PIK3C2G*	rs11044155	*PIK3C2G*	rs11044232	*PIK3C2G*	rs1558657	*PI4KA*
rs11044223	*PIK3C2G*	rs11044165	*PIK3C2G*	rs11044233	*PIK3C2G*	rs2072513	*PI4KA*
rs11044232	*PIK3C2G*	rs11044232	*PIK3C2G*	rs12227441	*PIK3C2G*	rs9608386	*PI4KA*

rs12227441	*PIK3C2G*	rs11044233	*PIK3C2G*	rs12312266	*PIK3C2G*	rs165862	*PI4KA*
rs12297325	*PIK3C2G*	rs12227441	*PIK3C2G*	rs12367676	*PIK3C2G*	rs17820181	*PI4KA*
rs12309567	*PIK3C2G*	rs12312266	*PIK3C2G*	rs12371624	*PIK3C2G*	rs165793	*PI4KA*
rs12312266	*PIK3C2G*	rs12367676	*PIK3C2G*	rs12422650	*PIK3C2G*	rs165924	*PI4KA*
rs12367676	*PIK3C2G*	rs12371624	*PIK3C2G*	rs12427286	*PIK3C2G*	rs178051	*PI4KA*
rs12371624	*PIK3C2G*	rs12422650	*PIK3C2G*	rs12581078	*PIK3C2G*	rs178058	*PI4KA*
rs12427286	*PIK3C2G*	rs12427286	*PIK3C2G*	rs12581163	*PIK3C2G*	rs4822606	*PI4KA*
rs12581078	*PIK3C2G*	rs12581078	*PIK3C2G*	rs12582971	*PIK3C2G*	rs165598	*PI4KA*
rs12581163	*PIK3C2G*	rs12581163	*PIK3C2G*	rs12818409	*PIK3C2G*	rs178070	*PI4KA*
rs12582971	*PIK3C2G*	rs12582971	*PIK3C2G*	rs12821147	*PIK3C2G*	rs10483104	*PI4KA*
rs12818409	*PIK3C2G*	rs12818409	*PIK3C2G*	rs12825580	*PIK3C2G*	rs10916019	*PI4KB*
rs12821147	*PIK3C2G*	rs12821147	*PIK3C2G*	rs12827287	*PIK3C2G*	rs697845	*PI4KB*
rs12822135	*PIK3C2G*	rs12825580	*PIK3C2G*	rs1374670	*PIK3C2G*	rs1144841	*PI4KB*
rs12825580	*PIK3C2G*	rs12827287	*PIK3C2G*	rs1447406	*PIK3C2G*	rs1144838	*PI4KB*
rs12827287	*PIK3C2G*	rs1374670	*PIK3C2G*	rs1447408	*PIK3C2G*	rs3754378	*PI4KB*
rs1374670	*PIK3C2G*	rs1447406	*PIK3C2G*	rs1447411	*PIK3C2G*	rs2236604	*PI4KB*
rs1447406	*PIK3C2G*	rs1447408	*PIK3C2G*	rs17418422	*PIK3C2G*	rs1288934	*PI4KB*
rs1447408	*PIK3C2G*	rs1447411	*PIK3C2G*	rs1816971	*PIK3C2G*	rs17522524	*PI4KB*
rs17409120	*PIK3C2G*	rs17418422	*PIK3C2G*	rs1868064	*PIK3C2G*	rs3820635	*PI4KB*
rs17411508	*PIK3C2G*	rs1816971	*PIK3C2G*	rs1992838	*PIK3C2G*	rs1144836	*PI4KB*
rs17418422	*PIK3C2G*	rs1868064	*PIK3C2G*	rs2277328	*PIK3C2G*	rs3768373	*PI4KB*
rs17419409	*PIK3C2G*	rs1992838	*PIK3C2G*	rs2290044	*PIK3C2G*	rs12077348	*PI4KB*
rs1816971	*PIK3C2G*	rs2277328	*PIK3C2G*	rs2305220	*PIK3C2G*	rs3754390	*PI4KB*
rs1868064	*PIK3C2G*	rs2290044	*PIK3C2G*	rs2931484	*PIK3C2G*	rs12094617	*PI4KB*
rs1992838	*PIK3C2G*	rs2305220	*PIK3C2G*	rs3813896	*PIK3C2G*	rs708766	*PI4KB*
rs2277328	*PIK3C2G*	rs2931484	*PIK3C2G*	rs4369463	*PIK3C2G*	rs697851	*PI4KB*
rs2290044	*PIK3C2G*	rs3813896	*PIK3C2G*	rs4385947	*PIK3C2G*	rs1341283	*PI4KB*
rs2305220	*PIK3C2G*	rs4369463	*PIK3C2G*	rs4488254	*PIK3C2G*	rs3768405	*PI4KB*
rs3813896	*PIK3C2G*	rs4385947	*PIK3C2G*	rs4534639	*PIK3C2G*	rs3768408	*PI4KB*
rs4369463	*PIK3C2G*	rs4488254	*PIK3C2G*	rs4595599	*PIK3C2G*	rs708772	*PI4KB*
rs4385947	*PIK3C2G*	rs4534639	*PIK3C2G*	rs4609650	*PIK3C2G*	rs3768414	*PI4KB*
rs4534639	*PIK3C2G*	rs4595599	*PIK3C2G*	rs4763508	*PIK3C2G*	rs10495249	*PI4KB*
rs4595599	*PIK3C2G*	rs4609650	*PIK3C2G*	rs4764409	*PIK3C2G*	rs708776	*PI4KB*
rs4609650	*PIK3C2G*	rs4763508	*PIK3C2G*	rs4764412	*PIK3C2G*	rs6667260	*PI4KB*
rs4763508	*PIK3C2G*	rs4764409	*PIK3C2G*	rs518345	*PIK3C2G*	rs708777	*PI4KB*
rs4764409	*PIK3C2G*	rs4764412	*PIK3C2G*	rs531697	*PIK3C2G*	rs708777	*PI4KB*
rs518345	*PIK3C2G*	rs518345	*PIK3C2G*	rs578947	*PIK3C2G*	rs11604561	*PIK3C2A*
rs621042	*PIK3C2G*	rs531697	*PIK3C2G*	rs621042	*PIK3C2G*	rs3950680	*PIK3C2A*
rs644164	*PIK3C2G*	rs578947	*PIK3C2G*	rs644164	*PIK3C2G*	rs2040859	*PIK3C2A*
rs666864	*PIK3C2G*	rs621042	*PIK3C2G*	rs666864	*PIK3C2G*	rs7946010	*PIK3C2A*
rs7133666	*PIK3C2G*	rs644164	*PIK3C2G*	rs719568	*PIK3C2G*	rs621246	*PIK3C2A*
rs719568	*PIK3C2G*	rs666864	*PIK3C2G*	rs7308591	*PIK3C2G*	rs621246	*PIK3C2A*
rs7308591	*PIK3C2G*	rs719568	*PIK3C2G*	rs7314398	*PIK3C2G*	rs12119503	*PIK3C2B*
rs7314398	*PIK3C2G*	rs7314398	*PIK3C2G*	rs7957373	*PIK3C2G*	rs12061474	*PIK3C2B*

rs7957373	*PIK3C2G*	rs7957373	*PIK3C2G*	rs7964182	*PIK3C2G*	rs4951373	*PIK3C2B*
rs7964182	*PIK3C2G*	rs7964182	*PIK3C2G*	rs9300118	*PIK3C2G*	rs17334387	*PIK3C2B*
rs7969452	*PIK3C2G*	rs7969452	*PIK3C2G*	rs9634063	*PIK3C2G*	rs2271421	*PIK3C2B*
rs9300118	*PIK3C2G*	rs9300118	*PIK3C2G*	rs982754	*PIK3C2G*	rs2271424	*PIK3C2B*
rs9634063	*PIK3C2G*	rs982754	*PIK3C2G*	rs1941526	*PIK3C3*	rs3747636	*PIK3C2B*
rs12954899	*PIK3C3*	rs1941526	*PIK3C3*	rs1944967	*PIK3C3*	rs12031854	*PIK3C2B*
rs1941526	*PIK3C3*	rs1944967	*PIK3C3*	rs6507451	*PIK3C3*	rs3747633	*PIK3C2B*
rs1944967	*PIK3C3*	rs6507451	*PIK3C3*	rs682408	*PIK3C3*	rs3106366	*PIK3C2B*
rs3764459	*PIK3C3*	rs682408	*PIK3C3*	rs7238178	*PIK3C3*	rs2271427	*PIK3C2B*
rs6507451	*PIK3C3*	rs7238178	*PIK3C3*	rs9956832	*PIK3C3*	rs3014637	*PIK3C2B*
rs682408	*PIK3C3*	rs9956832	*PIK3C3*	rs9958749	*PIK3C3*	rs2137255	*PIK3C2B*
rs7238178	*PIK3C3*	rs9958749	*PIK3C3*	rs13082485	*PIK3CA*	rs2999484	*PIK3C2B*
rs9956832	*PIK3C3*	rs13082485	*PIK3CA*	rs1607237	*PIK3CA*	rs2271415	*PIK3C2B*
rs13082485	*PIK3CA*	rs1607237	*PIK3CA*	rs2677760	*PIK3CA*	rs6594014	*PIK3C2B*
rs13320527	*PIK3CA*	rs2677760	*PIK3CA*	rs2699905	*PIK3CA*	rs11240748	*PIK3C2B*
rs1607237	*PIK3CA*	rs2699905	*PIK3CA*	rs6443624	*PIK3CA*	rs7556371	*PIK3C2B*
rs2677760	*PIK3CA*	rs6443624	*PIK3CA*	rs6807293	*PIK3CA*	rs7556371	*PIK3C2B*
rs2699905	*PIK3CA*	rs6807293	*PIK3CA*	rs7614305	*PIK3CA*	rs10770333	*PIK3C2G*
rs6443624	*PIK3CA*	rs7614305	*PIK3CA*	rs7621329	*PIK3CA*	rs4369463	*PIK3C2G*
rs6807293	*PIK3CA*	rs7621329	*PIK3CA*	rs7641889	*PIK3CA*	rs4385947	*PIK3C2G*
rs7614305	*PIK3CA*	rs7641889	*PIK3CA*	rs7646409	*PIK3CA*	rs17409120	*PIK3C2G*
rs7621329	*PIK3CA*	rs7646409	*PIK3CA*	rs9878820	*PIK3CB*	rs11044004	*PIK3C2G*
rs7641889	*PIK3CA*	rs9878820	*PIK3CB*	rs12075554	*PIK3CD*	rs7133666	*PIK3C2G*
rs7646409	*PIK3CA*	rs12075554	*PIK3CD*	rs12568084	*PIK3CD*	rs4534639	*PIK3C2G*
rs10513055	*PIK3CB*	rs12568084	*PIK3CD*	rs12569008	*PIK3CD*	rs12582971	*PIK3C2G*
rs361059	*PIK3CB*	rs12569008	*PIK3CD*	rs4129341	*PIK3CD*	rs10505811	*PIK3C2G*
rs497900	*PIK3CB*	rs4129341	*PIK3CD*	rs4240896	*PIK3CD*	rs9300118	*PIK3C2G*
rs500687	*PIK3CB*	rs4240896	*PIK3CD*	rs4240910	*PIK3CD*	rs10160860	*PIK3C2G*
rs531577	*PIK3CB*	rs4240910	*PIK3CD*	rs6540985	*PIK3CD*	rs4609650	*PIK3C2G*
rs558905	*PIK3CB*	rs6540985	*PIK3CD*	rs6541017	*PIK3CD*	rs11044026	*PIK3C2G*
rs693293	*PIK3CB*	rs6541017	*PIK3CD*	rs9430506	*PIK3CD*	rs10505810	*PIK3C2G*
rs9878820	*PIK3CB*	rs9430506	*PIK3CD*	rs10215499	*PIK3CG*	rs12309567	*PIK3C2G*
rs12075554	*PIK3CD*	rs10215499	*PIK3CG*	rs1526083	*PIK3CG*	rs10841019	*PIK3C2G*
rs12568084	*PIK3CD*	rs1526083	*PIK3CG*	rs17153527	*PIK3CG*	rs12297325	*PIK3C2G*
rs4129341	*PIK3CD*	rs17153527	*PIK3CG*	rs1724262	*PIK3CG*	rs4595599	*PIK3C2G*
rs4240896	*PIK3CD*	rs193740	*PIK3CG*	rs193740	*PIK3CG*	rs12422650	*PIK3C2G*
rs4240910	*PIK3CD*	rs4727666	*PIK3CG*	rs4727666	*PIK3CG*	rs11044058	*PIK3C2G*
rs6540985	*PIK3CD*	rs4730205	*PIK3CG*	rs4730205	*PIK3CG*	rs11044070	*PIK3C2G*
rs6541017	*PIK3CD*	rs6956373	*PIK3CG*	rs6956373	*PIK3CG*	rs12227441	*PIK3C2G*
rs9430506	*PIK3CD*	rs757902	*PIK3CG*	rs757902	*PIK3CG*	rs11044075	*PIK3C2G*
rs1526083	*PIK3CG*	rs757903	*PIK3CG*	rs757903	*PIK3CG*	rs11044082	*PIK3C2G*
rs1636808	*PIK3CG*	rs849375	*PIK3CG*	rs849375	*PIK3CG*	rs12822135	*PIK3C2G*
rs17153527	*PIK3CG*	rs849376	*PIK3CG*	rs849376	*PIK3CG*	rs11044084	*PIK3C2G*
rs1724262	*PIK3CG*	rs849380	*PIK3CG*	rs849380	*PIK3CG*	rs10841023	*PIK3C2G*

rs193740	*PIK3CG*	rs849412	*PIK3CG*	rs849412	*PIK3CG*	rs10770359	*PIK3C2G*
rs4727666	*PIK3CG*	rs10189031	*PIKFYVE*	rs10189031	*PIKFYVE*	rs12581163	*PIK3C2G*
rs4730205	*PIK3CG*	rs10208191	*PIKFYVE*	rs10190458	*PIKFYVE*	rs10841025	*PIK3C2G*
rs6956373	*PIK3CG*	rs1465804	*PIKFYVE*	rs10208191	*PIKFYVE*	rs11044103	*PIK3C2G*
rs757902	*PIK3CG*	rs1584200	*PIKFYVE*	rs10932260	*PIKFYVE*	rs3813896	*PIK3C2G*
rs757903	*PIK3CG*	rs16841092	*PIKFYVE*	rs1465804	*PIKFYVE*	rs12427286	*PIK3C2G*
rs849367	*PIK3CG*	rs17652774	*PIKFYVE*	rs1584200	*PIKFYVE*	rs9634063	*PIK3C2G*
rs849375	*PIK3CG*	rs2118295	*PIKFYVE*	rs16841092	*PIKFYVE*	rs7308591	*PIK3C2G*
rs849376	*PIK3CG*	rs2118297	*PIKFYVE*	rs17652774	*PIKFYVE*	rs1447406	*PIK3C2G*
rs849380	*PIK3CG*	rs2289171	*PIKFYVE*	rs2118295	*PIKFYVE*	rs12371624	*PIK3C2G*
rs10177810	*PIKFYVE*	rs3769521	*PIKFYVE*	rs2118297	*PIKFYVE*	rs7314398	*PIK3C2G*
rs10189031	*PIKFYVE*	rs4673402	*PIKFYVE*	rs2289171	*PIKFYVE*	rs1868064	*PIK3C2G*
rs10190458	*PIKFYVE*	rs4675754	*PIKFYVE*	rs3769521	*PIKFYVE*	rs1447408	*PIK3C2G*
rs13407268	*PIKFYVE*	rs4675764	*PIKFYVE*	rs4673402	*PIKFYVE*	rs10743273	*PIK3C2G*
rs1584200	*PIKFYVE*	rs6435450	*PIKFYVE*	rs4675754	*PIKFYVE*	rs1374670	*PIK3C2G*
rs17652774	*PIKFYVE*	rs6435453	*PIKFYVE*	rs4675764	*PIKFYVE*	rs719568	*PIK3C2G*
rs1866046	*PIKFYVE*	rs6746926	*PIKFYVE*	rs6435450	*PIKFYVE*	rs12312266	*PIK3C2G*
rs2289171	*PIKFYVE*	rs7569723	*PIKFYVE*	rs6435453	*PIKFYVE*	rs2290044	*PIK3C2G*
rs3769521	*PIKFYVE*	rs9646839	*PIKFYVE*	rs6746926	*PIKFYVE*	rs17411508	*PIK3C2G*
rs4673402	*PIKFYVE*	rs994696	*PIKFYVE*	rs7569723	*PIKFYVE*	rs2305220	*PIK3C2G*
rs4675764	*PIKFYVE*	rs1062190	*PIP4K2A*	rs9646839	*PIKFYVE*	rs11044155	*PIK3C2G*
rs6746926	*PIKFYVE*	rs10764344	*PIP4K2A*	rs994696	*PIKFYVE*	rs7957373	*PIK3C2G*
rs999890	*PIKFYVE*	rs10828316	*PIP4K2A*	rs1062190	*PIP4K2A*	rs10505824	*PIK3C2G*
rs10159847	*PIP4K2A*	rs10828317	*PIP4K2A*	rs10764344	*PIP4K2A*	rs11044165	*PIK3C2G*
rs10508651	*PIP4K2A*	rs11013053	*PIP4K2A*	rs10828316	*PIP4K2A*	rs7969452	*PIK3C2G*
rs1062190	*PIP4K2A*	rs11013067	*PIP4K2A*	rs10828317	*PIP4K2A*	rs11044171	*PIK3C2G*
rs10764344	*PIP4K2A*	rs11013069	*PIP4K2A*	rs11013053	*PIP4K2A*	rs12367676	*PIK3C2G*
rs10828316	*PIP4K2A*	rs11013095	*PIP4K2A*	rs11013069	*PIP4K2A*	rs17418422	*PIK3C2G*
rs10828317	*PIP4K2A*	rs1171509	*PIP4K2A*	rs11013095	*PIP4K2A*	rs1992838	*PIK3C2G*
rs11013053	*PIP4K2A*	rs12253847	*PIP4K2A*	rs12253847	*PIP4K2A*	rs10841036	*PIK3C2G*
rs11013067	*PIP4K2A*	rs12355895	*PIP4K2A*	rs12355895	*PIP4K2A*	rs10841041	*PIK3C2G*
rs11013069	*PIP4K2A*	rs1326341	*PIP4K2A*	rs1326341	*PIP4K2A*	rs10841043	*PIK3C2G*
rs11013086	*PIP4K2A*	rs1409396	*PIP4K2A*	rs1409396	*PIP4K2A*	rs12827287	*PIK3C2G*
rs11013095	*PIP4K2A*	rs1539628	*PIP4K2A*	rs1539628	*PIP4K2A*	rs12581078	*PIK3C2G*
rs11013103	*PIP4K2A*	rs1627983	*PIP4K2A*	rs1627983	*PIP4K2A*	rs17419409	*PIK3C2G*
rs11592199	*PIP4K2A*	rs16922601	*PIP4K2A*	rs16922601	*PIP4K2A*	rs12821147	*PIK3C2G*
rs11597156	*PIP4K2A*	rs16922629	*PIP4K2A*	rs16922629	*PIP4K2A*	rs12818409	*PIK3C2G*
rs1171509	*PIP4K2A*	rs1750758	*PIP4K2A*	rs1750758	*PIP4K2A*	rs2277328	*PIK3C2G*
rs12098721	*PIP4K2A*	rs1750761	*PIP4K2A*	rs1750761	*PIP4K2A*	rs1816971	*PIK3C2G*
rs12253847	*PIP4K2A*	rs1750770	*PIP4K2A*	rs1750770	*PIP4K2A*	rs7964182	*PIK3C2G*
rs12355895	*PIP4K2A*	rs1750775	*PIP4K2A*	rs1750775	*PIP4K2A*	rs10770372	*PIK3C2G*
rs12357384	*PIP4K2A*	rs1778353	*PIP4K2A*	rs1778353	*PIP4K2A*	rs12825580	*PIK3C2G*
rs12773197	*PIP4K2A*	rs1891877	*PIP4K2A*	rs1891877	*PIP4K2A*	rs10841049	*PIK3C2G*
rs1409396	*PIP4K2A*	rs2559523	*PIP4K2A*	rs2559523	*PIP4K2A*	rs11044223	*PIK3C2G*

rs1539628	*PIP4K2A*	rs2559524	*PIP4K2A*	rs2559524	*PIP4K2A*	rs4764409	*PIK3C2G*
rs1627983	*PIP4K2A*	rs3793753	*PIP4K2A*	rs3793753	*PIP4K2A*	rs666864	*PIK3C2G*
rs16922578	*PIP4K2A*	rs4532931	*PIP4K2A*	rs4532931	*PIP4K2A*	rs621042	*PIK3C2G*
rs1750758	*PIP4K2A*	rs6482235	*PIP4K2A*	rs6482235	*PIP4K2A*	rs4763508	*PIK3C2G*
rs1750761	*PIP4K2A*	rs7071450	*PIP4K2A*	rs7071450	*PIP4K2A*	rs644164	*PIK3C2G*
rs1750770	*PIP4K2A*	rs7078735	*PIP4K2A*	rs7078735	*PIP4K2A*	rs11044232	*PIK3C2G*
rs1750775	*PIP4K2A*	rs7084115	*PIP4K2A*	rs7084115	*PIP4K2A*	rs11044233	*PIK3C2G*
rs1778302	*PIP4K2A*	rs7088318	*PIP4K2A*	rs7088318	*PIP4K2A*	rs518345	*PIK3C2G*
rs1778317	*PIP4K2A*	rs7094131	*PIP4K2A*	rs7094131	*PIP4K2A*	rs531697	*PIK3C2G*
rs1778353	*PIP4K2A*	rs7094187	*PIP4K2A*	rs7094187	*PIP4K2A*	rs4764412	*PIK3C2G*
rs1891877	*PIP4K2A*	rs7899156	*PIP4K2A*	rs7899156	*PIP4K2A*	rs2931484	*PIK3C2G*
rs2559523	*PIP4K2A*	rs7912144	*PIP4K2A*	rs7912144	*PIP4K2A*	rs10734680	*PIK3C2G*
rs2559524	*PIP4K2A*	rs7914338	*PIP4K2A*	rs7914338	*PIP4K2A*	rs578947	*PIK3C2G*
rs2765993	*PIP4K2A*	rs7919839	*PIP4K2A*	rs7919839	*PIP4K2A*	rs982754	*PIK3C2G*
rs3793753	*PIP4K2A*	rs943189	*PIP4K2A*	rs943189	*PIP4K2A*	rs982754	*PIK3C2G*
rs4532931	*PIP4K2A*	rs943196	*PIP4K2A*	rs115327	*PIP4K2B*	rs6507451	*PIK3C3*
rs7071450	*PIP4K2A*	rs115327	*PIP4K2B*	rs11653487	*PIP4K2B*	rs1944967	*PIK3C3*
rs7075433	*PIP4K2A*	rs11653487	*PIP4K2B*	rs2075061	*PIP4K2B*	rs9956832	*PIK3C3*
rs7075499	*PIP4K2A*	rs2075061	*PIP4K2B*	rs228249	*PIP4K2B*	rs682408	*PIK3C3*
rs7084115	*PIP4K2A*	rs228249	*PIP4K2B*	rs228285	*PIP4K2B*	rs12954899	*PIK3C3*
rs7088318	*PIP4K2A*	rs228285	*PIP4K2B*	rs2338115	*PIP4K2B*	rs3764459	*PIK3C3*
rs7093543	*PIP4K2A*	rs2338115	*PIP4K2B*	rs626866	*PIP4K2B*	rs7238178	*PIK3C3*
rs7094131	*PIP4K2A*	rs626866	*PIP4K2B*	rs764190	*PIP4K2B*	rs1941526	*PIK3C3*
rs7899156	*PIP4K2A*	rs764190	*PIP4K2B*	rs11172254	*PIP4K2C*	rs1941526	*PIK3C3*
rs7912144	*PIP4K2A*	rs11172254	*PIP4K2C*	rs775250	*PIP4K2C*	rs6807293	*PIK3CA*
rs7919839	*PIP4K2A*	rs775250	*PIP4K2C*	rs775251	*PIP4K2C*	rs7621329	*PIK3CA*
rs943189	*PIP4K2A*	rs775251	*PIP4K2C*	rs812315	*PIP4K2C*	rs2699905	*PIK3CA*
rs943196	*PIP4K2A*	rs812315	*PIP4K2C*	rs10749660	*PIP5K1A*	rs7641889	*PIK3CA*
rs115327	*PIP4K2B*	rs10749660	*PIP5K1A*	rs4520422	*PIP5K1A*	rs6443624	*PIK3CA*
rs11653487	*PIP4K2B*	rs4520422	*PIP5K1A*	rs4970944	*PIP5K1A*	rs7646409	*PIK3CA*
rs12453325	*PIP4K2B*	rs4970944	*PIP5K1A*	rs7532935	*PIP5K1A*	rs2677760	*PIK3CA*
rs2075061	*PIP4K2B*	rs7532935	*PIP5K1A*	rs10114872	*PIP5K1B*	rs13082485	*PIK3CA*
rs228285	*PIP4K2B*	rs10114872	*PIP5K1B*	rs10117043	*PIP5K1B*	rs7614305	*PIK3CA*
rs228307	*PIP4K2B*	rs10117043	*PIP5K1B*	rs10121993	*PIP5K1B*	rs13320527	*PIK3CA*
rs2338115	*PIP4K2B*	rs10121993	*PIP5K1B*	rs1014237	*PIP5K1B*	rs1607237	*PIK3CA*
rs764190	*PIP4K2B*	rs1014237	*PIP5K1B*	rs1014807	*PIP5K1B*	rs1607237	*PIK3CA*
rs11172254	*PIP4K2C*	rs1014807	*PIP5K1B*	rs10511963	*PIP5K1B*	rs9878820	*PIK3CB*
rs775250	*PIP4K2C*	rs10511963	*PIP5K1B*	rs10735624	*PIP5K1B*	rs531577	*PIK3CB*
rs775251	*PIP4K2C*	rs10735624	*PIP5K1B*	rs10735625	*PIP5K1B*	rs693293	*PIK3CB*
rs812315	*PIP4K2C*	rs10735625	*PIP5K1B*	rs10746974	*PIP5K1B*	rs497900	*PIK3CB*
rs4520422	*PIP5K1A*	rs10746974	*PIP5K1B*	rs10746983	*PIP5K1B*	rs361059	*PIK3CB*
rs4970944	*PIP5K1A*	rs10746983	*PIP5K1B*	rs10781117	*PIP5K1B*	rs10513055	*PIK3CB*
rs7532935	*PIP5K1A*	rs10781117	*PIP5K1B*	rs10781329	*PIP5K1B*	rs558905	*PIK3CB*
rs10114872	*PIP5K1B*	rs10781329	*PIP5K1B*	rs10869335	*PIP5K1B*	rs500687	*PIK3CB*

rs10117043	*PIP5K1B*	rs10869335	*PIP5K1B*	rs10869396	*PIP5K1B*	rs361068	*PIK3CB*
rs10121993	*PIP5K1B*	rs10869396	*PIP5K1B*	rs10869400	*PIP5K1B*	rs361068	*PIK3CB*
rs10125301	*PIP5K1B*	rs10869400	*PIP5K1B*	rs10869420	*PIP5K1B*	rs9430506	*PIK3CD*
rs1014237	*PIP5K1B*	rs10869420	*PIP5K1B*	rs10869538	*PIP5K1B*	rs4129341	*PIK3CD*
rs1014807	*PIP5K1B*	rs10869538	*PIP5K1B*	rs10869686	*PIP5K1B*	rs6540985	*PIK3CD*
rs10511963	*PIP5K1B*	rs10869686	*PIP5K1B*	rs1107108	*PIP5K1B*	rs4240896	*PIK3CD*
rs10735625	*PIP5K1B*	rs1107108	*PIP5K1B*	rs11143417	*PIP5K1B*	rs4240910	*PIK3CD*
rs10746974	*PIP5K1B*	rs11143417	*PIP5K1B*	rs11143833	*PIP5K1B*	rs12075554	*PIK3CD*
rs10746983	*PIP5K1B*	rs11143833	*PIP5K1B*	rs11144133	*PIP5K1B*	rs6541017	*PIK3CD*
rs10781117	*PIP5K1B*	rs11144133	*PIP5K1B*	rs12339235	*PIP5K1B*	rs12569008	*PIK3CD*
rs10781306	*PIP5K1B*	rs12339235	*PIP5K1B*	rs12349586	*PIP5K1B*	rs12569008	*PIK3CD*
rs10781329	*PIP5K1B*	rs12349586	*PIP5K1B*	rs12378665	*PIP5K1B*	rs193740	*PIK3CG*
rs10869335	*PIP5K1B*	rs12378665	*PIP5K1B*	rs12686355	*PIP5K1B*	rs1724262	*PIK3CG*
rs10869396	*PIP5K1B*	rs12686355	*PIP5K1B*	rs12686693	*PIP5K1B*	rs1636808	*PIK3CG*
rs10869400	*PIP5K1B*	rs12686693	*PIP5K1B*	rs13296679	*PIP5K1B*	rs849375	*PIK3CG*
rs10869420	*PIP5K1B*	rs13296679	*PIP5K1B*	rs1412988	*PIP5K1B*	rs849376	*PIK3CG*
rs10869538	*PIP5K1B*	rs1412988	*PIP5K1B*	rs1412990	*PIP5K1B*	rs17153527	*PIK3CG*
rs10869686	*PIP5K1B*	rs1412990	*PIP5K1B*	rs1541084	*PIP5K1B*	rs849380	*PIK3CG*
rs1107108	*PIP5K1B*	rs1541084	*PIP5K1B*	rs1556751	*PIP5K1B*	rs757902	*PIK3CG*
rs11143417	*PIP5K1B*	rs1556751	*PIP5K1B*	rs17058792	*PIP5K1B*	rs757903	*PIK3CG*
rs11144027	*PIP5K1B*	rs17058792	*PIP5K1B*	rs17058884	*PIP5K1B*	rs6956373	*PIK3CG*
rs11144133	*PIP5K1B*	rs17058884	*PIP5K1B*	rs17391840	*PIP5K1B*	rs4727666	*PIK3CG*
rs12339235	*PIP5K1B*	rs17391840	*PIP5K1B*	rs17392931	*PIP5K1B*	rs1526083	*PIK3CG*
rs12349586	*PIP5K1B*	rs17392931	*PIP5K1B*	rs1889150	*PIP5K1B*	rs849367	*PIK3CG*
rs12380573	*PIP5K1B*	rs1889150	*PIP5K1B*	rs2039624	*PIP5K1B*	rs4730205	*PIK3CG*
rs12686355	*PIP5K1B*	rs2039624	*PIP5K1B*	rs2151418	*PIP5K1B*	rs11766675	*PIK3CG*
rs12686693	*PIP5K1B*	rs2151418	*PIP5K1B*	rs2152649	*PIP5K1B*	rs849412	*PIK3CG*
rs13296679	*PIP5K1B*	rs2152649	*PIP5K1B*	rs2871223	*PIP5K1B*	rs849412	*PIK3CG*
rs1412988	*PIP5K1B*	rs2871223	*PIP5K1B*	rs3812537	*PIP5K1B*	rs3769521	*PIKFYVE*
rs1412990	*PIP5K1B*	rs3812537	*PIP5K1B*	rs3829061	*PIP5K1B*	rs17652774	*PIKFYVE*
rs1414954	*PIP5K1B*	rs3829061	*PIP5K1B*	rs4237270	*PIP5K1B*	rs6746926	*PIKFYVE*
rs1541084	*PIP5K1B*	rs4237270	*PIP5K1B*	rs4745231	*PIP5K1B*	rs1584200	*PIKFYVE*
rs1556751	*PIP5K1B*	rs4745231	*PIP5K1B*	rs4745296	*PIP5K1B*	rs10189031	*PIKFYVE*
rs17058792	*PIP5K1B*	rs4745296	*PIP5K1B*	rs4745321	*PIP5K1B*	rs999890	*PIKFYVE*
rs17058884	*PIP5K1B*	rs4745321	*PIP5K1B*	rs4745402	*PIP5K1B*	rs1866046	*PIKFYVE*
rs17391840	*PIP5K1B*	rs4745402	*PIP5K1B*	rs4745451	*PIP5K1B*	rs2289171	*PIKFYVE*
rs17392931	*PIP5K1B*	rs4745451	*PIP5K1B*	rs4745466	*PIP5K1B*	rs10190458	*PIKFYVE*
rs1889150	*PIP5K1B*	rs4745466	*PIP5K1B*	rs4745514	*PIP5K1B*	rs13407268	*PIKFYVE*
rs2151414	*PIP5K1B*	rs4745514	*PIP5K1B*	rs4745520	*PIP5K1B*	rs10177810	*PIKFYVE*
rs2151418	*PIP5K1B*	rs4745520	*PIP5K1B*	rs6560397	*PIP5K1B*	rs4673402	*PIKFYVE*
rs2152649	*PIP5K1B*	rs6560397	*PIP5K1B*	rs6560444	*PIP5K1B*	rs4675764	*PIKFYVE*
rs2871223	*PIP5K1B*	rs6560444	*PIP5K1B*	rs7041004	*PIP5K1B*	rs12355895	*PIP4K2A*
rs3812537	*PIP5K1B*	rs7041004	*PIP5K1B*	rs7044422	*PIP5K1B*	rs7899156	*PIP4K2A*
rs4237270	*PIP5K1B*	rs7044422	*PIP5K1B*	rs7048825	*PIP5K1B*	rs4532931	*PIP4K2A*

rs4745231	*PIP5K1B*	rs7048825	*PIP5K1B*	rs7849727	*PIP5K1B*	rs10828316	*PIP4K2A*
rs4745296	*PIP5K1B*	rs7849727	*PIP5K1B*	rs7855908	*PIP5K1B*	rs10828317	*PIP4K2A*
rs4745321	*PIP5K1B*	rs7855908	*PIP5K1B*	rs7859638	*PIP5K1B*	rs943189	*PIP4K2A*
rs4745375	*PIP5K1B*	rs7859638	*PIP5K1B*	rs7866673	*PIP5K1B*	rs7094131	*PIP4K2A*
rs4745402	*PIP5K1B*	rs7866673	*PIP5K1B*	rs7870383	*PIP5K1B*	rs7088318	*PIP4K2A*
rs4745451	*PIP5K1B*	rs7870383	*PIP5K1B*	rs8114	*PIP5K1B*	rs3793753	*PIP4K2A*
rs4745466	*PIP5K1B*	rs8114	*PIP5K1B*	rs872077	*PIP5K1B*	rs12253847	*PIP4K2A*
rs6560397	*PIP5K1B*	rs872077	*PIP5K1B*	rs883952	*PIP5K1B*	rs7919839	*PIP4K2A*
rs6560444	*PIP5K1B*	rs883952	*PIP5K1B*	rs9314841	*PIP5K1B*	rs1062190	*PIP4K2A*
rs7041004	*PIP5K1B*	rs9314841	*PIP5K1B*	rs963707	*PIP5K1B*	rs11013053	*PIP4K2A*
rs7044422	*PIP5K1B*	rs963707	*PIP5K1B*	rs11672559	*PIP5K1C*	rs7071450	*PIP4K2A*
rs7048825	*PIP5K1B*	rs11672559	*PIP5K1C*	rs2270083	*PIP5K1C*	rs7075433	*PIP4K2A*
rs7859638	*PIP5K1B*	rs2270083	*PIP5K1C*	rs2271875	*PIP5K1C*	rs12357384	*PIP4K2A*
rs7866673	*PIP5K1B*	rs2271875	*PIP5K1C*	rs2286435	*PIP5K1C*	rs7912144	*PIP4K2A*
rs8114	*PIP5K1B*	rs2286435	*PIP5K1C*	rs3746124	*PIP5K1C*	rs1409396	*PIP4K2A*
rs883751	*PIP5K1B*	rs3746124	*PIP5K1C*	rs4807492	*PIP5K1C*	rs10764344	*PIP4K2A*
rs883952	*PIP5K1B*	rs4807492	*PIP5K1C*	rs4807493	*PIP5K1C*	rs12773197	*PIP4K2A*
rs11672559	*PIP5K1C*	rs4807493	*PIP5K1C*	rs740873	*PIP5K1C*	rs2765993	*PIP4K2A*
rs1476592	*PIP5K1C*	rs740873	*PIP5K1C*	rs757454	*PIP5K1C*	rs2559524	*PIP4K2A*
rs2270083	*PIP5K1C*	rs757454	*PIP5K1C*	rs3739821	*PIP5KL1*	rs7093543	*PIP4K2A*
rs2271875	*PIP5K1C*	rs3739821	*PIP5KL1*	rs4836593	*PIP5KL1*	rs2559523	*PIP4K2A*
rs4807493	*PIP5K1C*	rs4836593	*PIP5KL1*	rs6478803	*PIP5KL1*	rs1778353	*PIP4K2A*
rs740873	*PIP5K1C*	rs6478803	*PIP5KL1*	rs6781	*PIP5KL1*	rs11013067	*PIP4K2A*
rs757454	*PIP5K1C*	rs6781	*PIP5KL1*	rs7859	*PIP5KL1*	rs1750758	*PIP4K2A*
rs8109485	*PIP5K1C*	rs7859	*PIP5KL1*	rs1018443	*PLCB1*	rs1778302	*PIP4K2A*
rs3739821	*PIP5KL1*	rs1018443	*PLCB1*	rs1033566	*PLCB1*	rs11013069	*PIP4K2A*
rs6478803	*PIP5KL1*	rs1033566	*PLCB1*	rs1033684	*PLCB1*	rs1750761	*PIP4K2A*
rs7859	*PIP5KL1*	rs1033684	*PLCB1*	rs1040496	*PLCB1*	rs1627983	*PIP4K2A*
rs1005987	*PLCB1*	rs1040496	*PLCB1*	rs1047383	*PLCB1*	rs1778317	*PIP4K2A*
rs1015170	*PLCB1*	rs1047383	*PLCB1*	rs10485723	*PLCB1*	rs1750770	*PIP4K2A*
rs1018443	*PLCB1*	rs10485723	*PLCB1*	rs10485724	*PLCB1*	rs1539628	*PIP4K2A*
rs1033566	*PLCB1*	rs10485724	*PLCB1*	rs10485728	*PLCB1*	rs943196	*PIP4K2A*
rs1033684	*PLCB1*	rs10485728	*PLCB1*	rs11087808	*PLCB1*	rs1750775	*PIP4K2A*
rs1040496	*PLCB1*	rs11087808	*PLCB1*	rs11698656	*PLCB1*	rs7075499	*PIP4K2A*
rs1047383	*PLCB1*	rs11698656	*PLCB1*	rs11906514	*PLCB1*	rs12098721	*PIP4K2A*
rs10485722	*PLCB1*	rs11906514	*PLCB1*	rs12053642	*PLCB1*	rs10508651	*PIP4K2A*
rs10485723	*PLCB1*	rs12053642	*PLCB1*	rs1232779	*PLCB1*	rs11013086	*PIP4K2A*
rs10485724	*PLCB1*	rs1232779	*PLCB1*	rs1232782	*PLCB1*	rs1171509	*PIP4K2A*
rs10485728	*PLCB1*	rs1232782	*PLCB1*	rs1232783	*PLCB1*	rs16922578	*PIP4K2A*
rs11087808	*PLCB1*	rs1232783	*PLCB1*	rs1237829	*PLCB1*	rs10159847	*PIP4K2A*
rs11698656	*PLCB1*	rs1237829	*PLCB1*	rs1238232	*PLCB1*	rs11597156	*PIP4K2A*
rs11906514	*PLCB1*	rs1238232	*PLCB1*	rs12480099	*PLCB1*	rs11013095	*PIP4K2A*
rs12053642	*PLCB1*	rs12480099	*PLCB1*	rs12624339	*PLCB1*	rs1891877	*PIP4K2A*
rs1232779	*PLCB1*	rs12624339	*PLCB1*	rs13040221	*PLCB1*	rs11592199	*PIP4K2A*

rs1232782	*PLCB1*	rs13040221	*PLCB1*	rs1342585	*PLCB1*	rs11013103	*PIP4K2A*
rs1232783	*PLCB1*	rs1342585	*PLCB1*	rs1474581	*PLCB1*	rs7084115	*PIP4K2A*
rs1237829	*PLCB1*	rs1474581	*PLCB1*	rs1474683	*PLCB1*	rs7094187	*PIP4K2A*
rs1238232	*PLCB1*	rs1474683	*PLCB1*	rs1474937	*PLCB1*	rs6482235	*PIP4K2A*
rs12480099	*PLCB1*	rs1474937	*PLCB1*	rs1534897	*PLCB1*	rs6482235	*PIP4K2A*
rs12624339	*PLCB1*	rs1534897	*PLCB1*	rs1534968	*PLCB1*	rs764190	*PIP4K2B*
rs13037679	*PLCB1*	rs1534968	*PLCB1*	rs1555212	*PLCB1*	rs228285	*PIP4K2B*
rs13040221	*PLCB1*	rs1555212	*PLCB1*	rs1569604	*PLCB1*	rs2338115	*PIP4K2B*
rs1342585	*PLCB1*	rs1569604	*PLCB1*	rs16995111	*PLCB1*	rs2075061	*PIP4K2B*
rs1474581	*PLCB1*	rs16995111	*PLCB1*	rs16995121	*PLCB1*	rs12453325	*PIP4K2B*
rs1474683	*PLCB1*	rs16995121	*PLCB1*	rs17446308	*PLCB1*	rs11653487	*PIP4K2B*
rs1474937	*PLCB1*	rs17446308	*PLCB1*	rs17446441	*PLCB1*	rs228307	*PIP4K2B*
rs1534897	*PLCB1*	rs17446441	*PLCB1*	rs1883503	*PLCB1*	rs115327	*PIP4K2B*
rs1534968	*PLCB1*	rs1883503	*PLCB1*	rs1967681	*PLCB1*	rs228249	*PIP4K2B*
rs1555212	*PLCB1*	rs1967681	*PLCB1*	rs2050090	*PLCB1*	rs626866	*PIP4K2B*
rs1569604	*PLCB1*	rs2050090	*PLCB1*	rs2064272	*PLCB1*	rs626866	*PIP4K2B*
rs1605791	*PLCB1*	rs2064272	*PLCB1*	rs2076409	*PLCB1*	rs11172254	*PIP4K2C*
rs16995121	*PLCB1*	rs2076409	*PLCB1*	rs2076685	*PLCB1*	rs775250	*PIP4K2C*
rs17347805	*PLCB1*	rs2076685	*PLCB1*	rs2103653	*PLCB1*	rs775251	*PIP4K2C*
rs17362299	*PLCB1*	rs2103653	*PLCB1*	rs2143205	*PLCB1*	rs812315	*PIP4K2C*
rs17431073	*PLCB1*	rs2143205	*PLCB1*	rs2143266	*PLCB1*	rs812315	*PIP4K2C*
rs17436253	*PLCB1*	rs2143266	*PLCB1*	rs2179138	*PLCB1*	rs4970944	*PIP5K1A*
rs17446308	*PLCB1*	rs2179138	*PLCB1*	rs2179478	*PLCB1*	rs10749660	*PIP5K1A*
rs17446441	*PLCB1*	rs2179478	*PLCB1*	rs2179984	*PLCB1*	rs4520422	*PIP5K1A*
rs1883503	*PLCB1*	rs2179984	*PLCB1*	rs2206422	*PLCB1*	rs7532935	*PIP5K1A*
rs1935671	*PLCB1*	rs2206422	*PLCB1*	rs2206423	*PLCB1*	rs7532935	*PIP5K1A*
rs1967681	*PLCB1*	rs2206423	*PLCB1*	rs2206489	*PLCB1*	rs1014237	*PIP5K1B*
rs2050090	*PLCB1*	rs2206489	*PLCB1*	rs2221695	*PLCB1*	rs1014807	*PIP5K1B*
rs2064272	*PLCB1*	rs2221695	*PLCB1*	rs2223538	*PLCB1*	rs11143417	*PIP5K1B*
rs2076409	*PLCB1*	rs2223538	*PLCB1*	rs2223837	*PLCB1*	rs1412990	*PIP5K1B*
rs2076685	*PLCB1*	rs2223837	*PLCB1*	rs227130	*PLCB1*	rs1107108	*PIP5K1B*
rs2103653	*PLCB1*	rs227130	*PLCB1*	rs227133	*PLCB1*	rs3812537	*PIP5K1B*
rs2143205	*PLCB1*	rs227133	*PLCB1*	rs227134	*PLCB1*	rs2151418	*PIP5K1B*
rs2143266	*PLCB1*	rs227134	*PLCB1*	rs2294259	*PLCB1*	rs10781117	*PIP5K1B*
rs2179138	*PLCB1*	rs2294259	*PLCB1*	rs2294597	*PLCB1*	rs10121993	*PIP5K1B*
rs2179440	*PLCB1*	rs2294597	*PLCB1*	rs2295179	*PLCB1*	rs10125301	*PIP5K1B*
rs2179478	*PLCB1*	rs2295179	*PLCB1*	rs2327046	*PLCB1*	rs4745231	*PIP5K1B*
rs2179984	*PLCB1*	rs2327046	*PLCB1*	rs2327070	*PLCB1*	rs13296679	*PIP5K1B*
rs2206423	*PLCB1*	rs2327070	*PLCB1*	rs2423360	*PLCB1*	rs7044422	*PIP5K1B*
rs2206489	*PLCB1*	rs2423360	*PLCB1*	rs2423364	*PLCB1*	rs963707	*PIP5K1B*
rs2221695	*PLCB1*	rs2423364	*PLCB1*	rs2423366	*PLCB1*	rs7866673	*PIP5K1B*
rs2223538	*PLCB1*	rs2423366	*PLCB1*	rs2662999	*PLCB1*	rs12380573	*PIP5K1B*
rs2223837	*PLCB1*	rs2662999	*PLCB1*	rs2719774	*PLCB1*	rs12686355	*PIP5K1B*
rs227130	*PLCB1*	rs2719774	*PLCB1*	rs2719776	*PLCB1*	rs10735625	*PIP5K1B*

rs227133	*PLCB1*	rs2719776	*PLCB1*	rs2719795	*PLCB1*	rs2151414	*PIP5K1B*
rs227134	*PLCB1*	rs2719795	*PLCB1*	rs2719804	*PLCB1*	rs8114	*PIP5K1B*
rs227142	*PLCB1*	rs2719804	*PLCB1*	rs2719807	*PLCB1*	rs1412988	*PIP5K1B*
rs2294259	*PLCB1*	rs2719807	*PLCB1*	rs2745755	*PLCB1*	rs12339235	*PIP5K1B*
rs2294597	*PLCB1*	rs2745755	*PLCB1*	rs2745772	*PLCB1*	rs10511963	*PIP5K1B*
rs2295179	*PLCB1*	rs2745772	*PLCB1*	rs2745776	*PLCB1*	rs10869335	*PIP5K1B*
rs2327025	*PLCB1*	rs2745776	*PLCB1*	rs2876140	*PLCB1*	rs1556751	*PIP5K1B*
rs2327046	*PLCB1*	rs2876140	*PLCB1*	rs2876145	*PLCB1*	rs4745296	*PIP5K1B*
rs2327070	*PLCB1*	rs2876145	*PLCB1*	rs3817881	*PLCB1*	rs17391840	*PIP5K1B*
rs2423354	*PLCB1*	rs3817881	*PLCB1*	rs3848835	*PLCB1*	rs1889150	*PIP5K1B*
rs2423356	*PLCB1*	rs3848835	*PLCB1*	rs3891453	*PLCB1*	rs10869396	*PIP5K1B*
rs2423360	*PLCB1*	rs3891453	*PLCB1*	rs3902336	*PLCB1*	rs4745321	*PIP5K1B*
rs2423361	*PLCB1*	rs3902336	*PLCB1*	rs4083408	*PLCB1*	rs10869400	*PIP5K1B*
rs2423363	*PLCB1*	rs4083408	*PLCB1*	rs4142365	*PLCB1*	rs10869420	*PIP5K1B*
rs2423364	*PLCB1*	rs4142365	*PLCB1*	rs4399790	*PLCB1*	rs7041004	*PIP5K1B*
rs2423366	*PLCB1*	rs4399790	*PLCB1*	rs4419296	*PLCB1*	rs6560397	*PIP5K1B*
rs2662985	*PLCB1*	rs4419296	*PLCB1*	rs4432538	*PLCB1*	rs11144027	*PIP5K1B*
rs2662999	*PLCB1*	rs4432538	*PLCB1*	rs4471960	*PLCB1*	rs17392931	*PIP5K1B*
rs2719774	*PLCB1*	rs4471960	*PLCB1*	rs4496390	*PLCB1*	rs11144133	*PIP5K1B*
rs2719776	*PLCB1*	rs4496390	*PLCB1*	rs4813853	*PLCB1*	rs4237270	*PIP5K1B*
rs2719795	*PLCB1*	rs4813853	*PLCB1*	rs4813865	*PLCB1*	rs4745375	*PIP5K1B*
rs2719804	*PLCB1*	rs4813865	*PLCB1*	rs4816047	*PLCB1*	rs883952	*PIP5K1B*
rs2719807	*PLCB1*	rs4816047	*PLCB1*	rs4816085	*PLCB1*	rs17058792	*PIP5K1B*
rs2745755	*PLCB1*	rs4816085	*PLCB1*	rs4816089	*PLCB1*	rs12686693	*PIP5K1B*
rs2745756	*PLCB1*	rs4816089	*PLCB1*	rs4816090	*PLCB1*	rs17058884	*PIP5K1B*
rs2745772	*PLCB1*	rs4816090	*PLCB1*	rs6039040	*PLCB1*	rs1414954	*PIP5K1B*
rs2745776	*PLCB1*	rs6039040	*PLCB1*	rs6039049	*PLCB1*	rs7859638	*PIP5K1B*
rs2745787	*PLCB1*	rs6039049	*PLCB1*	rs6039104	*PLCB1*	rs2152649	*PIP5K1B*
rs2876140	*PLCB1*	rs6039104	*PLCB1*	rs6039109	*PLCB1*	rs10869538	*PIP5K1B*
rs2876145	*PLCB1*	rs6039109	*PLCB1*	rs6039189	*PLCB1*	rs6560444	*PIP5K1B*
rs3817881	*PLCB1*	rs6039189	*PLCB1*	rs6039190	*PLCB1*	rs4745402	*PIP5K1B*
rs3848835	*PLCB1*	rs6039190	*PLCB1*	rs6039191	*PLCB1*	rs10746974	*PIP5K1B*
rs3902336	*PLCB1*	rs6039191	*PLCB1*	rs6039206	*PLCB1*	rs10114872	*PIP5K1B*
rs4083408	*PLCB1*	rs6039206	*PLCB1*	rs6039211	*PLCB1*	rs12349586	*PIP5K1B*
rs4142365	*PLCB1*	rs6039211	*PLCB1*	rs6039237	*PLCB1*	rs1541084	*PIP5K1B*
rs4399790	*PLCB1*	rs6039237	*PLCB1*	rs6039268	*PLCB1*	rs10117043	*PIP5K1B*
rs4419296	*PLCB1*	rs6039268	*PLCB1*	rs6039298	*PLCB1*	rs10781306	*PIP5K1B*
rs4432538	*PLCB1*	rs6039298	*PLCB1*	rs6039307	*PLCB1*	rs7048825	*PIP5K1B*
rs4471960	*PLCB1*	rs6039307	*PLCB1*	rs6039312	*PLCB1*	rs4745451	*PIP5K1B*
rs4496390	*PLCB1*	rs6039312	*PLCB1*	rs6039319	*PLCB1*	rs12378665	*PIP5K1B*
rs4813853	*PLCB1*	rs6039319	*PLCB1*	rs6055550	*PLCB1*	rs4745466	*PIP5K1B*
rs4813854	*PLCB1*	rs6055550	*PLCB1*	rs6055562	*PLCB1*	rs10746983	*PIP5K1B*
rs4813863	*PLCB1*	rs6055562	*PLCB1*	rs6055578	*PLCB1*	rs883751	*PIP5K1B*
rs4813865	*PLCB1*	rs6055578	*PLCB1*	rs6055594	*PLCB1*	rs2871223	*PIP5K1B*

rs4816047	*PLCB1*	rs6055594	*PLCB1*	rs6055601	*PLCB1*	rs10781329	*PIP5K1B*
rs4816085	*PLCB1*	rs6055601	*PLCB1*	rs6055603	*PLCB1*	rs10869686	*PIP5K1B*
rs4816089	*PLCB1*	rs6055603	*PLCB1*	rs6055625	*PLCB1*	rs872077	*PIP5K1B*
rs4816090	*PLCB1*	rs6055625	*PLCB1*	rs6055652	*PLCB1*	rs4745514	*PIP5K1B*
rs6039040	*PLCB1*	rs6055652	*PLCB1*	rs6055685	*PLCB1*	rs4745520	*PIP5K1B*
rs6039049	*PLCB1*	rs6055685	*PLCB1*	rs6055889	*PLCB1*	rs9314841	*PIP5K1B*
rs6039104	*PLCB1*	rs6055889	*PLCB1*	rs6055910	*PLCB1*	rs9314841	*PIP5K1B*
rs6039107	*PLCB1*	rs6055910	*PLCB1*	rs6055912	*PLCB1*	rs4807492	*PIP5K1C*
rs6039109	*PLCB1*	rs6055912	*PLCB1*	rs6055922	*PLCB1*	rs2271875	*PIP5K1C*
rs6039189	*PLCB1*	rs6055922	*PLCB1*	rs6055923	*PLCB1*	rs4807493	*PIP5K1C*
rs6039190	*PLCB1*	rs6055923	*PLCB1*	rs6055926	*PLCB1*	rs2270083	*PIP5K1C*
rs6039191	*PLCB1*	rs6055926	*PLCB1*	rs6055927	*PLCB1*	rs757454	*PIP5K1C*
rs6039206	*PLCB1*	rs6055927	*PLCB1*	rs6055928	*PLCB1*	rs8109485	*PIP5K1C*
rs6039211	*PLCB1*	rs6055928	*PLCB1*	rs6055944	*PLCB1*	rs11672559	*PIP5K1C*
rs6039237	*PLCB1*	rs6055944	*PLCB1*	rs6055995	*PLCB1*	rs1476592	*PIP5K1C*
rs6039268	*PLCB1*	rs6055995	*PLCB1*	rs6056006	*PLCB1*	rs740873	*PIP5K1C*
rs6039298	*PLCB1*	rs6056006	*PLCB1*	rs6056080	*PLCB1*	rs740873	*PIP5K1C*
rs6039307	*PLCB1*	rs6056080	*PLCB1*	rs6056111	*PLCB1*	rs6781	*PIP5KL1*
rs6039312	*PLCB1*	rs6056111	*PLCB1*	rs6056114	*PLCB1*	rs3739821	*PIP5KL1*
rs6055550	*PLCB1*	rs6056114	*PLCB1*	rs6056198	*PLCB1*	rs7859	*PIP5KL1*
rs6055562	*PLCB1*	rs6056198	*PLCB1*	rs6056209	*PLCB1*	rs6478803	*PIP5KL1*
rs6055578	*PLCB1*	rs6056209	*PLCB1*	rs6056226	*PLCB1*	rs6478803	*PIP5KL1*
rs6055594	*PLCB1*	rs6056226	*PLCB1*	rs6056230	*PLCB1*	rs6055550	*PLCB1*
rs6055601	*PLCB1*	rs6056230	*PLCB1*	rs6077326	*PLCB1*	rs6086343	*PLCB1*
rs6055603	*PLCB1*	rs6077326	*PLCB1*	rs6077350	*PLCB1*	rs6086345	*PLCB1*
rs6055625	*PLCB1*	rs6077350	*PLCB1*	rs6077396	*PLCB1*	rs6118073	*PLCB1*
rs6055652	*PLCB1*	rs6077396	*PLCB1*	rs6077420	*PLCB1*	rs2179984	*PLCB1*
rs6055685	*PLCB1*	rs6077420	*PLCB1*	rs6077425	*PLCB1*	rs6118075	*PLCB1*
rs6055748	*PLCB1*	rs6077425	*PLCB1*	rs6077434	*PLCB1*	rs6086346	*PLCB1*
rs6055853	*PLCB1*	rs6077434	*PLCB1*	rs6086343	*PLCB1*	rs6039040	*PLCB1*
rs6055858	*PLCB1*	rs6086343	*PLCB1*	rs6086346	*PLCB1*	rs6086348	*PLCB1*
rs6055889	*PLCB1*	rs6086346	*PLCB1*	rs6086348	*PLCB1*	rs6118083	*PLCB1*
rs6055910	*PLCB1*	rs6086348	*PLCB1*	rs6086374	*PLCB1*	rs4142365	*PLCB1*
rs6055912	*PLCB1*	rs6086374	*PLCB1*	rs6086402	*PLCB1*	rs6140549	*PLCB1*
rs6055922	*PLCB1*	rs6086402	*PLCB1*	rs6086458	*PLCB1*	rs6055562	*PLCB1*
rs6055923	*PLCB1*	rs6086458	*PLCB1*	rs6086459	*PLCB1*	rs8114499	*PLCB1*
rs6055926	*PLCB1*	rs6086459	*PLCB1*	rs6086477	*PLCB1*	rs6039049	*PLCB1*
rs6055927	*PLCB1*	rs6086477	*PLCB1*	rs6086493	*PLCB1*	rs4816047	*PLCB1*
rs6055928	*PLCB1*	rs6086493	*PLCB1*	rs6086495	*PLCB1*	rs2327025	*PLCB1*
rs6055944	*PLCB1*	rs6086495	*PLCB1*	rs6086511	*PLCB1*	rs6055578	*PLCB1*
rs6055990	*PLCB1*	rs6086511	*PLCB1*	rs6086518	*PLCB1*	rs978266	*PLCB1*
rs6055995	*PLCB1*	rs6086518	*PLCB1*	rs6086525	*PLCB1*	rs728213	*PLCB1*
rs6056006	*PLCB1*	rs6086525	*PLCB1*	rs6086543	*PLCB1*	rs7271063	*PLCB1*
rs6056024	*PLCB1*	rs6086543	*PLCB1*	rs6086567	*PLCB1*	rs6055594	*PLCB1*

rs6056028	*PLCB1*	rs6086567	*PLCB1*	rs6086570	*PLCB1*	rs6055601	*PLCB1*
rs6056080	*PLCB1*	rs6086570	*PLCB1*	rs6086582	*PLCB1*	rs6055603	*PLCB1*
rs6056111	*PLCB1*	rs6086582	*PLCB1*	rs6086590	*PLCB1*	rs6055625	*PLCB1*
rs6056114	*PLCB1*	rs6086590	*PLCB1*	rs6086617	*PLCB1*	rs2423354	*PLCB1*
rs6056198	*PLCB1*	rs6086617	*PLCB1*	rs6086627	*PLCB1*	rs12480099	*PLCB1*
rs6056209	*PLCB1*	rs6086627	*PLCB1*	rs6086645	*PLCB1*	rs6077326	*PLCB1*
rs6056226	*PLCB1*	rs6086645	*PLCB1*	rs6108152	*PLCB1*	rs6055652	*PLCB1*
rs6077326	*PLCB1*	rs6108152	*PLCB1*	rs6108159	*PLCB1*	rs8118127	*PLCB1*
rs6077332	*PLCB1*	rs6108159	*PLCB1*	rs6108160	*PLCB1*	rs6077332	*PLCB1*
rs6077350	*PLCB1*	rs6108160	*PLCB1*	rs6108174	*PLCB1*	rs4813853	*PLCB1*
rs6077396	*PLCB1*	rs6108174	*PLCB1*	rs6108205	*PLCB1*	rs4399790	*PLCB1*
rs6077411	*PLCB1*	rs6108205	*PLCB1*	rs6118073	*PLCB1*	rs6133556	*PLCB1*
rs6077414	*PLCB1*	rs6118073	*PLCB1*	rs6118075	*PLCB1*	rs10485722	*PLCB1*
rs6077420	*PLCB1*	rs6118075	*PLCB1*	rs6118083	*PLCB1*	rs6086374	*PLCB1*
rs6077425	*PLCB1*	rs6118083	*PLCB1*	rs6118234	*PLCB1*	rs6055685	*PLCB1*
rs6077434	*PLCB1*	rs6118234	*PLCB1*	rs6118268	*PLCB1*	rs4813854	*PLCB1*
rs6086343	*PLCB1*	rs6118268	*PLCB1*	rs6133556	*PLCB1*	rs6039104	*PLCB1*
rs6086345	*PLCB1*	rs6133556	*PLCB1*	rs6133564	*PLCB1*	rs1534897	*PLCB1*
rs6086346	*PLCB1*	rs6133564	*PLCB1*	rs6133566	*PLCB1*	rs6140561	*PLCB1*
rs6086348	*PLCB1*	rs6133566	*PLCB1*	rs6133567	*PLCB1*	rs1474581	*PLCB1*
rs6086374	*PLCB1*	rs6133567	*PLCB1*	rs6133573	*PLCB1*	rs1474937	*PLCB1*
rs6086402	*PLCB1*	rs6133573	*PLCB1*	rs6133610	*PLCB1*	rs995040	*PLCB1*
rs6086458	*PLCB1*	rs6133610	*PLCB1*	rs6133612	*PLCB1*	rs2179138	*PLCB1*
rs6086459	*PLCB1*	rs6133612	*PLCB1*	rs6133635	*PLCB1*	rs6039107	*PLCB1*
rs6086477	*PLCB1*	rs6133635	*PLCB1*	rs6140549	*PLCB1*	rs6039109	*PLCB1*
rs6086490	*PLCB1*	rs6140549	*PLCB1*	rs6140561	*PLCB1*	rs6140562	*PLCB1*
rs6086493	*PLCB1*	rs6140561	*PLCB1*	rs6140562	*PLCB1*	rs727684	*PLCB1*
rs6086495	*PLCB1*	rs6140562	*PLCB1*	rs6140566	*PLCB1*	rs2294259	*PLCB1*
rs6086511	*PLCB1*	rs6140566	*PLCB1*	rs6140595	*PLCB1*	rs6133564	*PLCB1*
rs6086518	*PLCB1*	rs6140595	*PLCB1*	rs6140611	*PLCB1*	rs6133566	*PLCB1*
rs6086525	*PLCB1*	rs6140611	*PLCB1*	rs6140613	*PLCB1*	rs8125486	*PLCB1*
rs6086543	*PLCB1*	rs6140613	*PLCB1*	rs6140619	*PLCB1*	rs6133567	*PLCB1*
rs6086567	*PLCB1*	rs6140619	*PLCB1*	rs6140629	*PLCB1*	rs1033684	*PLCB1*
rs6086570	*PLCB1*	rs6140629	*PLCB1*	rs6140671	*PLCB1*	rs2719807	*PLCB1*
rs6086582	*PLCB1*	rs6140671	*PLCB1*	rs6140677	*PLCB1*	rs10485723	*PLCB1*
rs6086590	*PLCB1*	rs6140677	*PLCB1*	rs6140683	*PLCB1*	rs2745755	*PLCB1*
rs6086617	*PLCB1*	rs6140683	*PLCB1*	rs6140786	*PLCB1*	rs17431073	*PLCB1*
rs6108152	*PLCB1*	rs6140786	*PLCB1*	rs6516403	*PLCB1*	rs2719804	*PLCB1*
rs6108159	*PLCB1*	rs6516403	*PLCB1*	rs708912	*PLCB1*	rs6133573	*PLCB1*
rs6108160	*PLCB1*	rs708912	*PLCB1*	rs708916	*PLCB1*	rs742615	*PLCB1*
rs6108174	*PLCB1*	rs708916	*PLCB1*	rs708920	*PLCB1*	rs6086402	*PLCB1*
rs6108205	*PLCB1*	rs708920	*PLCB1*	rs708925	*PLCB1*	rs2745772	*PLCB1*
rs6118073	*PLCB1*	rs708925	*PLCB1*	rs708931	*PLCB1*	rs2662999	*PLCB1*
rs6118075	*PLCB1*	rs708931	*PLCB1*	rs722665	*PLCB1*	rs2745776	*PLCB1*

rs6118083	*PLCB1*	rs722665	*PLCB1*	rs7269546	*PLCB1*	rs6077350	*PLCB1*
rs6118219	*PLCB1*	rs7269546	*PLCB1*	rs727684	*PLCB1*	rs6055748	*PLCB1*
rs6118234	*PLCB1*	rs727684	*PLCB1*	rs728213	*PLCB1*	rs2719774	*PLCB1*
rs6118252	*PLCB1*	rs728213	*PLCB1*	rs742615	*PLCB1*	rs2719776	*PLCB1*
rs6118257	*PLCB1*	rs742615	*PLCB1*	rs742616	*PLCB1*	rs13037679	*PLCB1*
rs6118262	*PLCB1*	rs742616	*PLCB1*	rs764439	*PLCB1*	rs2745787	*PLCB1*
rs6118268	*PLCB1*	rs764439	*PLCB1*	rs764440	*PLCB1*	rs1232783	*PLCB1*
rs6133556	*PLCB1*	rs764440	*PLCB1*	rs768989	*PLCB1*	rs1232782	*PLCB1*
rs6133564	*PLCB1*	rs768989	*PLCB1*	rs771941	*PLCB1*	rs1605791	*PLCB1*
rs6133566	*PLCB1*	rs771941	*PLCB1*	rs771944	*PLCB1*	rs1005987	*PLCB1*
rs6133567	*PLCB1*	rs771944	*PLCB1*	rs771945	*PLCB1*	rs17347805	*PLCB1*
rs6133573	*PLCB1*	rs771945	*PLCB1*	rs8114499	*PLCB1*	rs708931	*PLCB1*
rs6133610	*PLCB1*	rs8114499	*PLCB1*	rs8117234	*PLCB1*	rs708925	*PLCB1*
rs6133612	*PLCB1*	rs8117234	*PLCB1*	rs8118127	*PLCB1*	rs6140611	*PLCB1*
rs6133635	*PLCB1*	rs8118127	*PLCB1*	rs8123323	*PLCB1*	rs1237829	*PLCB1*
rs6140549	*PLCB1*	rs8123323	*PLCB1*	rs8125486	*PLCB1*	rs1232779	*PLCB1*
rs6140561	*PLCB1*	rs8125486	*PLCB1*	rs8126070	*PLCB1*	rs2745756	*PLCB1*
rs6140562	*PLCB1*	rs8126070	*PLCB1*	rs978266	*PLCB1*	rs708920	*PLCB1*
rs6140613	*PLCB1*	rs978266	*PLCB1*	rs995040	*PLCB1*	rs1935671	*PLCB1*
rs6140619	*PLCB1*	rs995040	*PLCB1*	rs10163076	*PLCB2*	rs1238232	*PLCB1*
rs6140629	*PLCB1*	rs10163076	*PLCB2*	rs12439272	*PLCB2*	rs6140613	*PLCB1*
rs6140671	*PLCB1*	rs12439272	*PLCB2*	rs1869901	*PLCB2*	rs708916	*PLCB1*
rs6140774	*PLCB1*	rs1869901	*PLCB2*	rs3784399	*PLCB2*	rs2662985	*PLCB1*
rs6516403	*PLCB1*	rs3784399	*PLCB2*	rs4924445	*PLCB2*	rs742616	*PLCB1*
rs708912	*PLCB1*	rs4924445	*PLCB2*	rs936213	*PLCB2*	rs708912	*PLCB1*
rs708916	*PLCB1*	rs936213	*PLCB2*	rs961090	*PLCB2*	rs2719795	*PLCB1*
rs708920	*PLCB1*	rs961090	*PLCB2*	rs12798333	*PLCB3*	rs771945	*PLCB1*
rs708925	*PLCB1*	rs12798333	*PLCB3*	rs2244621	*PLCB3*	rs771944	*PLCB1*
rs708931	*PLCB1*	rs2244621	*PLCB3*	rs2244625	*PLCB3*	rs771941	*PLCB1*
rs722665	*PLCB1*	rs2244625	*PLCB3*	rs3741403	*PLCB3*	rs227142	*PLCB1*
rs724110	*PLCB1*	rs3741403	*PLCB3*	rs594942	*PLCB3*	rs227134	*PLCB1*
rs7269546	*PLCB1*	rs594942	*PLCB3*	rs660442	*PLCB3*	rs8115925	*PLCB1*
rs7271063	*PLCB1*	rs660442	*PLCB3*	rs915987	*PLCB3*	rs227133	*PLCB1*
rs727684	*PLCB1*	rs915987	*PLCB3*	rs1028338	*PLCB4*	rs227130	*PLCB1*
rs728213	*PLCB1*	rs1028338	*PLCB4*	rs11087845	*PLCB4*	rs2221695	*PLCB1*
rs742615	*PLCB1*	rs11087845	*PLCB4*	rs11904996	*PLCB4*	rs764440	*PLCB1*
rs742616	*PLCB1*	rs11904996	*PLCB4*	rs13041524	*PLCB4*	rs6086458	*PLCB1*
rs764439	*PLCB1*	rs13041524	*PLCB4*	rs13044386	*PLCB4*	rs764439	*PLCB1*
rs764440	*PLCB1*	rs13044386	*PLCB4*	rs1321581	*PLCB4*	rs6140619	*PLCB1*
rs768989	*PLCB1*	rs1321581	*PLCB4*	rs1407101	*PLCB4*	rs17362299	*PLCB1*
rs771941	*PLCB1*	rs1407101	*PLCB4*	rs16995654	*PLCB4*	rs6086459	*PLCB1*
rs771944	*PLCB1*	rs16995654	*PLCB4*	rs16995731	*PLCB4*	rs4471960	*PLCB1*
rs771945	*PLCB1*	rs16995731	*PLCB4*	rs16995800	*PLCB4*	rs10485724	*PLCB1*
rs8114499	*PLCB1*	rs16995800	*PLCB4*	rs1997696	*PLCB4*	rs2423356	*PLCB1*

rs8115925	*PLCB1*	rs1997696	*PLCB4*	rs2072954	*PLCB4*	rs1342585	*PLCB1*
rs8117234	*PLCB1*	rs2072954	*PLCB4*	rs2076393	*PLCB4*	rs4813863	*PLCB1*
rs8118127	*PLCB1*	rs2076393	*PLCB4*	rs2179321	*PLCB4*	rs2327070	*PLCB1*
rs8123323	*PLCB1*	rs2179321	*PLCB4*	rs2206138	*PLCB4*	rs6055853	*PLCB1*
rs8125486	*PLCB1*	rs2206138	*PLCB4*	rs2208297	*PLCB4*	rs6055858	*PLCB1*
rs8126070	*PLCB1*	rs2208297	*PLCB4*	rs2276483	*PLCB4*	rs1534968	*PLCB1*
rs8126112	*PLCB1*	rs2276483	*PLCB4*	rs2276484	*PLCB4*	rs6140629	*PLCB1*
rs978266	*PLCB1*	rs2276484	*PLCB4*	rs2299676	*PLCB4*	rs2050090	*PLCB1*
rs995040	*PLCB1*	rs2299676	*PLCB4*	rs2299679	*PLCB4*	rs6118219	*PLCB1*
rs12439272	*PLCB2*	rs2299679	*PLCB4*	rs2327162	*PLCB4*	rs6086477	*PLCB1*
rs1869901	*PLCB2*	rs2327162	*PLCB4*	rs2327164	*PLCB4*	rs2423360	*PLCB1*
rs3784399	*PLCB2*	rs2327164	*PLCB4*	rs3787309	*PLCB4*	rs2423361	*PLCB1*
rs4924445	*PLCB2*	rs3787309	*PLCB4*	rs3819579	*PLCB4*	rs1569604	*PLCB1*
rs936213	*PLCB2*	rs3819579	*PLCB4*	rs4141984	*PLCB4*	rs722665	*PLCB1*
rs12798333	*PLCB3*	rs4141984	*PLCB4*	rs4369940	*PLCB4*	rs6118234	*PLCB1*
rs2244621	*PLCB3*	rs4369940	*PLCB4*	rs5011374	*PLCB4*	rs2143205	*PLCB1*
rs2244625	*PLCB3*	rs5011374	*PLCB4*	rs6039386	*PLCB4*	rs2423363	*PLCB1*
rs3741403	*PLCB3*	rs6039386	*PLCB4*	rs6039393	*PLCB4*	rs2064272	*PLCB1*
rs594942	*PLCB3*	rs6039393	*PLCB4*	rs6039399	*PLCB4*	rs2423364	*PLCB1*
rs915987	*PLCB3*	rs6039399	*PLCB4*	rs6039410	*PLCB4*	rs2423366	*PLCB1*
rs1028338	*PLCB4*	rs6039410	*PLCB4*	rs6039442	*PLCB4*	rs6055889	*PLCB1*
rs13041524	*PLCB4*	rs6039442	*PLCB4*	rs6039443	*PLCB4*	rs2179440	*PLCB1*
rs13044386	*PLCB4*	rs6039443	*PLCB4*	rs6056386	*PLCB4*	rs6086490	*PLCB1*
rs1321581	*PLCB4*	rs6056386	*PLCB4*	rs6056440	*PLCB4*	rs3817881	*PLCB1*
rs1407101	*PLCB4*	rs6056440	*PLCB4*	rs6056448	*PLCB4*	rs11087808	*PLCB1*
rs16995573	*PLCB4*	rs6056448	*PLCB4*	rs6056500	*PLCB4*	rs6039189	*PLCB1*
rs16995731	*PLCB4*	rs6056500	*PLCB4*	rs6056505	*PLCB4*	rs17436253	*PLCB1*
rs17481185	*PLCB4*	rs6056505	*PLCB4*	rs6056519	*PLCB4*	rs6086493	*PLCB1*
rs1997696	*PLCB4*	rs6056519	*PLCB4*	rs6056522	*PLCB4*	rs6086495	*PLCB1*
rs2072954	*PLCB4*	rs6056522	*PLCB4*	rs6056526	*PLCB4*	rs2206423	*PLCB1*
rs2076393	*PLCB4*	rs6056526	*PLCB4*	rs6056552	*PLCB4*	rs6039190	*PLCB1*
rs2179321	*PLCB4*	rs6056552	*PLCB4*	rs6056570	*PLCB4*	rs6039191	*PLCB1*
rs2206138	*PLCB4*	rs6056570	*PLCB4*	rs6056595	*PLCB4*	rs6108152	*PLCB1*
rs2208297	*PLCB4*	rs6056595	*PLCB4*	rs6056628	*PLCB4*	rs13040221	*PLCB1*
rs2224357	*PLCB4*	rs6056628	*PLCB4*	rs6056645	*PLCB4*	rs6118252	*PLCB1*
rs2276483	*PLCB4*	rs6056645	*PLCB4*	rs6077510	*PLCB4*	rs2103653	*PLCB1*
rs2276484	*PLCB4*	rs6077510	*PLCB4*	rs6077511	*PLCB4*	rs6055910	*PLCB1*
rs2299676	*PLCB4*	rs6077511	*PLCB4*	rs6086762	*PLCB4*	rs1883503	*PLCB1*
rs2299679	*PLCB4*	rs6086762	*PLCB4*	rs6086799	*PLCB4*	rs6055912	*PLCB1*
rs2327162	*PLCB4*	rs6086799	*PLCB4*	rs6086897	*PLCB4*	rs6086511	*PLCB1*
rs2327164	*PLCB4*	rs6086897	*PLCB4*	rs6086900	*PLCB4*	rs6118257	*PLCB1*
rs2876163	*PLCB4*	rs6086900	*PLCB4*	rs6086904	*PLCB4*	rs12624339	*PLCB1*
rs3787309	*PLCB4*	rs6086904	*PLCB4*	rs6108255	*PLCB4*	rs2327046	*PLCB1*
rs3819579	*PLCB4*	rs6108255	*PLCB4*	rs6108263	*PLCB4*	rs6055922	*PLCB1*

rs4369940	*PLCB4*	rs6108263	*PLCB4*	rs6118479	*PLCB4*	rs6055923	*PLCB1*
rs5011374	*PLCB4*	rs6118479	*PLCB4*	rs6118505	*PLCB4*	rs6108159	*PLCB1*
rs6039386	*PLCB4*	rs6118505	*PLCB4*	rs6118508	*PLCB4*	rs6055926	*PLCB1*
rs6039393	*PLCB4*	rs6118508	*PLCB4*	rs6118558	*PLCB4*	rs6055927	*PLCB1*
rs6039410	*PLCB4*	rs6118558	*PLCB4*	rs6118591	*PLCB4*	rs6055928	*PLCB1*
rs6039442	*PLCB4*	rs6118591	*PLCB4*	rs6118611	*PLCB4*	rs6086518	*PLCB1*
rs6039443	*PLCB4*	rs6118611	*PLCB4*	rs6118616	*PLCB4*	rs6108160	*PLCB1*
rs6056386	*PLCB4*	rs6118616	*PLCB4*	rs6118618	*PLCB4*	rs6086525	*PLCB1*
rs6056427	*PLCB4*	rs6118618	*PLCB4*	rs6133703	*PLCB4*	rs6118262	*PLCB1*
rs6056440	*PLCB4*	rs6133703	*PLCB4*	rs6133707	*PLCB4*	rs6039206	*PLCB1*
rs6056448	*PLCB4*	rs6133707	*PLCB4*	rs6140861	*PLCB4*	rs4813865	*PLCB1*
rs6056505	*PLCB4*	rs6140861	*PLCB4*	rs6140909	*PLCB4*	rs2223538	*PLCB1*
rs6056519	*PLCB4*	rs6140909	*PLCB4*	rs6516454	*PLCB4*	rs6055944	*PLCB1*
rs6056526	*PLCB4*	rs6516454	*PLCB4*	rs725941	*PLCB4*	rs6118268	*PLCB1*
rs6056552	*PLCB4*	rs725941	*PLCB4*	rs7269910	*PLCB4*	rs8126112	*PLCB1*
rs6056570	*PLCB4*	rs7269910	*PLCB4*	rs7272444	*PLCB4*	rs6077396	*PLCB1*
rs6056595	*PLCB4*	rs7272444	*PLCB4*	rs8115948	*PLCB4*	rs8126070	*PLCB1*
rs6056628	*PLCB4*	rs8115948	*PLCB4*	rs8183334	*PLCB4*	rs4432538	*PLCB1*
rs6077510	*PLCB4*	rs8183334	*PLCB4*	rs976649	*PLCB4*	rs4419296	*PLCB1*
rs6077511	*PLCB4*	rs976649	*PLCB4*	rs2212042	*PLCD1*	rs2223837	*PLCB1*
rs6077516	*PLCB4*	rs2212042	*PLCD1*	rs2226462	*PLCD1*	rs6086543	*PLCB1*
rs6086762	*PLCB4*	rs2226462	*PLCD1*	rs4389435	*PLCD1*	rs12053642	*PLCB1*
rs6086799	*PLCB4*	rs4389435	*PLCD1*	rs6599099	*PLCD1*	rs6039211	*PLCB1*
rs6086834	*PLCB4*	rs6599099	*PLCD1*	rs928807	*PLCD1*	rs1018443	*PLCB1*
rs6086865	*PLCB4*	rs928807	*PLCD1*	rs1052169	*PLCD3*	rs6140671	*PLCB1*
rs6108255	*PLCB4*	rs1052169	*PLCD3*	rs1053733	*PLCD3*	rs6055990	*PLCB1*
rs6108263	*PLCB4*	rs1053733	*PLCD3*	rs12944434	*PLCD3*	rs6108174	*PLCB1*
rs6118479	*PLCB4*	rs12944434	*PLCD3*	rs2239925	*PLCD3*	rs1033566	*PLCB1*
rs6118505	*PLCB4*	rs2239925	*PLCD3*	rs2269746	*PLCD3*	rs6055995	*PLCB1*
rs6118508	*PLCB4*	rs2269746	*PLCD3*	rs2285426	*PLCD3*	rs6056006	*PLCB1*
rs6118558	*PLCB4*	rs2285426	*PLCD3*	rs2285427	*PLCD3*	rs2876140	*PLCB1*
rs6118591	*PLCB4*	rs2285427	*PLCD3*	rs3744760	*PLCD3*	rs6039237	*PLCB1*
rs6118611	*PLCB4*	rs3744760	*PLCD3*	rs3744761	*PLCD3*	rs768989	*PLCB1*
rs6118616	*PLCB4*	rs3744761	*PLCD3*	rs4362432	*PLCD3*	rs6056024	*PLCB1*
rs6118618	*PLCB4*	rs4362432	*PLCD3*	rs4986172	*PLCD3*	rs2295179	*PLCB1*
rs6133703	*PLCB4*	rs4986172	*PLCD3*	rs713101	*PLCD3*	rs6056028	*PLCB1*
rs6133707	*PLCB4*	rs713101	*PLCD3*	rs7207047	*PLCD3*	rs16995121	*PLCB1*
rs6140861	*PLCB4*	rs7207047	*PLCD3*	rs7223320	*PLCD3*	rs6077411	*PLCB1*
rs6516454	*PLCB4*	rs7223320	*PLCD3*	rs8069937	*PLCD3*	rs6133610	*PLCB1*
rs725941	*PLCB4*	rs8069937	*PLCD3*	rs8070447	*PLCD3*	rs8123323	*PLCB1*
rs7265537	*PLCB4*	rs8070447	*PLCD3*	rs12989189	*PLCD4*	rs2076685	*PLCB1*
rs7268671	*PLCB4*	rs12989189	*PLCD4*	rs13032927	*PLCD4*	rs17446308	*PLCB1*
rs7272444	*PLCB4*	rs13032927	*PLCD4*	rs3770214	*PLCD4*	rs8117234	*PLCB1*
rs8115510	*PLCB4*	rs3770214	*PLCD4*	rs3845836	*PLCD4*	rs6077414	*PLCB1*

rs8115948	*PLCB4*	rs3845836	*PLCD4*	rs10786152	*PLCE1*	rs1555212	*PLCB1*
rs8183334	*PLCB4*	rs10786152	*PLCE1*	rs10786155	*PLCE1*	rs1015170	*PLCB1*
rs964310	*PLCB4*	rs10786155	*PLCE1*	rs10882378	*PLCE1*	rs17446441	*PLCB1*
rs976649	*PLCB4*	rs10882378	*PLCE1*	rs10882380	*PLCE1*	rs6086567	*PLCB1*
rs11922130	*PLCD1*	rs10882380	*PLCE1*	rs10882381	*PLCE1*	rs6133612	*PLCB1*
rs2226462	*PLCD1*	rs10882381	*PLCE1*	rs10882406	*PLCE1*	rs6086570	*PLCB1*
rs6599099	*PLCD1*	rs10882406	*PLCE1*	rs10882412	*PLCE1*	rs2143266	*PLCB1*
rs928807	*PLCD1*	rs10882412	*PLCE1*	rs11187749	*PLCE1*	rs2179478	*PLCB1*
rs9861030	*PLCD1*	rs11187749	*PLCE1*	rs11187789	*PLCE1*	rs6086582	*PLCB1*
rs1052169	*PLCD3*	rs11187789	*PLCE1*	rs11187808	*PLCE1*	rs6056080	*PLCB1*
rs1053733	*PLCD3*	rs11187808	*PLCE1*	rs11187815	*PLCE1*	rs6077420	*PLCB1*
rs12944434	*PLCD3*	rs11187815	*PLCE1*	rs11187825	*PLCE1*	rs6086590	*PLCB1*
rs2239925	*PLCD3*	rs11187825	*PLCE1*	rs11187842	*PLCE1*	rs1040496	*PLCB1*
rs2269746	*PLCD3*	rs11187842	*PLCE1*	rs1223577	*PLCE1*	rs4496390	*PLCB1*
rs2285426	*PLCD3*	rs1223577	*PLCE1*	rs1223583	*PLCE1*	rs4816085	*PLCB1*
rs2285427	*PLCD3*	rs1223583	*PLCE1*	rs1223585	*PLCE1*	rs6039268	*PLCB1*
rs3744760	*PLCD3*	rs1223585	*PLCE1*	rs12244826	*PLCE1*	rs2206489	*PLCB1*
rs4362432	*PLCD3*	rs12244826	*PLCE1*	rs12263737	*PLCE1*	rs6056111	*PLCB1*
rs713101	*PLCD3*	rs12263737	*PLCE1*	rs12766693	*PLCE1*	rs2294597	*PLCB1*
rs7207047	*PLCD3*	rs12766693	*PLCE1*	rs12769135	*PLCE1*	rs6056114	*PLCB1*
rs7223320	*PLCD3*	rs12769135	*PLCE1*	rs1408820	*PLCE1*	rs6516403	*PLCB1*
rs7224944	*PLCD3*	rs1408820	*PLCE1*	rs1547643	*PLCE1*	rs1967681	*PLCB1*
rs8069937	*PLCD3*	rs1547643	*PLCE1*	rs17109869	*PLCE1*	rs11906514	*PLCB1*
rs12989189	*PLCD4*	rs17109869	*PLCE1*	rs17109928	*PLCE1*	rs1474683	*PLCB1*
rs10786152	*PLCE1*	rs17109928	*PLCE1*	rs17416616	*PLCE1*	rs2076409	*PLCB1*
rs10786155	*PLCE1*	rs17416616	*PLCE1*	rs1776842	*PLCE1*	rs4816089	*PLCB1*
rs10882378	*PLCE1*	rs1776842	*PLCE1*	rs1776946	*PLCE1*	rs6077425	*PLCB1*
rs10882380	*PLCE1*	rs1776946	*PLCE1*	rs1858608	*PLCE1*	rs4816090	*PLCB1*
rs10882381	*PLCE1*	rs1858608	*PLCE1*	rs1925243	*PLCE1*	rs724110	*PLCB1*
rs10882406	*PLCE1*	rs1925243	*PLCE1*	rs1935960	*PLCE1*	rs2876145	*PLCB1*
rs10882412	*PLCE1*	rs1935960	*PLCE1*	rs1935961	*PLCE1*	rs6086617	*PLCB1*
rs11187749	*PLCE1*	rs1935961	*PLCE1*	rs1998709	*PLCE1*	rs6039298	*PLCB1*
rs11187789	*PLCE1*	rs1998709	*PLCE1*	rs2077218	*PLCE1*	rs6056198	*PLCB1*
rs11187808	*PLCE1*	rs2077218	*PLCE1*	rs2182093	*PLCE1*	rs6077434	*PLCB1*
rs11187815	*PLCE1*	rs2182093	*PLCE1*	rs2209442	*PLCE1*	rs11698656	*PLCB1*
rs11187825	*PLCE1*	rs2209442	*PLCE1*	rs2226170	*PLCE1*	rs6086627	*PLCB1*
rs11187828	*PLCE1*	rs2226170	*PLCE1*	rs2274223	*PLCE1*	rs10485728	*PLCB1*
rs11187842	*PLCE1*	rs2274223	*PLCE1*	rs2689693	*PLCE1*	rs3902336	*PLCB1*
rs11593126	*PLCE1*	rs2689693	*PLCE1*	rs2689694	*PLCE1*	rs6056209	*PLCB1*
rs1223577	*PLCE1*	rs2689694	*PLCE1*	rs2689698	*PLCE1*	rs6039307	*PLCB1*
rs1223583	*PLCE1*	rs2689698	*PLCE1*	rs2689700	*PLCE1*	rs6140774	*PLCB1*
rs1223585	*PLCE1*	rs2689700	*PLCE1*	rs2764343	*PLCE1*	rs6133635	*PLCB1*
rs12263737	*PLCE1*	rs2764343	*PLCE1*	rs2797998	*PLCE1*	rs6108205	*PLCB1*
rs12766693	*PLCE1*	rs2797998	*PLCE1*	rs2798001	*PLCE1*	rs4083408	*PLCB1*

rs12769135	*PLCE1*	rs2798001	*PLCE1*	rs2860746	*PLCE1*	rs6039312	*PLCB1*
rs1408820	*PLCE1*	rs2860746	*PLCE1*	rs3740360	*PLCE1*	rs3848835	*PLCB1*
rs1547643	*PLCE1*	rs3740360	*PLCE1*	rs3765524	*PLCE1*	rs7269546	*PLCB1*
rs17109869	*PLCE1*	rs3765524	*PLCE1*	rs3781264	*PLCE1*	rs6056226	*PLCB1*
rs17416616	*PLCE1*	rs3781264	*PLCE1*	rs4545470	*PLCE1*	rs1047383	*PLCB1*
rs17516904	*PLCE1*	rs4545470	*PLCE1*	rs4917450	*PLCE1*	rs6056230	*PLCB1*
rs1776842	*PLCE1*	rs4917450	*PLCE1*	rs4918070	*PLCE1*	rs4924445	*PLCB2*
rs1776946	*PLCE1*	rs4918070	*PLCE1*	rs4918082	*PLCE1*	rs936213	*PLCB2*
rs1858608	*PLCE1*	rs4918082	*PLCE1*	rs4918188	*PLCE1*	rs12439272	*PLCB2*
rs1925243	*PLCE1*	rs4918188	*PLCE1*	rs6583926	*PLCE1*	rs1869901	*PLCB2*
rs1935960	*PLCE1*	rs6583926	*PLCE1*	rs7085672	*PLCE1*	rs3784399	*PLCB2*
rs1935961	*PLCE1*	rs7085672	*PLCE1*	rs7093524	*PLCE1*	rs10163076	*PLCB2*
rs1998709	*PLCE1*	rs7093524	*PLCE1*	rs753724	*PLCE1*	rs961090	*PLCB2*
rs2077218	*PLCE1*	rs753724	*PLCE1*	rs7908334	*PLCE1*	rs961090	*PLCB2*
rs2182093	*PLCE1*	rs7908334	*PLCE1*	rs7919066	*PLCE1*	rs3741403	*PLCB3*
rs2209442	*PLCE1*	rs7919066	*PLCE1*	rs829232	*PLCE1*	rs594942	*PLCB3*
rs2226170	*PLCE1*	rs829232	*PLCE1*	rs12624863	*PLCG1*	rs12798333	*PLCB3*
rs2274223	*PLCE1*	rs12624863	*PLCG1*	rs2235360	*PLCG1*	rs2244625	*PLCB3*
rs2689693	*PLCE1*	rs2235360	*PLCG1*	rs2235366	*PLCG1*	rs2244621	*PLCB3*
rs2689694	*PLCE1*	rs2235366	*PLCG1*	rs6129760	*PLCG1*	rs915987	*PLCB3*
rs2689698	*PLCE1*	rs6129760	*PLCG1*	rs753381	*PLCG1*	rs660442	*PLCB3*
rs2689700	*PLCE1*	rs753381	*PLCG1*	rs10445097	*PLCG2*	rs660442	*PLCB3*
rs2798001	*PLCE1*	rs10445097	*PLCG2*	rs10514519	*PLCG2*	rs6056386	*PLCB4*
rs2860746	*PLCE1*	rs10514519	*PLCG2*	rs11643875	*PLCG2*	rs6086762	*PLCB4*
rs3740360	*PLCE1*	rs11643875	*PLCG2*	rs11862662	*PLCG2*	rs6118479	*PLCB4*
rs3765524	*PLCE1*	rs11862662	*PLCG2*	rs12446070	*PLCG2*	rs6039386	*PLCB4*
rs3781264	*PLCE1*	rs12446070	*PLCG2*	rs12446596	*PLCG2*	rs6108255	*PLCB4*
rs4394764	*PLCE1*	rs12446596	*PLCG2*	rs12448055	*PLCG2*	rs8115948	*PLCB4*
rs4918070	*PLCE1*	rs12448055	*PLCG2*	rs12448334	*PLCG2*	rs6140861	*PLCB4*
rs4918082	*PLCE1*	rs12448334	*PLCG2*	rs12598194	*PLCG2*	rs964310	*PLCB4*
rs4918188	*PLCE1*	rs12598194	*PLCG2*	rs12598402	*PLCG2*	rs6039393	*PLCB4*
rs6583926	*PLCE1*	rs12598402	*PLCG2*	rs12599264	*PLCG2*	rs13041524	*PLCB4*
rs7085672	*PLCE1*	rs12599264	*PLCG2*	rs12918369	*PLCG2*	rs6108263	*PLCB4*
rs753724	*PLCE1*	rs12918369	*PLCG2*	rs12921780	*PLCG2*	rs16995573	*PLCB4*
rs7908334	*PLCE1*	rs12921780	*PLCG2*	rs16956011	*PLCG2*	rs13044386	*PLCB4*
rs7919066	*PLCE1*	rs16956011	*PLCG2*	rs16956040	*PLCG2*	rs6118505	*PLCB4*
rs829232	*PLCE1*	rs16956040	*PLCG2*	rs17793122	*PLCG2*	rs2224357	*PLCB4*
rs12624863	*PLCG1*	rs17793122	*PLCG2*	rs3922849	*PLCG2*	rs6056427	*PLCB4*
rs2235360	*PLCG1*	rs3922849	*PLCG2*	rs3934954	*PLCG2*	rs6118508	*PLCB4*
rs6129760	*PLCG1*	rs3934954	*PLCG2*	rs3936112	*PLCG2*	rs6056440	*PLCB4*
rs753381	*PLCG1*	rs3936112	*PLCG2*	rs4072683	*PLCG2*	rs6086799	*PLCB4*
rs10445097	*PLCG2*	rs4072683	*PLCG2*	rs4073828	*PLCG2*	rs6056448	*PLCB4*
rs10514519	*PLCG2*	rs4073828	*PLCG2*	rs4133124	*PLCG2*	rs2208297	*PLCB4*
rs11643875	*PLCG2*	rs4133124	*PLCG2*	rs4243211	*PLCG2*	rs1407101	*PLCB4*

rs11644436	*PLCG2*	rs4243211	*PLCG2*	rs4243218	*PLCG2*	rs1321581	*PLCB4*
rs11644646	*PLCG2*	rs4243218	*PLCG2*	rs4243226	*PLCG2*	rs2876163	*PLCB4*
rs11859107	*PLCG2*	rs4243226	*PLCG2*	rs4254322	*PLCG2*	rs6039410	*PLCB4*
rs11862662	*PLCG2*	rs4254322	*PLCG2*	rs4286103	*PLCG2*	rs8183334	*PLCB4*
rs11864701	*PLCG2*	rs4286103	*PLCG2*	rs4306504	*PLCG2*	rs5011374	*PLCB4*
rs12446070	*PLCG2*	rs4306504	*PLCG2*	rs4312298	*PLCG2*	rs6056505	*PLCB4*
rs12446596	*PLCG2*	rs4312298	*PLCG2*	rs4325546	*PLCG2*	rs2206138	*PLCB4*
rs12448055	*PLCG2*	rs4325546	*PLCG2*	rs4328435	*PLCG2*	rs6118558	*PLCB4*
rs12448334	*PLCG2*	rs4328435	*PLCG2*	rs4369658	*PLCG2*	rs6516454	*PLCB4*
rs12596639	*PLCG2*	rs4369658	*PLCG2*	rs4369659	*PLCG2*	rs16995731	*PLCB4*
rs12598194	*PLCG2*	rs4369659	*PLCG2*	rs4398100	*PLCG2*	rs6056519	*PLCB4*
rs12598402	*PLCG2*	rs4398100	*PLCG2*	rs4405545	*PLCG2*	rs2327162	*PLCB4*
rs12599264	*PLCG2*	rs4405545	*PLCG2*	rs4508413	*PLCG2*	rs6077510	*PLCB4*
rs12716928	*PLCG2*	rs4508413	*PLCG2*	rs4580153	*PLCG2*	rs6077511	*PLCB4*
rs12918369	*PLCG2*	rs4580153	*PLCG2*	rs4580154	*PLCG2*	rs6086834	*PLCB4*
rs12921780	*PLCG2*	rs4580154	*PLCG2*	rs4603554	*PLCG2*	rs6056526	*PLCB4*
rs13331678	*PLCG2*	rs4603554	*PLCG2*	rs4611452	*PLCG2*	rs2327164	*PLCB4*
rs16956040	*PLCG2*	rs4611452	*PLCG2*	rs4888181	*PLCG2*	rs7272444	*PLCB4*
rs17203310	*PLCG2*	rs4888181	*PLCG2*	rs4888191	*PLCG2*	rs6056552	*PLCB4*
rs17793122	*PLCG2*	rs4888191	*PLCG2*	rs4888197	*PLCG2*	rs8115510	*PLCB4*
rs3922849	*PLCG2*	rs4888197	*PLCG2*	rs4889384	*PLCG2*	rs6039442	*PLCB4*
rs3935743	*PLCG2*	rs4889384	*PLCG2*	rs4889393	*PLCG2*	rs7268671	*PLCB4*
rs3935877	*PLCG2*	rs4889393	*PLCG2*	rs4889411	*PLCG2*	rs6077516	*PLCB4*
rs3936112	*PLCG2*	rs4889411	*PLCG2*	rs4889422	*PLCG2*	rs6039443	*PLCB4*
rs4072683	*PLCG2*	rs4889422	*PLCG2*	rs4889426	*PLCG2*	rs6056570	*PLCB4*
rs4073828	*PLCG2*	rs4889426	*PLCG2*	rs4889432	*PLCG2*	rs17481185	*PLCB4*
rs4074445	*PLCG2*	rs4889432	*PLCG2*	rs4889448	*PLCG2*	rs725941	*PLCB4*
rs4133124	*PLCG2*	rs4889448	*PLCG2*	rs4997772	*PLCG2*	rs2299676	*PLCB4*
rs4133125	*PLCG2*	rs4997772	*PLCG2*	rs6420427	*PLCG2*	rs6086865	*PLCB4*
rs4243211	*PLCG2*	rs6420427	*PLCG2*	rs6564915	*PLCG2*	rs1028338	*PLCB4*
rs4243218	*PLCG2*	rs6564915	*PLCG2*	rs6564940	*PLCG2*	rs6133703	*PLCB4*
rs4243221	*PLCG2*	rs6564940	*PLCG2*	rs7185362	*PLCG2*	rs7265537	*PLCB4*
rs4254322	*PLCG2*	rs7185362	*PLCG2*	rs7197601	*PLCG2*	rs2179321	*PLCB4*
rs4284633	*PLCG2*	rs7187863	*PLCG2*	rs7201045	*PLCG2*	rs6118591	*PLCB4*
rs4306504	*PLCG2*	rs7197601	*PLCG2*	rs7203619	*PLCG2*	rs1997696	*PLCB4*
rs4312298	*PLCG2*	rs7201045	*PLCG2*	rs7342694	*PLCG2*	rs6133707	*PLCB4*
rs4325546	*PLCG2*	rs7203619	*PLCG2*	rs7499275	*PLCG2*	rs6056595	*PLCB4*
rs4328435	*PLCG2*	rs7342694	*PLCG2*	rs7499440	*PLCG2*	rs3787309	*PLCB4*
rs4369658	*PLCG2*	rs7499275	*PLCG2*	rs7500286	*PLCG2*	rs3819579	*PLCB4*
rs4369659	*PLCG2*	rs7499440	*PLCG2*	rs8043593	*PLCG2*	rs2299679	*PLCB4*
rs4398100	*PLCG2*	rs7500286	*PLCG2*	rs8043619	*PLCG2*	rs6118611	*PLCB4*
rs4405545	*PLCG2*	rs8043593	*PLCG2*	rs8047356	*PLCG2*	rs4369940	*PLCB4*
rs4405546	*PLCG2*	rs8043619	*PLCG2*	rs8055043	*PLCG2*	rs6118616	*PLCB4*
rs4456499	*PLCG2*	rs8047356	*PLCG2*	rs8055576	*PLCG2*	rs6118618	*PLCB4*

rs4508413	*PLCG2*	rs8055043	*PLCG2*	rs8056564	*PLCG2*	rs6056628	*PLCB4*
rs4580153	*PLCG2*	rs8055576	*PLCG2*	rs8063120	*PLCG2*	rs976649	*PLCB4*
rs4580154	*PLCG2*	rs8056564	*PLCG2*	rs8063604	*PLCG2*	rs2072954	*PLCB4*
rs4603554	*PLCG2*	rs8063120	*PLCG2*	rs8063813	*PLCG2*	rs2276483	*PLCB4*
rs4611452	*PLCG2*	rs8063604	*PLCG2*	rs9928191	*PLCG2*	rs2276484	*PLCB4*
rs4888179	*PLCG2*	rs8063813	*PLCG2*	rs9932716	*PLCG2*	rs2076393	*PLCB4*
rs4888181	*PLCG2*	rs9928191	*PLCG2*	rs9937704	*PLCG2*	rs6086897	*PLCB4*
rs4888184	*PLCG2*	rs9932716	*PLCG2*	rs9938623	*PLCG2*	rs6086900	*PLCB4*
rs4888191	*PLCG2*	rs9937704	*PLCG2*	rs9938764	*PLCG2*	rs6086904	*PLCB4*
rs4888197	*PLCG2*	rs9938623	*PLCG2*	rs9938835	*PLCG2*	rs6056645	*PLCB4*
rs4889384	*PLCG2*	rs9938764	*PLCG2*	rs1125539	*PLCH1*	rs6056645	*PLCB4*
rs4889393	*PLCG2*	rs9938835	*PLCG2*	rs1850847	*PLCH1*	rs928807	*PLCD1*
rs4889411	*PLCG2*	rs1125539	*PLCH1*	rs359565	*PLCH1*	rs9861030	*PLCD1*
rs4889422	*PLCG2*	rs1850847	*PLCH1*	rs359570	*PLCH1*	rs11922130	*PLCD1*
rs4889425	*PLCG2*	rs359565	*PLCH1*	rs4679748	*PLCH1*	rs6599099	*PLCD1*
rs4889426	*PLCG2*	rs359570	*PLCH1*	rs517971	*PLCH1*	rs2226462	*PLCD1*
rs4889428	*PLCG2*	rs4679748	*PLCH1*	rs592982	*PLCH1*	rs2212042	*PLCD1*
rs4889432	*PLCG2*	rs517971	*PLCH1*	rs6440997	*PLCH1*	rs4389435	*PLCD1*
rs4889436	*PLCG2*	rs592982	*PLCH1*	rs7629025	*PLCH1*	rs4389435	*PLCD1*
rs4889444	*PLCG2*	rs6440997	*PLCH1*	rs7637342	*PLCH1*	rs1053733	*PLCD3*
rs4889448	*PLCG2*	rs7629025	*PLCH1*	rs10910078	*PLCH2*	rs2269746	*PLCD3*
rs4997772	*PLCG2*	rs7637342	*PLCH1*	rs12049628	*PLCH2*	rs2239925	*PLCD3*
rs6420427	*PLCG2*	rs10910078	*PLCH2*	rs13376356	*PLCH2*	rs1052169	*PLCD3*
rs6564915	*PLCG2*	rs12049628	*PLCH2*	rs2236395	*PLCH2*	rs2285426	*PLCD3*
rs6564940	*PLCG2*	rs13376356	*PLCH2*	rs2494626	*PLCH2*	rs2285427	*PLCD3*
rs7185362	*PLCG2*	rs2236395	*PLCH2*	rs3762444	*PLCH2*	rs713101	*PLCD3*
rs7187863	*PLCG2*	rs2494626	*PLCH2*	rs7535528	*PLCH2*	rs3744760	*PLCD3*
rs7194131	*PLCG2*	rs3762444	*PLCH2*	rs1021266	*PLCZ1*	rs4362432	*PLCD3*
rs7197601	*PLCG2*	rs7535528	*PLCH2*	rs1021267	*PLCZ1*	rs7207047	*PLCD3*
rs7201045	*PLCG2*	rs1021266	*PLCZ1*	rs1027032	*PLCZ1*	rs12944434	*PLCD3*
rs7202205	*PLCG2*	rs1021267	*PLCZ1*	rs10841075	*PLCZ1*	rs7223320	*PLCD3*
rs7203619	*PLCG2*	rs1027032	*PLCZ1*	rs10841077	*PLCZ1*	rs7224944	*PLCD3*
rs7342694	*PLCG2*	rs10841075	*PLCZ1*	rs12823264	*PLCZ1*	rs8069937	*PLCD3*
rs7499275	*PLCG2*	rs10841077	*PLCZ1*	rs1386398	*PLCZ1*	rs4986172	*PLCD3*
rs7499440	*PLCG2*	rs12823264	*PLCZ1*	rs1471891	*PLCZ1*	rs8070447	*PLCD3*
rs7500286	*PLCG2*	rs1386398	*PLCZ1*	rs1550990	*PLCZ1*	rs8070447	*PLCD3*
rs8043593	*PLCG2*	rs1471891	*PLCZ1*	rs2306798	*PLCZ1*	rs12989189	*PLCD4*
rs8043619	*PLCG2*	rs1550990	*PLCZ1*	rs4764417	*PLCZ1*	rs3845836	*PLCD4*
rs8047356	*PLCG2*	rs2306798	*PLCZ1*	rs513426	*PLCZ1*	rs3770214	*PLCD4*
rs8055043	*PLCG2*	rs4764417	*PLCZ1*	rs7485517	*PLCZ1*	rs3770214	*PLCD4*
rs8055576	*PLCG2*	rs513426	*PLCZ1*	rs7972408	*PLCZ1*	rs1223577	*PLCE1*
rs8056564	*PLCG2*	rs7485517	*PLCZ1*	rs7974908	*PLCZ1*	rs11187749	*PLCE1*
rs8062633	*PLCG2*	rs7972408	*PLCZ1*	rs901528	*PLCZ1*	rs7085672	*PLCE1*
rs8063120	*PLCG2*	rs7974908	*PLCZ1*	rs969489	*PLCZ1*	rs11593126	*PLCE1*

rs8063355	*PLCG2*	rs901528	*PLCZ1*	rs1048257	*PLD4*	rs829232	*PLCE1*
rs8063604	*PLCG2*	rs969489	*PLCZ1*	rs2819419	*PLD4*	rs10882378	*PLCE1*
rs8063813	*PLCG2*	rs1048257	*PLD4*	rs2841277	*PLD4*	rs1223583	*PLCE1*
rs9928191	*PLCG2*	rs2819419	*PLD4*	rs3001421	*PLD4*	rs1223585	*PLCE1*
rs9932716	*PLCG2*	rs2841277	*PLD4*	rs2245715	*PPIP5K1*	rs10882380	*PLCE1*
rs9937223	*PLCG2*	rs3001421	*PLD4*	rs2251844	*PPIP5K1*	rs1776842	*PLCE1*
rs9937704	*PLCG2*	rs2245715	*PPIP5K1*	rs2255042	*PPIP5K1*	rs10882381	*PLCE1*
rs9938623	*PLCG2*	rs2251844	*PPIP5K1*	rs2255663	*PPIP5K1*	rs1935960	*PLCE1*
rs10513478	*PLCH1*	rs2255042	*PPIP5K1*	rs496584	*PPIP5K1*	rs1935961	*PLCE1*
rs1125539	*PLCH1*	rs2255663	*PPIP5K1*	rs689797	*PPIP5K1*	rs2182093	*PLCE1*
rs13085233	*PLCH1*	rs496584	*PPIP5K1*	rs694985	*PPIP5K1*	rs1925243	*PLCE1*
rs1850847	*PLCH1*	rs689797	*PPIP5K1*	rs183752	*PPIP5K2*	rs2209442	*PLCE1*
rs359565	*PLCH1*	rs694985	*PPIP5K1*	rs246912	*PPIP5K2*	rs4918070	*PLCE1*
rs359570	*PLCH1*	rs183752	*PPIP5K2*	rs26258	*PPIP5K2*	rs6583926	*PLCE1*
rs3851357	*PLCH1*	rs246912	*PPIP5K2*	rs26521	*PPIP5K2*	rs4918082	*PLCE1*
rs517971	*PLCH1*	rs26258	*PPIP5K2*	rs26819	*PPIP5K2*	rs11187789	*PLCE1*
rs592982	*PLCH1*	rs26521	*PPIP5K2*	rs26821	*PPIP5K2*	rs17416616	*PLCE1*
rs6440997	*PLCH1*	rs26819	*PPIP5K2*	rs34813	*PPIP5K2*	rs1998709	*PLCE1*
rs6790399	*PLCH1*	rs26821	*PPIP5K2*	rs10509532	*PTEN*	rs10786152	*PLCE1*
rs7629025	*PLCH1*	rs34813	*PPIP5K2*	rs10887758	*PTEN*	rs1776946	*PLCE1*
rs7630469	*PLCH1*	rs10509532	*PTEN*	rs11202607	*PTEN*	rs2689694	*PLCE1*
rs7637342	*PLCH1*	rs10887758	*PTEN*	rs1234212	*PTEN*	rs2689700	*PLCE1*
rs9289957	*PLCH1*	rs11202607	*PTEN*	rs1234220	*PTEN*	rs11187808	*PLCE1*
rs934591	*PLCH1*	rs1234212	*PTEN*	rs1234221	*PTEN*	rs2689698	*PLCE1*
rs10910078	*PLCH2*	rs1234220	*PTEN*	rs2248293	*PTEN*	rs4545470	*PLCE1*
rs11588930	*PLCH2*	rs1234221	*PTEN*	rs2299939	*PTEN*	rs2797998	*PLCE1*
rs12049628	*PLCH2*	rs2248293	*PTEN*	rs478839	*PTEN*	rs10786155	*PLCE1*
rs13376356	*PLCH2*	rs2299939	*PTEN*	rs10470165	*SYNJ1*	rs2689693	*PLCE1*
rs17373634	*PLCH2*	rs478839	*PTEN*	rs11702774	*SYNJ1*	rs2860746	*PLCE1*
rs2236395	*PLCH2*	rs11702774	*SYNJ1*	rs1783099	*SYNJ1*	rs7908334	*PLCE1*
rs2477703	*PLCH2*	rs1783099	*SYNJ1*	rs2254562	*SYNJ1*	rs2798001	*PLCE1*
rs2494626	*PLCH2*	rs2254562	*SYNJ1*	rs582547	*SYNJ1*	rs1858608	*PLCE1*
rs3762444	*PLCH2*	rs582547	*SYNJ1*	rs632324	*SYNJ1*	rs11187815	*PLCE1*
rs7535528	*PLCH2*	rs632324	*SYNJ1*	rs648648	*SYNJ1*	rs12769135	*PLCE1*
rs1021266	*PLCZ1*	rs648648	*SYNJ1*	rs7279487	*SYNJ1*	rs10882406	*PLCE1*
rs1021267	*PLCZ1*	rs7279487	*SYNJ1*	rs845016	*SYNJ1*	rs11187825	*PLCE1*
rs1027032	*PLCZ1*	rs845016	*SYNJ1*	rs845018	*SYNJ1*	rs2226170	*PLCE1*
rs10505831	*PLCZ1*	rs845018	*SYNJ1*	rs1009014	*SYNJ2*	rs11187828	*PLCE1*
rs10841075	*PLCZ1*	rs1009014	*SYNJ2*	rs10455936	*SYNJ2*	rs10882412	*PLCE1*
rs10841077	*PLCZ1*	rs10455936	*SYNJ2*	rs10806791	*SYNJ2*	rs17109869	*PLCE1*
rs11044268	*PLCZ1*	rs10806791	*SYNJ2*	rs12202135	*SYNJ2*	rs4917450	*PLCE1*
rs11833512	*PLCZ1*	rs12202135	*SYNJ2*	rs12663163	*SYNJ2*	rs7919066	*PLCE1*
rs1386398	*PLCZ1*	rs12663163	*SYNJ2*	rs16900563	*SYNJ2*	rs1547643	*PLCE1*
rs1471891	*PLCZ1*	rs16900563	*SYNJ2*	rs1744169	*SYNJ2*	rs1408820	*PLCE1*

rs1550990	*PLCZ1*	rs1744169	*SYNJ2*	rs1744173	*SYNJ2*	rs3740360	*PLCE1*
rs2306798	*PLCZ1*	rs1744173	*SYNJ2*	rs1750043	*SYNJ2*	rs4918188	*PLCE1*
rs4764417	*PLCZ1*	rs1750043	*SYNJ2*	rs1977356	*SYNJ2*	rs12263737	*PLCE1*
rs513426	*PLCZ1*	rs1977356	*SYNJ2*	rs2025641	*SYNJ2*	rs753724	*PLCE1*
rs6486914	*PLCZ1*	rs2025641	*SYNJ2*	rs2181190	*SYNJ2*	rs11187842	*PLCE1*
rs665197	*PLCZ1*	rs2181190	*SYNJ2*	rs2295893	*SYNJ2*	rs3765524	*PLCE1*
rs7485517	*PLCZ1*	rs2295893	*SYNJ2*	rs2295894	*SYNJ2*	rs12766693	*PLCE1*
rs7972408	*PLCZ1*	rs2295894	*SYNJ2*	rs2475556	*SYNJ2*	rs4394764	*PLCE1*
rs7974908	*PLCZ1*	rs2475556	*SYNJ2*	rs2502601	*SYNJ2*	rs2274223	*PLCE1*
rs901528	*PLCZ1*	rs2502601	*SYNJ2*	rs2502618	*SYNJ2*	rs17516904	*PLCE1*
rs1048257	*PLD4*	rs2502618	*SYNJ2*	rs2502620	*SYNJ2*	rs3781264	*PLCE1*
rs1595947	*PLD4*	rs2502620	*SYNJ2*	rs2885911	*SYNJ2*	rs2077218	*PLCE1*
rs2819419	*PLD4*	rs2885911	*SYNJ2*	rs350289	*SYNJ2*	rs17109928	*PLCE1*
rs2841277	*PLD4*	rs350289	*SYNJ2*	rs3818457	*SYNJ2*	rs17109928	*PLCE1*
rs3001421	*PLD4*	rs3818457	*SYNJ2*	rs4333441	*SYNJ2*	rs6129760	*PLCG1*
rs12912505	*PPIP5K1*	rs4333441	*SYNJ2*	rs6455990	*SYNJ2*	rs12624863	*PLCG1*
rs2245715	*PPIP5K1*	rs6455990	*SYNJ2*	rs750997	*SYNJ2*	rs2228246	*PLCG1*
rs2251844	*PPIP5K1*	rs750997	*SYNJ2*	rs751873	*SYNJ2*	rs753381	*PLCG1*
rs2255042	*PPIP5K1*	rs751873	*SYNJ2*	rs7768038	*SYNJ2*	rs2235360	*PLCG1*
rs2255663	*PPIP5K1*	rs7768038	*SYNJ2*	rs9356200	*SYNJ2*	rs2235366	*PLCG1*
rs496584	*PPIP5K1*	rs9356200	*SYNJ2*	rs9365674	*SYNJ2*	rs2664537	*PLCG1*
rs689797	*PPIP5K1*	rs9365674	*SYNJ2*	rs9365723	*SYNJ2*	rs2664537	*PLCG1*
rs689931	*PPIP5K1*	rs9365723	*SYNJ2*	rs9365724	*SYNJ2*	rs8063120	*PLCG2*
rs694985	*PPIP5K1*	rs9365724	*SYNJ2*	rs9458975	*SYNJ2*	rs9937704	*PLCG2*
rs11744885	*PPIP5K2*	rs9458975	*SYNJ2*	rs9459056	*SYNJ2*	rs10445097	*PLCG2*
rs183752	*PPIP5K2*	rs9459056	*SYNJ2*	rs9459093	*SYNJ2*	rs4328435	*PLCG2*
rs246912	*PPIP5K2*	rs9459093	*SYNJ2*	rs9459154	*SYNJ2*	rs6564915	*PLCG2*
rs246916	*PPIP5K2*	rs9459154	*SYNJ2*	rs9654570	*SYNJ2*	rs4254322	*PLCG2*
rs26258	*PPIP5K2*	rs9654570	*SYNJ2*	rs999613	*SYNJ2*	rs4398100	*PLCG2*
rs26521	*PPIP5K2*	rs999613	*SYNJ2*	rs2238114	*TPI1*	rs4580153	*PLCG2*
rs26819	*PPIP5K2*	rs2238114	*TPI1*			rs4580154	*PLCG2*
rs26821	*PPIP5K2*					rs4243211	*PLCG2*
rs34813	*PPIP5K2*					rs12598194	*PLCG2*
rs1022427	*PTEN*					rs12448334	*PLCG2*
rs10887758	*PTEN*					rs4889384	*PLCG2*
rs11202596	*PTEN*					rs12599264	*PLCG2*
rs11202607	*PTEN*					rs4889393	*PLCG2*
rs1234212	*PTEN*					rs9937223	*PLCG2*
rs1234220	*PTEN*					rs4456499	*PLCG2*
rs1234221	*PTEN*					rs6420427	*PLCG2*
rs2248293	*PTEN*					rs4405545	*PLCG2*
rs2299939	*PTEN*					rs7499275	*PLCG2*
rs11702774	*SYNJ1*					rs7194131	*PLCG2*
rs17694546	*SYNJ1*					rs4325546	*PLCG2*

rs1783099	*SYNJ1*	rs8043593	*PLCG2*
rs2254562	*SYNJ1*	rs4889411	*PLCG2*
rs582547	*SYNJ1*	rs4888179	*PLCG2*
rs648648	*SYNJ1*	rs7202205	*PLCG2*
rs7279487	*SYNJ1*	rs4072683	*PLCG2*
rs844996	*SYNJ1*	rs7185362	*PLCG2*
rs845016	*SYNJ1*	rs4074445	*PLCG2*
rs845018	*SYNJ1*	rs4888181	*PLCG2*
rs1009014	*SYNJ2*	rs12596639	*PLCG2*
rs10455936	*SYNJ2*	rs11643875	*PLCG2*
rs10806791	*SYNJ2*	rs4243218	*PLCG2*
rs12202135	*SYNJ2*	rs3935877	*PLCG2*
rs12208248	*SYNJ2*	rs4889422	*PLCG2*
rs12663163	*SYNJ2*	rs7342694	*PLCG2*
rs13217929	*SYNJ2*	rs11644646	*PLCG2*
rs1744169	*SYNJ2*	rs4997772	*PLCG2*
rs1744173	*SYNJ2*	rs4888184	*PLCG2*
rs1744178	*SYNJ2*	rs9932716	*PLCG2*
rs17489570	*SYNJ2*	rs3935743	*PLCG2*
rs1750040	*SYNJ2*	rs7197601	*PLCG2*
rs1750043	*SYNJ2*	rs11644436	*PLCG2*
rs1977356	*SYNJ2*	rs8062633	*PLCG2*
rs2025641	*SYNJ2*	rs12446070	*PLCG2*
rs2181190	*SYNJ2*	rs8063355	*PLCG2*
rs2295893	*SYNJ2*	rs4133125	*PLCG2*
rs2295894	*SYNJ2*	rs4133124	*PLCG2*
rs2502601	*SYNJ2*	rs8055576	*PLCG2*
rs2502618	*SYNJ2*	rs4889425	*PLCG2*
rs2502620	*SYNJ2*	rs4889426	*PLCG2*
rs350289	*SYNJ2*	rs4889428	*PLCG2*
rs350292	*SYNJ2*	rs4889432	*PLCG2*
rs3818457	*SYNJ2*	rs4889436	*PLCG2*
rs4333441	*SYNJ2*	rs4243221	*PLCG2*
rs6455990	*SYNJ2*	rs11859107	*PLCG2*
rs750997	*SYNJ2*	rs4369658	*PLCG2*
rs751873	*SYNJ2*	rs4306504	*PLCG2*
rs7768038	*SYNJ2*	rs11864701	*PLCG2*
rs9295289	*SYNJ2*	rs12448055	*PLCG2*
rs9356200	*SYNJ2*	rs3922849	*PLCG2*
rs9365674	*SYNJ2*	rs13331678	*PLCG2*
rs9365723	*SYNJ2*	rs7203619	*PLCG2*
rs9365724	*SYNJ2*	rs9938623	*PLCG2*
rs9458975	*SYNJ2*	rs7201045	*PLCG2*
rs9459056	*SYNJ2*	rs8063604	*PLCG2*

rs9459093	*SYNJ2*
rs9459154	*SYNJ2*
rs9654570	*SYNJ2*
rs999613	*SYNJ2*
rs10744720	*TPI1*
rs2238114	*TPI1*
rs4304152	*IP6K3*
rs1536500	*IP6K3*
rs6919321	*IP6K3*
rs9380376	*IP6K3*
rs12211490	*IP6K3*
rs1536501	*IP6K3*
rs4711345	*IP6K3*
rs755495	*IP6K3*
rs4711348	*IP6K3*
rs6904716	*IP6K3*
rs6933607	*IP6K3*
rs17318874	*MTM1*
rs5925269	*MTM1*
rs490733	*MTM1*
rs12557844	*MTM1*
rs10521887	*MTM1*
rs522599	*MTM1*
rs222383	*MTM1*
rs5924834	*MTM1*
rs5925391	*MTM1*
rs222347	*MTM1*
rs222358	*MTM1*
rs222364	*MTM1*
rs1533423	*MTM1*
rs5925397	*MTM1*
rs5925398	*MTM1*
rs222410	*MTM1*
rs222417	*MTM1*
rs222418	*MTM1*
rs222420	*MTM1*
rs5925403	*MTM1*
rs6584138	*PI4K2A*
rs11189310	*PI4K2A*
rs3890727	*PI4K2A*
rs4919128	*PI4K2A*
rs2065672	*PI4K2A*
rs11189321	*PI4K2A*
rs10444068	*PI4K2A*
rs12446596	*PLCG2*
rs6564940	*PLCG2*
rs7187863	*PLCG2*
rs7499440	*PLCG2*
rs7500286	*PLCG2*
rs12598402	*PLCG2*
rs8043619	*PLCG2*
rs4073828	*PLCG2*
rs3936112	*PLCG2*
rs4369659	*PLCG2*
rs4888191	*PLCG2*
rs12918369	*PLCG2*
rs4405546	*PLCG2*
rs4889444	*PLCG2*
rs10514519	*PLCG2*
rs11862662	*PLCG2*
rs16956040	*PLCG2*
rs4611452	*PLCG2*
rs17203310	*PLCG2*
rs4508413	*PLCG2*
rs12716928	*PLCG2*
rs12921780	*PLCG2*
rs8056564	*PLCG2*
rs8055043	*PLCG2*
rs4284633	*PLCG2*
rs4312298	*PLCG2*
rs4603554	*PLCG2*
rs8063813	*PLCG2*
rs8047356	*PLCG2*
rs4888197	*PLCG2*
rs4889448	*PLCG2*
rs9928191	*PLCG2*
rs17793122	*PLCG2*
rs4286103	*PLCG2*
rs4243226	*PLCG2*
rs9938835	*PLCG2*
rs4888201	*PLCG2*
rs4888201	*PLCG2*
rs1125539	*PLCH1*
rs934591	*PLCH1*
rs6790399	*PLCH1*
rs7637342	*PLCH1*
rs10513478	*PLCH1*
rs6440997	*PLCH1*

rs10786364	*PI4K2A*	rs592982	*PLCH1*
rs3115231	*PI4K2B*	rs3851357	*PLCH1*
rs313548	*PI4K2B*	rs517971	*PLCH1*
rs7661189	*PI4K2B*	rs7630469	*PLCH1*
rs313566	*PI4K2B*	rs1850847	*PLCH1*
rs313541	*PI4K2B*	rs9289957	*PLCH1*
rs313533	*PI4K2B*	rs359565	*PLCH1*
rs3796780	*PI4K2B*	rs359570	*PLCH1*
rs6435435	*PIKFYVE*	rs7629025	*PLCH1*
rs3769521	*PIKFYVE*	rs10910078	*PLCH2*
rs17652774	*PIKFYVE*	rs2494626	*PLCH2*
rs6746926	*PIKFYVE*	rs13376356	*PLCH2*
rs2044457	*PIKFYVE*	rs11588930	*PLCH2*
rs1584200	*PIKFYVE*	rs12049628	*PLCH2*
rs7569723	*PIKFYVE*	rs17373634	*PLCH2*
rs10189031	*PIKFYVE*	rs2477703	*PLCH2*
rs999890	*PIKFYVE*	rs3762444	*PLCH2*
rs4675754	*PIKFYVE*	rs7535528	*PLCH2*
rs1866046	*PIKFYVE*	rs2236395	*PLCH2*
rs2289171	*PIKFYVE*	rs513426	*PLCZ1*
rs6435450	*PIKFYVE*	rs665197	*PLCZ1*
rs10190458	*PIKFYVE*	rs1550990	*PLCZ1*
rs2118295	*PIKFYVE*	rs1386398	*PLCZ1*
rs13407268	*PIKFYVE*	rs2306798	*PLCZ1*
rs1465804	*PIKFYVE*	rs11833512	*PLCZ1*
rs2118297	*PIKFYVE*	rs4764417	*PLCZ1*
rs994696	*PIKFYVE*	rs1471891	*PLCZ1*
rs10177810	*PIKFYVE*	rs901528	*PLCZ1*
rs6435453	*PIKFYVE*	rs10841075	*PLCZ1*
rs10208191	*PIKFYVE*	rs10841077	*PLCZ1*
rs9646839	*PIKFYVE*	rs7974908	*PLCZ1*
rs4673402	*PIKFYVE*	rs1021266	*PLCZ1*
rs4675764	*PIKFYVE*	rs1021267	*PLCZ1*
rs4836593	*PIP5KL1*	rs6486914	*PLCZ1*
rs3739821	*PIP5KL1*	rs11044268	*PLCZ1*
rs7859	*PIP5KL1*	rs10505831	*PLCZ1*
rs6478803	*PIP5KL1*	rs1027032	*PLCZ1*
rs694985	*PPIP5K1*	rs7972408	*PLCZ1*
rs12912505	*PPIP5K1*	rs7485517	*PLCZ1*
rs2245715	*PPIP5K1*	rs17488409	*PLCZ1*
rs689931	*PPIP5K1*	rs969489	*PLCZ1*
rs689797	*PPIP5K1*	rs969489	*PLCZ1*
rs2255663	*PPIP5K1*	rs1595947	*PLD4*
rs496584	*PPIP5K1*	rs3001421	*PLD4*

rs2255042	*PPIP5K1*
rs2251844	*PPIP5K1*
rs34813	*PPIP5K2*
rs26521	*PPIP5K2*
rs28043	*PPIP5K2*
rs183752	*PPIP5K2*
rs26821	*PPIP5K2*
rs26819	*PPIP5K2*
rs11744885	*PPIP5K2*
rs26258	*PPIP5K2*
rs246916	*PPIP5K2*
rs246912	*PPIP5K2*

rs2841277	*PLD4*
rs1048257	*PLD4*
rs694985	*PPIP5K1*
rs12912505	*PPIP5K1*
rs2245715	*PPIP5K1*
rs689931	*PPIP5K1*
rs689797	*PPIP5K1*
rs2255663	*PPIP5K1*
rs496584	*PPIP5K1*
rs2255042	*PPIP5K1*
rs2251844	*PPIP5K1*
rs34813	*PPIP5K2*
rs26521	*PPIP5K2*
rs183752	*PPIP5K2*
rs26821	*PPIP5K2*
rs26819	*PPIP5K2*
rs11744885	*PPIP5K2*
rs26258	*PPIP5K2*
rs246916	*PPIP5K2*
rs246912	*PPIP5K2*
rs246912	*PPIP5K2*
rs10887758	*PTEN*
rs1022427	*PTEN*
rs1234212	*PTEN*
rs1234221	*PTEN*
rs1234220	*PTEN*
rs11202596	*PTEN*
rs2299939	*PTEN*
rs2248293	*PTEN*
rs11202607	*PTEN*
rs478839	*PTEN*
rs10509532	*PTEN*
rs10509532	*PTEN*
rs845016	*SYNJ1*
rs845018	*SYNJ1*
rs7279487	*SYNJ1*
rs11702774	*SYNJ1*
rs10470165	*SYNJ1*
rs17694546	*SYNJ1*
rs844996	*SYNJ1*
rs2254562	*SYNJ1*
rs1783099	*SYNJ1*
rs582547	*SYNJ1*
rs648648	*SYNJ1*

rs632324	*SYNJ1*
rs12626242	*SYNJ1*
rs12626242	*SYNJ1*
rs9654570	*SYNJ2*
rs9458975	*SYNJ2*
rs12663163	*SYNJ2*
rs2025641	*SYNJ2*
rs9365674	*SYNJ2*
rs9459056	*SYNJ2*
rs10455936	*SYNJ2*
rs10806791	*SYNJ2*
rs9356200	*SYNJ2*
rs12202135	*SYNJ2*
rs9459093	*SYNJ2*
rs9365723	*SYNJ2*
rs9365724	*SYNJ2*
rs9459154	*SYNJ2*
rs999613	*SYNJ2*
rs7768038	*SYNJ2*
rs2295893	*SYNJ2*
rs2295894	*SYNJ2*
rs1750043	*SYNJ2*
rs1977356	*SYNJ2*
rs9295289	*SYNJ2*
rs750997	*SYNJ2*
rs2502620	*SYNJ2*
rs2502618	*SYNJ2*
rs1009014	*SYNJ2*
rs17489570	*SYNJ2*
rs751873	*SYNJ2*
rs4333441	*SYNJ2*
rs6455990	*SYNJ2*
rs1744178	*SYNJ2*
rs1750040	*SYNJ2*
rs3818457	*SYNJ2*
rs350292	*SYNJ2*
rs12208248	*SYNJ2*
rs1744173	*SYNJ2*
rs1744169	*SYNJ2*
rs350289	*SYNJ2*
rs13217929	*SYNJ2*
rs2502601	*SYNJ2*
rs2475556	*SYNJ2*
rs2475556	*SYNJ2*

						rs10744720	*TPI1*
						rs2238114	*TPI1*
						rs2238114	*TPI1*

**Pancreatic cancer**		**Breast cancer**		**Prostate cancer**		**Bladder cancer**	
SNP	*Gene*	SNP	*Gene*	SNP	*Gene*	SNP	*Gene*
rs3742809	*ALDH6A1*	rs17096208	*ALDH6A1*	rs3742809	*ALDH6A1*	rs2072294	*ALDH6A1*
rs2072294	*ALDH6A1*	rs2072294	*ALDH6A1*	rs2072294	*ALDH6A1*	rs7144433	*CALM1*
rs765719	*ALDH6A1*	rs2300193	*ALDH6A1*	rs765719	*ALDH6A1*	rs8006462	*CALM1*
rs17096208	*ALDH6A1*	rs3742809	*ALDH6A1*	rs17096208	*ALDH6A1*	rs10865222	*CALM2*
rs2300193	*ALDH6A1*	rs765719	*ALDH6A1*	rs2300193	*ALDH6A1*	rs13410472	*CALM2*
rs6575129	*CALM1*	rs1058903	*CALM1*	rs2300193	*ALDH6A1*	rs17036320	*CALM2*
rs7144433	*CALM1*	rs2300497	*CALM1*	rs6575129	*CALM1*	rs815802	*CALM2*
rs2300497	*CALM1*	rs2300502	*CALM1*	rs7144433	*CALM1*	rs314669	*CALM3*
rs2300502	*CALM1*	rs3213718	*CALM1*	rs2300497	*CALM1*	rs8113456	*CALM3*
rs3213718	*CALM1*	rs5871	*CALM1*	rs2300502	*CALM1*	rs13331733	*CDIPT*
rs5871	*CALM1*	rs6575129	*CALM1*	rs3213718	*CALM1*	rs8047140	*CDIPT*
rs1058903	*CALM1*	rs7144433	*CALM1*	rs5871	*CALM1*	rs16976956	*IMPA2*
rs8006462	*CALM1*	rs8006462	*CALM1*	rs1058903	*CALM1*	rs17593321	*IMPA2*
rs8006462	*CALM1*	rs10865222	*CALM2*	rs8006462	*CALM1*	rs2360082	*IMPA2*
rs4953470	*CALM2*	rs13410472	*CALM2*	rs4953470	*CALM2*	rs613993	*IMPA2*
rs815808	*CALM2*	rs17036320	*CALM2*	rs815808	*CALM2*	rs638063	*IMPA2*
rs815804	*CALM2*	rs4953470	*CALM2*	rs815804	*CALM2*	rs647077	*IMPA2*
rs10865222	*CALM2*	rs7581908	*CALM2*	rs10865222	*CALM2*	rs6505700	*IMPA2*
rs17036320	*CALM2*	rs815802	*CALM2*	rs17036320	*CALM2*	rs7244678	*IMPA2*
rs815802	*CALM2*	rs815804	*CALM2*	rs815802	*CALM2*	rs9955952	*IMPA2*
rs815815	*CALM2*	rs815808	*CALM2*	rs13410472	*CALM2*	rs9973072	*IMPA2*
rs13410472	*CALM2*	rs10113	*CALM3*	rs7581908	*CALM2*	rs13257046	*IMPAD1*
rs7581908	*CALM2*	rs11083841	*CALM3*	rs7258489	*CALM3*	rs4738558	*IMPAD1*
rs9808216	*CALM2*	rs1126510	*CALM3*	rs7259810	*CALM3*	rs10931450	*INPP1*
rs9808216	*CALM2*	rs4274528	*CALM3*	rs10113	*CALM3*	rs2067417	*INPP1*
rs4274528	*CALM3*	rs7258489	*CALM3*	rs11083841	*CALM3*	rs2067418	*INPP1*
rs7258489	*CALM3*	rs7259810	*CALM3*	rs1126510	*CALM3*	rs2067434	*INPP1*
rs7259810	*CALM3*	rs12917712	*CDIPT*	rs3815822	*CDIPT*	rs909270	*INPP1*
rs10113	*CALM3*	rs3815822	*CDIPT*	rs12917712	*CDIPT*	rs11676357	*INPP4A*
rs11083841	*CALM3*	rs4787483	*CDIPT*	rs4787483	*CDIPT*	rs17504837	*INPP4A*
rs1126510	*CALM3*	rs1967328	*IMPA1*	rs4787483	*CDIPT*	rs2278214	*INPP4A*
rs1126510	*CALM3*	rs2912821	*IMPA1*	rs1967328	*IMPA1*	rs3754876	*INPP4A*
rs3815822	*CDIPT*	rs2955005	*IMPA1*	rs2912821	*IMPA1*	rs10013734	*INPP4B*
rs12917712	*CDIPT*	rs1020294	*IMPA2*	rs2955005	*IMPA1*	rs10020322	*INPP4B*
rs4787483	*CDIPT*	rs1250171	*IMPA2*	rs679246	*IMPA2*	rs1017527	*INPP4B*
rs2912821	*IMPA1*	rs1262056	*IMPA2*	rs1787984	*IMPA2*	rs1219274	*INPP4B*
rs2955005	*IMPA1*	rs16976931	*IMPA2*	rs662383	*IMPA2*	rs13109869	*INPP4B*
rs2955005	*IMPA1*	rs16976956	*IMPA2*	rs3786305	*IMPA2*	rs13119090	*INPP4B*
rs679246	*IMPA2*	rs17593321	*IMPA2*	rs647077	*IMPA2*	rs13133181	*INPP4B*

rs1787984	*IMPA2*	rs1787984	*IMPA2*	rs2360082	*IMPA2*	rs13148456	*INPP4B*
rs662383	*IMPA2*	rs2002212	*IMPA2*	rs7506045	*IMPA2*	rs1391099	*INPP4B*
rs3786305	*IMPA2*	rs2360082	*IMPA2*	rs6505700	*IMPA2*	rs1425520	*INPP4B*
rs647077	*IMPA2*	rs3786284	*IMPA2*	rs663591	*IMPA2*	rs1443187	*INPP4B*
rs2360082	*IMPA2*	rs3786305	*IMPA2*	rs638063	*IMPA2*	rs1489577	*INPP4B*
rs7506045	*IMPA2*	rs613993	*IMPA2*	rs9973072	*IMPA2*	rs17015594	*INPP4B*
rs6505700	*IMPA2*	rs628419	*IMPA2*	rs684680	*IMPA2*	rs17015754	*INPP4B*
rs663591	*IMPA2*	rs630110	*IMPA2*	rs636173	*IMPA2*	rs17015882	*INPP4B*
rs638063	*IMPA2*	rs636173	*IMPA2*	rs7244678	*IMPA2*	rs17015920	*INPP4B*
rs9973072	*IMPA2*	rs638063	*IMPA2*	rs16976931	*IMPA2*	rs17016027	*INPP4B*
rs684680	*IMPA2*	rs647077	*IMPA2*	rs17593321	*IMPA2*	rs17380914	*INPP4B*
rs636173	*IMPA2*	rs6505700	*IMPA2*	rs3786284	*IMPA2*	rs1907108	*INPP4B*
rs7244678	*IMPA2*	rs662383	*IMPA2*	rs7235476	*IMPA2*	rs2165819	*INPP4B*
rs16976931	*IMPA2*	rs663591	*IMPA2*	rs16976956	*IMPA2*	rs2627804	*INPP4B*
rs17593321	*IMPA2*	rs679246	*IMPA2*	rs628419	*IMPA2*	rs2627808	*INPP4B*
rs3786284	*IMPA2*	rs684680	*IMPA2*	rs1020294	*IMPA2*	rs2636660	*INPP4B*
rs7235476	*IMPA2*	rs7235476	*IMPA2*	rs1262056	*IMPA2*	rs2636670	*INPP4B*
rs16976956	*IMPA2*	rs7244678	*IMPA2*	rs1250171	*IMPA2*	rs2636683	*INPP4B*
rs1020294	*IMPA2*	rs7506045	*IMPA2*	rs613993	*IMPA2*	rs2667100	*INPP4B*
rs1262056	*IMPA2*	rs9955952	*IMPA2*	rs630110	*IMPA2*	rs2874870	*INPP4B*
rs1250171	*IMPA2*	rs9973072	*IMPA2*	rs9955952	*IMPA2*	rs331941	*INPP4B*
rs613993	*IMPA2*	rs1044731	*IMPAD1*	rs9955952	*IMPA2*	rs336298	*INPP4B*
rs630110	*IMPA2*	rs13257046	*IMPAD1*	rs1508088	*IMPAD1*	rs336347	*INPP4B*
rs9955952	*IMPA2*	rs4738558	*IMPAD1*	rs6474091	*IMPAD1*	rs336361	*INPP4B*
rs6474091	*IMPAD1*	rs6997875	*IMPAD1*	rs8718	*IMPAD1*	rs336394	*INPP4B*
rs8718	*IMPAD1*	rs8718	*IMPAD1*	rs1044731	*IMPAD1*	rs336407	*INPP4B*
rs1044731	*IMPAD1*	rs10931450	*INPP1*	rs4738558	*IMPAD1*	rs336408	*INPP4B*
rs4738558	*IMPAD1*	rs1108939	*INPP1*	rs13257046	*IMPAD1*	rs3756125	*INPP4B*
rs13257046	*IMPAD1*	rs1866852	*INPP1*	rs6997875	*IMPAD1*	rs3775601	*INPP4B*
rs6997875	*IMPAD1*	rs2067416	*INPP1*	rs291429	*INPP1*	rs3775671	*INPP4B*
rs6997875	*IMPAD1*	rs2067417	*INPP1*	rs3791809	*INPP1*	rs3775696	*INPP4B*
rs291429	*INPP1*	rs2067418	*INPP1*	rs2067416	*INPP1*	rs3822135	*INPP4B*
rs3791809	*INPP1*	rs2067434	*INPP1*	rs2067417	*INPP1*	rs3822152	*INPP4B*
rs2067416	*INPP1*	rs2736619	*INPP1*	rs2067418	*INPP1*	rs4690699	*INPP4B*
rs2067417	*INPP1*	rs291429	*INPP1*	rs2067434	*INPP1*	rs6820463	*INPP4B*
rs2067418	*INPP1*	rs3791809	*INPP1*	rs10931450	*INPP1*	rs6837163	*INPP4B*
rs2067434	*INPP1*	rs909270	*INPP1*	rs909270	*INPP1*	rs6842783	*INPP4B*
rs10931450	*INPP1*	rs11676357	*INPP4A*	rs1108939	*INPP1*	rs6846839	*INPP4B*
rs909270	*INPP1*	rs17504837	*INPP4A*	rs2736619	*INPP1*	rs6847049	*INPP4B*
rs1108939	*INPP1*	rs222	*INPP4A*	rs1866852	*INPP1*	rs713071	*INPP4B*
rs2736619	*INPP1*	rs2278206	*INPP4A*	rs3820947	*INPP4A*	rs7688435	*INPP4B*
rs1866852	*INPP1*	rs2278210	*INPP4A*	rs3754886	*INPP4A*	rs966457	*INPP4B*
rs1866852	*INPP1*	rs2278214	*INPP4A*	rs11676357	*INPP4A*	rs11146487	*INPP5A*
rs3820947	*INPP4A*	rs3754876	*INPP4A*	rs2278214	*INPP4A*	rs12267364	*INPP5A*

rs3754886	*INPP4A*	rs3754886	*INPP4A*	rs17504837	*INPP4A*	rs2492768	*INPP5A*
rs11676357	*INPP4A*	rs3820947	*INPP4A*	rs222	*INPP4A*	rs2737398	*INPP5A*
rs2278214	*INPP4A*	rs6734569	*INPP4A*	rs2278210	*INPP4A*	rs4272721	*INPP5A*
rs17504837	*INPP4A*	rs10000770	*INPP4B*	rs6734569	*INPP4A*	rs7084105	*INPP5A*
rs222	*INPP4A*	rs10009093	*INPP4B*	rs2278206	*INPP4A*	rs7903076	*INPP5A*
rs2278210	*INPP4A*	rs10013734	*INPP4B*	rs3754876	*INPP4A*	rs7907513	*INPP5A*
rs6734569	*INPP4A*	rs10020322	*INPP4B*	rs2457997	*INPP4B*	rs7912273	*INPP5A*
rs2278206	*INPP4A*	rs10020507	*INPP4B*	rs17380914	*INPP4B*	rs7914354	*INPP5A*
rs3754876	*INPP4A*	rs1017527	*INPP4B*	rs11736202	*INPP4B*	rs873946	*INPP5A*
rs3754876	*INPP4A*	rs10519631	*INPP4B*	rs336361	*INPP4B*	rs913193	*INPP5A*
rs17380914	*INPP4B*	rs10519638	*INPP4B*	rs3775601	*INPP4B*	rs4308943	*INPP5B*
rs11736202	*INPP4B*	rs10519649	*INPP4B*	rs3775605	*INPP4B*	rs7512424	*INPP5B*
rs336361	*INPP4B*	rs10857395	*INPP4B*	rs2667096	*INPP4B*	rs10203185	*INPP5D*
rs3775601	*INPP4B*	rs11736202	*INPP4B*	rs1511252	*INPP4B*	rs10803668	*INPP5D*
rs3775605	*INPP4B*	rs11930321	*INPP4B*	rs2667100	*INPP4B*	rs10933435	*INPP5D*
rs2667096	*INPP4B*	rs11930849	*INPP4B*	rs3775616	*INPP4B*	rs11682728	*INPP5D*
rs1511252	*INPP4B*	rs11934541	*INPP4B*	rs4956433	*INPP4B*	rs12694922	*INPP5D*
rs2667100	*INPP4B*	rs11943687	*INPP4B*	rs3822135	*INPP4B*	rs13021302	*INPP5D*
rs3775616	*INPP4B*	rs1219266	*INPP4B*	rs336298	*INPP4B*	rs13031194	*INPP5D*
rs4956433	*INPP4B*	rs1219274	*INPP4B*	rs17468315	*INPP4B*	rs13385922	*INPP5D*
rs6537101	*INPP4B*	rs1219275	*INPP4B*	rs336308	*INPP4B*	rs3792117	*INPP5D*
rs3822135	*INPP4B*	rs12498546	*INPP4B*	rs336332	*INPP4B*	rs4315512	*INPP5D*
rs336298	*INPP4B*	rs12504681	*INPP4B*	rs6842783	*INPP4B*	rs4439944	*INPP5D*
rs17468315	*INPP4B*	rs13109869	*INPP4B*	rs12498546	*INPP4B*	rs4468807	*INPP5D*
rs336308	*INPP4B*	rs13119090	*INPP4B*	rs978752	*INPP4B*	rs4503982	*INPP5D*
rs336332	*INPP4B*	rs13125853	*INPP4B*	rs3775641	*INPP4B*	rs4603757	*INPP5D*
rs6842783	*INPP4B*	rs13133181	*INPP4B*	rs336407	*INPP4B*	rs4663784	*INPP5D*
rs12498546	*INPP4B*	rs13148456	*INPP4B*	rs336408	*INPP4B*	rs4663834	*INPP5D*
rs978752	*INPP4B*	rs1353624	*INPP4B*	rs13148456	*INPP4B*	rs4973063	*INPP5D*
rs3775641	*INPP4B*	rs1364920	*INPP4B*	rs10519631	*INPP4B*	rs4973599	*INPP5D*
rs336407	*INPP4B*	rs1373036	*INPP4B*	rs3775664	*INPP4B*	rs6431580	*INPP5D*
rs336408	*INPP4B*	rs1390994	*INPP4B*	rs17015594	*INPP4B*	rs6715810	*INPP5D*
rs13148456	*INPP4B*	rs1391099	*INPP4B*	rs336391	*INPP4B*	rs7425956	*INPP5D*
rs10519631	*INPP4B*	rs1425520	*INPP4B*	rs336394	*INPP4B*	rs7566856	*INPP5D*
rs3775664	*INPP4B*	rs1425522	*INPP4B*	rs1219275	*INPP4B*	rs7569837	*INPP5D*
rs17015594	*INPP4B*	rs1425531	*INPP4B*	rs336384	*INPP4B*	rs7608422	*INPP5D*
rs336391	*INPP4B*	rs1425533	*INPP4B*	rs1219266	*INPP4B*	rs1127162	*INPP5E*
rs336394	*INPP4B*	rs1443187	*INPP4B*	rs10013734	*INPP4B*	rs1128877	*INPP5E*
rs1219275	*INPP4B*	rs1476122	*INPP4B*	rs1219274	*INPP4B*	rs1004243	*INPP5J*
rs336384	*INPP4B*	rs1489577	*INPP4B*	rs2636683	*INPP4B*	rs5753463	*INPP5J*
rs1219266	*INPP4B*	rs1489578	*INPP4B*	rs1390994	*INPP4B*	rs5753472	*INPP5J*
rs10013734	*INPP4B*	rs1497391	*INPP4B*	rs2055212	*INPP4B*	rs2270227	*INPP5K*
rs1219274	*INPP4B*	rs1497393	*INPP4B*	rs3775671	*INPP4B*	rs3900852	*INPPL1*
rs2636683	*INPP4B*	rs1497400	*INPP4B*	rs2627804	*INPP4B*	rs9829155	*IP6K1*

rs1390994	*INPP4B*	rs1511252	*INPP4B*	rs17015754	*INPP4B*	rs9855505	*IP6K1*
rs2055212	*INPP4B*	rs17015594	*INPP4B*	rs13109869	*INPP4B*	rs12497850	*IP6K2*
rs3775671	*INPP4B*	rs17015754	*INPP4B*	rs6846839	*INPP4B*	rs990211	*IP6K2*
rs2627804	*INPP4B*	rs17015882	*INPP4B*	rs6847049	*INPP4B*	rs10947435	*IP6K3*
rs17015754	*INPP4B*	rs17015920	*INPP4B*	rs3113511	*INPP4B*	rs12211490	*IP6K3*
rs13109869	*INPP4B*	rs17016027	*INPP4B*	rs2636660	*INPP4B*	rs1536501	*IP6K3*
rs6846839	*INPP4B*	rs17016550	*INPP4B*	rs2636643	*INPP4B*	rs4304152	*IP6K3*
rs6847049	*INPP4B*	rs17380914	*INPP4B*	rs1907134	*INPP4B*	rs4711345	*IP6K3*
rs3113511	*INPP4B*	rs17468315	*INPP4B*	rs2627813	*INPP4B*	rs4711348	*IP6K3*
rs2636660	*INPP4B*	rs1872293	*INPP4B*	rs2636632	*INPP4B*	rs622917	*IP6K3*
rs2636643	*INPP4B*	rs1872297	*INPP4B*	rs1872297	*INPP4B*	rs6933607	*IP6K3*
rs1907134	*INPP4B*	rs1907107	*INPP4B*	rs3822152	*INPP4B*	rs9380374	*IP6K3*
rs2627813	*INPP4B*	rs1907108	*INPP4B*	rs10519638	*INPP4B*	rs11006086	*IPMK*
rs2636632	*INPP4B*	rs1907134	*INPP4B*	rs3775692	*INPP4B*	rs7068428	*IPMK*
rs1872297	*INPP4B*	rs1908968	*INPP4B*	rs17015882	*INPP4B*	rs10992420	*IPPK*
rs3822152	*INPP4B*	rs1982965	*INPP4B*	rs2627808	*INPP4B*	rs13285641	*IPPK*
rs10519638	*INPP4B*	rs1992418	*INPP4B*	rs1907108	*INPP4B*	rs4744143	*IPPK*
rs3775692	*INPP4B*	rs2017146	*INPP4B*	rs3775696	*INPP4B*	rs9969804	*IPPK*
rs17015882	*INPP4B*	rs2055212	*INPP4B*	rs1907107	*INPP4B*	rs11672385	*ISYNA1*
rs2627808	*INPP4B*	rs2165819	*INPP4B*	rs2636638	*INPP4B*	rs16982345	*ISYNA1*
rs1907108	*INPP4B*	rs2457997	*INPP4B*	rs1872293	*INPP4B*	rs10136012	*ITPK1*
rs3775696	*INPP4B*	rs2627804	*INPP4B*	rs2636670	*INPP4B*	rs11625662	*ITPK1*
rs1907107	*INPP4B*	rs2627808	*INPP4B*	rs2636671	*INPP4B*	rs11628021	*ITPK1*
rs2636638	*INPP4B*	rs2627813	*INPP4B*	rs3756121	*INPP4B*	rs12435423	*ITPK1*
rs1872293	*INPP4B*	rs2635429	*INPP4B*	rs960678	*INPP4B*	rs12586382	*ITPK1*
rs2636670	*INPP4B*	rs2636632	*INPP4B*	rs17015920	*INPP4B*	rs12589455	*ITPK1*
rs2636671	*INPP4B*	rs2636638	*INPP4B*	rs2635429	*INPP4B*	rs12895695	*ITPK1*
rs3756121	*INPP4B*	rs2636643	*INPP4B*	rs1908968	*INPP4B*	rs1612612	*ITPK1*
rs960678	*INPP4B*	rs2636660	*INPP4B*	rs716762	*INPP4B*	rs17128706	*ITPK1*
rs17015920	*INPP4B*	rs2636670	*INPP4B*	rs1497393	*INPP4B*	rs2180369	*ITPK1*
rs2635429	*INPP4B*	rs2636671	*INPP4B*	rs1353624	*INPP4B*	rs2402226	*ITPK1*
rs716762	*INPP4B*	rs2636683	*INPP4B*	rs17016027	*INPP4B*	rs3783919	*ITPK1*
rs1497393	*INPP4B*	rs2667096	*INPP4B*	rs11930321	*INPP4B*	rs4586354	*ITPK1*
rs1353624	*INPP4B*	rs2667100	*INPP4B*	rs1982965	*INPP4B*	rs4905029	*ITPK1*
rs17016027	*INPP4B*	rs2874870	*INPP4B*	rs1497400	*INPP4B*	rs2297380	*ITPKA*
rs11930321	*INPP4B*	rs3113511	*INPP4B*	rs1489578	*INPP4B*	rs8023530	*ITPKA*
rs1982965	*INPP4B*	rs331941	*INPP4B*	rs1489577	*INPP4B*	rs1050492	*ITPKB*
rs1497400	*INPP4B*	rs331946	*INPP4B*	rs3775707	*INPP4B*	rs10916019	*ITPKB*
rs1489578	*INPP4B*	rs336298	*INPP4B*	rs6820463	*INPP4B*	rs1144838	*ITPKB*
rs1489577	*INPP4B*	rs336308	*INPP4B*	rs3756125	*INPP4B*	rs12077348	*ITPKB*
rs3775707	*INPP4B*	rs336309	*INPP4B*	rs1497391	*INPP4B*	rs12094617	*ITPKB*
rs6820463	*INPP4B*	rs336317	*INPP4B*	rs3775720	*INPP4B*	rs17522524	*ITPKB*
rs3756125	*INPP4B*	rs336332	*INPP4B*	rs1391099	*INPP4B*	rs3820635	*ITPKB*
rs1497391	*INPP4B*	rs336347	*INPP4B*	rs7668133	*INPP4B*	rs697845	*ITPKB*

rs3775720	*INPP4B*	rs336355	*INPP4B*	rs6837163	*INPP4B*	rs10420685	*ITPKC*
rs1391099	*INPP4B*	rs336361	*INPP4B*	rs713071	*INPP4B*	rs1870087	*ITPKC*
rs7668133	*INPP4B*	rs336384	*INPP4B*	rs1425533	*INPP4B*	rs3865451	*ITPKC*
rs6837163	*INPP4B*	rs336391	*INPP4B*	rs1425531	*INPP4B*	rs140524	*MIOX*
rs713071	*INPP4B*	rs336394	*INPP4B*	rs1364920	*INPP4B*	rs2273257	*MIOX*
rs1425533	*INPP4B*	rs336407	*INPP4B*	rs7691439	*INPP4B*	rs16883137	*NUDT3*
rs1425531	*INPP4B*	rs336408	*INPP4B*	rs975136	*INPP4B*	rs11107008	*NUDT4*
rs1364920	*INPP4B*	rs3756121	*INPP4B*	rs10857395	*INPP4B*	rs17790482	*NUDT4*
rs7691439	*INPP4B*	rs3756125	*INPP4B*	rs10519649	*INPP4B*	rs17837158	*NUDT4*
rs975136	*INPP4B*	rs3775601	*INPP4B*	rs1476122	*INPP4B*	rs7973701	*NUDT4*
rs10857395	*INPP4B*	rs3775605	*INPP4B*	rs1425520	*INPP4B*	rs7977140	*NUDT4*
rs1476122	*INPP4B*	rs3775616	*INPP4B*	rs6814775	*INPP4B*	rs10444068	*PI4K2A*
rs1425520	*INPP4B*	rs3775636	*INPP4B*	rs11943687	*INPP4B*	rs10786364	*PI4K2A*
rs6814775	*INPP4B*	rs3775641	*INPP4B*	rs1425522	*INPP4B*	rs11189321	*PI4K2A*
rs11943687	*INPP4B*	rs3775664	*INPP4B*	rs7688435	*INPP4B*	rs313533	*PI4K2B*
rs1425522	*INPP4B*	rs3775669	*INPP4B*	rs2874870	*INPP4B*	rs165924	*PI4KA*
rs7688435	*INPP4B*	rs3775671	*INPP4B*	rs2017146	*INPP4B*	rs178058	*PI4KA*
rs2874870	*INPP4B*	rs3775692	*INPP4B*	rs7685002	*INPP4B*	rs178070	*PI4KA*
rs2017146	*INPP4B*	rs3775696	*INPP4B*	rs745719	*INPP4B*	rs17820181	*PI4KA*
rs7685002	*INPP4B*	rs3775707	*INPP4B*	rs13125853	*INPP4B*	rs4822606	*PI4KA*
rs745719	*INPP4B*	rs3775720	*INPP4B*	rs13119090	*INPP4B*	rs9608386	*PI4KA*
rs13125853	*INPP4B*	rs3822135	*INPP4B*	rs1017527	*INPP4B*	rs1752382	*PI4KB*
rs1017527	*INPP4B*	rs3822152	*INPP4B*	rs10020322	*INPP4B*	rs2031797	*PI4KB*
rs10020322	*INPP4B*	rs4690699	*INPP4B*	rs13133181	*INPP4B*	rs4971030	*PI4KB*
rs13133181	*INPP4B*	rs4690728	*INPP4B*	rs6830886	*INPP4B*	rs3950680	*PIK3C2A*
rs6830886	*INPP4B*	rs4956433	*INPP4B*	rs10000770	*INPP4B*	rs11240748	*PIK3C2B*
rs10000770	*INPP4B*	rs6537129	*INPP4B*	rs1373036	*INPP4B*	rs12031854	*PIK3C2B*
rs11943397	*INPP4B*	rs6814775	*INPP4B*	rs1443187	*INPP4B*	rs12119503	*PIK3C2B*
rs1373036	*INPP4B*	rs6820463	*INPP4B*	rs4690699	*INPP4B*	rs17334387	*PIK3C2B*
rs11942593	*INPP4B*	rs6830886	*INPP4B*	rs2165819	*INPP4B*	rs2137255	*PIK3C2B*
rs10009093	*INPP4B*	rs6837163	*INPP4B*	rs331941	*INPP4B*	rs2271421	*PIK3C2B*
rs1443187	*INPP4B*	rs6842783	*INPP4B*	rs331946	*INPP4B*	rs2999484	*PIK3C2B*
rs12644329	*INPP4B*	rs6846839	*INPP4B*	rs966457	*INPP4B*	rs3014637	*PIK3C2B*
rs4690699	*INPP4B*	rs6847049	*INPP4B*	rs12504681	*INPP4B*	rs3106366	*PIK3C2B*
rs2165819	*INPP4B*	rs713071	*INPP4B*	rs1992418	*INPP4B*	rs3747633	*PIK3C2B*
rs331941	*INPP4B*	rs716762	*INPP4B*	rs4690728	*INPP4B*	rs7556371	*PIK3C2B*
rs331946	*INPP4B*	rs745719	*INPP4B*	rs6537129	*INPP4B*	rs10160860	*PIK3C2G*
rs966457	*INPP4B*	rs7668133	*INPP4B*	rs7914354	*INPP5A*	rs10841019	*PIK3C2G*
rs12504681	*INPP4B*	rs7685002	*INPP4B*	rs10747068	*INPP5A*	rs10841025	*PIK3C2G*
rs1992418	*INPP4B*	rs7688435	*INPP4B*	rs4272721	*INPP5A*	rs10841036	*PIK3C2G*
rs4690728	*INPP4B*	rs7691439	*INPP4B*	rs7903076	*INPP5A*	rs10841043	*PIK3C2G*
rs6537129	*INPP4B*	rs960678	*INPP4B*	rs4394754	*INPP5A*	rs10841049	*PIK3C2G*
rs6537129	*INPP4B*	rs966457	*INPP4B*	rs11818345	*INPP5A*	rs11044026	*PIK3C2G*
rs7914354	*INPP5A*	rs975136	*INPP4B*	rs7912273	*INPP5A*	rs11044075	*PIK3C2G*

rs10747068	*INPP5A*	rs978752	*INPP4B*	rs12241389	*INPP5A*	rs11044082	*PIK3C2G*
rs4272721	*INPP5A*	rs10747068	*INPP5A*	rs2786900	*INPP5A*	rs11044084	*PIK3C2G*
rs7903076	*INPP5A*	rs10781583	*INPP5A*	rs2492768	*INPP5A*	rs11044171	*PIK3C2G*
rs4394754	*INPP5A*	rs10781585	*INPP5A*	rs7084105	*INPP5A*	rs11044223	*PIK3C2G*
rs11818345	*INPP5A*	rs11146457	*INPP5A*	rs7907513	*INPP5A*	rs12227441	*PIK3C2G*
rs7912273	*INPP5A*	rs11146487	*INPP5A*	rs7091957	*INPP5A*	rs12297325	*PIK3C2G*
rs2786900	*INPP5A*	rs1133400	*INPP5A*	rs4880419	*INPP5A*	rs12367676	*PIK3C2G*
rs2492768	*INPP5A*	rs11818345	*INPP5A*	rs12412313	*INPP5A*	rs12371624	*PIK3C2G*
rs7084105	*INPP5A*	rs12241389	*INPP5A*	rs10781583	*INPP5A*	rs12427286	*PIK3C2G*
rs7907513	*INPP5A*	rs12267364	*INPP5A*	rs7914822	*INPP5A*	rs12581163	*PIK3C2G*
rs7091957	*INPP5A*	rs12412313	*INPP5A*	rs11146457	*INPP5A*	rs12582971	*PIK3C2G*
rs4880419	*INPP5A*	rs12775208	*INPP5A*	rs2767419	*INPP5A*	rs12822135	*PIK3C2G*
rs12412313	*INPP5A*	rs12783312	*INPP5A*	rs2246288	*INPP5A*	rs12827287	*PIK3C2G*
rs1133400	*INPP5A*	rs2246288	*INPP5A*	rs2803997	*INPP5A*	rs1447408	*PIK3C2G*
rs10781583	*INPP5A*	rs2492768	*INPP5A*	rs873946	*INPP5A*	rs17409120	*PIK3C2G*
rs7914822	*INPP5A*	rs2737398	*INPP5A*	rs3793669	*INPP5A*	rs17411508	*PIK3C2G*
rs11146457	*INPP5A*	rs2767419	*INPP5A*	rs3793670	*INPP5A*	rs17418422	*PIK3C2G*
rs2767419	*INPP5A*	rs2786900	*INPP5A*	rs913193	*INPP5A*	rs17419409	*PIK3C2G*
rs2246288	*INPP5A*	rs2803989	*INPP5A*	rs3793673	*INPP5A*	rs2290044	*PIK3C2G*
rs2803997	*INPP5A*	rs2803997	*INPP5A*	rs11146487	*INPP5A*	rs2931484	*PIK3C2G*
rs873946	*INPP5A*	rs3793669	*INPP5A*	rs10781585	*INPP5A*	rs4369463	*PIK3C2G*
rs3793669	*INPP5A*	rs3793670	*INPP5A*	rs12267364	*INPP5A*	rs4534639	*PIK3C2G*
rs3793670	*INPP5A*	rs3793673	*INPP5A*	rs2803989	*INPP5A*	rs4595599	*PIK3C2G*
rs913193	*INPP5A*	rs3793687	*INPP5A*	rs3793687	*INPP5A*	rs4764409	*PIK3C2G*
rs3793673	*INPP5A*	rs4272721	*INPP5A*	rs12775208	*INPP5A*	rs4764412	*PIK3C2G*
rs11146487	*INPP5A*	rs4394754	*INPP5A*	rs2737398	*INPP5A*	rs7314398	*PIK3C2G*
rs10781585	*INPP5A*	rs4880419	*INPP5A*	rs473279	*INPP5B*	rs7964182	*PIK3C2G*
rs12267364	*INPP5A*	rs7084105	*INPP5A*	rs4653342	*INPP5B*	rs9634063	*PIK3C2G*
rs2803989	*INPP5A*	rs7091957	*INPP5A*	rs1400349	*INPP5D*	rs1944967	*PIK3C3*
rs3793687	*INPP5A*	rs7903076	*INPP5A*	rs12694922	*INPP5D*	rs6507451	*PIK3C3*
rs12775208	*INPP5A*	rs7907513	*INPP5A*	rs12694923	*INPP5D*	rs9956832	*PIK3C3*
rs2737398	*INPP5A*	rs7912273	*INPP5A*	rs4246649	*INPP5D*	rs13320527	*PIK3CA*
rs2737398	*INPP5A*	rs7914354	*INPP5A*	rs4973063	*INPP5D*	rs558905	*PIK3CB*
rs473279	*INPP5B*	rs7914822	*INPP5A*	rs4439944	*INPP5D*	rs10864436	*PIK3CD*
rs4653342	*INPP5B*	rs873946	*INPP5A*	rs4257390	*INPP5D*	rs6540962	*PIK3CD*
rs4653342	*INPP5B*	rs913193	*INPP5A*	rs4073363	*INPP5D*	rs11766675	*PIK3CG*
rs1400349	*INPP5D*	rs473279	*INPP5B*	rs4973599	*INPP5D*	rs4727666	*PIK3CG*
rs12694922	*INPP5D*	rs10193128	*INPP5D*	rs4597514	*INPP5D*	rs6956373	*PIK3CG*
rs12694923	*INPP5D*	rs10203185	*INPP5D*	rs4315512	*INPP5D*	rs757902	*PIK3CG*
rs4246649	*INPP5D*	rs10803668	*INPP5D*	rs4603757	*INPP5D*	rs849367	*PIK3CG*
rs4973063	*INPP5D*	rs10929316	*INPP5D*	rs7425956	*INPP5D*	rs849375	*PIK3CG*
rs4439944	*INPP5D*	rs10933435	*INPP5D*	rs4356648	*INPP5D*	rs10177810	*PIKFYVE*
rs4257390	*INPP5D*	rs11673739	*INPP5D*	rs7608422	*INPP5D*	rs10190458	*PIKFYVE*
rs4073363	*INPP5D*	rs11674483	*INPP5D*	rs7566856	*INPP5D*	rs13407268	*PIKFYVE*

rs4973599	*INPP5D*	rs11682728	*INPP5D*	rs4335931	*INPP5D*	rs17652774	*PIKFYVE*
rs4315512	*INPP5D*	rs11693862	*INPP5D*	rs6437089	*INPP5D*	rs1866046	*PIKFYVE*
rs4603757	*INPP5D*	rs12694922	*INPP5D*	rs9750891	*INPP5D*	rs2289171	*PIKFYVE*
rs7425956	*INPP5D*	rs12694923	*INPP5D*	rs7570061	*INPP5D*	rs4675764	*PIKFYVE*
rs4356648	*INPP5D*	rs13021302	*INPP5D*	rs9288685	*INPP5D*	rs10828316	*PIP4K2A*
rs7608422	*INPP5D*	rs13031194	*INPP5D*	rs10193128	*INPP5D*	rs11013067	*PIP4K2A*
rs7566856	*INPP5D*	rs13385922	*INPP5D*	rs10933435	*INPP5D*	rs11013069	*PIP4K2A*
rs4335931	*INPP5D*	rs1400349	*INPP5D*	rs11693862	*INPP5D*	rs11013086	*PIP4K2A*
rs6437089	*INPP5D*	rs14243	*INPP5D*	rs11682728	*INPP5D*	rs11013095	*PIP4K2A*
rs9750891	*INPP5D*	rs3792117	*INPP5D*	rs10929316	*INPP5D*	rs11013103	*PIP4K2A*
rs7570061	*INPP5D*	rs3890760	*INPP5D*	rs11673739	*INPP5D*	rs11597156	*PIP4K2A*
rs9288685	*INPP5D*	rs4073363	*INPP5D*	rs6715810	*INPP5D*	rs12098721	*PIP4K2A*
rs10193128	*INPP5D*	rs4246649	*INPP5D*	rs10803668	*INPP5D*	rs12253847	*PIP4K2A*
rs10933435	*INPP5D*	rs4257390	*INPP5D*	rs7569837	*INPP5D*	rs12355895	*PIP4K2A*
rs11693862	*INPP5D*	rs4315512	*INPP5D*	rs13385922	*INPP5D*	rs12357384	*PIP4K2A*
rs11682728	*INPP5D*	rs4335931	*INPP5D*	rs6720896	*INPP5D*	rs12773197	*PIP4K2A*
rs7421653	*INPP5D*	rs4356648	*INPP5D*	rs4571051	*INPP5D*	rs16922578	*PIP4K2A*
rs10929316	*INPP5D*	rs4436949	*INPP5D*	rs4503982	*INPP5D*	rs2559523	*PIP4K2A*
rs11673739	*INPP5D*	rs4439944	*INPP5D*	rs4436949	*INPP5D*	rs2559524	*PIP4K2A*
rs6715810	*INPP5D*	rs4468807	*INPP5D*	rs6740918	*INPP5D*	rs2765993	*PIP4K2A*
rs10803668	*INPP5D*	rs4503982	*INPP5D*	rs7584458	*INPP5D*	rs7071450	*PIP4K2A*
rs7569837	*INPP5D*	rs4571051	*INPP5D*	rs3890760	*INPP5D*	rs7075433	*PIP4K2A*
rs13385922	*INPP5D*	rs4597514	*INPP5D*	rs10203185	*INPP5D*	rs7075499	*PIP4K2A*
rs6720896	*INPP5D*	rs4603757	*INPP5D*	rs11674483	*INPP5D*	rs7088318	*PIP4K2A*
rs4571051	*INPP5D*	rs4663784	*INPP5D*	rs4468807	*INPP5D*	rs7094131	*PIP4K2A*
rs4503982	*INPP5D*	rs4663834	*INPP5D*	rs9247	*INPP5D*	rs7094187	*PIP4K2A*
rs4436949	*INPP5D*	rs4973063	*INPP5D*	rs4663784	*INPP5D*	rs7912144	*PIP4K2A*
rs6740918	*INPP5D*	rs4973599	*INPP5D*	rs3792117	*INPP5D*	rs7919839	*PIP4K2A*
rs7584458	*INPP5D*	rs6431580	*INPP5D*	rs14243	*INPP5D*	rs943196	*PIP4K2A*
rs3890760	*INPP5D*	rs6431586	*INPP5D*	rs13031194	*INPP5D*	rs11613457	*PIP4K2C*
rs10203185	*INPP5D*	rs6437089	*INPP5D*	rs4663834	*INPP5D*	rs703837	*PIP4K2C*
rs11674483	*INPP5D*	rs6715810	*INPP5D*	rs6431580	*INPP5D*	rs4520422	*PIP5K1A*
rs4468807	*INPP5D*	rs6720896	*INPP5D*	rs6431586	*INPP5D*	rs10746974	*PIP5K1B*
rs9247	*INPP5D*	rs6740918	*INPP5D*	rs13021302	*INPP5D*	rs10781117	*PIP5K1B*
rs4663784	*INPP5D*	rs7425956	*INPP5D*	rs7580869	*INPP5D*	rs10869396	*PIP5K1B*
rs3792117	*INPP5D*	rs7566856	*INPP5D*	rs1130635	*INPP5E*	rs11143417	*PIP5K1B*
rs14243	*INPP5D*	rs7569837	*INPP5D*	rs1128877	*INPP5E*	rs11144027	*PIP5K1B*
rs13031194	*INPP5D*	rs7570061	*INPP5D*	rs1127162	*INPP5E*	rs12339235	*PIP5K1B*
rs4663834	*INPP5D*	rs7580869	*INPP5D*	rs1127152	*INPP5E*	rs12349586	*PIP5K1B*
rs6431580	*INPP5D*	rs7584458	*INPP5D*	rs5997872	*INPP5J*	rs12380573	*PIP5K1B*
rs6431586	*INPP5D*	rs7608422	*INPP5D*	rs2074736	*INPP5J*	rs12686355	*PIP5K1B*
rs13021302	*INPP5D*	rs9247	*INPP5D*	rs2240430	*INPP5J*	rs12686693	*PIP5K1B*
rs7580869	*INPP5D*	rs9288685	*INPP5D*	rs917208	*INPP5J*	rs17058884	*PIP5K1B*
rs1130635	*INPP5E*	rs9750891	*INPP5D*	rs1004243	*INPP5J*	rs17392931	*PIP5K1B*

rs1128877	*INPP5E*	rs1127152	*INPP5E*	rs5753463	*INPP5J*	rs6560444	*PIP5K1B*
rs1127162	*INPP5E*	rs1127162	*INPP5E*	rs2017301	*INPP5J*	rs11672559	*PIP5K1C*
rs1127152	*INPP5E*	rs1128877	*INPP5E*	rs3747152	*INPP5J*	rs3746124	*PIP5K1C*
rs1127152	*INPP5E*	rs1130635	*INPP5E*	rs2240432	*INPP5J*	rs3739821	*PIP5KL1*
rs5997872	*INPP5J*	rs1004243	*INPP5J*	rs5753469	*INPP5J*	rs1018443	*PLCB1*
rs2074736	*INPP5J*	rs2017301	*INPP5J*	rs8135641	*INPP5J*	rs1033684	*PLCB1*
rs2240430	*INPP5J*	rs2074736	*INPP5J*	rs2232183	*INPP5J*	rs11087808	*PLCB1*
rs917208	*INPP5J*	rs2074739	*INPP5J*	rs5753472	*INPP5J*	rs11698656	*PLCB1*
rs1004243	*INPP5J*	rs2232176	*INPP5J*	rs2074739	*INPP5J*	rs11906514	*PLCB1*
rs5753463	*INPP5J*	rs2232183	*INPP5J*	rs2232176	*INPP5J*	rs12053642	*PLCB1*
rs2283877	*INPP5J*	rs2240430	*INPP5J*	rs3788428	*INPP5J*	rs1232782	*PLCB1*
rs2017301	*INPP5J*	rs2240432	*INPP5J*	rs3761431	*INPP5J*	rs12480099	*PLCB1*
rs3747152	*INPP5J*	rs2283877	*INPP5J*	rs5753480	*INPP5J*	rs12624339	*PLCB1*
rs2240432	*INPP5J*	rs3747152	*INPP5J*	rs4820944	*INPP5J*	rs13040221	*PLCB1*
rs5753469	*INPP5J*	rs3761431	*INPP5J*	rs2358973	*INPP5K*	rs1342585	*PLCB1*
rs8135641	*INPP5J*	rs3788428	*INPP5J*	rs7218128	*INPP5K*	rs1474683	*PLCB1*
rs2232183	*INPP5J*	rs4820944	*INPP5J*	rs1879488	*INPP5K*	rs1474937	*PLCB1*
rs5753472	*INPP5J*	rs5753463	*INPP5J*	rs1109303	*INPP5K*	rs1534897	*PLCB1*
rs2074739	*INPP5J*	rs5753469	*INPP5J*	rs15362	*INPP5K*	rs1555212	*PLCB1*
rs2232176	*INPP5J*	rs5753472	*INPP5J*	rs2277669	*INPP5K*	rs1569604	*PLCB1*
rs3788428	*INPP5J*	rs5753480	*INPP5J*	rs10521113	*INPP5K*	rs1605791	*PLCB1*
rs3761431	*INPP5J*	rs5997872	*INPP5J*	rs17761155	*INPP5K*	rs16995121	*PLCB1*
rs5753480	*INPP5J*	rs8135641	*INPP5J*	rs2270227	*INPP5K*	rs17362299	*PLCB1*
rs4820944	*INPP5J*	rs917208	*INPP5J*	rs2270229	*INPP5K*	rs17431073	*PLCB1*
rs7218128	*INPP5K*	rs10521113	*INPP5K*	rs11235468	*INPPL1*	rs17436253	*PLCB1*
rs1109303	*INPP5K*	rs1109303	*INPP5K*	rs514933	*INPPL1*	rs17446308	*PLCB1*
rs15362	*INPP5K*	rs15362	*INPP5K*	rs2276048	*INPPL1*	rs17446441	*PLCB1*
rs2277669	*INPP5K*	rs17761155	*INPP5K*	rs7110260	*INPPL1*	rs2064272	*PLCB1*
rs10521113	*INPP5K*	rs1879488	*INPP5K*	rs3749237	*IP6K1*	rs2143205	*PLCB1*
rs17761155	*INPP5K*	rs2270227	*INPP5K*	rs9855505	*IP6K1*	rs2143266	*PLCB1*
rs2270227	*INPP5K*	rs2270229	*INPP5K*	rs9829155	*IP6K1*	rs2179138	*PLCB1*
rs2270229	*INPP5K*	rs2277669	*INPP5K*	rs6802890	*IP6K1*	rs2179984	*PLCB1*
rs2270229	*INPP5K*	rs2358973	*INPP5K*	rs7629936	*IP6K1*	rs2221695	*PLCB1*
rs11235468	*INPPL1*	rs7214615	*INPP5K*	rs990211	*IP6K2*	rs227133	*PLCB1*
rs651933	*INPPL1*	rs7218128	*INPP5K*	rs3172494	*IP6K2*	rs2327025	*PLCB1*
rs514933	*INPPL1*	rs11235468	*INPPL1*	rs12497850	*IP6K2*	rs2423354	*PLCB1*
rs2276048	*INPPL1*	rs2276048	*INPPL1*	rs9882443	*IP6K2*	rs2423356	*PLCB1*
rs7110260	*INPPL1*	rs514933	*INPPL1*	rs4077495	*IP6K2*	rs2423363	*PLCB1*
rs7110260	*INPPL1*	rs651933	*INPPL1*	rs6766238	*IP6K2*	rs2423366	*PLCB1*
rs3749237	*IP6K1*	rs7110260	*INPPL1*	rs498114	*IP6K3*	rs2719795	*PLCB1*
rs9855505	*IP6K1*	rs3749237	*IP6K1*	rs2281829	*IP6K3*	rs2745772	*PLCB1*
rs9829155	*IP6K1*	rs6802890	*IP6K1*	rs542441	*IP6K3*	rs2745776	*PLCB1*
rs6802890	*IP6K1*	rs7629936	*IP6K1*	rs9380374	*IP6K3*	rs3848835	*PLCB1*
rs7629936	*IP6K1*	rs9829155	*IP6K1*	rs3818532	*IP6K3*	rs4419296	*PLCB1*

rs7629936	*IP6K1*	rs9855505	*IP6K1*	rs649775	*IP6K3*	rs4496390	*PLCB1*
rs990211	*IP6K2*	rs12497850	*IP6K2*	rs2966	*IP6K3*	rs4813853	*PLCB1*
rs3172494	*IP6K2*	rs3172494	*IP6K2*	rs10947433	*IP6K3*	rs4813854	*PLCB1*
rs12497850	*IP6K2*	rs4077495	*IP6K2*	rs4713668	*IP6K3*	rs4813863	*PLCB1*
rs9882443	*IP6K2*	rs6766238	*IP6K2*	rs471942	*IP6K3*	rs4816085	*PLCB1*
rs4077495	*IP6K2*	rs9882443	*IP6K2*	rs6457740	*IP6K3*	rs4816090	*PLCB1*
rs6766238	*IP6K2*	rs990211	*IP6K2*	rs622917	*IP6K3*	rs6039104	*PLCB1*
rs6766238	*IP6K2*	rs10947433	*IP6K3*	rs652049	*IP6K3*	rs6039107	*PLCB1*
rs498114	*IP6K3*	rs10947435	*IP6K3*	rs9469583	*IP6K3*	rs6039189	*PLCB1*
rs2281829	*IP6K3*	rs12211490	*IP6K3*	rs10947435	*IP6K3*	rs6039190	*PLCB1*
rs542441	*IP6K3*	rs1536500	*IP6K3*	rs4304152	*IP6K3*	rs6039268	*PLCB1*
rs9380374	*IP6K3*	rs1536501	*IP6K3*	rs1536500	*IP6K3*	rs6039298	*PLCB1*
rs3818532	*IP6K3*	rs2281829	*IP6K3*	rs6919321	*IP6K3*	rs6039312	*PLCB1*
rs649775	*IP6K3*	rs2966	*IP6K3*	rs9380376	*IP6K3*	rs6055562	*PLCB1*
rs2966	*IP6K3*	rs3818532	*IP6K3*	rs12211490	*IP6K3*	rs6055578	*PLCB1*
rs10947433	*IP6K3*	rs4304152	*IP6K3*	rs1536501	*IP6K3*	rs6055625	*PLCB1*
rs4713668	*IP6K3*	rs4711345	*IP6K3*	rs4711345	*IP6K3*	rs6055748	*PLCB1*
rs471942	*IP6K3*	rs4711348	*IP6K3*	rs755495	*IP6K3*	rs6055853	*PLCB1*
rs6457740	*IP6K3*	rs4713668	*IP6K3*	rs4711348	*IP6K3*	rs6055858	*PLCB1*
rs622917	*IP6K3*	rs471942	*IP6K3*	rs6904716	*IP6K3*	rs6055889	*PLCB1*
rs652049	*IP6K3*	rs498114	*IP6K3*	rs6933607	*IP6K3*	rs6055912	*PLCB1*
rs9469583	*IP6K3*	rs542441	*IP6K3*	rs1199098	*IPMK*	rs6055927	*PLCB1*
rs10947435	*IP6K3*	rs622917	*IP6K3*	rs7068428	*IPMK*	rs6055928	*PLCB1*
rs4304152	*IP6K3*	rs6457740	*IP6K3*	rs11006086	*IPMK*	rs6055944	*PLCB1*
rs1536500	*IP6K3*	rs649775	*IP6K3*	rs6481383	*IPMK*	rs6056006	*PLCB1*
rs6919321	*IP6K3*	rs652049	*IP6K3*	rs1867571	*IPMK*	rs6056028	*PLCB1*
rs9380376	*IP6K3*	rs6904716	*IP6K3*	rs1416764	*IPMK*	rs6056080	*PLCB1*
rs12211490	*IP6K3*	rs6919321	*IP6K3*	rs1980705	*IPPK*	rs6056111	*PLCB1*
rs1536501	*IP6K3*	rs6933607	*IP6K3*	rs7043114	*IPPK*	rs6056114	*PLCB1*
rs4711345	*IP6K3*	rs755495	*IP6K3*	rs7863890	*IPPK*	rs6056198	*PLCB1*
rs755495	*IP6K3*	rs9380374	*IP6K3*	rs9969804	*IPPK*	rs6077326	*PLCB1*
rs4711348	*IP6K3*	rs9380376	*IP6K3*	rs4744143	*IPPK*	rs6077350	*PLCB1*
rs6904716	*IP6K3*	rs9469583	*IP6K3*	rs912261	*IPPK*	rs6077420	*PLCB1*
rs6933607	*IP6K3*	rs11006086	*IPMK*	rs13285641	*IPPK*	rs6077425	*PLCB1*
rs6933607	*IP6K3*	rs1416764	*IPMK*	rs10992420	*IPPK*	rs6086343	*PLCB1*
rs1199098	*IPMK*	rs1867571	*IPMK*	rs2385089	*ISYNA1*	rs6086346	*PLCB1*
rs7068428	*IPMK*	rs6481383	*IPMK*	rs731945	*ISYNA1*	rs6086402	*PLCB1*
rs11006086	*IPMK*	rs7068428	*IPMK*	rs731945	*ISYNA1*	rs6086458	*PLCB1*
rs6481383	*IPMK*	rs7087498	*IPMK*	rs1043542	*ITPK1*	rs6086490	*PLCB1*
rs2275442	*IPMK*	rs7899961	*IPMK*	rs11446	*ITPK1*	rs6086495	*PLCB1*
rs1867571	*IPMK*	rs10992420	*IPPK*	rs3783925	*ITPK1*	rs6086518	*PLCB1*
rs1416764	*IPMK*	rs13285641	*IPPK*	rs2295394	*ITPK1*	rs6086525	*PLCB1*
rs1416764	*IPMK*	rs1980705	*IPPK*	rs4900164	*ITPK1*	rs6086582	*PLCB1*
rs1980705	*IPPK*	rs4744143	*IPPK*	rs1006888	*ITPK1*	rs6108174	*PLCB1*

rs7043114	*IPPK*	rs7043114	*IPPK*	rs2402226	*ITPK1*	rs6108205	*PLCB1*
rs7863890	*IPPK*	rs7863890	*IPPK*	rs3818176	*ITPK1*	rs6118073	*PLCB1*
rs9969804	*IPPK*	rs912261	*IPPK*	rs4586354	*ITPK1*	rs6118083	*PLCB1*
rs4744143	*IPPK*	rs9969804	*IPPK*	rs11625662	*ITPK1*	rs6118219	*PLCB1*
rs912261	*IPPK*	rs731945	*ISYNA1*	rs941578	*ITPK1*	rs6118257	*PLCB1*
rs13285641	*IPPK*	rs1006888	*ITPK1*	rs12587187	*ITPK1*	rs6133556	*PLCB1*
rs10992420	*IPPK*	rs10136012	*ITPK1*	rs3783919	*ITPK1*	rs6133566	*PLCB1*
rs10992420	*IPPK*	rs1043542	*ITPK1*	rs17128706	*ITPK1*	rs6133612	*PLCB1*
rs731945	*ISYNA1*	rs11446	*ITPK1*	rs3783914	*ITPK1*	rs6133635	*PLCB1*
rs731945	*ISYNA1*	rs11625662	*ITPK1*	rs12589455	*ITPK1*	rs6140549	*PLCB1*
rs1043542	*ITPK1*	rs11628021	*ITPK1*	rs3783913	*ITPK1*	rs6140562	*PLCB1*
rs11446	*ITPK1*	rs12434958	*ITPK1*	rs12435423	*ITPK1*	rs6140619	*PLCB1*
rs3783925	*ITPK1*	rs12435325	*ITPK1*	rs12435325	*ITPK1*	rs6140774	*PLCB1*
rs2295394	*ITPK1*	rs12435423	*ITPK1*	rs12586382	*ITPK1*	rs708912	*PLCB1*
rs4900164	*ITPK1*	rs12586382	*ITPK1*	rs4905029	*ITPK1*	rs708931	*PLCB1*
rs1006888	*ITPK1*	rs12587187	*ITPK1*	rs17128737	*ITPK1*	rs7271063	*PLCB1*
rs2402226	*ITPK1*	rs12589455	*ITPK1*	rs3783910	*ITPK1*	rs768989	*PLCB1*
rs4586354	*ITPK1*	rs12895695	*ITPK1*	rs11628021	*ITPK1*	rs771944	*PLCB1*
rs11625662	*ITPK1*	rs1612612	*ITPK1*	rs1612612	*ITPK1*	rs8115925	*PLCB1*
rs941578	*ITPK1*	rs17128706	*ITPK1*	rs1740696	*ITPK1*	rs8117234	*PLCB1*
rs12587187	*ITPK1*	rs17128737	*ITPK1*	rs1740598	*ITPK1*	rs8123323	*PLCB1*
rs3783919	*ITPK1*	rs1740596	*ITPK1*	rs10136012	*ITPK1*	rs8125486	*PLCB1*
rs17128706	*ITPK1*	rs1740598	*ITPK1*	rs1740596	*ITPK1*	rs2242119	*PLCB2*
rs3783914	*ITPK1*	rs1740689	*ITPK1*	rs1740694	*ITPK1*	rs4334271	*PLCB2*
rs12589455	*ITPK1*	rs1740694	*ITPK1*	rs2180369	*ITPK1*	rs11600990	*PLCB3*
rs3783913	*ITPK1*	rs1740696	*ITPK1*	rs941542	*ITPK1*	rs2282490	*PLCB3*
rs12435423	*ITPK1*	rs1740698	*ITPK1*	rs749619	*ITPK1*	rs1028338	*PLCB4*
rs12435325	*ITPK1*	rs2180369	*ITPK1*	rs2749509	*ITPK1*	rs13041524	*PLCB4*
rs12586382	*ITPK1*	rs2295394	*ITPK1*	rs1740689	*ITPK1*	rs13044386	*PLCB4*
rs4905029	*ITPK1*	rs2402226	*ITPK1*	rs1740698	*ITPK1*	rs16995573	*PLCB4*
rs17128737	*ITPK1*	rs2749509	*ITPK1*	rs4905043	*ITPK1*	rs16995731	*PLCB4*
rs3783910	*ITPK1*	rs3783910	*ITPK1*	rs941541	*ITPK1*	rs2076393	*PLCB4*
rs11628021	*ITPK1*	rs3783913	*ITPK1*	rs957362	*ITPK1*	rs2179321	*PLCB4*
rs1612612	*ITPK1*	rs3783914	*ITPK1*	rs768356	*ITPK1*	rs2208297	*PLCB4*
rs1740696	*ITPK1*	rs3783919	*ITPK1*	rs12895695	*ITPK1*	rs2224357	*PLCB4*
rs1740598	*ITPK1*	rs3783925	*ITPK1*	rs12434958	*ITPK1*	rs2276483	*PLCB4*
rs10136012	*ITPK1*	rs3818176	*ITPK1*	rs12434958	*ITPK1*	rs2276484	*PLCB4*
rs1740596	*ITPK1*	rs4586354	*ITPK1*	rs1757463	*ITPKA*	rs2299679	*PLCB4*
rs1740694	*ITPK1*	rs4900164	*ITPK1*	rs170296	*ITPKA*	rs2327162	*PLCB4*
rs2180369	*ITPK1*	rs4905029	*ITPK1*	rs2305030	*ITPKA*	rs2876163	*PLCB4*
rs941542	*ITPK1*	rs4905043	*ITPK1*	rs2305030	*ITPKA*	rs3787309	*PLCB4*
rs749619	*ITPK1*	rs749619	*ITPK1*	rs10916019	*ITPKB*	rs3819579	*PLCB4*
rs2749509	*ITPK1*	rs768356	*ITPK1*	rs697845	*ITPKB*	rs4369940	*PLCB4*
rs1740689	*ITPK1*	rs941541	*ITPK1*	rs1144841	*ITPKB*	rs5011374	*PLCB4*

rs1740698	*ITPK1*	rs941542	*ITPK1*	rs1050492	*ITPKB*	rs6039386	*PLCB4*
rs4905043	*ITPK1*	rs941578	*ITPK1*	rs1144838	*ITPKB*	rs6039410	*PLCB4*
rs941541	*ITPK1*	rs957362	*ITPK1*	rs3754378	*ITPKB*	rs6056386	*PLCB4*
rs957362	*ITPK1*	rs170296	*ITPKA*	rs2236604	*ITPKB*	rs6056427	*PLCB4*
rs768356	*ITPK1*	rs1757463	*ITPKA*	rs1288934	*ITPKB*	rs6056448	*PLCB4*
rs12895695	*ITPK1*	rs2305030	*ITPKA*	rs17522524	*ITPKB*	rs6056519	*PLCB4*
rs12434958	*ITPK1*	rs10495249	*ITPKB*	rs3820635	*ITPKB*	rs6056526	*PLCB4*
rs1757463	*ITPKA*	rs1050492	*ITPKB*	rs1144836	*ITPKB*	rs6056552	*PLCB4*
rs170296	*ITPKA*	rs10916019	*ITPKB*	rs3768373	*ITPKB*	rs6056628	*PLCB4*
rs2305030	*ITPKA*	rs1144836	*ITPKB*	rs12077348	*ITPKB*	rs6056645	*PLCB4*
rs10916019	*ITPKB*	rs1144838	*ITPKB*	rs3754390	*ITPKB*	rs6077516	*PLCB4*
rs697845	*ITPKB*	rs1144841	*ITPKB*	rs12094617	*ITPKB*	rs6086762	*PLCB4*
rs1144841	*ITPKB*	rs12077348	*ITPKB*	rs708766	*ITPKB*	rs6086834	*PLCB4*
rs1050492	*ITPKB*	rs12078663	*ITPKB*	rs697851	*ITPKB*	rs6086865	*PLCB4*
rs1144838	*ITPKB*	rs12094617	*ITPKB*	rs1341283	*ITPKB*	rs6086897	*PLCB4*
rs3754378	*ITPKB*	rs1288934	*ITPKB*	rs3768405	*ITPKB*	rs6086904	*PLCB4*
rs2236604	*ITPKB*	rs1341283	*ITPKB*	rs3768408	*ITPKB*	rs6118479	*PLCB4*
rs1288934	*ITPKB*	rs17522524	*ITPKB*	rs708772	*ITPKB*	rs6118558	*PLCB4*
rs17522524	*ITPKB*	rs2236604	*ITPKB*	rs3768414	*ITPKB*	rs6118591	*PLCB4*
rs3820635	*ITPKB*	rs3754378	*ITPKB*	rs10495249	*ITPKB*	rs6118616	*PLCB4*
rs1144836	*ITPKB*	rs3754390	*ITPKB*	rs708776	*ITPKB*	rs6140861	*PLCB4*
rs3768373	*ITPKB*	rs3754407	*ITPKB*	rs6667260	*ITPKB*	rs6516454	*PLCB4*
rs12077348	*ITPKB*	rs3768373	*ITPKB*	rs708777	*ITPKB*	rs8183334	*PLCB4*
rs3754390	*ITPKB*	rs3768405	*ITPKB*	rs3865451	*ITPKC*	rs9825655	*PLCD1*
rs12094617	*ITPKB*	rs3768408	*ITPKB*	rs3865452	*ITPKC*	rs9858321	*PLCD1*
rs708766	*ITPKB*	rs3768414	*ITPKB*	rs2604913	*ITPKC*	rs1052169	*PLCD3*
rs697851	*ITPKB*	rs3820635	*ITPKB*	rs890934	*ITPKC*	rs12944434	*PLCD3*
rs1341283	*ITPKB*	rs6667260	*ITPKB*	rs11668501	*ITPKC*	rs2239925	*PLCD3*
rs3768405	*ITPKB*	rs697845	*ITPKB*	rs3745216	*ITPKC*	rs2269746	*PLCD3*
rs3768408	*ITPKB*	rs697851	*ITPKB*	rs1870087	*ITPKC*	rs3744760	*PLCD3*
rs708772	*ITPKB*	rs708766	*ITPKB*	rs11202426	*MINPP1*	rs4362432	*PLCD3*
rs3768414	*ITPKB*	rs708772	*ITPKB*	rs10509407	*MINPP1*	rs7207047	*PLCD3*
rs10495249	*ITPKB*	rs708776	*ITPKB*	rs3843597	*MINPP1*	rs7224944	*PLCD3*
rs708776	*ITPKB*	rs708777	*ITPKB*	rs2311115	*MINPP1*	rs12989189	*PLCD4*
rs6667260	*ITPKB*	rs11668501	*ITPKC*	rs11202429	*MINPP1*	rs3845836	*PLCD4*
rs708777	*ITPKB*	rs1870087	*ITPKC*	rs3847448	*MINPP1*	rs10786155	*PLCE1*
rs708777	*ITPKB*	rs2604913	*ITPKC*	rs3847452	*MINPP1*	rs10882412	*PLCE1*
rs3865451	*ITPKC*	rs3745216	*ITPKC*	rs10509408	*MINPP1*	rs11187789	*PLCE1*
rs3865452	*ITPKC*	rs3865451	*ITPKC*	rs1408377	*MINPP1*	rs11187808	*PLCE1*
rs2604913	*ITPKC*	rs3865452	*ITPKC*	rs2147287	*MINPP1*	rs11187815	*PLCE1*
rs890934	*ITPKC*	rs890934	*ITPKC*	rs10788550	*MINPP1*	rs11187828	*PLCE1*
rs11668501	*ITPKC*	rs10509408	*MINPP1*	rs4824152	*MIOX*	rs11593126	*PLCE1*
rs10420685	*ITPKC*	rs10788550	*MINPP1*	rs9616854	*MIOX*	rs1223585	*PLCE1*
rs3745216	*ITPKC*	rs11202426	*MINPP1*	rs4824157	*MIOX*	rs12248509	*PLCE1*

rs1870087	*ITPKC*	rs11202429	*MINPP1*	rs8138406	*MIOX*	rs12766693	*PLCE1*
rs1870087	*ITPKC*	rs11202434	*MINPP1*	rs8138406	*MIOX*	rs12769135	*PLCE1*
rs11202426	*MINPP1*	rs1408377	*MINPP1*	rs16883137	*NUDT3*	rs17109869	*PLCE1*
rs3843597	*MINPP1*	rs2147287	*MINPP1*	rs10947494	*NUDT3*	rs17109928	*PLCE1*
rs2311115	*MINPP1*	rs2311115	*MINPP1*	rs1057691	*NUDT3*	rs17416616	*PLCE1*
rs11202429	*MINPP1*	rs2871690	*MINPP1*	rs464553	*NUDT3*	rs17516904	*PLCE1*
rs3847448	*MINPP1*	rs3843597	*MINPP1*	rs206937	*NUDT3*	rs1925243	*PLCE1*
rs3847452	*MINPP1*	rs3847448	*MINPP1*	rs3798560	*NUDT3*	rs1935961	*PLCE1*
rs10509408	*MINPP1*	rs3847452	*MINPP1*	rs4761517	*NUDT4*	rs2077218	*PLCE1*
rs1408377	*MINPP1*	rs4824152	*MIOX*	rs4247307	*NUDT4*	rs2226170	*PLCE1*
rs2147287	*MINPP1*	rs4824157	*MIOX*	rs11107007	*NUDT4*	rs2689693	*PLCE1*
rs10788550	*MINPP1*	rs8138406	*MIOX*	rs17837158	*NUDT4*	rs2689698	*PLCE1*
rs10788550	*MINPP1*	rs9616854	*MIOX*	rs11107008	*NUDT4*	rs6583926	*PLCE1*
rs4824152	*MIOX*	rs1003976	*NUDT3*	rs7487813	*NUDT4*	rs7908334	*PLCE1*
rs9616854	*MIOX*	rs10456425	*NUDT3*	rs7977140	*NUDT4*	rs7919066	*PLCE1*
rs4824157	*MIOX*	rs1057691	*NUDT3*	rs17790482	*NUDT4*	rs12624863	*PLCG1*
rs8138406	*MIOX*	rs10947415	*NUDT3*	rs12597	*NUDT4*	rs11643875	*PLCG2*
rs8138406	*MIOX*	rs10947427	*NUDT3*	rs7973701	*NUDT4*	rs11644436	*PLCG2*
rs16883137	*NUDT3*	rs10947433	*NUDT3*	rs12816436	*NUDT4*	rs11644646	*PLCG2*
rs10947494	*NUDT3*	rs10947435	*NUDT3*	rs6584138	*PI4K2A*	rs11859107	*PLCG2*
rs1057691	*NUDT3*	rs10947460	*NUDT3*	rs11189310	*PI4K2A*	rs11862662	*PLCG2*
rs464553	*NUDT3*	rs10947461	*NUDT3*	rs3890727	*PI4K2A*	rs11864701	*PLCG2*
rs206937	*NUDT3*	rs10947463	*NUDT3*	rs4919128	*PI4K2A*	rs12448055	*PLCG2*
rs3798560	*NUDT3*	rs10947479	*NUDT3*	rs2065672	*PI4K2A*	rs12448334	*PLCG2*
rs3798560	*NUDT3*	rs10947482	*NUDT3*	rs11189321	*PI4K2A*	rs12596639	*PLCG2*
rs4761517	*NUDT4*	rs10947494	*NUDT3*	rs10444068	*PI4K2A*	rs12598194	*PLCG2*
rs4247307	*NUDT4*	rs1105695	*NUDT3*	rs10786364	*PI4K2A*	rs12598402	*PLCG2*
rs11107007	*NUDT4*	rs1122554	*NUDT3*	rs3115231	*PI4K2B*	rs12599264	*PLCG2*
rs17837158	*NUDT4*	rs11751469	*NUDT3*	rs313548	*PI4K2B*	rs12716928	*PLCG2*
rs11107008	*NUDT4*	rs11751591	*NUDT3*	rs7661189	*PI4K2B*	rs12918369	*PLCG2*
rs7487813	*NUDT4*	rs11753213	*NUDT3*	rs313566	*PI4K2B*	rs12921780	*PLCG2*
rs7977140	*NUDT4*	rs11759461	*NUDT3*	rs313541	*PI4K2B*	rs13331678	*PLCG2*
rs17790482	*NUDT4*	rs12055409	*NUDT3*	rs313533	*PI4K2B*	rs16956040	*PLCG2*
rs12597	*NUDT4*	rs12195485	*NUDT3*	rs3796780	*PI4K2B*	rs17203310	*PLCG2*
rs7973701	*NUDT4*	rs12206652	*NUDT3*	rs4820579	*PI4KA*	rs17793122	*PLCG2*
rs12816436	*NUDT4*	rs12211490	*NUDT3*	rs1558657	*PI4KA*	rs3935877	*PLCG2*
rs12816436	*NUDT4*	rs12211633	*NUDT3*	rs2072513	*PI4KA*	rs3936112	*PLCG2*
rs6584138	*PI4K2A*	rs12529825	*NUDT3*	rs9608386	*PI4KA*	rs4133125	*PLCG2*
rs11189310	*PI4K2A*	rs12664430	*NUDT3*	rs165862	*PI4KA*	rs4243226	*PLCG2*
rs3890727	*PI4K2A*	rs13203745	*NUDT3*	rs17820181	*PI4KA*	rs4284633	*PLCG2*
rs4919128	*PI4K2A*	rs13205164	*NUDT3*	rs165793	*PI4KA*	rs4306504	*PLCG2*
rs2065672	*PI4K2A*	rs14398	*NUDT3*	rs165924	*PI4KA*	rs4369659	*PLCG2*
rs11189321	*PI4K2A*	rs1535948	*NUDT3*	rs178051	*PI4KA*	rs4398100	*PLCG2*
rs10444068	*PI4K2A*	rs1535950	*NUDT3*	rs178058	*PI4KA*	rs4405546	*PLCG2*

rs10786364	*PI4K2A*	rs1536036	*NUDT3*	rs4822606	*PI4KA*	rs4456499	*PLCG2*
rs10786364	*PI4K2A*	rs1536041	*NUDT3*	rs165598	*PI4KA*	rs4580153	*PLCG2*
rs3115231	*PI4K2B*	rs1536500	*NUDT3*	rs178070	*PI4KA*	rs4603554	*PLCG2*
rs313548	*PI4K2B*	rs1536501	*NUDT3*	rs10483104	*PI4KA*	rs4611452	*PLCG2*
rs7661189	*PI4K2B*	rs1547669	*NUDT3*	rs10483104	*PI4KA*	rs4888181	*PLCG2*
rs313566	*PI4K2B*	rs1555688	*NUDT3*	rs2298265	*PI4KB*	rs4889393	*PLCG2*
rs313541	*PI4K2B*	rs1565356	*NUDT3*	rs2031797	*PI4KB*	rs4889411	*PLCG2*
rs313533	*PI4K2B*	rs1565361	*NUDT3*	rs4971030	*PI4KB*	rs4889422	*PLCG2*
rs3796780	*PI4K2B*	rs1570760	*NUDT3*	rs1752388	*PI4KB*	rs4889425	*PLCG2*
rs3796780	*PI4K2B*	rs16869652	*NUDT3*	rs1056847	*PI4KB*	rs4889426	*PLCG2*
rs11705170	*PI4KA*	rs16869677	*NUDT3*	rs5022636	*PI4KB*	rs4889444	*PLCG2*
rs1558657	*PI4KA*	rs16883137	*NUDT3*	rs1752382	*PI4KB*	rs6420427	*PLCG2*
rs2072513	*PI4KA*	rs169737	*NUDT3*	rs11604561	*PIK3C2A*	rs6564940	*PLCG2*
rs9608386	*PI4KA*	rs17535965	*NUDT3*	rs3950680	*PIK3C2A*	rs7185362	*PLCG2*
rs165862	*PI4KA*	rs1776897	*NUDT3*	rs2040859	*PIK3C2A*	rs7187863	*PLCG2*
rs17820181	*PI4KA*	rs1873246	*NUDT3*	rs7946010	*PIK3C2A*	rs7194131	*PLCG2*
rs165793	*PI4KA*	rs1873249	*NUDT3*	rs621246	*PIK3C2A*	rs7201045	*PLCG2*
rs165924	*PI4KA*	rs1873250	*NUDT3*	rs621246	*PIK3C2A*	rs7202205	*PLCG2*
rs178051	*PI4KA*	rs1906953	*NUDT3*	rs12119503	*PIK3C2B*	rs7203619	*PLCG2*
rs178058	*PI4KA*	rs1994582	*NUDT3*	rs12061474	*PIK3C2B*	rs7342694	*PLCG2*
rs4822606	*PI4KA*	rs2029461	*NUDT3*	rs4951373	*PIK3C2B*	rs7499275	*PLCG2*
rs165598	*PI4KA*	rs206937	*NUDT3*	rs17334387	*PIK3C2B*	rs7500286	*PLCG2*
rs178070	*PI4KA*	rs210120	*NUDT3*	rs2271421	*PIK3C2B*	rs8047356	*PLCG2*
rs10483104	*PI4KA*	rs210131	*NUDT3*	rs2271424	*PIK3C2B*	rs8055043	*PLCG2*
rs10483104	*PI4KA*	rs210132	*NUDT3*	rs3747636	*PIK3C2B*	rs8055576	*PLCG2*
rs2298265	*PI4KB*	rs210134	*NUDT3*	rs12031854	*PIK3C2B*	rs8056564	*PLCG2*
rs2031797	*PI4KB*	rs210138	*NUDT3*	rs3747633	*PIK3C2B*	rs8062633	*PLCG2*
rs4971030	*PI4KB*	rs210139	*NUDT3*	rs3106366	*PIK3C2B*	rs8063355	*PLCG2*
rs1752388	*PI4KB*	rs210152	*NUDT3*	rs2271427	*PIK3C2B*	rs8063604	*PLCG2*
rs1056847	*PI4KB*	rs210170	*NUDT3*	rs3014637	*PIK3C2B*	rs8063813	*PLCG2*
rs5022636	*PI4KB*	rs210179	*NUDT3*	rs2137255	*PIK3C2B*	rs9932716	*PLCG2*
rs1752382	*PI4KB*	rs210203	*NUDT3*	rs2999484	*PIK3C2B*	rs9937223	*PLCG2*
rs1752382	*PI4KB*	rs2104362	*NUDT3*	rs2271415	*PIK3C2B*	rs9937704	*PLCG2*
rs11604561	*PIK3C2A*	rs211452	*NUDT3*	rs6594014	*PIK3C2B*	rs9938623	*PLCG2*
rs3950680	*PIK3C2A*	rs211455	*NUDT3*	rs11240748	*PIK3C2B*	rs3851357	*PLCH1*
rs2040859	*PIK3C2A*	rs211456	*NUDT3*	rs7556371	*PIK3C2B*	rs6440997	*PLCH1*
rs7946010	*PIK3C2A*	rs211457	*NUDT3*	rs10770333	*PIK3C2G*	rs7629025	*PLCH1*
rs621246	*PIK3C2A*	rs2229634	*NUDT3*	rs4369463	*PIK3C2G*	rs934591	*PLCH1*
rs621246	*PIK3C2A*	rs2229901	*NUDT3*	rs4385947	*PIK3C2G*	rs12049628	*PLCH2*
rs12119503	*PIK3C2B*	rs2239839	*NUDT3*	rs17409120	*PIK3C2G*	rs17373634	*PLCH2*
rs12061474	*PIK3C2B*	rs2247385	*NUDT3*	rs11044004	*PIK3C2G*	rs1021266	*PLCZ1*
rs17334387	*PIK3C2B*	rs2274154	*NUDT3*	rs7133666	*PIK3C2G*	rs1021267	*PLCZ1*
rs2271421	*PIK3C2B*	rs2274459	*NUDT3*	rs4237974	*PIK3C2G*	rs17488409	*PLCZ1*
rs2271424	*PIK3C2B*	rs2281820	*NUDT3*	rs4534639	*PIK3C2G*	rs6486914	*PLCZ1*

rs3747636	*PIK3C2B*	rs2281829	*NUDT3*	rs12582971	*PIK3C2G*	rs665197	*PLCZ1*
rs12031854	*PIK3C2B*	rs2282850	*NUDT3*	rs10505811	*PIK3C2G*	rs7972408	*PLCZ1*
rs3747633	*PIK3C2B*	rs2282851	*NUDT3*	rs9300118	*PIK3C2G*	rs12433746	*PLD4*
rs3106366	*PIK3C2B*	rs2296328	*NUDT3*	rs10160860	*PIK3C2G*	rs2841249	*PLD4*
rs2271427	*PIK3C2B*	rs2296329	*NUDT3*	rs4609650	*PIK3C2G*	rs2251844	*PPIP5K1*
rs3014637	*PIK3C2B*	rs2296337	*NUDT3*	rs11044026	*PIK3C2G*	rs2255042	*PPIP5K1*
rs2137255	*PIK3C2B*	rs2296340	*NUDT3*	rs10505810	*PIK3C2G*	rs2255663	*PPIP5K1*
rs2999484	*PIK3C2B*	rs2296341	*NUDT3*	rs11044034	*PIK3C2G*	rs496584	*PPIP5K1*
rs2271415	*PIK3C2B*	rs2296343	*NUDT3*	rs12309567	*PIK3C2G*	rs689797	*PPIP5K1*
rs6594014	*PIK3C2B*	rs2296748	*NUDT3*	rs10841019	*PIK3C2G*	rs694985	*PPIP5K1*
rs11240748	*PIK3C2B*	rs2395399	*NUDT3*	rs12297325	*PIK3C2G*	rs11744885	*PPIP5K2*
rs7556371	*PIK3C2B*	rs2451330	*NUDT3*	rs4595599	*PIK3C2G*	rs246912	*PPIP5K2*
rs7556371	*PIK3C2B*	rs2451334	*NUDT3*	rs11044058	*PIK3C2G*	rs246916	*PPIP5K2*
rs10770333	*PIK3C2G*	rs2451351	*NUDT3*	rs11044070	*PIK3C2G*	rs26258	*PPIP5K2*
rs4369463	*PIK3C2G*	rs2451361	*NUDT3*	rs12227441	*PIK3C2G*	rs26819	*PPIP5K2*
rs4385947	*PIK3C2G*	rs2477223	*NUDT3*	rs11044075	*PIK3C2G*	rs11202596	*PTEN*
rs17409120	*PIK3C2G*	rs2477232	*NUDT3*	rs11044082	*PIK3C2G*	rs11202607	*PTEN*
rs11044004	*PIK3C2G*	rs2495965	*NUDT3*	rs12822135	*PIK3C2G*	rs2248293	*PTEN*
rs7133666	*PIK3C2G*	rs2495966	*NUDT3*	rs11044084	*PIK3C2G*	rs10470165	*SYNJ1*
rs4534639	*PIK3C2G*	rs2495970	*NUDT3*	rs10841023	*PIK3C2G*	rs11702774	*SYNJ1*
rs12582971	*PIK3C2G*	rs2495971	*NUDT3*	rs10770359	*PIK3C2G*	rs12626242	*SYNJ1*
rs10505811	*PIK3C2G*	rs2495972	*NUDT3*	rs12581163	*PIK3C2G*	rs17694546	*SYNJ1*
rs9300118	*PIK3C2G*	rs2495975	*NUDT3*	rs10841025	*PIK3C2G*	rs1783099	*SYNJ1*
rs10160860	*PIK3C2G*	rs2495976	*NUDT3*	rs11044103	*PIK3C2G*	rs632324	*SYNJ1*
rs4609650	*PIK3C2G*	rs2495985	*NUDT3*	rs3813896	*PIK3C2G*	rs7279487	*SYNJ1*
rs11044026	*PIK3C2G*	rs2495990	*NUDT3*	rs12427286	*PIK3C2G*	rs845018	*SYNJ1*
rs10505810	*PIK3C2G*	rs2499669	*NUDT3*	rs9634063	*PIK3C2G*	rs10455936	*SYNJ2*
rs12309567	*PIK3C2G*	rs2499677	*NUDT3*	rs7308591	*PIK3C2G*	rs12202135	*SYNJ2*
rs10841019	*PIK3C2G*	rs2499682	*NUDT3*	rs1447406	*PIK3C2G*	rs12208248	*SYNJ2*
rs12297325	*PIK3C2G*	rs2499694	*NUDT3*	rs12371624	*PIK3C2G*	rs13217929	*SYNJ2*
rs4595599	*PIK3C2G*	rs2499711	*NUDT3*	rs7314398	*PIK3C2G*	rs1744178	*SYNJ2*
rs12422650	*PIK3C2G*	rs2499713	*NUDT3*	rs1868064	*PIK3C2G*	rs17489570	*SYNJ2*
rs11044058	*PIK3C2G*	rs2499724	*NUDT3*	rs1447408	*PIK3C2G*	rs1750040	*SYNJ2*
rs11044070	*PIK3C2G*	rs2499730	*NUDT3*	rs10743273	*PIK3C2G*	rs1750043	*SYNJ2*
rs12227441	*PIK3C2G*	rs2499733	*NUDT3*	rs1374670	*PIK3C2G*	rs1977356	*SYNJ2*
rs11044075	*PIK3C2G*	rs2499742	*NUDT3*	rs719568	*PIK3C2G*	rs2181190	*SYNJ2*
rs11044082	*PIK3C2G*	rs2499757	*NUDT3*	rs12312266	*PIK3C2G*	rs2295894	*SYNJ2*
rs12822135	*PIK3C2G*	rs2499762	*NUDT3*	rs2290044	*PIK3C2G*	rs2502620	*SYNJ2*
rs11044084	*PIK3C2G*	rs2499766	*NUDT3*	rs17411508	*PIK3C2G*	rs7768038	*SYNJ2*
rs10841023	*PIK3C2G*	rs2772372	*NUDT3*	rs2305220	*PIK3C2G*	rs9356200	*SYNJ2*
rs10770359	*PIK3C2G*	rs2894342	*NUDT3*	rs7957373	*PIK3C2G*	rs9365674	*SYNJ2*
rs12581163	*PIK3C2G*	rs2966	*NUDT3*	rs10505824	*PIK3C2G*	rs9654570	*SYNJ2*
rs10841025	*PIK3C2G*	rs3106192	*NUDT3*	rs11044165	*PIK3C2G*	rs10744720	*TPI1*
rs11044103	*PIK3C2G*	rs3116713	*NUDT3*	rs7969452	*PIK3C2G*		

rs3813896	*PIK3C2G*	rs3117324	*NUDT3*	rs11044171	*PIK3C2G*
rs12427286	*PIK3C2G*	rs3130014	*NUDT3*	rs12367676	*PIK3C2G*
rs9634063	*PIK3C2G*	rs3130100	*NUDT3*	rs17418422	*PIK3C2G*
rs7308591	*PIK3C2G*	rs3130257	*NUDT3*	rs1992838	*PIK3C2G*
rs1447406	*PIK3C2G*	rs3130267	*NUDT3*	rs10841036	*PIK3C2G*
rs12371624	*PIK3C2G*	rs3130276	*NUDT3*	rs1348928	*PIK3C2G*
rs7314398	*PIK3C2G*	rs368716	*NUDT3*	rs10841041	*PIK3C2G*
rs1868064	*PIK3C2G*	rs3748079	*NUDT3*	rs10841043	*PIK3C2G*
rs1447408	*PIK3C2G*	rs375555	*NUDT3*	rs12827287	*PIK3C2G*
rs10743273	*PIK3C2G*	rs3763260	*NUDT3*	rs12581078	*PIK3C2G*
rs1374670	*PIK3C2G*	rs3798560	*NUDT3*	rs17419409	*PIK3C2G*
rs719568	*PIK3C2G*	rs3806109	*NUDT3*	rs12821147	*PIK3C2G*
rs12312266	*PIK3C2G*	rs3818528	*NUDT3*	rs12818409	*PIK3C2G*
rs2290044	*PIK3C2G*	rs3818532	*NUDT3*	rs2277328	*PIK3C2G*
rs17411508	*PIK3C2G*	rs3887555	*NUDT3*	rs1816971	*PIK3C2G*
rs2305220	*PIK3C2G*	rs394199	*NUDT3*	rs7964182	*PIK3C2G*
rs11044155	*PIK3C2G*	rs3957165	*NUDT3*	rs10770372	*PIK3C2G*
rs7957373	*PIK3C2G*	rs396746	*NUDT3*	rs12825580	*PIK3C2G*
rs10505824	*PIK3C2G*	rs420361	*NUDT3*	rs10841048	*PIK3C2G*
rs11044165	*PIK3C2G*	rs4259245	*NUDT3*	rs10841049	*PIK3C2G*
rs7969452	*PIK3C2G*	rs4304152	*NUDT3*	rs11044223	*PIK3C2G*
rs11044171	*PIK3C2G*	rs442745	*NUDT3*	rs4764409	*PIK3C2G*
rs12367676	*PIK3C2G*	rs449242	*NUDT3*	rs666864	*PIK3C2G*
rs17418422	*PIK3C2G*	rs455567	*NUDT3*	rs621042	*PIK3C2G*
rs1992838	*PIK3C2G*	rs4559081	*NUDT3*	rs4763508	*PIK3C2G*
rs10841036	*PIK3C2G*	rs456261	*NUDT3*	rs644164	*PIK3C2G*
rs10841041	*PIK3C2G*	rs461964	*NUDT3*	rs11044232	*PIK3C2G*
rs10841043	*PIK3C2G*	rs464553	*NUDT3*	rs10743276	*PIK3C2G*
rs12827287	*PIK3C2G*	rs4711332	*NUDT3*	rs518345	*PIK3C2G*
rs12581078	*PIK3C2G*	rs4711338	*NUDT3*	rs531697	*PIK3C2G*
rs17419409	*PIK3C2G*	rs4711345	*NUDT3*	rs4764412	*PIK3C2G*
rs12821147	*PIK3C2G*	rs4711348	*NUDT3*	rs2931484	*PIK3C2G*
rs12818409	*PIK3C2G*	rs4711358	*NUDT3*	rs10734680	*PIK3C2G*
rs2277328	*PIK3C2G*	rs4711369	*NUDT3*	rs578947	*PIK3C2G*
rs1816971	*PIK3C2G*	rs4711374	*NUDT3*	rs982754	*PIK3C2G*
rs7964182	*PIK3C2G*	rs4713646	*NUDT3*	rs982754	*PIK3C2G*
rs10770372	*PIK3C2G*	rs4713653	*NUDT3*	rs6507451	*PIK3C3*
rs12825580	*PIK3C2G*	rs4713654	*NUDT3*	rs1944967	*PIK3C3*
rs10841048	*PIK3C2G*	rs4713668	*NUDT3*	rs9956832	*PIK3C3*
rs10841049	*PIK3C2G*	rs4713691	*NUDT3*	rs682408	*PIK3C3*
rs11044223	*PIK3C2G*	rs4713693	*NUDT3*	rs12954899	*PIK3C3*
rs4764409	*PIK3C2G*	rs4713699	*NUDT3*	rs3764459	*PIK3C3*
rs666864	*PIK3C2G*	rs4713704	*NUDT3*	rs7238178	*PIK3C3*
rs621042	*PIK3C2G*	rs4713711	*NUDT3*	rs1941526	*PIK3C3*

rs4763508	*PIK3C2G*	rs4713731	*NUDT3*	rs1941526	*PIK3C3*
rs644164	*PIK3C2G*	rs4713750	*NUDT3*	rs6807293	*PIK3CA*
rs11044232	*PIK3C2G*	rs4713755	*NUDT3*	rs7621329	*PIK3CA*
rs11044233	*PIK3C2G*	rs471942	*NUDT3*	rs2699905	*PIK3CA*
rs518345	*PIK3C2G*	rs493871	*NUDT3*	rs7641889	*PIK3CA*
rs531697	*PIK3C2G*	rs498114	*NUDT3*	rs6443624	*PIK3CA*
rs4764412	*PIK3C2G*	rs499384	*NUDT3*	rs7646409	*PIK3CA*
rs2931484	*PIK3C2G*	rs510153	*NUDT3*	rs2677760	*PIK3CA*
rs10734680	*PIK3C2G*	rs513349	*NUDT3*	rs13082485	*PIK3CA*
rs578947	*PIK3C2G*	rs542441	*NUDT3*	rs7614305	*PIK3CA*
rs982754	*PIK3C2G*	rs5745568	*NUDT3*	rs2230461	*PIK3CA*
rs982754	*PIK3C2G*	rs594223	*NUDT3*	rs13320527	*PIK3CA*
rs6507451	*PIK3C3*	rs597386	*NUDT3*	rs1607237	*PIK3CA*
rs1944967	*PIK3C3*	rs622917	*NUDT3*	rs9878820	*PIK3CB*
rs9956832	*PIK3C3*	rs6457730	*NUDT3*	rs531577	*PIK3CB*
rs682408	*PIK3C3*	rs6457738	*NUDT3*	rs693293	*PIK3CB*
rs12954899	*PIK3C3*	rs6457740	*NUDT3*	rs497900	*PIK3CB*
rs3764459	*PIK3C3*	rs6457742	*NUDT3*	rs361059	*PIK3CB*
rs7238178	*PIK3C3*	rs6457744	*NUDT3*	rs10513055	*PIK3CB*
rs1941526	*PIK3C3*	rs6457748	*NUDT3*	rs558905	*PIK3CB*
rs6807293	*PIK3CA*	rs649775	*NUDT3*	rs500687	*PIK3CB*
rs7621329	*PIK3CA*	rs652049	*NUDT3*	rs361068	*PIK3CB*
rs2699905	*PIK3CA*	rs68191	*NUDT3*	rs9430506	*PIK3CD*
rs7641889	*PIK3CA*	rs684119	*NUDT3*	rs4129341	*PIK3CD*
rs6443624	*PIK3CA*	rs6900980	*NUDT3*	rs6540985	*PIK3CD*
rs7646409	*PIK3CA*	rs6904716	*NUDT3*	rs4240896	*PIK3CD*
rs2677760	*PIK3CA*	rs6905353	*NUDT3*	rs4240910	*PIK3CD*
rs13082485	*PIK3CA*	rs6908522	*NUDT3*	rs12075554	*PIK3CD*
rs7614305	*PIK3CA*	rs6914422	*NUDT3*	rs6541017	*PIK3CD*
rs13320527	*PIK3CA*	rs6919321	*NUDT3*	rs12569008	*PIK3CD*
rs1607237	*PIK3CA*	rs6921366	*NUDT3*	rs193740	*PIK3CG*
rs1607237	*PIK3CA*	rs6922119	*NUDT3*	rs1724262	*PIK3CG*
rs9878820	*PIK3CB*	rs6926872	*NUDT3*	rs1636808	*PIK3CG*
rs531577	*PIK3CB*	rs6929774	*NUDT3*	rs849375	*PIK3CG*
rs693293	*PIK3CB*	rs6933607	*NUDT3*	rs849376	*PIK3CG*
rs497900	*PIK3CB*	rs6934013	*NUDT3*	rs17153527	*PIK3CG*
rs361059	*PIK3CB*	rs6935041	*NUDT3*	rs849380	*PIK3CG*
rs10513055	*PIK3CB*	rs6935686	*NUDT3*	rs757902	*PIK3CG*
rs558905	*PIK3CB*	rs6939337	*NUDT3*	rs757903	*PIK3CG*
rs500687	*PIK3CB*	rs729424	*NUDT3*	rs6956373	*PIK3CG*
rs361068	*PIK3CB*	rs745222	*NUDT3*	rs4727666	*PIK3CG*
rs361068	*PIK3CB*	rs755495	*NUDT3*	rs1526083	*PIK3CG*
rs9430506	*PIK3CD*	rs767896	*NUDT3*	rs849367	*PIK3CG*
rs4129341	*PIK3CD*	rs769051	*NUDT3*	rs849370	*PIK3CG*

rs6540985	*PIK3CD*	rs7739273	*NUDT3*	rs4730205	*PIK3CG*
rs4240896	*PIK3CD*	rs7742369	*NUDT3*	rs11766675	*PIK3CG*
rs4240910	*PIK3CD*	rs7748976	*NUDT3*	rs849412	*PIK3CG*
rs12075554	*PIK3CD*	rs7751882	*NUDT3*	rs6435435	*PIKFYVE*
rs6541017	*PIK3CD*	rs7763695	*NUDT3*	rs3769521	*PIKFYVE*
rs6541017	*PIK3CD*	rs7765294	*NUDT3*	rs17652774	*PIKFYVE*
rs193740	*PIK3CG*	rs7769838	*NUDT3*	rs6746926	*PIKFYVE*
rs1724262	*PIK3CG*	rs7771877	*NUDT3*	rs1584200	*PIKFYVE*
rs1636808	*PIK3CG*	rs7772005	*NUDT3*	rs10189031	*PIKFYVE*
rs849375	*PIK3CG*	rs7772436	*NUDT3*	rs999890	*PIKFYVE*
rs849376	*PIK3CG*	rs7774407	*NUDT3*	rs1866046	*PIKFYVE*
rs17153527	*PIK3CG*	rs86715	*NUDT3*	rs2289171	*PIKFYVE*
rs849380	*PIK3CG*	rs914813	*NUDT3*	rs10190458	*PIKFYVE*
rs757902	*PIK3CG*	rs9277965	*NUDT3*	rs13407268	*PIKFYVE*
rs757903	*PIK3CG*	rs9278019	*NUDT3*	rs10177810	*PIKFYVE*
rs6956373	*PIK3CG*	rs9296097	*NUDT3*	rs4673402	*PIKFYVE*
rs4727666	*PIK3CG*	rs9296100	*NUDT3*	rs4675764	*PIKFYVE*
rs1526083	*PIK3CG*	rs9348927	*NUDT3*	rs12355895	*PIP4K2A*
rs849367	*PIK3CG*	rs9366829	*NUDT3*	rs7899156	*PIP4K2A*
rs849370	*PIK3CG*	rs9366831	*NUDT3*	rs4532931	*PIP4K2A*
rs4730205	*PIK3CG*	rs9368781	*NUDT3*	rs10828316	*PIP4K2A*
rs11766675	*PIK3CG*	rs9368782	*NUDT3*	rs10828317	*PIP4K2A*
rs849412	*PIK3CG*	rs9368783	*NUDT3*	rs943189	*PIP4K2A*
rs849412	*PIK3CG*	rs937035	*NUDT3*	rs7094131	*PIP4K2A*
rs6435435	*PIKFYVE*	rs937039	*NUDT3*	rs7088318	*PIP4K2A*
rs3769521	*PIKFYVE*	rs9380374	*NUDT3*	rs3793753	*PIP4K2A*
rs17652774	*PIKFYVE*	rs9380376	*NUDT3*	rs12253847	*PIP4K2A*
rs6746926	*PIKFYVE*	rs9380409	*NUDT3*	rs7919839	*PIP4K2A*
rs1584200	*PIKFYVE*	rs9394145	*NUDT3*	rs1062190	*PIP4K2A*
rs10189031	*PIKFYVE*	rs9394167	*NUDT3*	rs11013053	*PIP4K2A*
rs999890	*PIKFYVE*	rs9394169	*NUDT3*	rs7071450	*PIP4K2A*
rs1866046	*PIKFYVE*	rs9394174	*NUDT3*	rs7075433	*PIP4K2A*
rs2289171	*PIKFYVE*	rs9394178	*NUDT3*	rs12357384	*PIP4K2A*
rs10190458	*PIKFYVE*	rs9394180	*NUDT3*	rs7912144	*PIP4K2A*
rs13407268	*PIKFYVE*	rs942496	*NUDT3*	rs1409396	*PIP4K2A*
rs10177810	*PIKFYVE*	rs942510	*NUDT3*	rs10764342	*PIP4K2A*
rs4673402	*PIKFYVE*	rs942637	*NUDT3*	rs10764344	*PIP4K2A*
rs4675764	*PIKFYVE*	rs9461868	*NUDT3*	rs12773197	*PIP4K2A*
rs12355895	*PIP4K2A*	rs9461882	*NUDT3*	rs2765993	*PIP4K2A*
rs7899156	*PIP4K2A*	rs9461917	*NUDT3*	rs2559524	*PIP4K2A*
rs4532931	*PIP4K2A*	rs9461924	*NUDT3*	rs7093543	*PIP4K2A*
rs10828316	*PIP4K2A*	rs9461926	*NUDT3*	rs2559523	*PIP4K2A*
rs10828317	*PIP4K2A*	rs9461937	*NUDT3*	rs1778353	*PIP4K2A*
rs943189	*PIP4K2A*	rs9469442	*NUDT3*	rs11013067	*PIP4K2A*

rs7094131	*PIP4K2A*	rs9469529	*NUDT3*	rs1750758	*PIP4K2A*
rs7088318	*PIP4K2A*	rs9469583	*NUDT3*	rs1778302	*PIP4K2A*
rs3793753	*PIP4K2A*	rs9469598	*NUDT3*	rs11013069	*PIP4K2A*
rs12253847	*PIP4K2A*	rs9469619	*NUDT3*	rs1750761	*PIP4K2A*
rs7919839	*PIP4K2A*	rs9469655	*NUDT3*	rs1627983	*PIP4K2A*
rs1062190	*PIP4K2A*	rs9469761	*NUDT3*	rs1778317	*PIP4K2A*
rs11013053	*PIP4K2A*	rs999943	*NUDT3*	rs1750770	*PIP4K2A*
rs7071450	*PIP4K2A*	rs11107007	*NUDT4*	rs1539628	*PIP4K2A*
rs7075433	*PIP4K2A*	rs11107008	*NUDT4*	rs943196	*PIP4K2A*
rs12357384	*PIP4K2A*	rs12597	*NUDT4*	rs1750775	*PIP4K2A*
rs7912144	*PIP4K2A*	rs12816436	*NUDT4*	rs7075499	*PIP4K2A*
rs1409396	*PIP4K2A*	rs17790482	*NUDT4*	rs12098721	*PIP4K2A*
rs10764344	*PIP4K2A*	rs17837158	*NUDT4*	rs10508651	*PIP4K2A*
rs12773197	*PIP4K2A*	rs4247307	*NUDT4*	rs11013086	*PIP4K2A*
rs2765993	*PIP4K2A*	rs4761517	*NUDT4*	rs1171509	*PIP4K2A*
rs2559524	*PIP4K2A*	rs7487813	*NUDT4*	rs16922578	*PIP4K2A*
rs7093543	*PIP4K2A*	rs7973701	*NUDT4*	rs10159847	*PIP4K2A*
rs2559523	*PIP4K2A*	rs7977140	*NUDT4*	rs11597156	*PIP4K2A*
rs1778353	*PIP4K2A*	rs10444068	*PI4K2A*	rs11013095	*PIP4K2A*
rs11013067	*PIP4K2A*	rs10786364	*PI4K2A*	rs1891877	*PIP4K2A*
rs1750758	*PIP4K2A*	rs11189310	*PI4K2A*	rs11592199	*PIP4K2A*
rs1778302	*PIP4K2A*	rs11189321	*PI4K2A*	rs11013103	*PIP4K2A*
rs11013069	*PIP4K2A*	rs2065672	*PI4K2A*	rs7084115	*PIP4K2A*
rs1750761	*PIP4K2A*	rs3890727	*PI4K2A*	rs7094187	*PIP4K2A*
rs1627983	*PIP4K2A*	rs4919128	*PI4K2A*	rs6482235	*PIP4K2A*
rs1778317	*PIP4K2A*	rs6584138	*PI4K2A*	rs764190	*PIP4K2B*
rs1750770	*PIP4K2A*	rs11737332	*PI4K2B*	rs228285	*PIP4K2B*
rs1539628	*PIP4K2A*	rs3115231	*PI4K2B*	rs2338115	*PIP4K2B*
rs943196	*PIP4K2A*	rs313533	*PI4K2B*	rs2075061	*PIP4K2B*
rs1750775	*PIP4K2A*	rs313541	*PI4K2B*	rs7207178	*PIP4K2B*
rs7075499	*PIP4K2A*	rs313548	*PI4K2B*	rs12453325	*PIP4K2B*
rs12098721	*PIP4K2A*	rs313566	*PI4K2B*	rs11653487	*PIP4K2B*
rs10508651	*PIP4K2A*	rs3796780	*PI4K2B*	rs228307	*PIP4K2B*
rs11013086	*PIP4K2A*	rs7661189	*PI4K2B*	rs115327	*PIP4K2B*
rs1171509	*PIP4K2A*	rs1558657	*PI4KA*	rs228249	*PIP4K2B*
rs16922578	*PIP4K2A*	rs165598	*PI4KA*	rs626866	*PIP4K2B*
rs10159847	*PIP4K2A*	rs165793	*PI4KA*	rs626866	*PIP4K2B*
rs11597156	*PIP4K2A*	rs165862	*PI4KA*	rs11172254	*PIP4K2C*
rs11013095	*PIP4K2A*	rs165924	*PI4KA*	rs775250	*PIP4K2C*
rs1891877	*PIP4K2A*	rs178051	*PI4KA*	rs775251	*PIP4K2C*
rs11592199	*PIP4K2A*	rs178058	*PI4KA*	rs812315	*PIP4K2C*
rs11013103	*PIP4K2A*	rs178070	*PI4KA*	rs812315	*PIP4K2C*
rs7084115	*PIP4K2A*	rs17820181	*PI4KA*	rs4970944	*PIP5K1A*
rs7094187	*PIP4K2A*	rs2072513	*PI4KA*	rs4520422	*PIP5K1A*

rs6482235	*PIP4K2A*	rs4820579	*PI4KA*	rs7532935	*PIP5K1A*
rs6482235	*PIP4K2A*	rs4822606	*PI4KA*	rs1014237	*PIP5K1B*
rs764190	*PIP4K2B*	rs6004148	*PI4KA*	rs1014807	*PIP5K1B*
rs228285	*PIP4K2B*	rs9608386	*PI4KA*	rs11143417	*PIP5K1B*
rs2338115	*PIP4K2B*	rs9620548	*PI4KA*	rs1412990	*PIP5K1B*
rs2075061	*PIP4K2B*	rs1056847	*PI4KB*	rs1107108	*PIP5K1B*
rs12453325	*PIP4K2B*	rs1752382	*PI4KB*	rs3812537	*PIP5K1B*
rs11653487	*PIP4K2B*	rs1752388	*PI4KB*	rs2151418	*PIP5K1B*
rs228307	*PIP4K2B*	rs2031797	*PI4KB*	rs10781117	*PIP5K1B*
rs115327	*PIP4K2B*	rs2298265	*PI4KB*	rs10121993	*PIP5K1B*
rs228249	*PIP4K2B*	rs4971030	*PI4KB*	rs10125301	*PIP5K1B*
rs626866	*PIP4K2B*	rs5022636	*PI4KB*	rs4745231	*PIP5K1B*
rs11172254	*PIP4K2C*	rs11604561	*PIK3C2A*	rs13296679	*PIP5K1B*
rs775250	*PIP4K2C*	rs1541533	*PIK3C2A*	rs7044422	*PIP5K1B*
rs775251	*PIP4K2C*	rs2040859	*PIK3C2A*	rs7866673	*PIP5K1B*
rs812315	*PIP4K2C*	rs3950680	*PIK3C2A*	rs12380573	*PIP5K1B*
rs4970944	*PIP5K1A*	rs621246	*PIK3C2A*	rs12686355	*PIP5K1B*
rs10749660	*PIP5K1A*	rs7946010	*PIK3C2A*	rs10735625	*PIP5K1B*
rs4520422	*PIP5K1A*	rs11240748	*PIK3C2B*	rs2151414	*PIP5K1B*
rs7532935	*PIP5K1A*	rs12031854	*PIK3C2B*	rs8114	*PIP5K1B*
rs7532935	*PIP5K1A*	rs12061474	*PIK3C2B*	rs12339235	*PIP5K1B*
rs1014237	*PIP5K1B*	rs12119503	*PIK3C2B*	rs7018552	*PIP5K1B*
rs1014807	*PIP5K1B*	rs16853737	*PIK3C2B*	rs10511963	*PIP5K1B*
rs11143417	*PIP5K1B*	rs17334387	*PIK3C2B*	rs10869335	*PIP5K1B*
rs1412990	*PIP5K1B*	rs2137255	*PIK3C2B*	rs1556751	*PIP5K1B*
rs1107108	*PIP5K1B*	rs2271415	*PIK3C2B*	rs4745296	*PIP5K1B*
rs3812537	*PIP5K1B*	rs2271421	*PIK3C2B*	rs17391840	*PIP5K1B*
rs2151418	*PIP5K1B*	rs2271427	*PIK3C2B*	rs1889150	*PIP5K1B*
rs10781117	*PIP5K1B*	rs2999484	*PIK3C2B*	rs10869396	*PIP5K1B*
rs10121993	*PIP5K1B*	rs2999487	*PIK3C2B*	rs4745321	*PIP5K1B*
rs10125301	*PIP5K1B*	rs3014637	*PIK3C2B*	rs10869400	*PIP5K1B*
rs4745231	*PIP5K1B*	rs3106366	*PIK3C2B*	rs10869420	*PIP5K1B*
rs13296679	*PIP5K1B*	rs3747633	*PIK3C2B*	rs7041004	*PIP5K1B*
rs7044422	*PIP5K1B*	rs3747636	*PIK3C2B*	rs6560397	*PIP5K1B*
rs963707	*PIP5K1B*	rs4951373	*PIK3C2B*	rs11144027	*PIP5K1B*
rs7866673	*PIP5K1B*	rs6594014	*PIK3C2B*	rs17392931	*PIP5K1B*
rs12380573	*PIP5K1B*	rs7556371	*PIK3C2B*	rs11144133	*PIP5K1B*
rs12686355	*PIP5K1B*	rs10160860	*PIK3C2G*	rs4237270	*PIP5K1B*
rs10735625	*PIP5K1B*	rs10505810	*PIK3C2G*	rs4745375	*PIP5K1B*
rs2151414	*PIP5K1B*	rs10505811	*PIK3C2G*	rs883952	*PIP5K1B*
rs8114	*PIP5K1B*	rs10505824	*PIK3C2G*	rs17058792	*PIP5K1B*
rs1412988	*PIP5K1B*	rs10734680	*PIK3C2G*	rs1414954	*PIP5K1B*
rs12339235	*PIP5K1B*	rs10743273	*PIK3C2G*	rs2152649	*PIP5K1B*
rs10511963	*PIP5K1B*	rs10743276	*PIK3C2G*	rs10869538	*PIP5K1B*

rs10869335	*PIP5K1B*	rs10770333	*PIK3C2G*	rs6560444	*PIP5K1B*
rs1556751	*PIP5K1B*	rs10770359	*PIK3C2G*	rs4745402	*PIP5K1B*
rs4745296	*PIP5K1B*	rs10770372	*PIK3C2G*	rs10746974	*PIP5K1B*
rs17391840	*PIP5K1B*	rs10841017	*PIK3C2G*	rs10114872	*PIP5K1B*
rs1889150	*PIP5K1B*	rs10841019	*PIK3C2G*	rs12349586	*PIP5K1B*
rs10869396	*PIP5K1B*	rs10841023	*PIK3C2G*	rs1541084	*PIP5K1B*
rs4745321	*PIP5K1B*	rs10841025	*PIK3C2G*	rs10117043	*PIP5K1B*
rs10869400	*PIP5K1B*	rs10841036	*PIK3C2G*	rs10781306	*PIP5K1B*
rs10869420	*PIP5K1B*	rs10841041	*PIK3C2G*	rs7048825	*PIP5K1B*
rs7041004	*PIP5K1B*	rs10841043	*PIK3C2G*	rs4745451	*PIP5K1B*
rs6560397	*PIP5K1B*	rs10841048	*PIK3C2G*	rs4745466	*PIP5K1B*
rs11144027	*PIP5K1B*	rs10841049	*PIK3C2G*	rs10746983	*PIP5K1B*
rs17392931	*PIP5K1B*	rs11044004	*PIK3C2G*	rs883751	*PIP5K1B*
rs11144133	*PIP5K1B*	rs11044026	*PIK3C2G*	rs2871223	*PIP5K1B*
rs4237270	*PIP5K1B*	rs11044034	*PIK3C2G*	rs10781329	*PIP5K1B*
rs4745375	*PIP5K1B*	rs11044058	*PIK3C2G*	rs10869686	*PIP5K1B*
rs883952	*PIP5K1B*	rs11044070	*PIK3C2G*	rs872077	*PIP5K1B*
rs17058792	*PIP5K1B*	rs11044075	*PIK3C2G*	rs4745514	*PIP5K1B*
rs12686693	*PIP5K1B*	rs11044082	*PIK3C2G*	rs4745520	*PIP5K1B*
rs17058884	*PIP5K1B*	rs11044084	*PIK3C2G*	rs9314841	*PIP5K1B*
rs1414954	*PIP5K1B*	rs11044103	*PIK3C2G*	rs2271875	*PIP5K1C*
rs7859638	*PIP5K1B*	rs11044165	*PIK3C2G*	rs4807493	*PIP5K1C*
rs2152649	*PIP5K1B*	rs11044171	*PIK3C2G*	rs3746124	*PIP5K1C*
rs10869538	*PIP5K1B*	rs11044223	*PIK3C2G*	rs2286435	*PIP5K1C*
rs6560444	*PIP5K1B*	rs11044232	*PIK3C2G*	rs2270083	*PIP5K1C*
rs4745402	*PIP5K1B*	rs12227441	*PIK3C2G*	rs757454	*PIP5K1C*
rs10746974	*PIP5K1B*	rs12297325	*PIK3C2G*	rs8109485	*PIP5K1C*
rs10114872	*PIP5K1B*	rs12299348	*PIK3C2G*	rs11672559	*PIP5K1C*
rs12349586	*PIP5K1B*	rs12309567	*PIK3C2G*	rs1476592	*PIP5K1C*
rs1541084	*PIP5K1B*	rs12312266	*PIK3C2G*	rs516423	*PIP5K1C*
rs10117043	*PIP5K1B*	rs12367676	*PIK3C2G*	rs516423	*PIP5K1C*
rs10781306	*PIP5K1B*	rs12371624	*PIK3C2G*	rs3739821	*PIP5KL1*
rs7048825	*PIP5K1B*	rs12427286	*PIK3C2G*	rs7859	*PIP5KL1*
rs4745451	*PIP5K1B*	rs12581078	*PIK3C2G*	rs6478803	*PIP5KL1*
rs12378665	*PIP5K1B*	rs12581163	*PIK3C2G*	rs6055550	*PLCB1*
rs4745466	*PIP5K1B*	rs12582971	*PIK3C2G*	rs6086343	*PLCB1*
rs10746983	*PIP5K1B*	rs12818409	*PIK3C2G*	rs6086345	*PLCB1*
rs883751	*PIP5K1B*	rs12821147	*PIK3C2G*	rs6118073	*PLCB1*
rs2871223	*PIP5K1B*	rs12822135	*PIK3C2G*	rs2179984	*PLCB1*
rs10781329	*PIP5K1B*	rs12825580	*PIK3C2G*	rs6118075	*PLCB1*
rs10869686	*PIP5K1B*	rs12827287	*PIK3C2G*	rs6086346	*PLCB1*
rs872077	*PIP5K1B*	rs1348928	*PIK3C2G*	rs6039040	*PLCB1*
rs4745514	*PIP5K1B*	rs1374670	*PIK3C2G*	rs6086348	*PLCB1*
rs4745520	*PIP5K1B*	rs1447406	*PIK3C2G*	rs6118083	*PLCB1*

rs9314841	*PIP5K1B*	rs1447408	*PIK3C2G*	rs4142365	*PLCB1*
rs9314841	*PIP5K1B*	rs17409120	*PIK3C2G*	rs6140549	*PLCB1*
rs4807492	*PIP5K1C*	rs17411508	*PIK3C2G*	rs6055562	*PLCB1*
rs2271875	*PIP5K1C*	rs17418422	*PIK3C2G*	rs8114499	*PLCB1*
rs4807493	*PIP5K1C*	rs17419409	*PIK3C2G*	rs6039049	*PLCB1*
rs3746124	*PIP5K1C*	rs1816971	*PIK3C2G*	rs4816047	*PLCB1*
rs2270083	*PIP5K1C*	rs1868064	*PIK3C2G*	rs2327025	*PLCB1*
rs757454	*PIP5K1C*	rs1992838	*PIK3C2G*	rs6055578	*PLCB1*
rs8109485	*PIP5K1C*	rs2277328	*PIK3C2G*	rs978266	*PLCB1*
rs11672559	*PIP5K1C*	rs2290044	*PIK3C2G*	rs728213	*PLCB1*
rs1476592	*PIP5K1C*	rs2305220	*PIK3C2G*	rs7271063	*PLCB1*
rs740873	*PIP5K1C*	rs2931484	*PIK3C2G*	rs6055594	*PLCB1*
rs6781	*PIP5KL1*	rs3813896	*PIK3C2G*	rs6055601	*PLCB1*
rs3739821	*PIP5KL1*	rs4237974	*PIK3C2G*	rs6055603	*PLCB1*
rs7859	*PIP5KL1*	rs4369463	*PIK3C2G*	rs6055625	*PLCB1*
rs6478803	*PIP5KL1*	rs4385947	*PIK3C2G*	rs2423354	*PLCB1*
rs6478803	*PIP5KL1*	rs4534639	*PIK3C2G*	rs12480099	*PLCB1*
rs6055550	*PLCB1*	rs4595599	*PIK3C2G*	rs6077326	*PLCB1*
rs6086343	*PLCB1*	rs4609650	*PIK3C2G*	rs6055652	*PLCB1*
rs6086345	*PLCB1*	rs4763508	*PIK3C2G*	rs8118127	*PLCB1*
rs6118073	*PLCB1*	rs4764409	*PIK3C2G*	rs6077332	*PLCB1*
rs2179984	*PLCB1*	rs4764412	*PIK3C2G*	rs4813853	*PLCB1*
rs6118075	*PLCB1*	rs518345	*PIK3C2G*	rs4399790	*PLCB1*
rs6086346	*PLCB1*	rs531697	*PIK3C2G*	rs6133556	*PLCB1*
rs6039040	*PLCB1*	rs578947	*PIK3C2G*	rs10485722	*PLCB1*
rs6086348	*PLCB1*	rs621042	*PIK3C2G*	rs6086374	*PLCB1*
rs6118083	*PLCB1*	rs644164	*PIK3C2G*	rs6055685	*PLCB1*
rs4142365	*PLCB1*	rs666864	*PIK3C2G*	rs4813854	*PLCB1*
rs6140549	*PLCB1*	rs7133666	*PIK3C2G*	rs6039104	*PLCB1*
rs6055562	*PLCB1*	rs719568	*PIK3C2G*	rs1534897	*PLCB1*
rs8114499	*PLCB1*	rs7308591	*PIK3C2G*	rs6140561	*PLCB1*
rs6039049	*PLCB1*	rs7314398	*PIK3C2G*	rs1474581	*PLCB1*
rs4816047	*PLCB1*	rs7957373	*PIK3C2G*	rs1474937	*PLCB1*
rs2327025	*PLCB1*	rs7964182	*PIK3C2G*	rs995040	*PLCB1*
rs6055578	*PLCB1*	rs7969452	*PIK3C2G*	rs2179138	*PLCB1*
rs978266	*PLCB1*	rs9300118	*PIK3C2G*	rs6039107	*PLCB1*
rs728213	*PLCB1*	rs9634063	*PIK3C2G*	rs6039109	*PLCB1*
rs7271063	*PLCB1*	rs982754	*PIK3C2G*	rs6140562	*PLCB1*
rs6055594	*PLCB1*	rs12954899	*PIK3C3*	rs727684	*PLCB1*
rs6055601	*PLCB1*	rs1540057	*PIK3C3*	rs2294259	*PLCB1*
rs6055603	*PLCB1*	rs1941526	*PIK3C3*	rs6133564	*PLCB1*
rs6055625	*PLCB1*	rs1944967	*PIK3C3*	rs6133566	*PLCB1*
rs2423354	*PLCB1*	rs3764459	*PIK3C3*	rs8125486	*PLCB1*
rs12480099	*PLCB1*	rs6507451	*PIK3C3*	rs6133567	*PLCB1*

rs6077326	*PLCB1*	rs670429	*PIK3C3*	rs1033684	*PLCB1*
rs6055652	*PLCB1*	rs682408	*PIK3C3*	rs2719807	*PLCB1*
rs8118127	*PLCB1*	rs7238178	*PIK3C3*	rs10485723	*PLCB1*
rs6077332	*PLCB1*	rs9956832	*PIK3C3*	rs2745755	*PLCB1*
rs4813853	*PLCB1*	rs13082485	*PIK3CA*	rs17431073	*PLCB1*
rs4399790	*PLCB1*	rs13320527	*PIK3CA*	rs2719804	*PLCB1*
rs6133556	*PLCB1*	rs1607237	*PIK3CA*	rs6133573	*PLCB1*
rs10485722	*PLCB1*	rs2677760	*PIK3CA*	rs742615	*PLCB1*
rs6086374	*PLCB1*	rs2699905	*PIK3CA*	rs6086402	*PLCB1*
rs6055685	*PLCB1*	rs3729680	*PIK3CA*	rs2745772	*PLCB1*
rs4813854	*PLCB1*	rs6443624	*PIK3CA*	rs2662999	*PLCB1*
rs6039104	*PLCB1*	rs6807293	*PIK3CA*	rs2745776	*PLCB1*
rs1534897	*PLCB1*	rs7614305	*PIK3CA*	rs6077350	*PLCB1*
rs6140561	*PLCB1*	rs7621329	*PIK3CA*	rs6055748	*PLCB1*
rs1474581	*PLCB1*	rs7641889	*PIK3CA*	rs2719774	*PLCB1*
rs1474937	*PLCB1*	rs7646409	*PIK3CA*	rs2719776	*PLCB1*
rs995040	*PLCB1*	rs10513055	*PIK3CB*	rs13037679	*PLCB1*
rs2179138	*PLCB1*	rs16848263	*PIK3CB*	rs2745787	*PLCB1*
rs6039107	*PLCB1*	rs361059	*PIK3CB*	rs1232783	*PLCB1*
rs6039109	*PLCB1*	rs361068	*PIK3CB*	rs1232782	*PLCB1*
rs6140562	*PLCB1*	rs497900	*PIK3CB*	rs1605791	*PLCB1*
rs727684	*PLCB1*	rs500687	*PIK3CB*	rs1005987	*PLCB1*
rs2294259	*PLCB1*	rs531577	*PIK3CB*	rs17347805	*PLCB1*
rs6133564	*PLCB1*	rs558905	*PIK3CB*	rs708931	*PLCB1*
rs6133566	*PLCB1*	rs6795773	*PIK3CB*	rs708925	*PLCB1*
rs8125486	*PLCB1*	rs693293	*PIK3CB*	rs1237829	*PLCB1*
rs6133567	*PLCB1*	rs9878820	*PIK3CB*	rs1232779	*PLCB1*
rs1033684	*PLCB1*	rs12075554	*PIK3CD*	rs2745756	*PLCB1*
rs2719807	*PLCB1*	rs12569008	*PIK3CD*	rs708920	*PLCB1*
rs10485723	*PLCB1*	rs4129341	*PIK3CD*	rs1935671	*PLCB1*
rs2745755	*PLCB1*	rs4240896	*PIK3CD*	rs1238232	*PLCB1*
rs17431073	*PLCB1*	rs4240910	*PIK3CD*	rs6140613	*PLCB1*
rs2719804	*PLCB1*	rs6540985	*PIK3CD*	rs708916	*PLCB1*
rs6133573	*PLCB1*	rs6541017	*PIK3CD*	rs2662985	*PLCB1*
rs742615	*PLCB1*	rs9430506	*PIK3CD*	rs742616	*PLCB1*
rs6086402	*PLCB1*	rs11766675	*PIK3CG*	rs708912	*PLCB1*
rs2745772	*PLCB1*	rs1526083	*PIK3CG*	rs2719795	*PLCB1*
rs2662999	*PLCB1*	rs1636808	*PIK3CG*	rs771945	*PLCB1*
rs2745776	*PLCB1*	rs17153527	*PIK3CG*	rs771944	*PLCB1*
rs6077350	*PLCB1*	rs1724262	*PIK3CG*	rs771941	*PLCB1*
rs6055748	*PLCB1*	rs193740	*PIK3CG*	rs227142	*PLCB1*
rs2719774	*PLCB1*	rs4727666	*PIK3CG*	rs227134	*PLCB1*
rs2719776	*PLCB1*	rs4730205	*PIK3CG*	rs8115925	*PLCB1*
rs13037679	*PLCB1*	rs6956373	*PIK3CG*	rs227133	*PLCB1*

rs2745787	*PLCB1*	rs757902	*PIK3CG*	rs227130	*PLCB1*
rs1232783	*PLCB1*	rs757903	*PIK3CG*	rs2221695	*PLCB1*
rs1232782	*PLCB1*	rs849367	*PIK3CG*	rs764440	*PLCB1*
rs1605791	*PLCB1*	rs849370	*PIK3CG*	rs6086458	*PLCB1*
rs1005987	*PLCB1*	rs849375	*PIK3CG*	rs764439	*PLCB1*
rs17347805	*PLCB1*	rs849376	*PIK3CG*	rs6140619	*PLCB1*
rs708931	*PLCB1*	rs849380	*PIK3CG*	rs17362299	*PLCB1*
rs708925	*PLCB1*	rs849412	*PIK3CG*	rs6086459	*PLCB1*
rs6140611	*PLCB1*	rs10177810	*PIKFYVE*	rs4471960	*PLCB1*
rs1237829	*PLCB1*	rs10189031	*PIKFYVE*	rs10485724	*PLCB1*
rs1232779	*PLCB1*	rs10190458	*PIKFYVE*	rs2423356	*PLCB1*
rs2745756	*PLCB1*	rs13407268	*PIKFYVE*	rs1342585	*PLCB1*
rs708920	*PLCB1*	rs1584200	*PIKFYVE*	rs4813863	*PLCB1*
rs1935671	*PLCB1*	rs16840827	*PIKFYVE*	rs2327070	*PLCB1*
rs1238232	*PLCB1*	rs16841092	*PIKFYVE*	rs6108142	*PLCB1*
rs6140613	*PLCB1*	rs17652774	*PIKFYVE*	rs6055853	*PLCB1*
rs708916	*PLCB1*	rs1866046	*PIKFYVE*	rs6055858	*PLCB1*
rs2662985	*PLCB1*	rs2118297	*PIKFYVE*	rs1534968	*PLCB1*
rs742616	*PLCB1*	rs2289171	*PIKFYVE*	rs6140629	*PLCB1*
rs708912	*PLCB1*	rs3769521	*PIKFYVE*	rs2050090	*PLCB1*
rs2719795	*PLCB1*	rs4673402	*PIKFYVE*	rs6118219	*PLCB1*
rs771945	*PLCB1*	rs4675764	*PIKFYVE*	rs6086477	*PLCB1*
rs771944	*PLCB1*	rs6435435	*PIKFYVE*	rs2423360	*PLCB1*
rs771941	*PLCB1*	rs6746926	*PIKFYVE*	rs2423361	*PLCB1*
rs227142	*PLCB1*	rs7578972	*PIKFYVE*	rs1569604	*PLCB1*
rs227134	*PLCB1*	rs994696	*PIKFYVE*	rs722665	*PLCB1*
rs8115925	*PLCB1*	rs999890	*PIKFYVE*	rs6118234	*PLCB1*
rs227133	*PLCB1*	rs10159847	*PIP4K2A*	rs2143205	*PLCB1*
rs227130	*PLCB1*	rs10508651	*PIP4K2A*	rs2423363	*PLCB1*
rs2221695	*PLCB1*	rs1062190	*PIP4K2A*	rs2064272	*PLCB1*
rs764440	*PLCB1*	rs10764342	*PIP4K2A*	rs2423364	*PLCB1*
rs6086458	*PLCB1*	rs10764344	*PIP4K2A*	rs2423366	*PLCB1*
rs764439	*PLCB1*	rs10828316	*PIP4K2A*	rs6055889	*PLCB1*
rs6140619	*PLCB1*	rs10828317	*PIP4K2A*	rs2179440	*PLCB1*
rs17362299	*PLCB1*	rs11013053	*PIP4K2A*	rs6086490	*PLCB1*
rs6086459	*PLCB1*	rs11013067	*PIP4K2A*	rs3817881	*PLCB1*
rs4471960	*PLCB1*	rs11013069	*PIP4K2A*	rs11087808	*PLCB1*
rs10485724	*PLCB1*	rs11013086	*PIP4K2A*	rs6086491	*PLCB1*
rs2423356	*PLCB1*	rs11013095	*PIP4K2A*	rs6039189	*PLCB1*
rs1342585	*PLCB1*	rs11013103	*PIP4K2A*	rs17436253	*PLCB1*
rs4813863	*PLCB1*	rs11592199	*PIP4K2A*	rs6086493	*PLCB1*
rs2327070	*PLCB1*	rs11597156	*PIP4K2A*	rs6086495	*PLCB1*
rs6055853	*PLCB1*	rs1171509	*PIP4K2A*	rs2206423	*PLCB1*
rs6055858	*PLCB1*	rs12098721	*PIP4K2A*	rs6039190	*PLCB1*

rs1534968	*PLCB1*	rs12253847	*PIP4K2A*	rs6039191	*PLCB1*
rs6140629	*PLCB1*	rs12355895	*PIP4K2A*	rs6108152	*PLCB1*
rs2050090	*PLCB1*	rs12357384	*PIP4K2A*	rs13040221	*PLCB1*
rs6118219	*PLCB1*	rs12773197	*PIP4K2A*	rs6118252	*PLCB1*
rs6086477	*PLCB1*	rs1409396	*PIP4K2A*	rs2103653	*PLCB1*
rs2423360	*PLCB1*	rs1539628	*PIP4K2A*	rs6055910	*PLCB1*
rs2423361	*PLCB1*	rs1627983	*PIP4K2A*	rs1883503	*PLCB1*
rs1569604	*PLCB1*	rs16922578	*PIP4K2A*	rs6055912	*PLCB1*
rs722665	*PLCB1*	rs16922601	*PIP4K2A*	rs6086511	*PLCB1*
rs6118234	*PLCB1*	rs16922629	*PIP4K2A*	rs6118257	*PLCB1*
rs2143205	*PLCB1*	rs1750761	*PIP4K2A*	rs12624339	*PLCB1*
rs2423363	*PLCB1*	rs1750770	*PIP4K2A*	rs2327046	*PLCB1*
rs2064272	*PLCB1*	rs1750775	*PIP4K2A*	rs6055922	*PLCB1*
rs2423364	*PLCB1*	rs1778302	*PIP4K2A*	rs6055923	*PLCB1*
rs2423366	*PLCB1*	rs1778317	*PIP4K2A*	rs6108159	*PLCB1*
rs6055889	*PLCB1*	rs1778353	*PIP4K2A*	rs6055926	*PLCB1*
rs2179440	*PLCB1*	rs1891877	*PIP4K2A*	rs6055927	*PLCB1*
rs6086490	*PLCB1*	rs2559523	*PIP4K2A*	rs6055928	*PLCB1*
rs3817881	*PLCB1*	rs2559524	*PIP4K2A*	rs6086518	*PLCB1*
rs11087808	*PLCB1*	rs2765993	*PIP4K2A*	rs6108160	*PLCB1*
rs6086491	*PLCB1*	rs3793753	*PIP4K2A*	rs6086525	*PLCB1*
rs6039189	*PLCB1*	rs4532931	*PIP4K2A*	rs6118262	*PLCB1*
rs17436253	*PLCB1*	rs6482235	*PIP4K2A*	rs6039206	*PLCB1*
rs6086493	*PLCB1*	rs7071450	*PIP4K2A*	rs4813865	*PLCB1*
rs6086495	*PLCB1*	rs7075433	*PIP4K2A*	rs2223538	*PLCB1*
rs2206423	*PLCB1*	rs7075499	*PIP4K2A*	rs6055944	*PLCB1*
rs6039190	*PLCB1*	rs7078735	*PIP4K2A*	rs6118268	*PLCB1*
rs6039191	*PLCB1*	rs7084115	*PIP4K2A*	rs6055951	*PLCB1*
rs6108152	*PLCB1*	rs7088318	*PIP4K2A*	rs8126112	*PLCB1*
rs13040221	*PLCB1*	rs7093543	*PIP4K2A*	rs6077396	*PLCB1*
rs6118252	*PLCB1*	rs7094131	*PIP4K2A*	rs8126070	*PLCB1*
rs2103653	*PLCB1*	rs7094187	*PIP4K2A*	rs4432538	*PLCB1*
rs6055910	*PLCB1*	rs7899156	*PIP4K2A*	rs4419296	*PLCB1*
rs1883503	*PLCB1*	rs7912144	*PIP4K2A*	rs6055958	*PLCB1*
rs6055912	*PLCB1*	rs7914338	*PIP4K2A*	rs2223837	*PLCB1*
rs6086511	*PLCB1*	rs7919839	*PIP4K2A*	rs6086543	*PLCB1*
rs6118257	*PLCB1*	rs943189	*PIP4K2A*	rs12053642	*PLCB1*
rs12624339	*PLCB1*	rs943196	*PIP4K2A*	rs6039211	*PLCB1*
rs2327046	*PLCB1*	rs115327	*PIP4K2B*	rs1018443	*PLCB1*
rs6055922	*PLCB1*	rs11653487	*PIP4K2B*	rs6140671	*PLCB1*
rs6055923	*PLCB1*	rs12453325	*PIP4K2B*	rs6055990	*PLCB1*
rs6108159	*PLCB1*	rs2075061	*PIP4K2B*	rs6108174	*PLCB1*
rs6055926	*PLCB1*	rs228249	*PIP4K2B*	rs1033566	*PLCB1*
rs6055927	*PLCB1*	rs228285	*PIP4K2B*	rs6055995	*PLCB1*

rs6055928	*PLCB1*	rs228307	*PIP4K2B*	rs6056006	*PLCB1*
rs6086518	*PLCB1*	rs2338115	*PIP4K2B*	rs2876140	*PLCB1*
rs6108160	*PLCB1*	rs626866	*PIP4K2B*	rs6039237	*PLCB1*
rs6086525	*PLCB1*	rs7207178	*PIP4K2B*	rs768989	*PLCB1*
rs6118262	*PLCB1*	rs764190	*PIP4K2B*	rs6056024	*PLCB1*
rs6039206	*PLCB1*	rs11172254	*PIP4K2C*	rs2295179	*PLCB1*
rs4813865	*PLCB1*	rs775250	*PIP4K2C*	rs6056028	*PLCB1*
rs2223538	*PLCB1*	rs775251	*PIP4K2C*	rs16995121	*PLCB1*
rs6055944	*PLCB1*	rs812315	*PIP4K2C*	rs6077411	*PLCB1*
rs6118268	*PLCB1*	rs4520422	*PIP5K1A*	rs6133610	*PLCB1*
rs8126112	*PLCB1*	rs4970944	*PIP5K1A*	rs8123323	*PLCB1*
rs6077396	*PLCB1*	rs7532935	*PIP5K1A*	rs2076685	*PLCB1*
rs8126070	*PLCB1*	rs10114872	*PIP5K1B*	rs17446308	*PLCB1*
rs4432538	*PLCB1*	rs10117043	*PIP5K1B*	rs6086564	*PLCB1*
rs4419296	*PLCB1*	rs10121993	*PIP5K1B*	rs8117234	*PLCB1*
rs2223837	*PLCB1*	rs10123076	*PIP5K1B*	rs6077414	*PLCB1*
rs6086543	*PLCB1*	rs10125301	*PIP5K1B*	rs1555212	*PLCB1*
rs12053642	*PLCB1*	rs1014237	*PIP5K1B*	rs1015170	*PLCB1*
rs6039211	*PLCB1*	rs1014807	*PIP5K1B*	rs17446441	*PLCB1*
rs1018443	*PLCB1*	rs10511963	*PIP5K1B*	rs6086567	*PLCB1*
rs6140671	*PLCB1*	rs10735625	*PIP5K1B*	rs6133612	*PLCB1*
rs6140677	*PLCB1*	rs10746974	*PIP5K1B*	rs6086570	*PLCB1*
rs6055990	*PLCB1*	rs10746983	*PIP5K1B*	rs2143266	*PLCB1*
rs6108174	*PLCB1*	rs10781117	*PIP5K1B*	rs2179478	*PLCB1*
rs1033566	*PLCB1*	rs10781306	*PIP5K1B*	rs6086582	*PLCB1*
rs6055995	*PLCB1*	rs10781329	*PIP5K1B*	rs6056080	*PLCB1*
rs6056006	*PLCB1*	rs10869335	*PIP5K1B*	rs6077420	*PLCB1*
rs2876140	*PLCB1*	rs10869396	*PIP5K1B*	rs6086590	*PLCB1*
rs6039237	*PLCB1*	rs10869400	*PIP5K1B*	rs1040496	*PLCB1*
rs768989	*PLCB1*	rs10869420	*PIP5K1B*	rs4496390	*PLCB1*
rs6056024	*PLCB1*	rs10869538	*PIP5K1B*	rs4816085	*PLCB1*
rs2295179	*PLCB1*	rs10869686	*PIP5K1B*	rs6039268	*PLCB1*
rs6056028	*PLCB1*	rs1107108	*PIP5K1B*	rs2206489	*PLCB1*
rs16995121	*PLCB1*	rs11143417	*PIP5K1B*	rs6056111	*PLCB1*
rs6077411	*PLCB1*	rs11144027	*PIP5K1B*	rs2294597	*PLCB1*
rs6133610	*PLCB1*	rs11144133	*PIP5K1B*	rs6056114	*PLCB1*
rs8123323	*PLCB1*	rs12339235	*PIP5K1B*	rs6516403	*PLCB1*
rs2076685	*PLCB1*	rs12349586	*PIP5K1B*	rs1967681	*PLCB1*
rs17446308	*PLCB1*	rs12380573	*PIP5K1B*	rs11906514	*PLCB1*
rs8117234	*PLCB1*	rs12686355	*PIP5K1B*	rs1474683	*PLCB1*
rs6077414	*PLCB1*	rs12686693	*PIP5K1B*	rs2076409	*PLCB1*
rs1555212	*PLCB1*	rs13296679	*PIP5K1B*	rs4816089	*PLCB1*
rs1015170	*PLCB1*	rs1412988	*PIP5K1B*	rs6077425	*PLCB1*
rs17446441	*PLCB1*	rs1412990	*PIP5K1B*	rs4816090	*PLCB1*

rs6086567	*PLCB1*	rs1414954	*PIP5K1B*	rs724110	*PLCB1*
rs6133612	*PLCB1*	rs1541084	*PIP5K1B*	rs2876145	*PLCB1*
rs6086570	*PLCB1*	rs1556751	*PIP5K1B*	rs6086617	*PLCB1*
rs2143266	*PLCB1*	rs17058792	*PIP5K1B*	rs6039298	*PLCB1*
rs2179478	*PLCB1*	rs17058884	*PIP5K1B*	rs6056198	*PLCB1*
rs6086582	*PLCB1*	rs17059748	*PIP5K1B*	rs6077434	*PLCB1*
rs6056080	*PLCB1*	rs17391840	*PIP5K1B*	rs11698656	*PLCB1*
rs6077420	*PLCB1*	rs17392931	*PIP5K1B*	rs10485728	*PLCB1*
rs6086590	*PLCB1*	rs1889150	*PIP5K1B*	rs3902336	*PLCB1*
rs1040496	*PLCB1*	rs2151414	*PIP5K1B*	rs6056209	*PLCB1*
rs4496390	*PLCB1*	rs2151418	*PIP5K1B*	rs6039307	*PLCB1*
rs4816085	*PLCB1*	rs2152649	*PIP5K1B*	rs6140774	*PLCB1*
rs6039268	*PLCB1*	rs2871223	*PIP5K1B*	rs6133635	*PLCB1*
rs2206489	*PLCB1*	rs3812537	*PIP5K1B*	rs6108205	*PLCB1*
rs6056111	*PLCB1*	rs4237270	*PIP5K1B*	rs4083408	*PLCB1*
rs2294597	*PLCB1*	rs4745231	*PIP5K1B*	rs6039312	*PLCB1*
rs6056114	*PLCB1*	rs4745296	*PIP5K1B*	rs3848835	*PLCB1*
rs6516403	*PLCB1*	rs4745321	*PIP5K1B*	rs7269546	*PLCB1*
rs1967681	*PLCB1*	rs4745375	*PIP5K1B*	rs6056226	*PLCB1*
rs11906514	*PLCB1*	rs4745402	*PIP5K1B*	rs1047383	*PLCB1*
rs1474683	*PLCB1*	rs4745451	*PIP5K1B*	rs1047383	*PLCB1*
rs2076409	*PLCB1*	rs4745466	*PIP5K1B*	rs4924445	*PLCB2*
rs4816089	*PLCB1*	rs4745514	*PIP5K1B*	rs936213	*PLCB2*
rs6077425	*PLCB1*	rs4745520	*PIP5K1B*	rs12439272	*PLCB2*
rs926505	*PLCB1*	rs6560390	*PIP5K1B*	rs2305647	*PLCB2*
rs4816090	*PLCB1*	rs6560397	*PIP5K1B*	rs1869901	*PLCB2*
rs724110	*PLCB1*	rs6560444	*PIP5K1B*	rs3784399	*PLCB2*
rs2876145	*PLCB1*	rs7018552	*PIP5K1B*	rs10163076	*PLCB2*
rs6086617	*PLCB1*	rs7041004	*PIP5K1B*	rs934937	*PLCB2*
rs6039298	*PLCB1*	rs7044422	*PIP5K1B*	rs961090	*PLCB2*
rs6056198	*PLCB1*	rs7048825	*PIP5K1B*	rs961090	*PLCB2*
rs6077434	*PLCB1*	rs7859638	*PIP5K1B*	rs3741403	*PLCB3*
rs11698656	*PLCB1*	rs7866673	*PIP5K1B*	rs594942	*PLCB3*
rs6086627	*PLCB1*	rs8114	*PIP5K1B*	rs12798333	*PLCB3*
rs10485728	*PLCB1*	rs872077	*PIP5K1B*	rs2244625	*PLCB3*
rs3902336	*PLCB1*	rs883751	*PIP5K1B*	rs2244621	*PLCB3*
rs6056209	*PLCB1*	rs883952	*PIP5K1B*	rs915987	*PLCB3*
rs6039307	*PLCB1*	rs9314841	*PIP5K1B*	rs660442	*PLCB3*
rs6140774	*PLCB1*	rs11672559	*PIP5K1C*	rs6056386	*PLCB4*
rs6133635	*PLCB1*	rs1476592	*PIP5K1C*	rs6086762	*PLCB4*
rs6108205	*PLCB1*	rs2270083	*PIP5K1C*	rs6118479	*PLCB4*
rs4083408	*PLCB1*	rs4807493	*PIP5K1C*	rs6039386	*PLCB4*
rs6039312	*PLCB1*	rs516423	*PIP5K1C*	rs6108255	*PLCB4*
rs3848835	*PLCB1*	rs740873	*PIP5K1C*	rs8115948	*PLCB4*

rs7269546	*PLCB1*	rs757454	*PIP5K1C*	rs6140861	*PLCB4*
rs6056226	*PLCB1*	rs8109485	*PIP5K1C*	rs964310	*PLCB4*
rs1047383	*PLCB1*	rs3739821	*PIP5KL1*	rs6039393	*PLCB4*
rs6056230	*PLCB1*	rs6478803	*PIP5KL1*	rs13041524	*PLCB4*
rs6056230	*PLCB1*	rs6781	*PIP5KL1*	rs6108263	*PLCB4*
rs4924445	*PLCB2*	rs7859	*PIP5KL1*	rs16995573	*PLCB4*
rs936213	*PLCB2*	rs1005987	*PLCB1*	rs13044386	*PLCB4*
rs12439272	*PLCB2*	rs1015170	*PLCB1*	rs6118505	*PLCB4*
rs1869901	*PLCB2*	rs1018443	*PLCB1*	rs2224357	*PLCB4*
rs3784399	*PLCB2*	rs1033566	*PLCB1*	rs6056427	*PLCB4*
rs10163076	*PLCB2*	rs1033684	*PLCB1*	rs6118508	*PLCB4*
rs961090	*PLCB2*	rs1040496	*PLCB1*	rs6056440	*PLCB4*
rs3741403	*PLCB3*	rs1047383	*PLCB1*	rs6086799	*PLCB4*
rs594942	*PLCB3*	rs10485722	*PLCB1*	rs6056448	*PLCB4*
rs12798333	*PLCB3*	rs10485723	*PLCB1*	rs2208297	*PLCB4*
rs2244625	*PLCB3*	rs10485724	*PLCB1*	rs1407101	*PLCB4*
rs2244621	*PLCB3*	rs10485728	*PLCB1*	rs1321581	*PLCB4*
rs915987	*PLCB3*	rs11087808	*PLCB1*	rs2876163	*PLCB4*
rs660442	*PLCB3*	rs11698656	*PLCB1*	rs6039410	*PLCB4*
rs660442	*PLCB3*	rs11906095	*PLCB1*	rs8183334	*PLCB4*
rs6056386	*PLCB4*	rs11906514	*PLCB1*	rs5011374	*PLCB4*
rs6086762	*PLCB4*	rs12053642	*PLCB1*	rs6056505	*PLCB4*
rs6118479	*PLCB4*	rs1232779	*PLCB1*	rs2206138	*PLCB4*
rs6039386	*PLCB4*	rs1232782	*PLCB1*	rs10485730	*PLCB4*
rs6108255	*PLCB4*	rs1232783	*PLCB1*	rs6118558	*PLCB4*
rs8115948	*PLCB4*	rs1237829	*PLCB1*	rs6516454	*PLCB4*
rs964310	*PLCB4*	rs1238232	*PLCB1*	rs16995731	*PLCB4*
rs6039393	*PLCB4*	rs12480099	*PLCB1*	rs6056519	*PLCB4*
rs13041524	*PLCB4*	rs12624339	*PLCB1*	rs2327162	*PLCB4*
rs6108263	*PLCB4*	rs13037679	*PLCB1*	rs6077510	*PLCB4*
rs16995573	*PLCB4*	rs13040221	*PLCB1*	rs6077511	*PLCB4*
rs13044386	*PLCB4*	rs1342585	*PLCB1*	rs6086834	*PLCB4*
rs6118505	*PLCB4*	rs1474581	*PLCB1*	rs6056526	*PLCB4*
rs2224357	*PLCB4*	rs1474683	*PLCB1*	rs2327164	*PLCB4*
rs6056427	*PLCB4*	rs1474937	*PLCB1*	rs7272444	*PLCB4*
rs6118508	*PLCB4*	rs1534897	*PLCB1*	rs6056552	*PLCB4*
rs6056440	*PLCB4*	rs1534968	*PLCB1*	rs8115510	*PLCB4*
rs6086799	*PLCB4*	rs1555212	*PLCB1*	rs6039442	*PLCB4*
rs6056448	*PLCB4*	rs1569604	*PLCB1*	rs7268671	*PLCB4*
rs2208297	*PLCB4*	rs1605791	*PLCB1*	rs6077516	*PLCB4*
rs1407101	*PLCB4*	rs16995121	*PLCB1*	rs6039443	*PLCB4*
rs1321581	*PLCB4*	rs16995277	*PLCB1*	rs6056570	*PLCB4*
rs2876163	*PLCB4*	rs17347805	*PLCB1*	rs17481185	*PLCB4*
rs6039410	*PLCB4*	rs17362299	*PLCB1*	rs725941	*PLCB4*

rs8183334	*PLCB4*	rs17431073	*PLCB1*	rs2299676	*PLCB4*
rs5011374	*PLCB4*	rs17436253	*PLCB1*	rs6086865	*PLCB4*
rs6056505	*PLCB4*	rs17446308	*PLCB1*	rs1028338	*PLCB4*
rs2206138	*PLCB4*	rs17446441	*PLCB1*	rs6133703	*PLCB4*
rs6118558	*PLCB4*	rs1883503	*PLCB1*	rs7265537	*PLCB4*
rs6516454	*PLCB4*	rs1935671	*PLCB1*	rs2179321	*PLCB4*
rs16995731	*PLCB4*	rs1967681	*PLCB1*	rs6118591	*PLCB4*
rs6056519	*PLCB4*	rs2050090	*PLCB1*	rs1997696	*PLCB4*
rs2327162	*PLCB4*	rs2064272	*PLCB1*	rs6133707	*PLCB4*
rs6077510	*PLCB4*	rs2076409	*PLCB1*	rs6056595	*PLCB4*
rs6077511	*PLCB4*	rs2076685	*PLCB1*	rs3787309	*PLCB4*
rs6086834	*PLCB4*	rs2103653	*PLCB1*	rs3819579	*PLCB4*
rs6056526	*PLCB4*	rs2143205	*PLCB1*	rs2299679	*PLCB4*
rs2327164	*PLCB4*	rs2143266	*PLCB1*	rs6118611	*PLCB4*
rs7272444	*PLCB4*	rs2179138	*PLCB1*	rs4369940	*PLCB4*
rs6056552	*PLCB4*	rs2179440	*PLCB1*	rs6118616	*PLCB4*
rs8115510	*PLCB4*	rs2179478	*PLCB1*	rs6118618	*PLCB4*
rs6039442	*PLCB4*	rs2179984	*PLCB1*	rs6056628	*PLCB4*
rs7268671	*PLCB4*	rs2206422	*PLCB1*	rs976649	*PLCB4*
rs6077516	*PLCB4*	rs2206423	*PLCB1*	rs2072954	*PLCB4*
rs6039443	*PLCB4*	rs2206489	*PLCB1*	rs2276483	*PLCB4*
rs6056570	*PLCB4*	rs2221695	*PLCB1*	rs2276484	*PLCB4*
rs17481185	*PLCB4*	rs2223538	*PLCB1*	rs2076393	*PLCB4*
rs725941	*PLCB4*	rs2223837	*PLCB1*	rs6086897	*PLCB4*
rs2299676	*PLCB4*	rs227130	*PLCB1*	rs6086900	*PLCB4*
rs6086865	*PLCB4*	rs227133	*PLCB1*	rs6086904	*PLCB4*
rs1028338	*PLCB4*	rs227134	*PLCB1*	rs6056645	*PLCB4*
rs6133703	*PLCB4*	rs227142	*PLCB1*	rs6056645	*PLCB4*
rs7265537	*PLCB4*	rs2294259	*PLCB1*	rs928807	*PLCD1*
rs2179321	*PLCB4*	rs2294597	*PLCB1*	rs11922130	*PLCD1*
rs6118591	*PLCB4*	rs2295179	*PLCB1*	rs6599099	*PLCD1*
rs1997696	*PLCB4*	rs2327025	*PLCB1*	rs2226462	*PLCD1*
rs6133707	*PLCB4*	rs2327046	*PLCB1*	rs2212042	*PLCD1*
rs6056595	*PLCB4*	rs2327070	*PLCB1*	rs4389435	*PLCD1*
rs3787309	*PLCB4*	rs2423354	*PLCB1*	rs7630340	*PLCD1*
rs3819579	*PLCB4*	rs2423356	*PLCB1*	rs1053733	*PLCD3*
rs2299679	*PLCB4*	rs2423360	*PLCB1*	rs2269746	*PLCD3*
rs6118611	*PLCB4*	rs2423361	*PLCB1*	rs2239925	*PLCD3*
rs4369940	*PLCB4*	rs2423363	*PLCB1*	rs1052169	*PLCD3*
rs6118616	*PLCB4*	rs2423364	*PLCB1*	rs2285426	*PLCD3*
rs6118618	*PLCB4*	rs2423366	*PLCB1*	rs2285427	*PLCD3*
rs6056628	*PLCB4*	rs2662980	*PLCB1*	rs713101	*PLCD3*
rs976649	*PLCB4*	rs2662985	*PLCB1*	rs3744760	*PLCD3*
rs2072954	*PLCB4*	rs2662999	*PLCB1*	rs4362432	*PLCD3*

rs2276483	*PLCB4*	rs2719774	*PLCB1*	rs7207047	*PLCD3*
rs2276484	*PLCB4*	rs2719776	*PLCB1*	rs12944434	*PLCD3*
rs2076393	*PLCB4*	rs2719795	*PLCB1*	rs7223320	*PLCD3*
rs6086897	*PLCB4*	rs2719804	*PLCB1*	rs7224944	*PLCD3*
rs6086900	*PLCB4*	rs2719807	*PLCB1*	rs8069937	*PLCD3*
rs6086904	*PLCB4*	rs2745755	*PLCB1*	rs4986172	*PLCD3*
rs6056645	*PLCB4*	rs2745756	*PLCB1*	rs1053578	*PLCD3*
rs6056645	*PLCB4*	rs2745772	*PLCB1*	rs8070447	*PLCD3*
rs928807	*PLCD1*	rs2745776	*PLCB1*	rs8070447	*PLCD3*
rs9861030	*PLCD1*	rs2745787	*PLCB1*	rs12989189	*PLCD4*
rs11922130	*PLCD1*	rs2876140	*PLCB1*	rs3845836	*PLCD4*
rs6599099	*PLCD1*	rs2876145	*PLCB1*	rs3770214	*PLCD4*
rs2226462	*PLCD1*	rs3817881	*PLCB1*	rs7094594	*PLCE1*
rs2212042	*PLCD1*	rs3848835	*PLCB1*	rs1223577	*PLCE1*
rs4389435	*PLCD1*	rs3891453	*PLCB1*	rs11187749	*PLCE1*
rs7630340	*PLCD1*	rs3902336	*PLCB1*	rs7085672	*PLCE1*
rs7630340	*PLCD1*	rs4083408	*PLCB1*	rs11593126	*PLCE1*
rs1053733	*PLCD3*	rs4142365	*PLCB1*	rs829232	*PLCE1*
rs2269746	*PLCD3*	rs4399790	*PLCB1*	rs10882378	*PLCE1*
rs2239925	*PLCD3*	rs4419296	*PLCB1*	rs1223583	*PLCE1*
rs1052169	*PLCD3*	rs4432538	*PLCB1*	rs1223585	*PLCE1*
rs2285426	*PLCD3*	rs4471960	*PLCB1*	rs10882380	*PLCE1*
rs2285427	*PLCD3*	rs4496390	*PLCB1*	rs1776842	*PLCE1*
rs713101	*PLCD3*	rs4813853	*PLCB1*	rs10882381	*PLCE1*
rs3744760	*PLCD3*	rs4813854	*PLCB1*	rs1935960	*PLCE1*
rs3744761	*PLCD3*	rs4813863	*PLCB1*	rs1935961	*PLCE1*
rs4362432	*PLCD3*	rs4813865	*PLCB1*	rs7921117	*PLCE1*
rs7207047	*PLCD3*	rs4816047	*PLCB1*	rs2182093	*PLCE1*
rs12944434	*PLCD3*	rs4816085	*PLCB1*	rs1925243	*PLCE1*
rs7223320	*PLCD3*	rs4816089	*PLCB1*	rs2209442	*PLCE1*
rs7224944	*PLCD3*	rs4816090	*PLCB1*	rs4918070	*PLCE1*
rs8069937	*PLCD3*	rs6039040	*PLCB1*	rs6583926	*PLCE1*
rs4986172	*PLCD3*	rs6039049	*PLCB1*	rs4918082	*PLCE1*
rs8070447	*PLCD3*	rs6039104	*PLCB1*	rs11187789	*PLCE1*
rs12989189	*PLCD4*	rs6039107	*PLCB1*	rs17416616	*PLCE1*
rs3845836	*PLCD4*	rs6039109	*PLCB1*	rs1998709	*PLCE1*
rs3770214	*PLCD4*	rs6039189	*PLCB1*	rs10786152	*PLCE1*
rs3770214	*PLCD4*	rs6039190	*PLCB1*	rs1776946	*PLCE1*
rs1223577	*PLCE1*	rs6039191	*PLCB1*	rs2689694	*PLCE1*
rs11187749	*PLCE1*	rs6039206	*PLCB1*	rs2689700	*PLCE1*
rs7085672	*PLCE1*	rs6039211	*PLCB1*	rs11187808	*PLCE1*
rs11593126	*PLCE1*	rs6039237	*PLCB1*	rs2689698	*PLCE1*
rs829232	*PLCE1*	rs6039268	*PLCB1*	rs10786155	*PLCE1*
rs10882378	*PLCE1*	rs6039298	*PLCB1*	rs2689693	*PLCE1*

rs1223583	*PLCE1*	rs6039307	*PLCB1*	rs2860746	*PLCE1*
rs1223585	*PLCE1*	rs6039312	*PLCB1*	rs7908334	*PLCE1*
rs10882380	*PLCE1*	rs6055550	*PLCB1*	rs2798001	*PLCE1*
rs1776842	*PLCE1*	rs6055562	*PLCB1*	rs1858608	*PLCE1*
rs10882381	*PLCE1*	rs6055578	*PLCB1*	rs11187815	*PLCE1*
rs1935960	*PLCE1*	rs6055594	*PLCB1*	rs12769135	*PLCE1*
rs1935961	*PLCE1*	rs6055601	*PLCB1*	rs10882406	*PLCE1*
rs7921117	*PLCE1*	rs6055603	*PLCB1*	rs11187825	*PLCE1*
rs2182093	*PLCE1*	rs6055625	*PLCB1*	rs2226170	*PLCE1*
rs1925243	*PLCE1*	rs6055652	*PLCB1*	rs11187828	*PLCE1*
rs2209442	*PLCE1*	rs6055685	*PLCB1*	rs10882412	*PLCE1*
rs4918070	*PLCE1*	rs6055692	*PLCB1*	rs17109869	*PLCE1*
rs6583926	*PLCE1*	rs6055748	*PLCB1*	rs7919066	*PLCE1*
rs4918082	*PLCE1*	rs6055750	*PLCB1*	rs1547643	*PLCE1*
rs11187789	*PLCE1*	rs6055853	*PLCB1*	rs1408820	*PLCE1*
rs17416616	*PLCE1*	rs6055858	*PLCB1*	rs3740360	*PLCE1*
rs1998709	*PLCE1*	rs6055889	*PLCB1*	rs4918188	*PLCE1*
rs10786152	*PLCE1*	rs6055910	*PLCB1*	rs12263737	*PLCE1*
rs1776946	*PLCE1*	rs6055912	*PLCB1*	rs753724	*PLCE1*
rs2689694	*PLCE1*	rs6055922	*PLCB1*	rs11187842	*PLCE1*
rs2689700	*PLCE1*	rs6055923	*PLCB1*	rs3765524	*PLCE1*
rs11187808	*PLCE1*	rs6055926	*PLCB1*	rs12766693	*PLCE1*
rs2689698	*PLCE1*	rs6055927	*PLCB1*	rs4394764	*PLCE1*
rs4545470	*PLCE1*	rs6055928	*PLCB1*	rs2274223	*PLCE1*
rs2797998	*PLCE1*	rs6055944	*PLCB1*	rs17516904	*PLCE1*
rs10786155	*PLCE1*	rs6055951	*PLCB1*	rs3781264	*PLCE1*
rs2689693	*PLCE1*	rs6055990	*PLCB1*	rs2077218	*PLCE1*
rs2860746	*PLCE1*	rs6055995	*PLCB1*	rs17109928	*PLCE1*
rs7908334	*PLCE1*	rs6056006	*PLCB1*	rs6129760	*PLCG1*
rs2798001	*PLCE1*	rs6056024	*PLCB1*	rs12624863	*PLCG1*
rs1858608	*PLCE1*	rs6056028	*PLCB1*	rs753381	*PLCG1*
rs11187815	*PLCE1*	rs6056080	*PLCB1*	rs2235360	*PLCG1*
rs12769135	*PLCE1*	rs6056111	*PLCB1*	rs2235366	*PLCG1*
rs10882406	*PLCE1*	rs6056114	*PLCB1*	rs2664537	*PLCG1*
rs11187825	*PLCE1*	rs6056138	*PLCB1*	rs2664537	*PLCG1*
rs2226170	*PLCE1*	rs6056198	*PLCB1*	rs8063120	*PLCG2*
rs11187828	*PLCE1*	rs6056209	*PLCB1*	rs9937704	*PLCG2*
rs10882412	*PLCE1*	rs6056226	*PLCB1*	rs10445097	*PLCG2*
rs12248509	*PLCE1*	rs6077326	*PLCB1*	rs4328435	*PLCG2*
rs17109869	*PLCE1*	rs6077332	*PLCB1*	rs6564915	*PLCG2*
rs4917450	*PLCE1*	rs6077350	*PLCB1*	rs4254322	*PLCG2*
rs7919066	*PLCE1*	rs6077396	*PLCB1*	rs4398100	*PLCG2*
rs1547643	*PLCE1*	rs6077411	*PLCB1*	rs4580153	*PLCG2*
rs1408820	*PLCE1*	rs6077414	*PLCB1*	rs4580154	*PLCG2*

rs3740360	*PLCE1*	rs6077420	*PLCB1*	rs4243211	*PLCG2*
rs4918188	*PLCE1*	rs6077425	*PLCB1*	rs12598194	*PLCG2*
rs12263737	*PLCE1*	rs6077434	*PLCB1*	rs12448334	*PLCG2*
rs753724	*PLCE1*	rs6086343	*PLCB1*	rs4889384	*PLCG2*
rs11187842	*PLCE1*	rs6086346	*PLCB1*	rs12599264	*PLCG2*
rs3765524	*PLCE1*	rs6086348	*PLCB1*	rs4889393	*PLCG2*
rs12766693	*PLCE1*	rs6086374	*PLCB1*	rs9937223	*PLCG2*
rs4394764	*PLCE1*	rs6086402	*PLCB1*	rs4456499	*PLCG2*
rs2274223	*PLCE1*	rs6086458	*PLCB1*	rs6420427	*PLCG2*
rs17516904	*PLCE1*	rs6086459	*PLCB1*	rs4405545	*PLCG2*
rs3781264	*PLCE1*	rs6086477	*PLCB1*	rs7499275	*PLCG2*
rs2077218	*PLCE1*	rs6086490	*PLCB1*	rs7194131	*PLCG2*
rs17109928	*PLCE1*	rs6086493	*PLCB1*	rs4325546	*PLCG2*
rs17109928	*PLCE1*	rs6086495	*PLCB1*	rs8043593	*PLCG2*
rs6129760	*PLCG1*	rs6086511	*PLCB1*	rs4889411	*PLCG2*
rs12624863	*PLCG1*	rs6086518	*PLCB1*	rs4888179	*PLCG2*
rs2228246	*PLCG1*	rs6086525	*PLCB1*	rs7202205	*PLCG2*
rs753381	*PLCG1*	rs6086543	*PLCB1*	rs4072683	*PLCG2*
rs2235360	*PLCG1*	rs6086564	*PLCB1*	rs7185362	*PLCG2*
rs2235366	*PLCG1*	rs6086567	*PLCB1*	rs4074445	*PLCG2*
rs2664537	*PLCG1*	rs6086570	*PLCB1*	rs4888181	*PLCG2*
rs2664537	*PLCG1*	rs6086582	*PLCB1*	rs12596639	*PLCG2*
rs8063120	*PLCG2*	rs6086590	*PLCB1*	rs11643875	*PLCG2*
rs9937704	*PLCG2*	rs6086617	*PLCB1*	rs4243218	*PLCG2*
rs10445097	*PLCG2*	rs6086627	*PLCB1*	rs3935877	*PLCG2*
rs4328435	*PLCG2*	rs6108142	*PLCB1*	rs4889422	*PLCG2*
rs6564915	*PLCG2*	rs6108152	*PLCB1*	rs7342694	*PLCG2*
rs4254322	*PLCG2*	rs6108159	*PLCB1*	rs11644646	*PLCG2*
rs4398100	*PLCG2*	rs6108160	*PLCB1*	rs4997772	*PLCG2*
rs4580153	*PLCG2*	rs6108174	*PLCB1*	rs4888184	*PLCG2*
rs4580154	*PLCG2*	rs6108205	*PLCB1*	rs9932716	*PLCG2*
rs4243211	*PLCG2*	rs6118073	*PLCB1*	rs3935743	*PLCG2*
rs12598194	*PLCG2*	rs6118075	*PLCB1*	rs7197601	*PLCG2*
rs12448334	*PLCG2*	rs6118083	*PLCB1*	rs11644436	*PLCG2*
rs4889384	*PLCG2*	rs6118104	*PLCB1*	rs8062633	*PLCG2*
rs12599264	*PLCG2*	rs6118219	*PLCB1*	rs12446070	*PLCG2*
rs4889393	*PLCG2*	rs6118234	*PLCB1*	rs8063355	*PLCG2*
rs9937223	*PLCG2*	rs6118252	*PLCB1*	rs4133125	*PLCG2*
rs4456499	*PLCG2*	rs6118257	*PLCB1*	rs4133124	*PLCG2*
rs6420427	*PLCG2*	rs6118262	*PLCB1*	rs8055576	*PLCG2*
rs4405545	*PLCG2*	rs6118268	*PLCB1*	rs4889425	*PLCG2*
rs7499275	*PLCG2*	rs6133556	*PLCB1*	rs4889426	*PLCG2*
rs7194131	*PLCG2*	rs6133564	*PLCB1*	rs4889428	*PLCG2*
rs4325546	*PLCG2*	rs6133566	*PLCB1*	rs4889432	*PLCG2*

rs8043593	*PLCG2*	rs6133567	*PLCB1*	rs4889436	*PLCG2*
rs4889411	*PLCG2*	rs6133573	*PLCB1*	rs11859107	*PLCG2*
rs4888179	*PLCG2*	rs6133610	*PLCB1*	rs4369658	*PLCG2*
rs7202205	*PLCG2*	rs6133612	*PLCB1*	rs4306504	*PLCG2*
rs4072683	*PLCG2*	rs6133635	*PLCB1*	rs11864701	*PLCG2*
rs7185362	*PLCG2*	rs6140549	*PLCB1*	rs12448055	*PLCG2*
rs4074445	*PLCG2*	rs6140561	*PLCB1*	rs3922849	*PLCG2*
rs4888181	*PLCG2*	rs6140562	*PLCB1*	rs13331678	*PLCG2*
rs12596639	*PLCG2*	rs6140589	*PLCB1*	rs7203619	*PLCG2*
rs11643875	*PLCG2*	rs6140595	*PLCB1*	rs9938623	*PLCG2*
rs4243218	*PLCG2*	rs6140611	*PLCB1*	rs7201045	*PLCG2*
rs3935877	*PLCG2*	rs6140613	*PLCB1*	rs8063604	*PLCG2*
rs4889422	*PLCG2*	rs6140619	*PLCB1*	rs12446596	*PLCG2*
rs7342694	*PLCG2*	rs6140629	*PLCB1*	rs6564940	*PLCG2*
rs11644646	*PLCG2*	rs6140671	*PLCB1*	rs7187863	*PLCG2*
rs4997772	*PLCG2*	rs6140774	*PLCB1*	rs7499440	*PLCG2*
rs4888184	*PLCG2*	rs6516403	*PLCB1*	rs7500286	*PLCG2*
rs9932716	*PLCG2*	rs708912	*PLCB1*	rs12598402	*PLCG2*
rs3935743	*PLCG2*	rs708916	*PLCB1*	rs8043619	*PLCG2*
rs7197601	*PLCG2*	rs708920	*PLCB1*	rs4073828	*PLCG2*
rs11644436	*PLCG2*	rs708925	*PLCB1*	rs3936112	*PLCG2*
rs8062633	*PLCG2*	rs708931	*PLCB1*	rs4369659	*PLCG2*
rs12446070	*PLCG2*	rs722665	*PLCB1*	rs4888191	*PLCG2*
rs8063355	*PLCG2*	rs724110	*PLCB1*	rs12918369	*PLCG2*
rs4133125	*PLCG2*	rs7269546	*PLCB1*	rs4405546	*PLCG2*
rs4133124	*PLCG2*	rs7271063	*PLCB1*	rs4889444	*PLCG2*
rs8055576	*PLCG2*	rs727684	*PLCB1*	rs10514519	*PLCG2*
rs4889425	*PLCG2*	rs728213	*PLCB1*	rs11862662	*PLCG2*
rs4889426	*PLCG2*	rs7348709	*PLCB1*	rs16956040	*PLCG2*
rs4889428	*PLCG2*	rs742615	*PLCB1*	rs4611452	*PLCG2*
rs4889432	*PLCG2*	rs742616	*PLCB1*	rs17203310	*PLCG2*
rs4889436	*PLCG2*	rs764439	*PLCB1*	rs4508413	*PLCG2*
rs4243221	*PLCG2*	rs764440	*PLCB1*	rs12716928	*PLCG2*
rs11859107	*PLCG2*	rs768989	*PLCB1*	rs12921780	*PLCG2*
rs4369658	*PLCG2*	rs771941	*PLCB1*	rs8056564	*PLCG2*
rs4306504	*PLCG2*	rs771944	*PLCB1*	rs8055043	*PLCG2*
rs11864701	*PLCG2*	rs771945	*PLCB1*	rs4284633	*PLCG2*
rs12448055	*PLCG2*	rs8114499	*PLCB1*	rs4312298	*PLCG2*
rs3922849	*PLCG2*	rs8115925	*PLCB1*	rs4603554	*PLCG2*
rs13331678	*PLCG2*	rs8117234	*PLCB1*	rs8063813	*PLCG2*
rs7203619	*PLCG2*	rs8118127	*PLCB1*	rs8047356	*PLCG2*
rs9938623	*PLCG2*	rs8123323	*PLCB1*	rs4888197	*PLCG2*
rs7201045	*PLCG2*	rs8125486	*PLCB1*	rs4889448	*PLCG2*
rs8063604	*PLCG2*	rs8126112	*PLCB1*	rs9928191	*PLCG2*

rs12446596	*PLCG2*	rs978266	*PLCB1*	rs17793122	*PLCG2*
rs6564940	*PLCG2*	rs995040	*PLCB1*	rs4286103	*PLCG2*
rs7187863	*PLCG2*	rs10163076	*PLCB2*	rs4243226	*PLCG2*
rs7499440	*PLCG2*	rs12439272	*PLCB2*	rs9938835	*PLCG2*
rs7500286	*PLCG2*	rs1869901	*PLCB2*	rs4888201	*PLCG2*
rs12598402	*PLCG2*	rs2290550	*PLCB2*	rs4888201	*PLCG2*
rs8043619	*PLCG2*	rs2305647	*PLCB2*	rs1125539	*PLCH1*
rs4073828	*PLCG2*	rs3784399	*PLCB2*	rs934591	*PLCH1*
rs3936112	*PLCG2*	rs4924445	*PLCB2*	rs6790399	*PLCH1*
rs4369659	*PLCG2*	rs934937	*PLCB2*	rs7637342	*PLCH1*
rs4888191	*PLCG2*	rs936213	*PLCB2*	rs10513478	*PLCH1*
rs12918369	*PLCG2*	rs961090	*PLCB2*	rs6440997	*PLCH1*
rs4405546	*PLCG2*	rs12798333	*PLCB3*	rs592982	*PLCH1*
rs4889444	*PLCG2*	rs2244621	*PLCB3*	rs3851357	*PLCH1*
rs10514519	*PLCG2*	rs2244625	*PLCB3*	rs517971	*PLCH1*
rs11862662	*PLCG2*	rs3741403	*PLCB3*	rs7630469	*PLCH1*
rs16956040	*PLCG2*	rs594942	*PLCB3*	rs1850847	*PLCH1*
rs4611452	*PLCG2*	rs655896	*PLCB3*	rs9289957	*PLCH1*
rs4508413	*PLCG2*	rs660442	*PLCB3*	rs13085233	*PLCH1*
rs12716928	*PLCG2*	rs915987	*PLCB3*	rs359565	*PLCH1*
rs12921780	*PLCG2*	rs1028338	*PLCB4*	rs359570	*PLCH1*
rs8056564	*PLCG2*	rs10485730	*PLCB4*	rs359571	*PLCH1*
rs8055043	*PLCG2*	rs11904996	*PLCB4*	rs7629025	*PLCH1*
rs4284633	*PLCG2*	rs13041524	*PLCB4*	rs10910078	*PLCH2*
rs4312298	*PLCG2*	rs13044386	*PLCB4*	rs1998760	*PLCH2*
rs4603554	*PLCG2*	rs1321581	*PLCB4*	rs2494626	*PLCH2*
rs8063813	*PLCG2*	rs1407101	*PLCB4*	rs13376356	*PLCH2*
rs8047356	*PLCG2*	rs16995573	*PLCB4*	rs11588930	*PLCH2*
rs4888197	*PLCG2*	rs16995731	*PLCB4*	rs12049628	*PLCH2*
rs4889448	*PLCG2*	rs16995800	*PLCB4*	rs17373634	*PLCH2*
rs9928191	*PLCG2*	rs17481185	*PLCB4*	rs2477703	*PLCH2*
rs17793122	*PLCG2*	rs1997696	*PLCB4*	rs3762444	*PLCH2*
rs4286103	*PLCG2*	rs2072954	*PLCB4*	rs7535528	*PLCH2*
rs4243226	*PLCG2*	rs2076393	*PLCB4*	rs2236395	*PLCH2*
rs9938764	*PLCG2*	rs2179321	*PLCB4*	rs513426	*PLCZ1*
rs9938835	*PLCG2*	rs2206138	*PLCB4*	rs665197	*PLCZ1*
rs4888201	*PLCG2*	rs2208297	*PLCB4*	rs1550990	*PLCZ1*
rs1125539	*PLCH1*	rs2224357	*PLCB4*	rs1386398	*PLCZ1*
rs934591	*PLCH1*	rs2276483	*PLCB4*	rs2306798	*PLCZ1*
rs6790399	*PLCH1*	rs2276484	*PLCB4*	rs11833512	*PLCZ1*
rs7637342	*PLCH1*	rs2299676	*PLCB4*	rs4764417	*PLCZ1*
rs10513478	*PLCH1*	rs2299679	*PLCB4*	rs1471891	*PLCZ1*
rs3851357	*PLCH1*	rs2327162	*PLCB4*	rs901528	*PLCZ1*
rs517971	*PLCH1*	rs2327164	*PLCB4*	rs10841075	*PLCZ1*

rs7630469	*PLCH1*	rs2876163	*PLCB4*	rs10841077	*PLCZ1*
rs1850847	*PLCH1*	rs3787309	*PLCB4*	rs7974908	*PLCZ1*
rs9289957	*PLCH1*	rs3819579	*PLCB4*	rs1021266	*PLCZ1*
rs13085233	*PLCH1*	rs4141984	*PLCB4*	rs1021267	*PLCZ1*
rs359565	*PLCH1*	rs4369940	*PLCB4*	rs6486914	*PLCZ1*
rs359570	*PLCH1*	rs5011374	*PLCB4*	rs11044268	*PLCZ1*
rs7629025	*PLCH1*	rs6039386	*PLCB4*	rs10505831	*PLCZ1*
rs7629025	*PLCH1*	rs6039393	*PLCB4*	rs1027032	*PLCZ1*
rs10910078	*PLCH2*	rs6039410	*PLCB4*	rs7972408	*PLCZ1*
rs2494626	*PLCH2*	rs6039442	*PLCB4*	rs7485517	*PLCZ1*
rs13376356	*PLCH2*	rs6039443	*PLCB4*	rs17488409	*PLCZ1*
rs11588930	*PLCH2*	rs6056386	*PLCB4*	rs969489	*PLCZ1*
rs12049628	*PLCH2*	rs6056427	*PLCB4*	rs10841085	*PLCZ1*
rs17373634	*PLCH2*	rs6056440	*PLCB4*	rs10841085	*PLCZ1*
rs2477703	*PLCH2*	rs6056448	*PLCB4*	rs1595947	*PLD4*
rs3762444	*PLCH2*	rs6056505	*PLCB4*	rs3001421	*PLD4*
rs7535528	*PLCH2*	rs6056519	*PLCB4*	rs2841277	*PLD4*
rs2236395	*PLCH2*	rs6056526	*PLCB4*	rs1048257	*PLD4*
rs2236395	*PLCH2*	rs6056552	*PLCB4*	rs2819419	*PLD4*
rs513426	*PLCZ1*	rs6056570	*PLCB4*	rs694985	*PPIP5K1*
rs665197	*PLCZ1*	rs6056595	*PLCB4*	rs12912505	*PPIP5K1*
rs1550990	*PLCZ1*	rs6056628	*PLCB4*	rs2245715	*PPIP5K1*
rs1386398	*PLCZ1*	rs6056645	*PLCB4*	rs689931	*PPIP5K1*
rs2306798	*PLCZ1*	rs6077510	*PLCB4*	rs689797	*PPIP5K1*
rs11833512	*PLCZ1*	rs6077511	*PLCB4*	rs2255663	*PPIP5K1*
rs4764417	*PLCZ1*	rs6077516	*PLCB4*	rs496584	*PPIP5K1*
rs1471891	*PLCZ1*	rs6086762	*PLCB4*	rs2255042	*PPIP5K1*
rs901528	*PLCZ1*	rs6086799	*PLCB4*	rs2251844	*PPIP5K1*
rs10841075	*PLCZ1*	rs6086834	*PLCB4*	rs34813	*PPIP5K2*
rs10841077	*PLCZ1*	rs6086865	*PLCB4*	rs26521	*PPIP5K2*
rs7974908	*PLCZ1*	rs6086897	*PLCB4*	rs183752	*PPIP5K2*
rs1021266	*PLCZ1*	rs6086900	*PLCB4*	rs26821	*PPIP5K2*
rs1021267	*PLCZ1*	rs6086904	*PLCB4*	rs26819	*PPIP5K2*
rs6486914	*PLCZ1*	rs6108255	*PLCB4*	rs11744885	*PPIP5K2*
rs11044268	*PLCZ1*	rs6108263	*PLCB4*	rs26258	*PPIP5K2*
rs10505831	*PLCZ1*	rs6118479	*PLCB4*	rs246916	*PPIP5K2*
rs1027032	*PLCZ1*	rs6118505	*PLCB4*	rs246912	*PPIP5K2*
rs7972408	*PLCZ1*	rs6118508	*PLCB4*	rs10887758	*PTEN*
rs7485517	*PLCZ1*	rs6118558	*PLCB4*	rs1234212	*PTEN*
rs17488409	*PLCZ1*	rs6118591	*PLCB4*	rs1234221	*PTEN*
rs969489	*PLCZ1*	rs6118611	*PLCB4*	rs1234220	*PTEN*
rs10841085	*PLCZ1*	rs6118616	*PLCB4*	rs11202596	*PTEN*
rs1595947	*PLD4*	rs6118618	*PLCB4*	rs2299939	*PTEN*
rs3001421	*PLD4*	rs6133703	*PLCB4*	rs2248293	*PTEN*

rs2841277	*PLD4*	rs6133707	*PLCB4*	rs2673832	*PTEN*
rs1048257	*PLD4*	rs6140861	*PLCB4*	rs11202607	*PTEN*
rs2819419	*PLD4*	rs6516454	*PLCB4*	rs478839	*PTEN*
rs2819419	*PLD4*	rs725941	*PLCB4*	rs10509532	*PTEN*
rs694985	*PPIP5K1*	rs7265537	*PLCB4*	rs845016	*SYNJ1*
rs12912505	*PPIP5K1*	rs7266929	*PLCB4*	rs845018	*SYNJ1*
rs2245715	*PPIP5K1*	rs7268671	*PLCB4*	rs7279487	*SYNJ1*
rs689931	*PPIP5K1*	rs7269910	*PLCB4*	rs11702774	*SYNJ1*
rs689797	*PPIP5K1*	rs7272444	*PLCB4*	rs10470165	*SYNJ1*
rs2255663	*PPIP5K1*	rs8115510	*PLCB4*	rs17694546	*SYNJ1*
rs496584	*PPIP5K1*	rs8115948	*PLCB4*	rs2833942	*SYNJ1*
rs2255042	*PPIP5K1*	rs8183334	*PLCB4*	rs844996	*SYNJ1*
rs2251844	*PPIP5K1*	rs964310	*PLCB4*	rs2254562	*SYNJ1*
rs2251844	*PPIP5K1*	rs976649	*PLCB4*	rs1783099	*SYNJ1*
rs34813	*PPIP5K2*	rs11704319	*PLCD1*	rs582547	*SYNJ1*
rs26521	*PPIP5K2*	rs12484030	*PLCD1*	rs648648	*SYNJ1*
rs183752	*PPIP5K2*	rs12627970	*PLCD1*	rs632324	*SYNJ1*
rs26821	*PPIP5K2*	rs137602	*PLCD1*	rs12626242	*SYNJ1*
rs26819	*PPIP5K2*	rs137618	*PLCD1*	rs12626242	*SYNJ1*
rs11744885	*PPIP5K2*	rs137621	*PLCD1*	rs9654570	*SYNJ2*
rs26258	*PPIP5K2*	rs137625	*PLCD1*	rs9458975	*SYNJ2*
rs246916	*PPIP5K2*	rs137636	*PLCD1*	rs12663163	*SYNJ2*
rs246912	*PPIP5K2*	rs137653	*PLCD1*	rs2025641	*SYNJ2*
rs246912	*PPIP5K2*	rs1569498	*PLCD1*	rs9365674	*SYNJ2*
rs10887758	*PTEN*	rs2014842	*PLCD1*	rs9459056	*SYNJ2*
rs1022427	*PTEN*	rs2076125	*PLCD1*	rs10455936	*SYNJ2*
rs1234212	*PTEN*	rs54211	*PLCD1*	rs10806791	*SYNJ2*
rs1234221	*PTEN*	rs5757611	*PLCD1*	rs9356200	*SYNJ2*
rs1234220	*PTEN*	rs5995711	*PLCD1*	rs6904665	*SYNJ2*
rs11202596	*PTEN*	rs738331	*PLCD1*	rs12202135	*SYNJ2*
rs2299939	*PTEN*	rs79290	*PLCD1*	rs9459093	*SYNJ2*
rs2248293	*PTEN*	rs1052169	*PLCD3*	rs9365723	*SYNJ2*
rs11202607	*PTEN*	rs1053733	*PLCD3*	rs9365724	*SYNJ2*
rs478839	*PTEN*	rs12944434	*PLCD3*	rs6455949	*SYNJ2*
rs10509532	*PTEN*	rs2239925	*PLCD3*	rs9459154	*SYNJ2*
rs10509532	*PTEN*	rs2269746	*PLCD3*	rs999613	*SYNJ2*
rs845016	*SYNJ1*	rs2285426	*PLCD3*	rs7768038	*SYNJ2*
rs845018	*SYNJ1*	rs2285427	*PLCD3*	rs2295893	*SYNJ2*
rs7279487	*SYNJ1*	rs3744760	*PLCD3*	rs2295894	*SYNJ2*
rs11702774	*SYNJ1*	rs4362432	*PLCD3*	rs1750043	*SYNJ2*
rs10470165	*SYNJ1*	rs4986172	*PLCD3*	rs1977356	*SYNJ2*
rs17694546	*SYNJ1*	rs713101	*PLCD3*	rs9295289	*SYNJ2*
rs844996	*SYNJ1*	rs7207047	*PLCD3*	rs750997	*SYNJ2*
rs2254562	*SYNJ1*	rs7223320	*PLCD3*	rs2181190	*SYNJ2*

rs1783099	*SYNJ1*
rs582547	*SYNJ1*
rs648648	*SYNJ1*
rs632324	*SYNJ1*
rs12626242	*SYNJ1*
rs12626242	*SYNJ1*
rs9654570	*SYNJ2*
rs9458975	*SYNJ2*
rs12663163	*SYNJ2*
rs2025641	*SYNJ2*
rs9365674	*SYNJ2*
rs9459056	*SYNJ2*
rs10455936	*SYNJ2*
rs10806791	*SYNJ2*
rs9356200	*SYNJ2*
rs12202135	*SYNJ2*
rs9459093	*SYNJ2*
rs9365723	*SYNJ2*
rs9365724	*SYNJ2*
rs9459154	*SYNJ2*
rs999613	*SYNJ2*
rs7768038	*SYNJ2*
rs2295893	*SYNJ2*
rs2295894	*SYNJ2*
rs1750043	*SYNJ2*
rs1977356	*SYNJ2*
rs9295289	*SYNJ2*
rs750997	*SYNJ2*
rs2181190	*SYNJ2*
rs2502620	*SYNJ2*
rs2502618	*SYNJ2*
rs1009014	*SYNJ2*
rs17489570	*SYNJ2*
rs751873	*SYNJ2*
rs4333441	*SYNJ2*
rs6455990	*SYNJ2*
rs1744178	*SYNJ2*
rs1750040	*SYNJ2*
rs3818457	*SYNJ2*
rs350292	*SYNJ2*
rs12208248	*SYNJ2*
rs1744173	*SYNJ2*
rs1744169	*SYNJ2*
rs350289	*SYNJ2*
rs7224944	*PLCD3*
rs8069937	*PLCD3*
rs8070447	*PLCD3*
rs12989189	*PLCD4*
rs3770214	*PLCD4*
rs3845836	*PLCD4*
rs10786152	*PLCE1*
rs10786155	*PLCE1*
rs10882378	*PLCE1*
rs10882380	*PLCE1*
rs10882381	*PLCE1*
rs10882406	*PLCE1*
rs10882412	*PLCE1*
rs11187749	*PLCE1*
rs11187789	*PLCE1*
rs11187808	*PLCE1*
rs11187815	*PLCE1*
rs11187825	*PLCE1*
rs11187828	*PLCE1*
rs11187842	*PLCE1*
rs11593126	*PLCE1*
rs1223577	*PLCE1*
rs1223583	*PLCE1*
rs1223585	*PLCE1*
rs12248509	*PLCE1*
rs12249709	*PLCE1*
rs12250213	*PLCE1*
rs12263737	*PLCE1*
rs12766693	*PLCE1*
rs12769135	*PLCE1*
rs1408820	*PLCE1*
rs1547643	*PLCE1*
rs17109869	*PLCE1*
rs17109928	*PLCE1*
rs17416616	*PLCE1*
rs17516904	*PLCE1*
rs1776842	*PLCE1*
rs1776946	*PLCE1*
rs1858608	*PLCE1*
rs1925243	*PLCE1*
rs1935960	*PLCE1*
rs1935961	*PLCE1*
rs1998709	*PLCE1*
rs2077218	*PLCE1*
rs10946036	*SYNJ2*
rs2502620	*SYNJ2*
rs2502618	*SYNJ2*
rs1009014	*SYNJ2*
rs17489570	*SYNJ2*
rs751873	*SYNJ2*
rs4333441	*SYNJ2*
rs6455990	*SYNJ2*
rs1744178	*SYNJ2*
rs1750040	*SYNJ2*
rs3818457	*SYNJ2*
rs350292	*SYNJ2*
rs12208248	*SYNJ2*
rs1744173	*SYNJ2*
rs1744169	*SYNJ2*
rs350289	*SYNJ2*
rs13217929	*SYNJ2*
rs2502601	*SYNJ2*
rs2475556	*SYNJ2*
rs10744720	*TPI1*
rs2238114	*TPI1*
rs2238114	*TPI1*

rs13217929	*SYNJ2*	rs2182093	*PLCE1*
rs2502601	*SYNJ2*	rs2209442	*PLCE1*
rs2475556	*SYNJ2*	rs2226170	*PLCE1*
rs2475556	*SYNJ2*	rs2274223	*PLCE1*
rs10744720	*TPI1*	rs2689693	*PLCE1*
rs2238114	*TPI1*	rs2689694	*PLCE1*
rs2238114	*TPI1*	rs2689698	*PLCE1*
		rs2689700	*PLCE1*
		rs2764343	*PLCE1*
		rs2797998	*PLCE1*
		rs2798001	*PLCE1*
		rs2860746	*PLCE1*
		rs3740360	*PLCE1*
		rs3765524	*PLCE1*
		rs3781264	*PLCE1*
		rs4394764	*PLCE1*
		rs4918070	*PLCE1*
		rs4918082	*PLCE1*
		rs4918188	*PLCE1*
		rs6583926	*PLCE1*
		rs7085672	*PLCE1*
		rs7094594	*PLCE1*
		rs753724	*PLCE1*
		rs7908334	*PLCE1*
		rs7911571	*PLCE1*
		rs7919066	*PLCE1*
		rs7921117	*PLCE1*
		rs829232	*PLCE1*
		rs12624863	*PLCG1*
		rs2228246	*PLCG1*
		rs2235360	*PLCG1*
		rs2235366	*PLCG1*
		rs2664537	*PLCG1*
		rs6129760	*PLCG1*
		rs753381	*PLCG1*
		rs8122204	*PLCG1*
		rs10445097	*PLCG2*
		rs10514519	*PLCG2*
		rs1143689	*PLCG2*
		rs11643875	*PLCG2*
		rs11644436	*PLCG2*
		rs11644646	*PLCG2*
		rs11859107	*PLCG2*
		rs11862662	*PLCG2*

rs11864701	*PLCG2*
rs12446070	*PLCG2*
rs12446596	*PLCG2*
rs12448055	*PLCG2*
rs12448334	*PLCG2*
rs12596639	*PLCG2*
rs12598194	*PLCG2*
rs12598402	*PLCG2*
rs12599264	*PLCG2*
rs12716928	*PLCG2*
rs12918369	*PLCG2*
rs12921780	*PLCG2*
rs13331678	*PLCG2*
rs16956011	*PLCG2*
rs16956040	*PLCG2*
rs17203310	*PLCG2*
rs17793122	*PLCG2*
rs3922849	*PLCG2*
rs3934954	*PLCG2*
rs3935743	*PLCG2*
rs3935877	*PLCG2*
rs3936112	*PLCG2*
rs4072683	*PLCG2*
rs4073828	*PLCG2*
rs4074445	*PLCG2*
rs4133124	*PLCG2*
rs4133125	*PLCG2*
rs4243211	*PLCG2*
rs4243218	*PLCG2*
rs4243226	*PLCG2*
rs4254322	*PLCG2*
rs4284633	*PLCG2*
rs4286103	*PLCG2*
rs4306504	*PLCG2*
rs4312298	*PLCG2*
rs4325546	*PLCG2*
rs4328435	*PLCG2*
rs4369658	*PLCG2*
rs4369659	*PLCG2*
rs4398100	*PLCG2*
rs4405545	*PLCG2*
rs4405546	*PLCG2*
rs4456499	*PLCG2*
rs4508413	*PLCG2*

rs4580153	*PLCG2*
rs4580154	*PLCG2*
rs4603554	*PLCG2*
rs4611452	*PLCG2*
rs4888179	*PLCG2*
rs4888181	*PLCG2*
rs4888184	*PLCG2*
rs4888191	*PLCG2*
rs4888197	*PLCG2*
rs4888201	*PLCG2*
rs4889384	*PLCG2*
rs4889393	*PLCG2*
rs4889411	*PLCG2*
rs4889422	*PLCG2*
rs4889425	*PLCG2*
rs4889426	*PLCG2*
rs4889428	*PLCG2*
rs4889432	*PLCG2*
rs4889436	*PLCG2*
rs4889444	*PLCG2*
rs4889448	*PLCG2*
rs4997772	*PLCG2*
rs6420427	*PLCG2*
rs6564915	*PLCG2*
rs6564940	*PLCG2*
rs7185362	*PLCG2*
rs7187863	*PLCG2*
rs7194131	*PLCG2*
rs7197601	*PLCG2*
rs7201045	*PLCG2*
rs7202205	*PLCG2*
rs7203619	*PLCG2*
rs7342694	*PLCG2*
rs7499275	*PLCG2*
rs7499440	*PLCG2*
rs7500286	*PLCG2*
rs8043593	*PLCG2*
rs8043619	*PLCG2*
rs8047356	*PLCG2*
rs8055043	*PLCG2*
rs8055576	*PLCG2*
rs8056564	*PLCG2*
rs8062633	*PLCG2*
rs8063120	*PLCG2*

rs8063355	*PLCG2*
rs8063604	*PLCG2*
rs8063813	*PLCG2*
rs9928191	*PLCG2*
rs9932716	*PLCG2*
rs9937223	*PLCG2*
rs9937704	*PLCG2*
rs9938623	*PLCG2*
rs9938764	*PLCG2*
rs9938835	*PLCG2*
rs10513478	*PLCH1*
rs1125539	*PLCH1*
rs11915975	*PLCH1*
rs13085233	*PLCH1*
rs1850847	*PLCH1*
rs359565	*PLCH1*
rs359570	*PLCH1*
rs3851357	*PLCH1*
rs517971	*PLCH1*
rs592982	*PLCH1*
rs6440997	*PLCH1*
rs6790399	*PLCH1*
rs7629025	*PLCH1*
rs7630469	*PLCH1*
rs7637342	*PLCH1*
rs9289957	*PLCH1*
rs934591	*PLCH1*
rs10910078	*PLCH2*
rs11588930	*PLCH2*
rs12049628	*PLCH2*
rs13376356	*PLCH2*
rs17373634	*PLCH2*
rs1998760	*PLCH2*
rs2236395	*PLCH2*
rs2477703	*PLCH2*
rs2494626	*PLCH2*
rs3762444	*PLCH2*
rs7535528	*PLCH2*
rs1021266	*PLCZ1*
rs1021267	*PLCZ1*
rs1027032	*PLCZ1*
rs10505831	*PLCZ1*
rs10841075	*PLCZ1*
rs10841077	*PLCZ1*

rs10841085	*PLCZ1*
rs11044268	*PLCZ1*
rs11833512	*PLCZ1*
rs12823264	*PLCZ1*
rs1386398	*PLCZ1*
rs1471891	*PLCZ1*
rs1550990	*PLCZ1*
rs17488409	*PLCZ1*
rs2306798	*PLCZ1*
rs4764417	*PLCZ1*
rs513426	*PLCZ1*
rs6486914	*PLCZ1*
rs665197	*PLCZ1*
rs7485517	*PLCZ1*
rs7972408	*PLCZ1*
rs7974908	*PLCZ1*
rs901528	*PLCZ1*
rs969489	*PLCZ1*
rs1048257	*PLD4*
rs1595947	*PLD4*
rs2819419	*PLD4*
rs2841277	*PLD4*
rs3001421	*PLD4*
rs12912505	*PPIP5K1*
rs2245715	*PPIP5K1*
rs2251844	*PPIP5K1*
rs2255042	*PPIP5K1*
rs2255663	*PPIP5K1*
rs496584	*PPIP5K1*
rs689797	*PPIP5K1*
rs689931	*PPIP5K1*
rs694985	*PPIP5K1*
rs11744885	*PPIP5K2*
rs183752	*PPIP5K2*
rs246912	*PPIP5K2*
rs246916	*PPIP5K2*
rs26258	*PPIP5K2*
rs26521	*PPIP5K2*
rs26819	*PPIP5K2*
rs26821	*PPIP5K2*
rs34813	*PPIP5K2*
rs6867515	*PPIP5K2*
rs10509532	*PTEN*
rs10887758	*PTEN*

rs11202596	*PTEN*
rs11202607	*PTEN*
rs1234212	*PTEN*
rs1234220	*PTEN*
rs1234221	*PTEN*
rs2248293	*PTEN*
rs2299939	*PTEN*
rs2673832	*PTEN*
rs478839	*PTEN*
rs10470165	*SYNJ1*
rs11702774	*SYNJ1*
rs12626242	*SYNJ1*
rs17694546	*SYNJ1*
rs1783099	*SYNJ1*
rs2254562	*SYNJ1*
rs2833942	*SYNJ1*
rs582547	*SYNJ1*
rs632324	*SYNJ1*
rs648648	*SYNJ1*
rs7279487	*SYNJ1*
rs844996	*SYNJ1*
rs845016	*SYNJ1*
rs845018	*SYNJ1*
rs1009014	*SYNJ2*
rs10455936	*SYNJ2*
rs10806791	*SYNJ2*
rs10946036	*SYNJ2*
rs11961258	*SYNJ2*
rs12202135	*SYNJ2*
rs12208248	*SYNJ2*
rs12663163	*SYNJ2*
rs13217929	*SYNJ2*
rs1744169	*SYNJ2*
rs1744173	*SYNJ2*
rs1744178	*SYNJ2*
rs17489570	*SYNJ2*
rs1750040	*SYNJ2*
rs1750043	*SYNJ2*
rs1977356	*SYNJ2*
rs2025641	*SYNJ2*
rs2181190	*SYNJ2*
rs2295893	*SYNJ2*
rs2295894	*SYNJ2*
rs2475556	*SYNJ2*

rs2502601	*SYNJ2*
rs2502618	*SYNJ2*
rs2502620	*SYNJ2*
rs350289	*SYNJ2*
rs350292	*SYNJ2*
rs3818457	*SYNJ2*
rs4333441	*SYNJ2*
rs6455949	*SYNJ2*
rs6455990	*SYNJ2*
rs6904665	*SYNJ2*
rs750997	*SYNJ2*
rs751873	*SYNJ2*
rs7768038	*SYNJ2*
rs9295289	*SYNJ2*
rs9356200	*SYNJ2*
rs9365674	*SYNJ2*
rs9365723	*SYNJ2*
rs9365724	*SYNJ2*
rs9457002	*SYNJ2*
rs9458975	*SYNJ2*
rs9459056	*SYNJ2*
rs9459093	*SYNJ2*
rs9459154	*SYNJ2*
rs9654570	*SYNJ2*
rs999613	*SYNJ2*
rs10744720	*TPI1*
rs1076865	*TPI1*
rs2238114	*TPI1*
